# Enantioselective Intramolecular Iridium-Catalyzed Cyclopropanation of α-Carbonyl Sulfoxonium Ylides

**DOI:** 10.1021/acs.orglett.2c03396

**Published:** 2022-11-11

**Authors:** Lucas Vidal, Pan-Pan Chen, Eva Nicolas, Andrew Hackett, Craig M. Robertson, Kendall N. Houk, Christophe Aïssa

**Affiliations:** † Department of Chemistry, University of Liverpool, Crown Street, Liverpool L69 7ZD, United Kingdom; ‡ Department of Chemistry and Biochemistry, University of California, Los Angeles, California 90095, United States

## Abstract

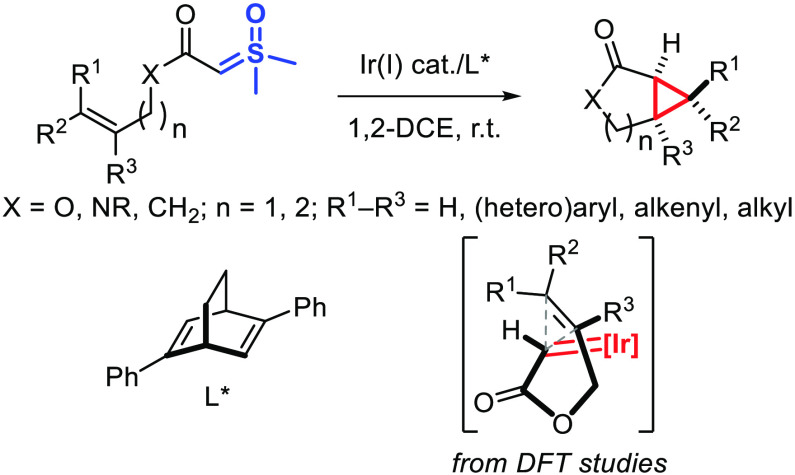

Enantioselective cyclopropanation of α-carbonyl sulfoxonium ylides (SY) has so far been limited to addition/ring closure reactions on electron-poor olefins. Herein, we report the iridium-catalyzed intramolecular cyclopropanation of SY in the presence of a chiral diene in up to 96% yield and 98% enantioselectivity. Moreover, density functional theory calculations suggest that the *re* face of the olefin preferably attacks an iridium carbene intermediate in an asynchronous concerted step that is independent of the geometry of the olefin.

The superior safety profile of α-carbonyl sulfoxonium ylides compared to that of their diazo counterpart has recently spurred the exploration of numerous metal-catalyzed reactions in which a metal-carbene has been proposed to be a pivotal intermediate.^1^ In this context, it is striking that enantioselective cyclopropanation of olefins by the intermediacy of a chiral metal-carbene, a hallmark of metal-carbene chemistry,^2^ has never been observed in metal-catalyzed reactions of α-carbonyl sulfoxonium ylides. Specifically, reports of cyclopropanation of α-carbonyl sulfoxonium ylides are limited to an arene C–H activation/cyclopropanation cascade with electron-poor allenes^3^ and enantioselective addition/ring closure on electron-poor olefins in the presence of either a chiral organocatalyst^4^ or a chiral Lewis acid (Scheme 1a).^5^ Thus, overcoming these limitations and expanding the scope of cyclopropanation of α-carbonyl sulfoxonium ylides beyond electron-poor olefins would improve our understanding of the reactivity of these ylides in homogeneous catalysis and benefit molecular science in view of the importance of cyclopropanes in drugs,^6^ natural products,^7^ and fragrances.^8^

**Scheme 1:**
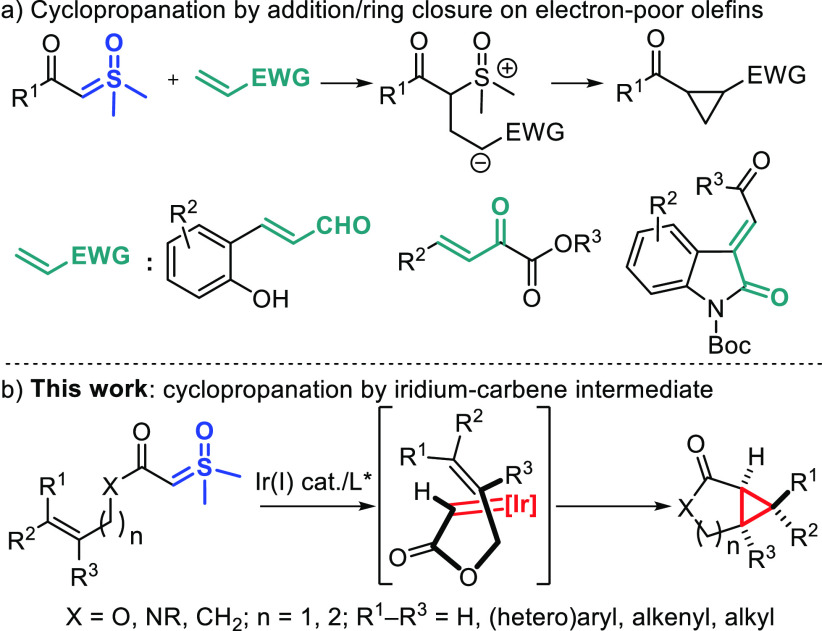
Enantioselective Cyclopropanation of α-Carbonyl Sulfoxonium Ylides

Iridium(I) complexes are versatile catalysts in a diverse set of reactions of sulfoxonium ylides such as X–H (X = B, N, O, or S) insertions^9^ and aromatic substitutions^10^ that all likely rely on an iridium carbene intermediate. We therefore hypothesized that Ir(I) catalysts would be good candidates for promoting the cyclopropanation of α-carbonyl sulfoxonium ylides with olefins that are not activated by an electron-withdrawing group. Moreover, we reasoned that chiral diene ligands would offer an ideal platform for the development of an enantioselective version of the reaction.^11^

Herein, we validate this hypothesis with the asymmetric synthesis of bicyclic lactones, lactams, and ketones by intramolecular cyclopropanation of sulfoxonium ylides (Scheme 1b). In addition, a stereochemical model supported by DFT calculations is proposed to explain how the enantioselectivity remains high regardless of the geometry of the olefin, in contrast with the known metal-catalyzed intramolecular cyclopropanations of allyl diazo acetates that have been optimized specifically for either the *E* or the *Z* olefins.^12,13^

At the beginning of our study, it became rapidly apparent during the optimization of the reaction that it was necessary to add sulfoxonium ylide **1a** slowly on a solution of the catalyst to avoid the formation of dimeric products. Under these conditions, and using [Ir(cod)Cl]_2_ (cod = cyclooctadiene) as a catalyst in 1,2-DCE (1,2-dichloroethane) at 80 °C, we obtained bicyclic lactone (±)-**2a** in 94% yield (eq 1). Other iridium and rhodium catalysts led to lower yields, and using Rh_2_(OAc)_4_ notably led to only traces of (±)-**2a** (see Table S1).
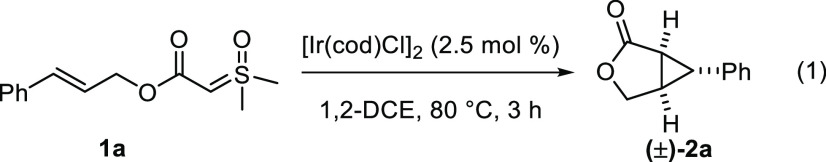
1

Moreover, during the initial optimization of the reaction, we noted that decreasing the temperature to 40 °C led to incomplete conversion after the slow addition of the substrate. Nevertheless, we reckoned that the catalyst was still active at this stage and that full conversion could be reached by longer exposure. We were pleased to verify this hypothesis and observed full conversion of **1a** to (−)-**2a** with 84% ee when commercially available (*R*,*R*)-**3** was used as the ligand (Table 1, entry 1). After other chiral dienes such as **4–8** had been examined, (*R*,*R*)-**3** remained the best ligand, and (−)-**2a** could be obtained in 90% ee when the reaction was conducted at room temperature (Table 1, entry 4 vs entries 2, 3, and 5–7).

**Table 1:**
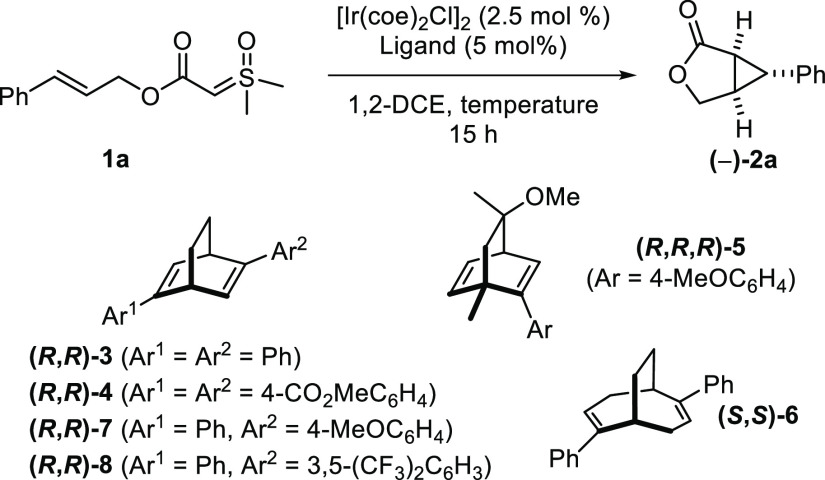
Optimization of the Enantioselectivity^a^

entry	ligand	*T* (°C)	yield^c^ (%)	ee^d^ (%)
1	(*R*,*R*)-**3**	40^b^	92	84
2	(*R*,*R*)-**4**	40^b^	80	78
3	(*R*,*R*,*R*)-**5**	40^b^	99	30
4	(*R*,*R*)-**3**	24	89^e^	90^f^
5	(*S*,*S*)-**6**	24	21	0
6	(*R*,*R*)-**7**	24	86	87
7	(*R*,*R*)-**8**	24	75^e^	75^f^

a Slow addition of a solution of **1a** (0.2 mmol) in 1,2-DCE (3 mL) to the metal catalyst and ligand in 1,2-DCE (9 mL) under N_2_ over 3 h and then stirring at the indicated temperature for 12 h. coe = cyclooctaene.

b Temperature of the heating block.

c Yield determined by ^1^H NMR of the crude with 1,3,5-trimethoxybenzene as the internal standard except where otherwise indicated.

d Enantiomeric excess of the crude material determined by HPLC.

e Yield of the isolated product.

f Enantiomeric excess of the isolated product determined by HPLC.

With these optimized conditions in hands, we examined their generality on α-carbonyl sulfoxonium ylides **1a–o** and were delighted to obtain the envisioned racemic bicyclic lactones, lactams, and ketones in 32–98% yields (Scheme 2). Thus, (hetero)aryl substituents were well tolerated [(±)-**2a–d**], as were alkenyl [(±)-**2e**] and alkyl substituents [(±)-**2k**]. Moreover, *Z* olefins led to the expected cyclopropanes with an only slight decrease in yield in the case of (±)-**2f–h** or in identical yield in the case of trisubstituted olefins that gave (±)-**2i** and (±)-**2j**. Another trisubstituted substrate **1l** gave (±)-**2l** in 70% yield. In addition to lactones, other tethers were efficient and bicyclic ketone (±)-**2m** and lactam (±)-**2n** were obtained in 98% and 86% yields, respectively. However, six-membered ring lactone (±)-**2o** could be obtained in only 32% yield.

**Scheme 2:**
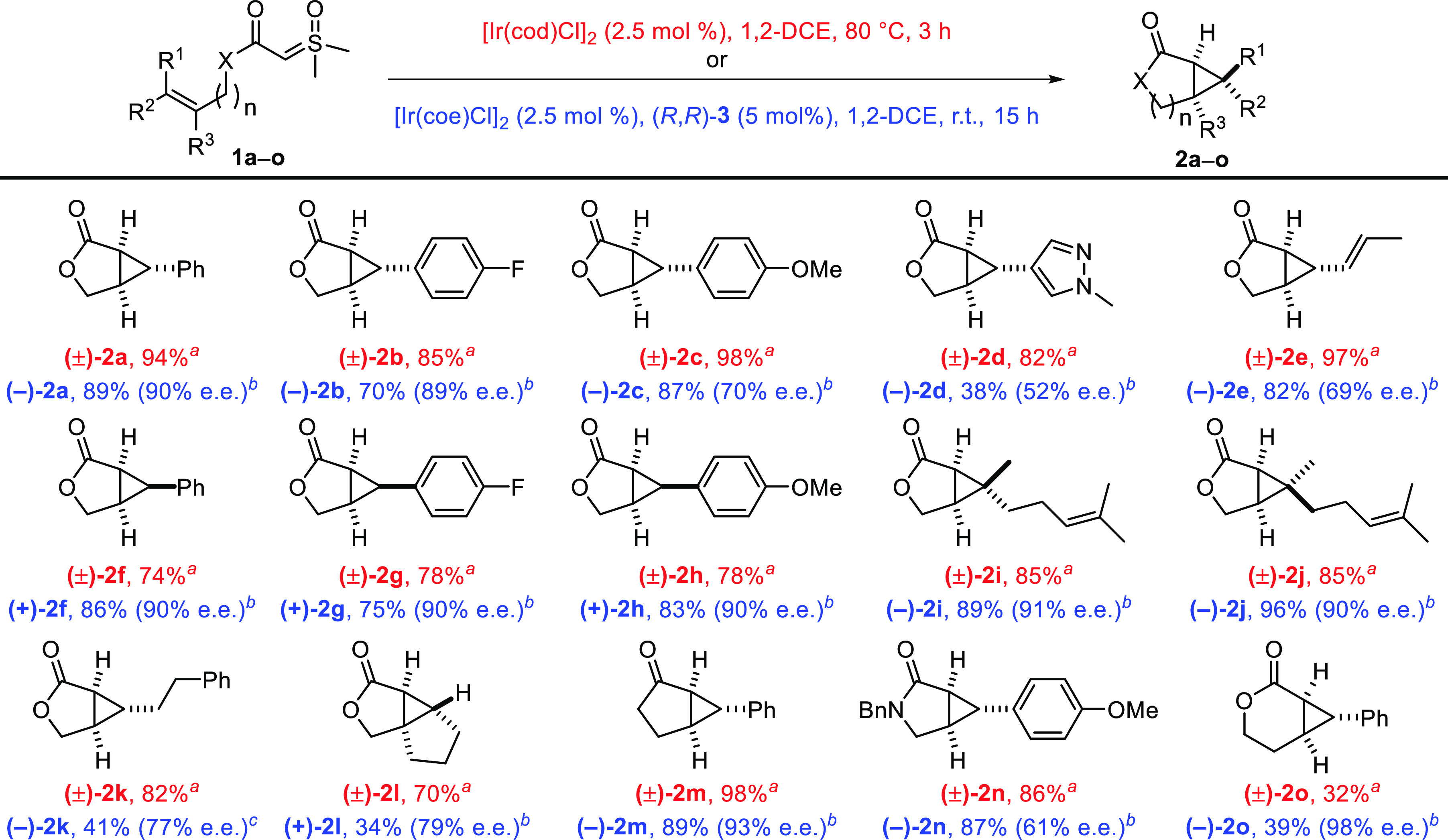
Enantioselective Intramolecular Iridium-Catalyzed Cyclopropanation of α-Carbonyl Sulfoxonium Ylides Under the conditions of eq 1. As in entry 4 of Table 1. Slow addition for 9 h and stirring for 96 h. Yields and ee’s of the isolated product.

Then, using (*R*,*R*)-**3** as the chiral ligand and under the conditions optimized for the asymmetric variant of this cyclopropanation, we obtained the enantioenriched products in 34–96% yields and 52–98% ee (Scheme 2).^14^ The best results were obtained with aryl-substituted olefins, whereas a pyrazole [(−)-**2d**], an alkenyl [(−)-**2e**], or an alkyl [(−)-**2k**] substituent was more detrimental to the enantioselectivity. Remarkably, when comparing the results of the enantioselective cyclopropanation of **1a** and its *Z* isomer **2f**, we established that the enantioselectivity remained high for both geometrical isomers of the olefin to give (−)-**2a** and (+)-**2f** with 90% ee.^15^ This observation is in strong contrast with the catalyzed reactions that have been developed to give optimal results with either the *E* or the *Z* isomer of allyl diazo acetates, but not with both (Table S2).^12,13^ Other pairs of geometrical isomers such as **1b** and **1g**, **1c** and **1h**, and **1i** and **1j** gave equally good results when treated with the chiral Ir-diene catalyst. The enantioselectivity remained high for bicyclic ketone (−)-**2m** and for six-membered lactone (−)-**2o**, but lactam (−)-**2n** was obtained with lower enantioselectivity.

Stereochemical models were evaluated by DFT calculations^16^ to understand the origins of the enantioselectivity observed in those reactions (Scheme 3). Iridium-carbenes **I** and **II** are most likely formed from **1a** and **1f**, respectively, under the reaction conditions (Scheme 3a).^9^ In the reactive conformers of **I** and **II** that connect with the transition states leading to the observed products, the C–Ir bond length [1.85 Å (**I** and **II**)] and the dihedral angle between the carbon–iridium bond and the carbonyl [θ = 287° (**I**), and θ = 309° (**II**)] are similar to those measured in an isolated iridium(I)-carbene formed from the reaction of [Ir(cod)Cl]_2_ and methyl 2-diazo-2-phenylacetate.^17^ Significantly, we found that a perpendicular approach of the olefin with respect to the iridium-carbene in **TS1** and **TS3** is favored over a parallel approach in **TS2** and **TS4**. Thus, **TS1** is favored over **TS2** by 1.3 kcal mol^–1^ in the case of *E* olefin **1a**, whereas **TS3** is favored over **TS4** by 6.0 kcal mol^–1^ in the case of *Z* olefin **1f**. In all cases, the tether is pointing toward the less sterically congested quadrant of the *C*_2_-symmetrical ligand (Scheme 3b). In addition, the C1–C2 and C1–C3 bonds of (−)-**2a** and (+)-**2f** are formed in an asynchronous concerted mechanism from **TS1** and **TS3**, respectively. In contrast, **TS2** and **TS4** are the highest-energy transition states of a two-step mechanism in which the C1–C2 bond is formed first to give intermediates **III** and **IV**, before eventually leading to the minor enantiomers through **TS5** and **TS6**. Moreover, a distortion–interaction analysis^18,19^ shows that the enantioselectivity mainly arises from a greater distortion in the substrate in least favored transition states **TS2** and **TS4** (Table 2). The origins of the substrate distortion were investigated through independent gradient model analysis.^20^ It revealed a stabilizing π interaction between the C1–H bond of the substrate and one of the phenyl rings of the ligand in **TS1–TS4**. However, maintaining that favorable C–H···π interaction in **TS2** and **TS4** comes at the cost of greater steric hindrance,^21^ and hence greater distortion, within the substrate. Overall, the DFT calculations suggest that the *re* face of the olefin is attacked preferentially in **TS1** and **TS3**, in agreement with our experimental results.

**Scheme 3:**
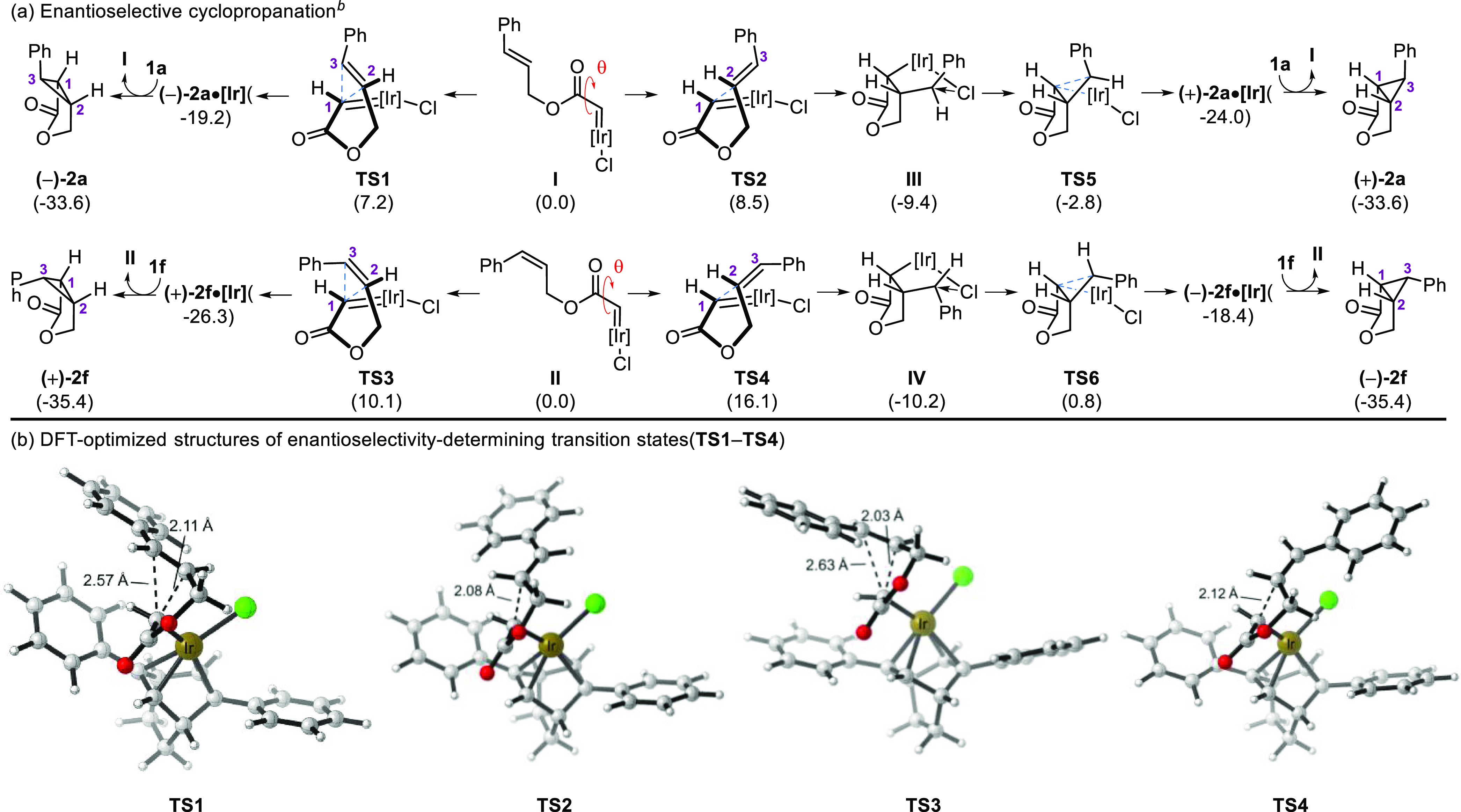
Stereochemical Model Computational method: M06/def2-TZVPP-SMD(dichloromethane)//B3LYP-D3/def2-SVP. Ligand omitted for the sake of clarity; energies (Δ*G*) are in kilocalories per mole.

**Table 2:** Distortion–Interaction Analysis^a^

	Δ*E*_dist(cat)_	Δ*E*_dist(sub)_	Δ*E*_int_	Δ*E*_act_
**TS1**	13.2	–10.9	–82.9	–80.6
**TS2**	13.8	1.0	–92.7	–77.9
ΔΔ*E*(**TS2**–**TS1**)	0.6	11.9	–9.8	2.7
**TS3**	13.0	–12.1	–78.9	–78.0
**TS4**	13.2	3.8	–88.6	–71.6
ΔΔ*E*(**TS4**–**TS3**)	0.2	15.9	–9.7	6.4

a Distortion–interaction analysis was performed at the M06/def2-TZVPP//B3LYP-D3/def2-SVP level of theory. Energies are in kilocalories per mole. The details of distortion–interaction analysis are provided in the Supporting Information.

Finally, as mentioned in the introduction, cyclopropanes are important motifs in drugs, and we could demonstrate the synthetic utility of the products obtained in this study by converting (±)-**2d** into (±)-**9**, which displays the same cyclopropane substitution pattern as (±)-**10**, a nanomolar inhibitor of hematopoietic kinase 1 (Scheme 4).^22^

**Scheme 4:**
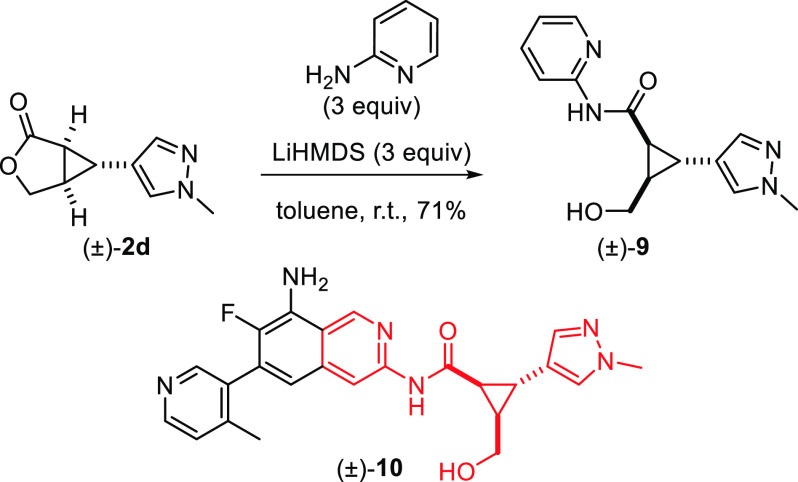
Potential Synthetic Utility

In conclusion, we have demonstrated the first example of enantioselective intramolecular cyclopropanation of α-carbonyl sulfoxonium ylides in the presence of a chiral iridium catalyst. Hence, the method enables access to enantioenriched bicyclic lactones, lactams, and ketones. This strategy expands the scope of cyclopropanation of α-carbonyl sulfoxonium ylides that had so far been limited to addition/ring closure reactions on electron-poor olefins. Moreover, DFT calculations revealed that an orthogonal approach of the *re* face of the olefin to an iridium carbene intermediate is preferred regardless of the geometry of the olefin, while the distortion of the substrate in the transition states is the main differentiating factor that determines the enantioselectivity.

## Notes

The data underlying this study are available in the published article and its online Supporting Information.

## Supporting Information Available

Experimental procedures, crystallographic data, chiral HPLC traces, NMR spectra, and details of computational studies (PDF)
