# Supplementary material for: Enantioselective Intramolecular Iridium-Catalyzed Cyclopropanation of α-Carbonyl Sulfoxonium Ylides
Source: Org Lett. 2022 Nov 11;24(46):8503–8. doi: 10.1021/acs.orglett.2c03396 (PMC9706811; doi:10.1021/acs.orglett.2c03396)
Supplement: Supplementary file 1 — ol2c03396_si_001.pdf [file ol2c03396_si_001.pdf]

Supporting Information

Enantioselective Intramolecular Iridium-Catalyzed Cyclopropanation of α-Carbonyl Sulfoxonium Ylides

Lucas Vidal,[#] Pan-Pan Chen,[§] Eva Nicolas,[#] Andrew Hackett,[#] Craig M. Robertson,[#] Kendall N. Houk,[§] and Christophe Aïssa[#,*]

[#] Department of Chemistry, University of Liverpool, Crown Street, Liverpool L69 7ZD, United Kingdom.

[§] Department of Chemistry and Biochemistry, University of California, Los Angeles, 90095, United States.

*E-mail: aissa@liverpool.ac.uk

General S2

Synthesis of sulfoxonium ylides **1a–1o** S3

Iridium-catalyzed cyclopropanation of α-carbonyl sulfoxonium ylides into **2a**–**2o** S16

1. Optimization of the cyclopropanation of sulfoxonium ylide **1a** into racemic (±)-**2a** S16
2. Representative procedure with racemic catalyst $[Ir(cod)Cl]_2$ S16
3. Synthesis of ligands **5–8** S17
4. Representative procedure with chiral catalyst $[Ir(coe)_2Cl]_2$/(*R*,*R*)-**3** S23
5. Representative procedure with chiral catalyst $[((R,R)\text{-}\mathbf{3})IrCl]_2$ S23

Synthesis of cyclopropane derivative (±)-**9** S31

X-Ray crystallography of compounds (–)-**SI-2a-Br** and (+)-**SI-2f-Br** S32

DFT calculations S39

1. Computational methods S39
2. Details of distortion-interaction analysis S39
3. Free energy diagrams of product formation S41
4. Tables of energies S47
5. Cartesian coordinates of the DFT-optimized structures S48

Copies of NMR spectra of precursors of **1a**–**1o** (new compounds) S81

Copies of NMR spectra of **1a**–**1o** S89

Copies of NMR spectra of **2a**–**2o** and **9** S129

Copies of NMR spectra of (–)-**SI-2a-Br**, and (+)-**SI-2f-Br** S163

Copies of NMR spectra of $[((R,R)\text{-}\mathbf{3})IrCl]_2$ S167

Copies of HPLC traces for ligand precursors S169

Copies of HPLC traces for compounds **2a**–**2o** S171

References S186

General

All purchased compounds were used as received. Dry 1,2-dichloroethane was purchased from Fisher Scientific (Acros 99.8%, Extra Dry, AcroSeal™). Dry THF, CH_2Cl_2 and Et_2O were obtained from an Innovative Technology PureSolv MD system. Flash chromatography: Merck silica gel 60 (230-400 mesh). NMR: the ^1H and $^{13}C\{^1H\}$ spectra were recorded on a Bruker DRX 500 or on a Bruker Avance 400 spectrometer. Chemical shifts (δ) are given in ppm and apparent splitting patterns are designated using the following abbreviations: s (singlet), d (doublet), t (triplet), q (quartet), quint (quintuplet), sept (septuplet), m (multiplet), br (broad), and the appropriate combinations (e.g. qd (quartet of doublets), dtt (doublet of triplets of triplets), br s (broad singlet)). The solvent signals were used as references for ^1H and $^{13}C\{^1H\}$ spectra ($CDCl_3$: $\delta_H = 7.26$, $\delta_C = 77.0$; DMSO-d_6: $\delta_H = 2.50$, $\delta_C = 39.5$; CD_2Cl_2 $\delta_H = 5.32$, $\delta_C = 53.4$). IR spectra were recorded on a PerkinElmer Spectrum 100 FT-IR spectrometer, and the wavenumbers ($\tilde{\nu}$) are given in cm^{-1}. HRMS determined at the University of Liverpool on Agilent 6540A Accurate-Mass Q-ToF MS with Agilent Jetstream Source (ESI); *m/z* values were calculated using the software Agilent MassHunter Qualitative Analysis Navigator for the molecular formula of the product (M) with additional positive (H^+, Na^+) or negative (Cl^-) ions. Melting points were measured on a Griffin melting point apparatus (not corrected). Elemental analyses: Elementar Vario Micro Cube instrument at University of Liverpool. Optical rotation: Bellingham Stanley ADP440+ (*c* is given in g/100 mL). HPLC: Enantiomeric excesses (ee) were determined by HPLC analyses using an Agilent Series 1200 system equipped with chiral stationary phase columns Chiralcel AD, AD-H, and OD provided by Daicel company (see specific columns and conditions).

Synthesis of sulfoxonium ylides 1a–1o

Precursors **S1b**,[1] **S1c**,[2] and **S1k**[3] are known compounds and were donated by Dr Stephanie Yip, formerly at The University of Liverpool. The preparation of the other precursors of **1a–1o** is described below in alphanumerical order.

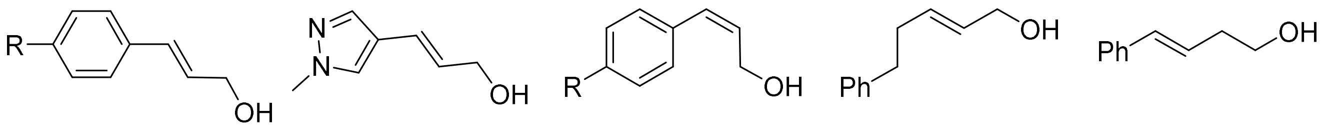

S1b (R = F) **S1c** (R = OMe) — **S2d** — **S1f** (R = H) **S2g** (R = F) **S2h** (R = OMe) — **S1k** — **S2o**

*Preparation of **S2d***

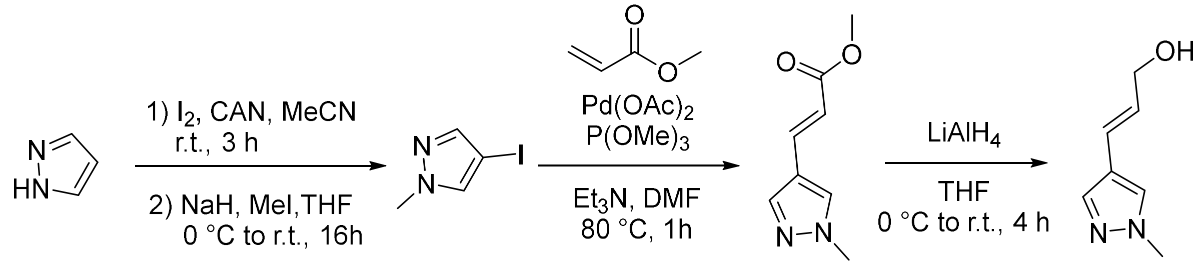

S1d **S2d**

Methyl (E)-3-(1-methyl-1H-pyrazol-4-yl)acrylate (S1d). Pyrazole (2.33 g, 34.2 mmol, 1.0 equiv) was dissolved in dry MeCN (35 mL) under nitrogen in a flame-dried round bottom flask. Iodine (5.21 g, 20.5 mmol, 0.6 equiv) was added to the solution followed by ceric ammonium nitrate (CAN) (11.26 g, 20.5 mmol, 1 equiv) that was in portions over 10 min at room temperature. After stirring the mixture for 3 hours at room temperature, the solvent was evaporated. EtOAc and chilled aqueous sodium bisulfite were added to the precipitate. The organic layer was washed with brine, dried over $MgSO_4$ and concentrated under vacuum to afford a solid that was added in portions over 10 min to a NaH (1.82 g, 60% in mineral oil, 1.5 equiv) suspension in THF (40 mL) at 0 °C under N_2. After 15 minutes at r.t., iodomethane (2.26 mL, 36.3 mmol, 1.2 equiv) was added dropwise at 0 °C. Upon completion of the addition, the reaction mixture was allowed to warm to room temperature and stirred overnight. The mixture was then quenched with water and concentrated under reduced pressure. An aqueous saturated solution of $NaHCO_3$ was added and the aqueous layer was extracted 3 times with EtOAc. The combined organic layers were dried over $MgSO_4$ and concentrated under vacuum. The residue was triturated with pentane to remove oil and afford a pale-yellow solid that was used directly in the next step without further purification. To an oven-dried flask equipped with a condenser under argon was added N-methyl iodopyrazole obtained above (1.0 g, 4.81 mmol, 1 equiv), $Pd(OAc)_2$ (22 mg, 0.01 mmol, 2 mol%), Et_3N (0.91 mL, 5.3 mmol, 1.1 equiv), methyl acrylate (1.3 mL, 14.4 mmol, 3 equiv.), $P(OMe)_3$ (23 µL, 0.19 mmol, 4 mol%) and DMF (12 mL, 0.4 M). After 2 hours at reflux, the reaction mixture was quenched with water at room temperature and extracted 3 times with EtOAc. The combined organic layers were washed with water, brine,

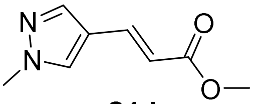

S1d

then dried over $MgSO_4$ and concentrated. Purification by silica gel column chromatography (30% EtOAc in hexane) to afford ester **S1** (327 mg, 41%) as a white solid. m.p.: 57-58° C. ^{1}H NMR (500 MHz, $CDCl_3$): δ 7.68 (s, 1H), 7.55 (d, J = 16.4 Hz, 1H), 7.54 (s, 1H), 6.16 (d, J = 16.0 Hz, 1H), 3.91 (s, 3H), 3.77 (s, 3H). ^{13}C{^{1}H} NMR (126 MHz, $CDCl_3$): δ 167.7, 138.7, 135.2, 130.3, 118.4, 115.4, 51.5, 39.2. HRMS (ESI+): *m/z* calculated for $C_8H_{10}N_2O_2$ $[M+H]^+$: 167.0821, found: 167.0813.

(*E*)-3-(1-methyl-1H-pyrazol-4-yl)prop-2-en-1-ol (S2d). To an oven-dried Schlenk tube under N_2 was added

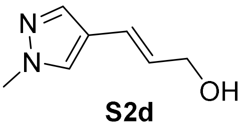

S2d

ester **S1d** (100 mg, 0.6 mmol, 1 equiv) in THF (1.2 mL, 0.5 M). $LiAlH_4$ (248 mg, 6.54 mmol, 1.25 equiv) was carefully added in portions at 0 °C. The reaction mixture gradually warmed to room temperature and stirred for 4 hours. The reaction was quenched with dropwise addition of 1 M HCl solution at 0 °C. The mixture was extracted 3 times with EtOAc, and the combined organic layers were washed with brine, dried over $MgSO_4$ and concentrated to afford **S2d** as an oil (53 mg, 63%) that was used directly in the next step without further purification. ^{1}H NMR (500 MHz, $CDCl_3$): δ 7.52 (s, 1H), 7.33 (s, 1H), 6.41 (dt, J = 15.9, 1.4 Hz, 1H), 6.07 (dt, J = 15.9, 6.0 Hz, 1H), 4.22 (dd, J = 6.0, 1.4 Hz, 2H), 3.85 (s, 3H). ^{13}C{^{1}H} NMR (126 MHz, $CDCl_3$): δ 137.2, 127.8, 126.6, 121.3, 120.0, 63.7, 38.9. HRMS (ESI+): *m/z* calculated for $C_7H_{10}N_2O$ $[M+H]^+$: 139.0871, found: 139.0866**.**

Preparation of ***S1f**, **S2g**, and **S2h***

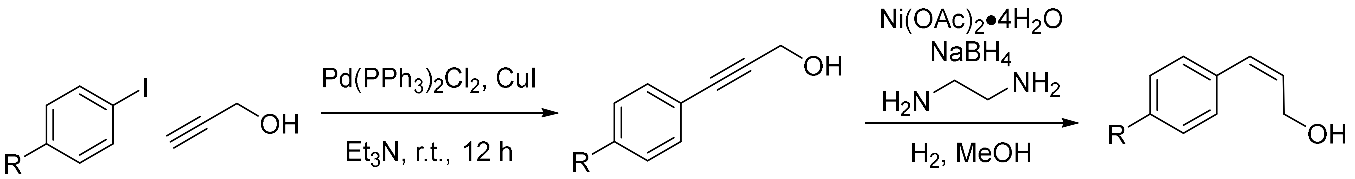

S1g (R = F)
S1h (R = OMe)

S1f (R = H)
S2g (R = F)
S2h (R = OMe)

(*Z*)-3-phenylprop-2-en-1-ol (S1f). According to a reported procedure,[4] $NaBH_4$ (25 mg, 0.66 mmol, 0.2 equiv)

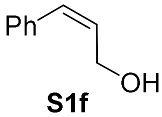

S1f

was added portionwise to a stirred solution of $Ni(OAc)_2•4H_2O$ (164 mg, 0.66 mmol, 0.2 equiv) in absolute MeOH (7 mL) at 0 °C and the resultant black mixture was stirred and allowed to warm to r.t. over 15 min. Ethylenediamine (134 µL, 2.00 mmol, 0.6 equiv) and a solution of 3-phenylprop-2-yn-1-ol (440 mg, 3.33 mmol, 1 equiv) in MeOH (4 mL) were added in succession at r.t. and the resultant suspension was vigorously stirred under hydrogen gas (balloon) at r.t. for 5 hours. The reaction mixture was filtered through Celite® (eluent EtOAc) and the filtrate was concentrated in vacuo. The crude residue was then purified by passing through a plug of silica using 10 to 40% hexane in EtOAc as eluent to yield 282 mg (63%) of a colorless oil. ^{1}H NMR (500 MHz, $CDCl_3$): δ 7.38 –7.33 (m, 2H), 7.30 –7.25 (m, 1H), 7.23 –7.19 (m, 2H), 6.57 (dt, J = 11.7, 1.5 Hz, 1H), 5.87 (dt, J = 11.8, 6.4 Hz, 1H), 4.43 (dd, J = 6.4 , 1.7 Hz, 2H), 2.14 (s, 1H(OH)); in agreement with literature data.[2]

3-(4-fluorophenyl)prop-2-yn-1-ol (S1g). $Pd(PPh_3)_2Cl_2$ (126 mg, 0.18 mmol, 2 mol%) and CuI (69 mg, 0.36 mmol, 4 mol%) were suspended in Et_3N (36 mL) under argon in an oven-dried round-bottom flask. Subsequently, 4-fluoro-iodobenzene (2.0 g, 9.0 mmol, 1 equiv) was added, followed by propargyl alcohol (0.57 mL, 9.9 mmol, 1.1 equiv) and the reaction was stirred at room temperature for 12 h. A saturated solution of NH_4Cl was then added, and the reaction mixture was partitioned between EtOAc and water. The aqueous phase was extracted twice with EtOAc, and the combined organic layers were washed in succession with a saturated solution of NH_4Cl and brine. The combined organic layers were then dried with $MgSO_4$, filtered and concentrated under vacuum. The crude residue was passed through a plug of silica (20% EtOAc in hexane) as eluent to give **S1** (1.41 g) as a yellow oil and used in the next step without further purification. ^{1}H NMR (500 MHz, $CDCl_3$): δ 7.44–7.39 (m, 2H), 7.05–6.96 (m, 2H), 4.49 (s, 2H); in agreement with literature data.[5]

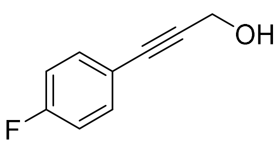

S1g

3-(4-methoxyphenyl)prop-2-yn-1-ol (S1g). This compound was obtained as a yellow oil (1.57 g) from 4-methoxy-iodobenzene (2.0 g, 8.54 mmol, 1 equiv) by following the procedure described for the preparation of **S1g** and used in the next step without further purification. ^{1}H NMR (500 MHz, $CDCl_3$): δ 7.41–7.35 (m, 2H), 6.90–6.71 (m, 2H), 4.48 (s, 2H), 3.81 (s, 3H); in agreement with literature data.[1]

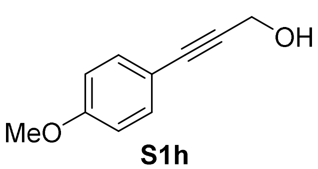

S1h

(*Z*)-3-(4-fluorophenyl)prop-2-en-1-ol (S2g). This compound was obtained as a colorless oil (410 mg, 81% over two steps) from **S1g** by following the procedure described for the preparation of **S1f**. ^{1}H NMR (500 MHz, $CDCl_3$): δ 7.22–7.17 (m, 2H), 7.07–7.00 (m, 2H), 6.54 (d, J = 11.7 Hz, 1H), 5.90–5.83 (m, 1H), 4.41 (dd, J = 6.5, 1.5 Hz, 2H); in agreement with literature data.[2]

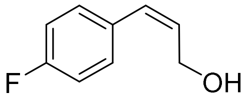

S2g

(*Z*)-3-(4-methoxyphenyl)prop-2-en-1-ol (S2h). This compound was obtained as a colorless oil (1.02 g, 65% over two steps) from **S2g** by following the procedure described for the preparation of **S1f**. ^{1}H NMR (500 MHz, $CDCl_3$): δ 7.16 (d, J = 8.6 Hz, 2H), 6.88 (d, J = 8.7 Hz, 2H), 6.51 (d, J = 11.7 Hz, 1H), 5.83–5.73 (m, 1H), 4.43 (dd, J = 6.4, 1.4 Hz, 2H), 3.82 (s, 3H); in agreement with literature data.[2]

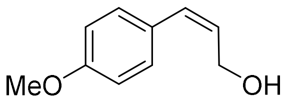

S2h

Preparation of ***S2o***

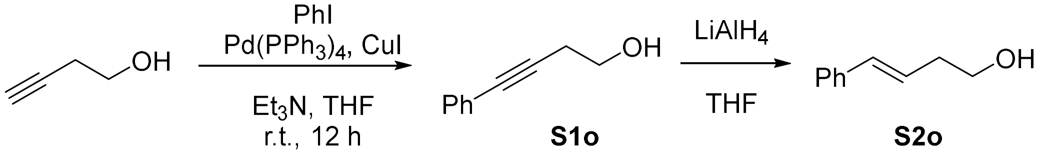

4-phenylbut-3-yn-1-ol (S1o). Under argon, iodobenzene (5.96 mL, 53.5 mmol, 1.5 equiv) and 3-Butyn-1-ol (2.50 g, 35.7 mmol, 1 equiv) were dissolved in THF (18 mL). Et_3N (71 mL), CuI (136 mg, 0.71 mmol, 2 mol%) and $Pd(PPh_3)_4$ (412 mg, 0.36 mmol, 1 mol%) were added in

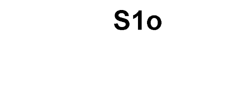

S1o

succession to the reaction flask. After stirring overnight at room temperature, the reaction mixture was filtered through Celite® (elution with EtOAc) and the solvents were evaporated. The crude residue was purified by silica gel column chromatography (10 to 20% EtOAc in hexane) to afford alkyne **S1o** (4.18 g, 80%) as a colorless oil. ^{1}H NMR (500 MHz, $CDCl_3$): δ 7.45 – 7.39 (m, 2H), 7.32 – 7.27 (m, 3H), 3.82 (q, J = 6.3 Hz, 2H), 2.70 (t, J = 6.2 Hz, 2H), 1.81 (t, J = 6.4 Hz, 1H); in agreement with literature data.[6]

(*E*)-4-phenylbut-3-en-1-ol (S2o). To a suspension of $LiAlH_4$ (3.26 g, 85.8 mmol, 3 equiv) in THF (37 mL)

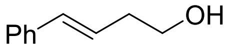

S2o

at 0 °C under nitrogen was added dropwise a solution of **S1o** (4.18 g, 28.6 mmol, 1 equiv) in THF (37 mL). The resulting mixture was brought to reflux overnight. The reaction was then cooled to 0 °C, and quenched by the sequential addition of water (2 mL), a 15% aqueous solution of NaOH (2 mL) and additional water (6 mL). The mixture was extracted with ether (3 × 15 mL), and the combined organic extracts were washed with brined and dried over $MgSO_4$, filtered and concentrated under reduced pressure. The crude residue was purified by silica gel column chromatography (30% EtOAc in hexanes) to afford homoallylic alcohol S# (3.65 g, 86%) as a yellow solid. ^{1}H NMR (500 MHz, $CDCl_3$): δ 7.36 (dd, J = 8.2, 1.0 Hz, 2H), 7.31 (t, J = 7.6 Hz, 2H), 7.25 – 7.19 (m, 1H), 6.51 (d, J = 15.9 Hz, 1H), 6.21 (dt, J = 15.8, 7.2 Hz, 1H), 3.77 (q, J = 6.1 Hz, 2H), 2.50 (qd, J = 6.4, 1.4 Hz, 2H), 1.46 (t, J = 5.8 Hz, 1H); in agreement with literature data.[7]

*Preparation of **1a–1o***

Compounds **1a**–**1h**, **1j,** and **1o** were prepared according to the scheme below. Compounds **1i**, **1k**, and **1l–1n** were prepared by alternative methods that are described in the following pages. Methods and characterization data for **1a**–**1o** are presented in alphanumerical order.

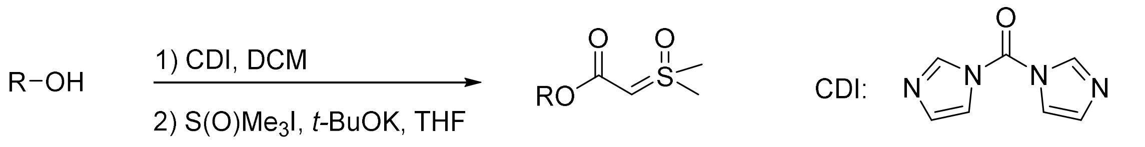

1a–h, 1j, 1o

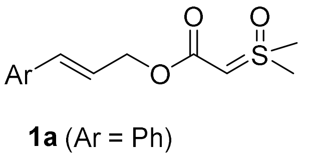

1a (Ar = Ph)
1b (Ar = 4-F-C_6H_4)
1c (Ar = 4-MeO-C_6H_4)

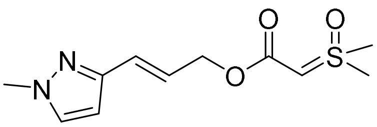

1d

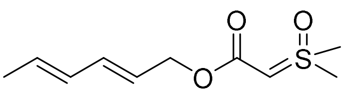

1e

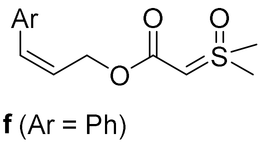

1f (Ar = Ph)
1g (Ar = 4-F-C_6H_4)
1h (Ar = 4-MeO-C_6H_4)

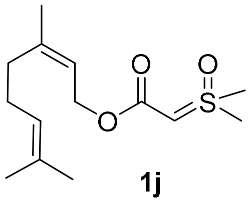

1j

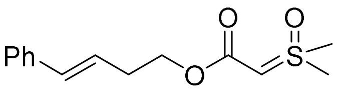

1o

Representative procedure – **(*E*)-3-phenylallyl 2-(dimethyl(oxo)-λ^6-sulfaneylidene) acetate (1a)**. A flame-

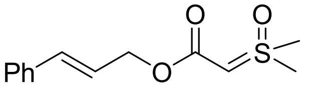

1a

dried 1L flask with a magnetic stirring bar was charged with CDI (3.63 g, 22.36 mmol, 1.5 equiv) in DCM (60 mL) under nitrogen. The flask was cooled in an ice-water bath. A solution of (*E*)-cinnamyl alcohol (2.00 g, 14.9 mmol, 1 equiv) in 15 mL DCM was slowly added over 5 min and the mixture was stirred for 2 h. Water (60 mL) was added and the mixture was then poured into a separatory funnel. The aqueous phase was extracted with DCM (3 x 30 mL), the combined organic phases were washed with water, then brine, dried over $MgSO_4$, filtered, and the solvent evaporated under vacuum. A second flame-dried round bottom flask, under nitrogen and protected from light with aluminium foil, was charged with trimethylsulfoxonium iodide (14.75 g, 67.1 mmol, 3 equiv) suspended in dry THF (36 mL). Then, *t*BuOK (7.52 g, 67.1 mmol, 3 equiv) was added and the mixture was stirred at reflux for 2 h. After cooling to 0 °C, a solution of the crude carbamate dissolved in THF (9 mL) was added over 5 min and the reaction mixture was stirred overnight at room temperature. The mixture was filtered through a plug of Celite® (elution DCM) and all volatiles were removed under vacuum. Purification by column chromatography (EtOAc) gave **1a** as white solid (1.59 g (42%)). m.p.: 92-94 °C. ^{1}H NMR (500 MHz, $CDCl_3$): *δ* 7.41 – 7.36 (m, 2H), 7.33 – 7.28 (m, 2H), 7.26 – 7.20 (m, 1H), 6.64 (d, *J* = 15.9 Hz, 1H), 6.32 (dt, *J* = 15.6, 6.2 Hz, 1H), 4.72 (br s, 1H), 4.00 (br s, 1H), 3.40 (s, 6H). $^{13}C\{^1H\}$ NMR (126 MHz, $CDCl_3$): *δ* {167.2}, 136.6, 133.0, 128.5 (2C), 127.7, 126.6 (2C), 124.8, 63.4, 54.8, 42.4 (2C); the resonance placed in bracket is not visible but inferred from HMBC. IR (neat): $\tilde{\nu}$ = 1620 (s) (C=O), 1176 (s) (S=O) cm^{-1}. HRMS (ESI+): *m/z* calculated for $C_{13}H_{16}O_3S$ $[M+Na]^+$: 275.0712, found: 275.0718.

(*E*)-3-(4-fluorophenyl)allyl 2-(dimethyl(oxo)-λ^6-sulfaneylidene) acetate (1b). This compound was

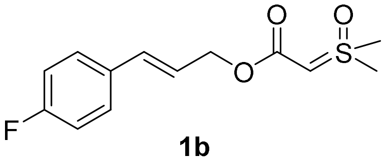

1b

obtained (152 mg (37%)) after purification by flash chromatography (EtOAc) from **S1b** (228 mg, 1.5 mmol) by following the representative procedure used to prepared compound **1a**. White solid; m.p: 102-104 °C. ^{1}H NMR (500 MHz, $CDCl_3$): *δ* 7.38-7.31 (m, 2H), 7.03-6.96 (m, 2H), 6.60 (d, *J* = 15.5 Hz, 1H), 6.23 (dt, *J* = 15.9, 6.3 Hz, 1H), 4.70 (d, *J* = 5.4 Hz, 2H), 4.00 (s, 1H), 3.40 (s, 6H). $^{13}C\{^1H\}$ NMR (125 MHz, $CDCl_3$): *δ* {167.0}, 162.3 (d, *J* = 246.9 Hz), 132.7 (d, *J* = 2.8 Hz), 131.8, 128.0 (d, *J* = 7.8 Hz, 2C), 124.5, 115.4 (d, *J* = 21.4 Hz, 2C), 63.2, 55.2, 42.3 (2C); the resonance placed in bracket is not visible but inferred from HMBC. ^{19}F NMR (471 MHz, $CDCl_3$) δ -114.26. IR (neat): $\tilde{\nu}$ = 1620 (s) (C=O), 1180 (s) (S=O) cm^{-1}. HRMS (ESI+): *m/z* calculated for $C_{13}H_{15}FO_3S$ $[M+Na^+]$: 293.0618, found: 293.0614.

(*E*)-3-(4-methoxyphenyl)allyl 2-(dimethyl(oxo)-λ^6-sulfaneylidene) acetate (1c). This compound was

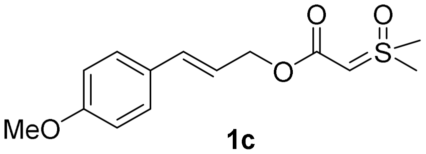

1c

obtained (472 mg (27%)) after purification by flash chromatography (5 to 25% EtOAc in DCM) from **S1c** (1.00 g, 6.09 mmol) by following the representative procedure used to prepared compound **1a**. Pale-yellow solid; m.p. 86-89 °C. ^{1}H NMR (500 MHz, $CDCl_3$): *δ* 7.32 (d, *J* = 8.7 Hz, 2H), 6.84 (d, *J* = 8.8 Hz, 2H), 6.58

(d, J = 15.8 Hz, 1H), 6.18 (dt, J = 13.2, 6.4 Hz, 1H), 4.69 (s, 2H), 3.99 (s, J = 24.6 Hz, 1H), 3.80 (s, J = 8.4 Hz, 3H), 3.39 (s, 6H). $^{13}C\{^1H\}$ NMR (126 MHz, $CDCl_3$): δ 167.1, 159.3, 132.8, 129.3, 127.8 (2C), 122.4, 113.9 (2C), 63.6, 55.2, 54.9, 42.4 (2C). IR (neat): $\tilde{\nu}$ = 1605 (s) (C=O), 1176 (s) (S=O) cm^{-1}. HRMS (ESI+): *m/z* calculated for $C_{14}H_{18}O_4S$ $[M+H]^+$: 283.0999, found: 283.0995.

(*E*)-3-(1-methyl-1H-pyrazol-4-yl)allyl 2-(dimethyl(oxo)-λ⁶-sulfaneylidene) acetate (1d). This compound

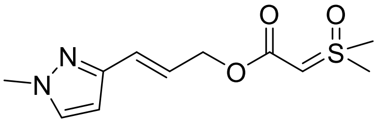

1d

was obtained (1.01 g (61%)) from **S2d** (536 mg, 3.88 mmol) by following the representative procedure used to prepared compound **1a**. Note that purification by flash chromatography (2% MeOH in DCM) initially gave a material that contained a small amount of impurities, and this material was dissolved in DCM in a small tube and Et_2O was gently layered at the surface of the DCM. Precipitation occurred after 4 h standing at r.t., and compound **1d** was obtained in satisfactory purity after washing the precipitate with Et_2O and drying under vacuum. White fluffy solid; m.p. 90-92 °C. ^1H NMR (500 MHz, $CDCl_3$): δ 7.53 (s, 1H), 7.34 (s, 1H), 6.45 (d, J = 15.9 Hz, 1H), 6.10 – 5.96 (m, 1H), 4.62 (br s, 2H), 3.98 (br s, 1H), 3.86 (s, 3H), 3.38 (s, 6H). $^{13}C\{^1H\}$ NMR (126 MHz, $CDCl_3$): δ 167.1, 137.4, 127.9, 123.5, 122.6, 120.0, 63.6, 54.9, 42.4 (2C), 38.9. IR (neat): $\tilde{\nu}$ = 1619 (s) (C=O), 1177 (s) (S=O) cm^{-1}. HRMS (ESI+): *m/z* calculated for $C_{11}H_{16}N_2O_3S$ $[M+H]^+$: 257.0954, found: 257.0952.

(2*E*,4*E*)-hexa-2,4-dien-1-yl 2-(dimethyl(oxo)-λ⁶-sulfaneylidene) acetate (1e). This compound was obtained

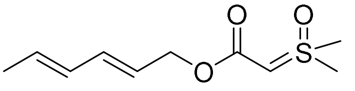

1e

(519 mg (47%)) after purification by flash chromatography (50% EtOAc in DCM to 100% EtOAc) from (2*E*,4*E*)-hexa-2,4-dien-1-ol (500 mg, 5.1 mmol) by following the representative procedure used to prepared compound **1a**. White solid; m.p.: 70-71 °C. ^1H NMR (500 MHz, $CDCl_3$): δ 6.23 (dd, J = 15.1, 10.5 Hz, 1H), 6.05 (ddd, J = 14.8, 10.5, 1.4 Hz, 1H), 5.79 – 5.58 (m, 2H), 4.55 (s, 2H), 3.96 (s, 1H), 3.38 (s, 5H), 1.75 (d, J = 6.7 Hz, 3H). $^{13}C\{^1H\}$ NMR (126 MHz, $CDCl_3$): δ 167.0, 133.8, 130.7, 130.4, 125.2, 63.3, 54.8, 42.4 (2C), 18.1. IR (neat): $\tilde{\nu}$ = 1623 (s) (C=O), 1172 (s) (S=O) cm^{-1}. HRMS (ESI+): *m/z* calculated for $C_{10}H_{16}O_3S$ $[M+H]^+$: 217.0893, found: 217.0898.

(*Z*)-3-phenylallyl 2-(dimethyl(oxo)-λ⁶-sulfaneylidene) acetate (1f). This compound was obtained (145 mg

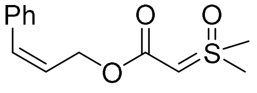

1f

(38%)) after purification by flash chromatography (50% DCM in EtOAc to 100% EtOAc) from **S1f** (200 mg, 1.5 mmol) by following the representative procedure used to prepared compound **1a**. White solid; m.p.: 86-89 °C. ^1H NMR (500 MHz, $CDCl_3$): δ 7.37 – 7.31 (m, 2H), 7.27 –7.21 (m, 3H), 6.61 (d, J = 11.8 Hz, 1H), 5.84 (dt, J = 11.5, 6.5 Hz, 1H), 4.83 (br s, 2H), 4.00 (br s, 1H), 3.37 (s, 6H). $^{13}C\{^1H\}$ NMR (126 MHz, $CDCl_3$): δ 167.1, 136.3, 131.9, 128.7 (2C), 128.3 (2C), 127.4, 127.2, 59.8, 55.0, 42.3 (2C). IR (neat): $\tilde{\nu}$ = 1633 (s) (C=O), 1162 (s) (S=O) cm^{-1}. HRMS (ESI+): *m/z* calculated for $C_{13}H_{16}O_3S$ $[M+H]^+$: 253.0893, found 253.0893.

(*Z*)-3-(4-fluorophenyl)allyl 2-(dimethyl(oxo)-λ⁶-sulfaneylidene) acetate (1g). This compound was obtained (310 mg (16%)) after purification by flash chromatography (0 to 15% EtOAc in DCM) from **S2g** (1.15 g, 7.6 mmol) by following the representative procedure used to prepared compound **1a**. Colorless oil (small impurities are visible in NMR). ^{1}H NMR (500 MHz, $CDCl_3$): *δ* 7.24 – 7.18 (m, 2H), 7.06 – 6.99 (m, 2H), 6.56 (d, *J* = 11.7 Hz, 1H), 5.83 (dt, *J* = 11.8, 6.6 Hz, 1H), 4.79 (d, *J* = 6.3 Hz, 2H), 3.98 (br s, 1H), 3.38 (s, 6H). ^{13}C{^{1}H} NMR (126 MHz, $CDCl_3$): *δ* {166.7}, 162.0 (d, *J* = 247.0 Hz), 132.4 (d, J = 2.1 Hz), 131.0, 130.4 (d, *J* = 8.0 Hz, 2C), 127.2, 115.2 (d, *J* = 21.5 Hz, 2C), 59.7, 55.1, 42.5 (2C); the resonance placed in bracket is not visible but inferred from HMBC. ^{19}F NMR (471 MHz, $CDCl_3$): *δ* -114.54. IR (neat): $\tilde{\nu}$ = 1631 (s) (C=O), 1177 (s) (S=O) cm^{-1}. HRMS (ESI+): *m/z* calculated for $C_{13}H_{15}FO_3S$ [M+H^{+}]: 271.0799, found: 271.0801.

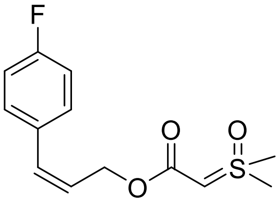

1g

(*Z*)-3-(4-methoxyphenyl)allyl 2-(dimethyl(oxo)-λ⁶-sulfaneylidene) acetate (1h). This compound was obtained (280 mg (16%)) after purification by flash chromatography (5 to 20% EtOAc in DCM) from **S2h** (1.02 g, 6.24 mmol) by following the representative procedure used to prepared compound **1a**. Pale-yellow solid; m.p.: 58-60 °C. ^{1}H NMR (500 MHz, $CDCl_3$): *δ* 7.19 (d, *J* = 8.6 Hz, 2H), 6.87 (d, *J* = 8.7 Hz, 2H), 6.55 (d, *J* = 11.7 Hz, 1H), 5.80 – 5.71 (m, 1H), 4.83 (d, *J* = 5.2 Hz, 2H), 3.99 (br s, 1H), 3.81 (s, 3H), 3.38 (s, 6H). ^{13}C{^{1}H} (126 MHz, $CDCl_3$): *δ* {167.0}, 158.8, 131.6, 130.1 (2C), 129.0, 125.6, 113.7 (2C), 60.0, 55.3, {55.0}, 42.5 (2C); the resonances placed in bracket are not visible but inferred from HMBC. IR (neat): $\tilde{\nu}$ = 1628 (s) (C=O), 1170 (s) (S=O) cm^{-1}. HRMS (ESI+): *m/z* calculated for $C_{14}H_{18}O_4S$ $[M+H]^{+}$: 283.0999, found: 283.0994.

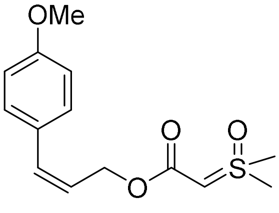

1h

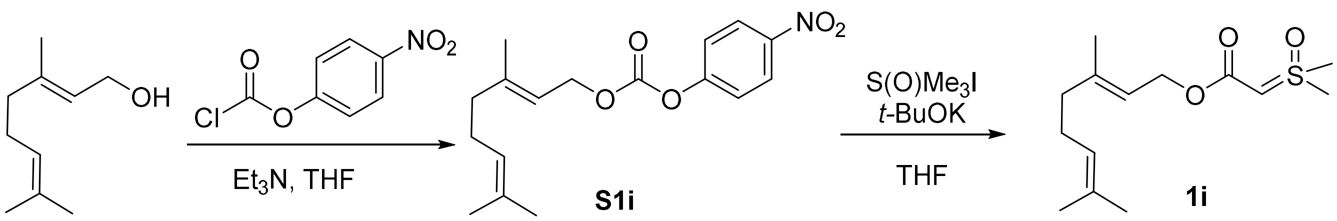

Representative procedure - (*E*)-3,7-dimethylocta-2,6-dien-1-yl (4-nitrophenyl) carbonate (S1i). 4-Nitrophenyl chloroformate (4.65 g, 25.4 mmol, 1 equiv) was dissolved in dry DCM (75 mL) in a flame dry two neck round bottom flask. A solution of geraniol (4.0 mL, 23.1 mmol, 1 equiv) and NEt_3 (3.6 mL, 25.4 mmol, 1.1 equiv) in dry DCM (38 mL) was added dropwise at 0 °C. After full addition, the reaction mixture was stirred overnight at room temperature. Then, 100 mL of water were added, and the two layers were separated. The aqueous layer was extracted 3 times with DCM. The combined organic layers were washed with brine, dried with $MgSO_4$, and filtered before concentration under vacuum. Purification by flash chromatography (5% EtOAc in hexane) gave **S1i** (, 65%) as light-yellow solid. m.p: 36-38 °C. ^{1}H NMR (500 MHz, $CDCl_3$): δ 8.26-8.21 (m, 2H), 7.38-7.34 (m, 2H), 5.43 (td, *J* = 7.33, 1.24 Hz, 1H), 5.06 (tt, *J* =

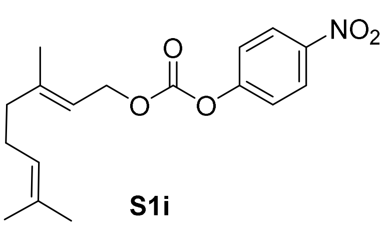

S1i

6.73, 1.30 Hz, 1H), 4.78 (d, J = 7.44 Hz, 2H), 2.14-2.04 (m, 4H), 1.75 (s, 3H), 1.66 (s, 3H), 1.58 (s, 3H); ^{13}C NMR (125 MHz, $CDCl_3$): δ 155.7, 152.5, 145.3, 144.7, 132.0, 125.3 (2C), 123.6 (2C), 121.8, 116.8, 66.1, 39.6, 26.2, 25.7, 17.7, 16.6. LRMS (ESI+): *m/z* (intensity) calculated for $C_{17}H_{21}NO_5$ [M+Na]$^+$: 320.1 (5%), found: 320.1. HMRS could not be obtained.

Representative procedure - (*E*)-3,7-dimethylocta-2,6-dien-1-yl 2-(dimethyl(oxo)-λ^6-sulfaneylidene) acetate (1i). Under nitrogen, trimethylsulfoxonium iodide (4.4 g, 19.8 mmol, 3.1 equiv) was suspended in dry THF (28 mL) in a flame dried two neck round bottom flask protected from light with aluminium foil. Potassium *tert*-butoxide (2.2 g, 19.8 mmol, 3.1 equiv) was added and the mixture was stirred at reflux for 2 hours. After cooling at 0 °C, 4-nitrophenyl carbonate **S1i** (2.0 g, 6.3 mmol, 1.0 equiv) in THF (15 mL) was added dropwise to the mixture. After full addition, the reaction mixture was stirred another hour at room temperature and then filtered through a plug of celite before all volatiles were removed under vacuum. Purification by flash chromatography (20% acetone in DCM) gave **1i** (411 mg, 24%) as a white solid. m.p.: 46-48 °C. ^{1}H NMR (500 MHz, $CDCl_3$): δ 5.33 – 5.23 (m, 1H), 5.01 (t, J = 6.6 Hz, 1H), 4.48 (br s, 2H), 3.93 (br s, 1H), 3.31 (s, 6H), 2.06-1.99 (m, 2H), 1.98-1.92 (m, 2H), 1.62 (s, 3H), 1.60 (s, 3H), 1.52 (s, 3H). ^{13}C{^{1}H} NMR (125 MHz, $CDCl_3$): δ {167.2}, 140.6, 131.4, 123.6, 119.4, 59.3, 55.3, 41.8 (2C), 39.3, 26.1, 25.4, 17.4, 16.2; the resonance placed in bracket is not visible but inferred from HMBC. IR (neat): $\tilde{\nu}$ = 1631 (s) (C=O), 1172 (s) (S=O) cm^{-1}. HRMS (ESI+): *m/z* calculated for $C_{14}H_{24}O_3S$ [M+H]$^+$: 273.1524, found: 273.1519.

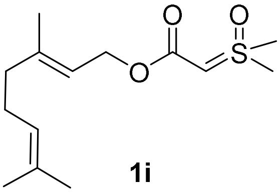

1i

(*Z*)-3,7-dimethylocta-2,6-dien-1-yl 2-(dimethyl(oxo)-λ^6-sulfaneylidene) acetate (1j). This compound was obtained (141 mg (16%)) after purification by flash chromatography (20% acetone in DCM) from nerol (500 mg, 3.24 mmol) by following the representative procedure used to prepared compound **1a**. Colorless oil. ^{1}H NMR (500 MHz, CD_2Cl_2): δ 5.41 – 5.28 (m, 1H), 5.16 – 5.06 (m, 1H), 4.49 (br s, 2H), 3.94 (br s, 1H), 3.34 (s, 6H), 2.19 – 2.01 (m, 4H), 1.75 (s, 3H), 1.68 (s, 3H), 1.61 (s, 3H). ^{13}C{^{1}H} NMR (126 MHz, CD_2Cl_2): δ 167.6, 141.4, 132.3, 124.2, 121.0, 59.5, 55.7, 42.5 (2C), 32.5, 27.1, 25.8, 23.6, 17.8. IR (neat): $\tilde{\nu}$ = 1632 (s) (C=O), 1178 (s) (S=O) cm^{-1}. HRMS (ESI+): *m/z* calculated for $C_{14}H_{24}O_3S$ [M+H]$^+$: 273.1524, found: 273.1525.

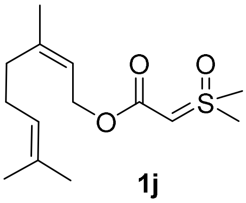

1j

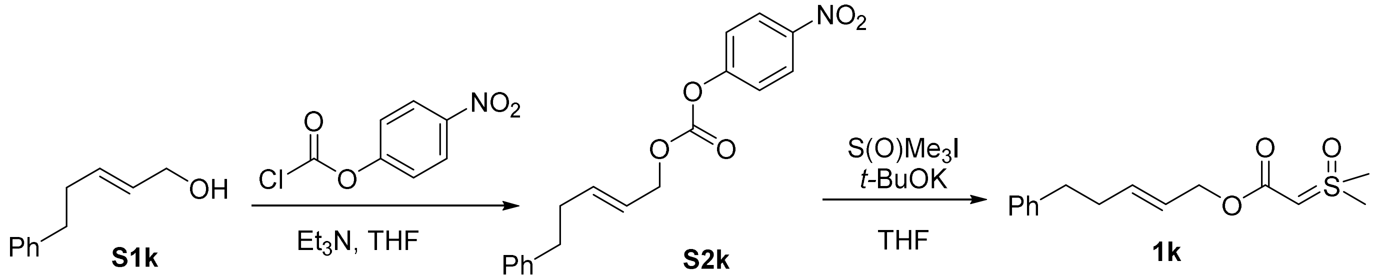

(*E*)-4-nitrophenyl (5-phenylpent-2-en-1-yl) carbonate (S2k). This compound was obtained (1.17 g (72%))

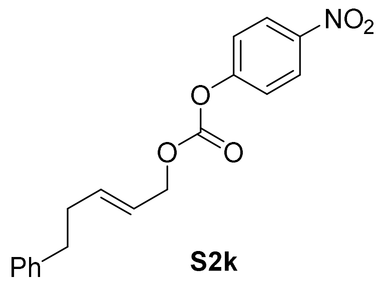

after purification by flash chromatography (7% to 10% EtOAc in hexane) from **S1k** (800 mg, 4.93 mmol) by following the representative procedure used to prepared compound **S1i**. White solid; m.p: 44–50 °C. ^{1}H NMR (500 MHz, $CDCl_3$): δ 8.30-8.25 (m, 2H), 7.41–7.35 (m, 2H), 7.32–7.27 (m, 2H), 7.23–7.17 (m, 3H), 5.99 – 5.91 (m, 1H), 5.68 (dtt, J = 15.4, 6.7, 1.4 Hz, 1H), 4.72 (dd, J = 6.8, 0.8 Hz, 2H), 2.76–2.71 (m, 2H), 2.47–2.40 (m, 2H); ^{13}C{^{1}H} NMR (125 MHz, $CDCl_3$): δ 155.5, 152.3, 145.3, 141.2, 137.7, 128.36 (2C), 128.34 (2C), 126.0, 125.2 (2C), 122.9, 121.8 (2C), 69.8, 35.0, 33.9. HRMS (ESI+): *m/z* calcd for $C_{18}H_{17}NO_5$ $[M+Na]^+$: 350.0999, found: 350.0997.

(*E*)-5-phenylpent-2-en-1-yl 2-(dimethyl(oxo)-λ^6-sulfaneylidene) acetate (1k). This compound was

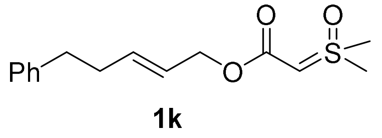

obtained (607 mg (83%)) after purification by flash chromatography (EtOAc) from **S2k** (850 mg, 2.59 mmol) by following the representative procedure used to prepared compound **1i**. White solid; m.p: 73–76 °C; ^{1}H NMR (500 MHz, $CDCl_3$): δ 7.30–7.24 (m, 2H), 7.21–7.14 (m, 3H), 5.79 (dt, J = 15.2, 6.7 Hz, 1H), 5.63 (dt, J = 15.2, 6.4 Hz, 1H), 4.49 (d, J = 6.3 Hz, 2H), 3.97 (br s, 1H), 3.36 (s, 6H), 2.70 (t, J = 7.3 Hz, 2H), 2.37 (q, J = 7.3 Hz, 2H). ^{13}C{^{1}H} NMR (125 MHz, $CDCl_3$): δ {167.2}, 141.6, 134.0, 128.3 (2C), 128.2 (2C), 125.7, 125.6, 63.3, 55.2 (br), 42.2 (2C), 35.3, 34.0; the resonance placed in bracket is not visible but inferred from HMBC. IR (neat): $\tilde{\nu}$ = 1626 (s) (C=O), 1179 (s) (S=O) cm^{-1}. HRMS (ESI+): *m/z* calcd for $C_{15}H_{20}O_3S$ $[M+H]^+$: 281.1206; found: 281.1204.

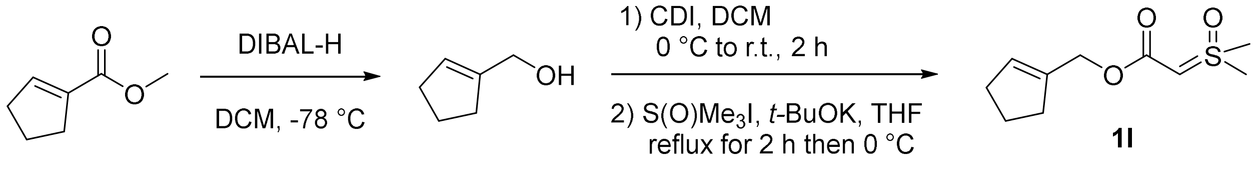

Cyclopent-1-en-1-ylmethyl 2-(dimethyl(oxo)-λ^6-sulfaneylidene) acetate (1l). To a solution of DIBAL-H

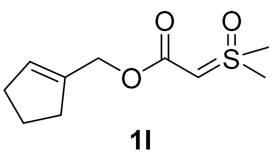

(9.1 mL, 9.11 mmol, 1M in hexanes, 2.3 equiv) in dry DCM (13.2 mL) at -78 °C under argon was added methyl 1-cyclopentene-1-carboxylate (485 µL, 3.96 mmol, 1 equiv) neat via syringe over 2 min. After 2 h at the same temperature, the reaction was treated with MeOH (7 mL) and diluted with 15% w/w NaOH aqueous solution (13 mL). The resulting slurry was

allowed to warm up to room temperature and after 30 min, the layers were separated. The aqueous phase was extracted twice with DCM, and the combined organic phases were washed with water, then brine, dried over $MgSO_4$, filtered and concentrated. The crude material thus obtained was used to make compound **1l** that was obtained (249 mg (29%) over two step) after purification by flash chromatography (50% EtOAc in DCM to 100% EtOAc) by following the representative procedure used to prepared compound **1a**. White crystalline solid; m.p.: 64-65 °C. ^{1}H NMR (500 MHz, CD_2Cl_2): δ 5.61 (m, 1H), 4.55 (br s, 2H), 3.99 (br s, 1H), 3.35 (s, 6H), 2.38 – 2.26 (m, 4H), 1.90 (quint, J = 7.5 Hz, 2H). ^{13}C{^{1}H} NMR (126 MHz, CD_2Cl_2): δ 167.0, 140.7, 126.9, 61.2, 55.3, 42.1 (2C), 32.9, 32.3, 23.3. IR (neat): $\tilde{\nu}$ = 1618 (s) (C=O), 1168 (s) (S=O) cm^{-1}. HRMS (ESI+): *m/z* calculated for $C_{10}H_{16}O_3S$ [M+H]$^+$: 217.0893, found: 217.0894.

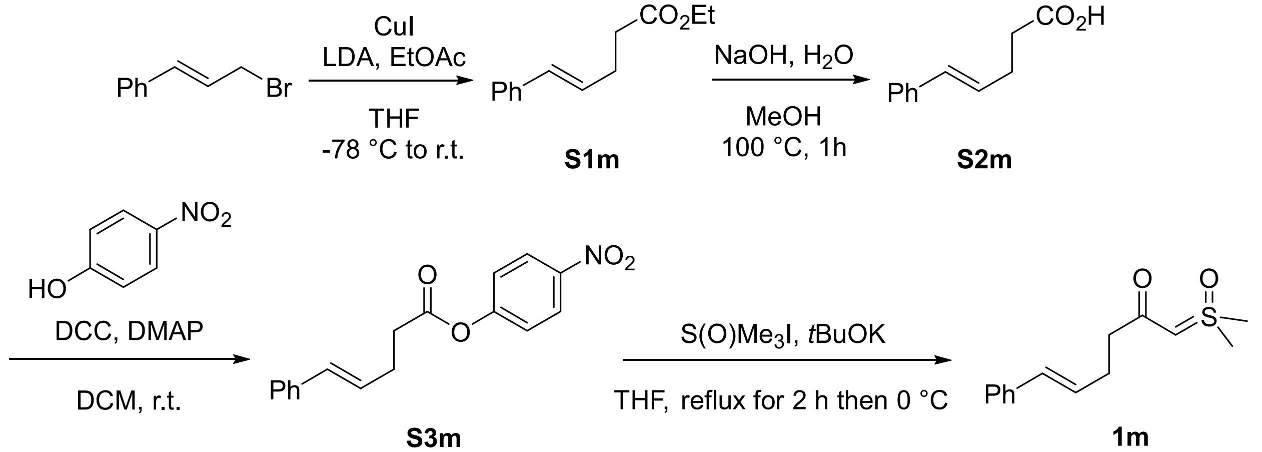

Ethyl (*E*)-5-phenylpent-4-enoate (S1m). Under Ar, *n*-BuLi (11.2 mL, 28.1 mmol, 2.8 equiv, 2.5 M solution in hexane) was added to a solution of *i*Pr_2NH (3.94 mL, 28.1 mmol, 2.8 equiv) in THF (20 mL) at -78 °C and the mixture was stirred for 20 min at this temperature before being added to a suspension of CuI (9.7 g, 51 mmol, 4 eq.) in distilled EtOAc (2.5 mL, 25.5 mmol, 2 eq.) and THF (60 mL) at -78 °C. After stirring for 30 min at -78 °C, a cooled solution of cinnamyl bromide (2.00 g, 10.15 mmol, 1 equiv) in THF (24 mL) at -78 °C was slowly added to the reaction flask at the same temperature. After stirring for 1 h at this temperature, the mixture was stirred at room temperature overnight. The reaction was quenched by addition of saturated aqueous solution $NaHCO_3$ and diluted with DCM. The aqueous layer was washed twice with DCM. The combined organic layers were washed with water, then brine, and then dried over $MgSO_4$, before filtration and solvent evaporation under vacuum. Purification of the crude product by column chromatography on silica gel (5 to 10% EtOAc in hexane) gave 1.38 g (67%) of ester **S1m** as colorless oil. ^{1}H NMR (500 MHz, $CDCl_3$): δ 7.36 – 7.31 (m, 2H), 7.31 – 7.27 (m, 2H), 7.23 – 7.18 (m, 1H), 6.43 (d, J = 15.8 Hz, 1H), 6.21 (dt, J = 15.8, 6.6 Hz, 1H), 4.15 (q, J = 7.1 Hz, 2H), 2.54 (ddt, J = 11.0, 7.7, 1.4 Hz, 2H), 2.51 – 2.45 (m, 2H), 1.26 (t, J = 7.1 Hz, 3H); in agreement with literature data.[8]

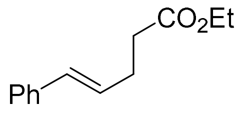

S1m

(*E*)-5-phenylpent-4-enoic acid (S2m). To a stirred solution of ester **S1m** (1.38 g, 6.67 mmol, 1 equiv) in 10% H_2O/MeOH (67 mL, 10 mL/mmol) was added NaOH (5.3g, 133 mmol, 20 equiv). The resulting solution was heated at 100 °C for 1h. The mixture was then cooled to rt, diluted with an aqueous 1 M NaOH solution, and poured into a separating funnel. The aqueous layer was extracted twice with DCM, and the organic layers were discarded. The aqueous layer was acidified with aqueous 4 M HCl solution and extracted five times with DCM. The combined organic layers were dried over $MgSO_4$, filtered and the solvent removed in vacuo to yield the carboxylic acid S# (1.11 g, 94%), which was used in the next step without further purification. ^{1}H NMR (500 MHz, $CDCl_3$): δ 7.34 (d, J = 7.3 Hz, 2H), 7.30 (t, J = 7.6 Hz, 2H), 7.25 – 7.18 (m, 1H), 6.45 (d, J = 15.8 Hz, 1H), 6.26 – 6.17 (m, 1H), 2.55 (d, J = 2.8 Hz, 4H); in agreement with literature data.[8]

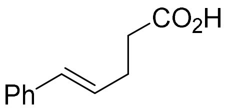

S2m

4-Nitrophenyl (*E*)-5-phenylpent-4-enoate (S3m). Under Ar, a flame-dried round bottom flask was charged with carboxylic acid # (300 mg, 1.70 mmol, 1 equiv), 4-nitrophenol (308 mg, 2.21 mmol, 1.3 equiv) and DMAP (21 mg, 0.17 mmol, 1.3 equiv). Dry DCM (6.8 mL, 0.25 M) was then added and the resulting mixture was stirred at room temperature. DCC (456 mg, 2.21 mmol, 1.3 equiv.) was added in one portion and the reaction mixture was allowed to stir at room temperature overnight. The mixture was filtered over Celite® (elution DCM) and concentrated under reduced pressure. The crude residue was purified by silica gel column chromatography (5 to 10% EtOAc in hexane) to afford ester **S3m** (406 mg, 80%). White solid, m.p.: 65-66 °C. ^{1}H NMR (500 MHz, $CDCl_3$): δ 8.26 (d, J = 9.1 Hz, 2H), 7.36 (d, J = 7.3 Hz, 2H), 7.32 (t, J = 7.6 Hz, 2H), 7.28 – 7.20 (m, 3H), 6.52 (d, J = 15.8 Hz, 1H), 6.27 (dt, J = 15.7, 6.8 Hz, 1H), 2.80 (t, J = 7.2 Hz, 2H), 2.68 (q, J = 7.1 Hz, 2H). $^{13}C\{^1H\}$ NMR (126 MHz, $CDCl_3$): δ 170.5, 155.4, 145.3, 137.0, 131.8, 128.6 (2C), 127.42, 127.38, 126.1 (2C), 125.2 (2C), 122.4 (2C), 34.1, 28.1. HRMS (ESI+): *m/z* calculated for $C_{17}H_{15}NO_4$ $[M+Na]^+$: 320.0893, found: 320.0890.

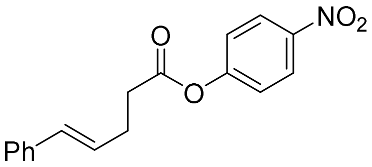

S3m

(*E*)-1-(dimethyl(oxo)-λ^6-sulfaneylidene)-6-phenylhex-5-en-2-one (1m). Under N_2, a flame-dried round bottom flask was placed under nitrogen and covered from light with aluminium foil. Then, trimethylsulfoxonium iodide (225 mg, 1.23 mmol, 3 equiv) was suspended in dry THF (1.0 mL) and *t*-BuOK (114 mg, 1.02 mmol, 3 equiv) was added. After 2 hours at reflux, the mixture was cooled to 0 °C, and a solution of carbonate S# (100 mg, 0.34 mmol, 1 equiv) in THF (0.4 mL) was added over 5 min. After overnight stirring room temperature, the mixture was filtered through a plug of Celite® (elution DCM) and all volatiles were removed under vacuum. Purification of the crude product was achieved by column chromatography on silica gel (2-3% MeOH in DCM) to give **1m** (62 mg (74%)) as a white solid. m.p.: 87-88 °C. ^{1}H NMR (500 MHz, CD_2Cl_2): δ 7.33 (d, J = 7.5 Hz, 2H), 7.28 (t, J = 7.6 Hz, 2H), 7.18 (t, J = 7.2 Hz, 1H), 6.41 (d, J = 15.8 Hz, 1H), 6.25 (dt, J = 15.7, 6.8 Hz, 1H), 4.47 (s, 1H), 3.35 (s, 6H), 2.46 (dd, J = 14.6, 7.2 Hz, 2H), 2.29 (t, J = 7.6 Hz, 2H). $^{13}C\{^1H\}$ NMR (126 MHz, CD_2Cl_2): δ 189.6, 137.8, 130.2, 129.8, 128.4 (2C), 126.8, 125.8 (2C), 69.4, 41.9 (2C), 40.4, 29.5.

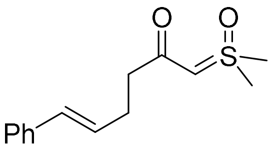

1m

IR (neat): $\tilde{\nu}$ = 1552 (s) (C=O), 1161 (s) (S=O) cm^{-1}. HRMS (ESI+): *m/z* calculated for $C_{14}H_{18}O_2S$ $[M+H]^+$: 251.1100, found: 251.1099.

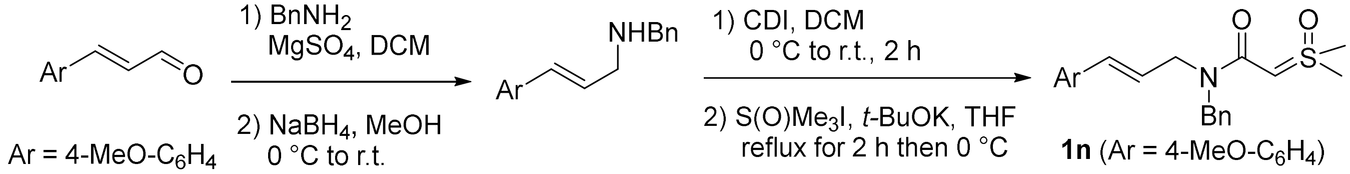

(*E*)-*N*-benzyl-2-(dimethyl(oxo)-λ⁶-sulfaneylidene)-*N*-(3-(4-methoxyphenyl)allyl) acetamide (1n). To a

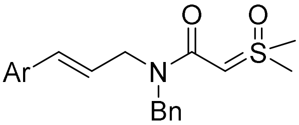

1n (Ar = 4-MeO-C6H4)

flame-dried flask under nitrogen was added $MgSO_4$ (2.0 g, 16.8 mmol, 2 equiv). The flask was evacuated and backfilled with nitrogen. Then, *trans*-p-methoxycinnamaldehyde (1.50 g, 9.25 mmol, 1.1 equiv) and dry DCM (14 mL) were added sequentially to the flask. Benzylamine (0.92 mL, 8.40 mmol, 1 equiv) was added dropwise to the suspension *via* syringe. The mixture was stirred at room temperature for 1 h, then filtered and concentrated under vacuum. The crude material was dissolved in anhydrous MeOH (10.5 mL, 0.8 M) and cooled to 0 °C in an ice-bath under nitrogen. $NaBH_4$ was added slowly in four portions. The reaction was allowed to warm up to r.t., and was stirred for 16 hours. The mixture was then quenched with sat. aq. solution of $NaHCO_3$, and the aqueous layer was extracted with DCM. The combined organic layers were dried over $MgSO_4$, concentrated under vacuum and the amine thus obtained was used without further purification in the same procedure used to prepare compound **1a**. Hence, from the amine (1.00 g, 3.95 mmol), **1n** was obtained by the following purification procedure. Column chromatography on silica gel (1-2% MeOH in DCM) afforded 408 mg of material that contained small impurities. Dissolving that material in hot DCM, before leaving that solution stand for 1 day at r.t. and then 2 days in the freezer (-20 °C) allowed the formation of crystals that were washed three times with Et_2O to give pure sulfoxonium ylide **1n** (215 mg, 18%) as white crystals. m.p. 127-128 °C. ^1H NMR (500 MHz, CD_2Cl_2): δ 7.37 – 7.19 (m, 7H), 6.84 (d, *J* = 8.7 Hz, 2H), 6.35 (d, *J* = 15.9 Hz, 1H), 6.01 (dt, *J* = 15.9, 6.0 Hz, 1H), 4.48 (br s, 2H), 3.98 (s, 1H), 3.93 (br s, 2H), 3.79 (s, 3H), 3.38 (s, 6H). $^{13}C\{^1H\}$ NMR (126 MHz, CD_2Cl_2): δ 168.0, 159.2, 139.1, 130.9, 129.6, 128.3 (2C), 127.4 (2C), 127.3 (2C), 126.7, 123.7, 113.8 (2C), 55.2, 55.1, {48.4}, {47.5}, 42.9 (2C); the resonances placed in bracket are not visible but inferred from HMBC and HSQC. IR (neat): $\tilde{\nu}$ = 1541 (s) (C=O), 1166 (s) (S=O) cm^{-1}. HRMS (ESI+): *m/z* calculated for $C_{21}H_{25}NO_3S$ $[M+H]^+$: 372.1628, found: 372.1624.

(*E*)-4-phenylbut-3-en-1-yl 2-(dimethyl(oxo)-λ⁶-sulfaneylidene)acetate (1o). This compound was obtained

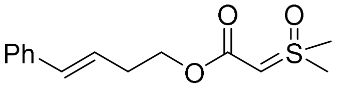

1o

(640 mg (18%)) after purification by flash chromatography (20% EtOAc in DCM) from **S2o** (2.00 g, 13.5 mmol) by following the representative procedure used to prepared compound **1a**. White solid; m.p.: 90-91 °C. ^1H NMR (500 MHz, $CDCl_3$): δ 7.38 – 7.32 (m, 2H), 7.29 (dd, *J* = 10.4, 4.9 Hz, 2H), 7.23 – 7.17 (m, 1H), 6.47 (d, *J* = 15.9 Hz, 1H), 6.22 (dt, *J* = 15.6, 6.9 Hz, 1H), 4.17 (br s, 2H), 3.95 (br s, 1H), 3.35 (s, 6H), 2.54 (br s, 2H). $^{13}C\{^1H\}$ NMR

(126 MHz, $CDCl_3$): δ 167.3, 137.4, 131.9, 128.5 (2C), 127.1, 126.5, 126.0 (2C), 62.1, 54.9, 42.3 (2C), 32.9. IR (neat): $\tilde{\nu}$ = 1650 (s) (C=O), 1159 (s) (S=O) cm^{-1}. HRMS (ESI+): *m/z* calculated for $C_{14}H_{18}O_3S$ $[M+H]^+$: 267.1055, found: 267.1054.

Iridium-catalyzed cyclopropanation of α-carbonyl sulfoxonium ylides into 2a–2o

1. Optimization of the cyclopropanation of sulfoxonium ylide 1a into racemic (±)-2a

Table S1. Optimization of the iridium-catalyzed cyclopropanation of α-carbonyl sulfoxonium ylides.[a]

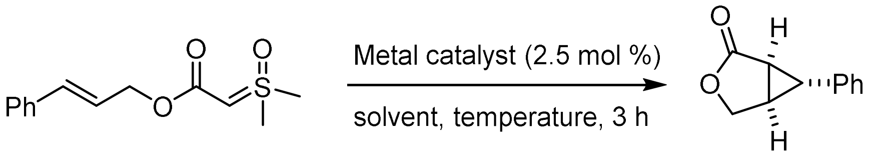

1a **(±)-2a**

entry	catalyst	solvent	T[b]	yield[c]
1	$[Ir(cod)Cl]_2$	1,2-DCE	80 °C	94%[d]
2	$[Ir(cod)Cl]_2$	THF	60 °C	5%
3	$[Ir(cod)Cl]_2$	acetone	60 °C	71%
4	$[Ir(cod)Cl]_2$	toluene/DMF[e]	80 °C	65%
5	$[Ir(cod)Cl]_2$	1,2-DCE[f]	80 °C	50%
6	$[Ir(cod)Cl]_2$	1,2-DCE[g]	80 °C	37%
7	$[Ir(cod)Cl]_2$	1,2-DCE	60 °C	77%[d]
8	$[Ir(cod)Cl]_2$	1,2-DCE	40 °C	70%
9	$[Cp^*IrCl_2]_2$	1,2-DCE	80 °C	65%
10	$[Cp^*RhCl_2]_2$	1,2-DCE	80 °C	0%
11	$Rh_2(OAc)_4$	1,2-DCE	80 °C	2%
12	$[Rh(cod)Cl]_2$	1,2-DCE	80 °C	5%

[a] Slow addition of **1a** (0.2 mmol) in solvent (3 mL) to the metal catalyst in solvent (9 mL) under N_2 over 3 hours. [b] Temperature of heating block. [c] Yield determined by ^1H NMR of the crude with 1,3,5-trimethoxybenzene as internal standard. [d] Yield of isolated product. [e] 0.1 mL of DMF was added to solubilize the starting material. [f] Solvent was not degassed. [g] Under air. cod: cyclooctadiene; Cp*: 1,2,3,4,5-pentamethylcyclopentadienyl; 1,2-DCE: 1,2-dichloroethane; THF: tetrahydrofuran; DMF: dimethylformamide.

2. Representative procedure with racemic catalyst $[Ir(cod)Cl]_2$

Under Ar, an oven-dried Radley vial was charged with $[Ir(cod)Cl]_2$ (3.4 mg, 0.005 mmol, 2.5 mol%) and 1,2-DCE (9 mL) was added (the solvent had been freshly degassed by argon bubbling for 15 min). The vial was placed in a Radleys Carousel 12 Chemistry Reaction System and was heated to reflux (temperature of metal block set at 80 °C). Sulfoxonium ylide **1a** (50 mg, 0.2 mmol, 1 equiv) was then dissolved in degassed 1,2-DCE (3 mL) and this solution was slowly added to the catalyst solution over 3 h with a syringe pump (1 mL/h). Upon completion of the addition, the reaction mixture was allowed to cool down to room temperature and the solvent was evaporated under vacuum. The crude residue was purified by column chromatography (20% EtOAc in hexane) to give 32.9 mg (94%) of lactone **(±)-2a** as a white solid.

3. Synthesis of ligands 5–8

(*R*,*R*)-**3** was purchased from Strem. (*R*,*R*)-**4** was donated by Dr Andrew Carnell from The University of Liverpool, United Kingdom. Other ligands were prepared as described below.

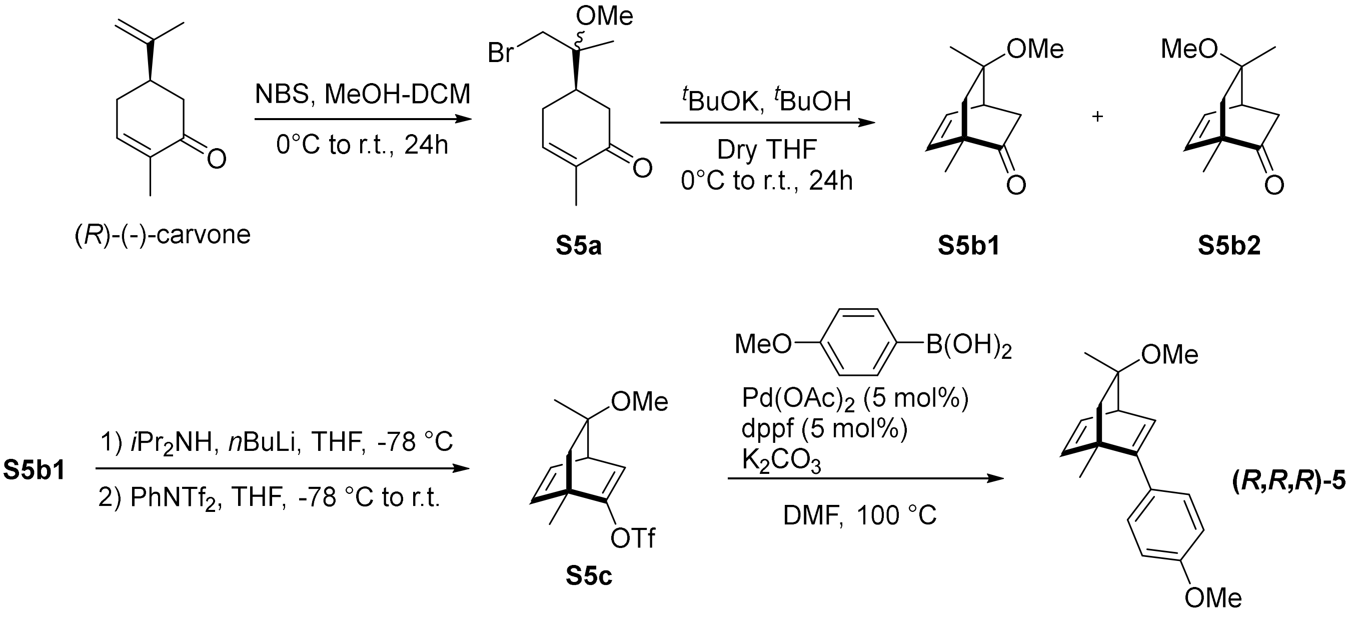

(*R*)-5-(1-bromo-2-methoxypropan-2-yl)-2-methylcyclohex-2-en-1-one (S5a). To a solution of (*R*)-(-)-carvone (10.0 g, 66.5 mmol, 1 equiv) cooled to 0 °C in a 2:3 mixture of MeOH:DCM (100 mL) was added N-bromosuccinimide (14.2 g, 79.8 mmol, 1.2 equiv) in portions over a period of 1 hour. The reaction mixture was stirred 24 h at room temperature, diluted with DCM, washed successively with 1 M NaOH and brine, dried ($MgSO_4$) and concentrated. Purification by flash chromatography (10% EtOAc in hexane) gave 309 mg (59%) of a 1:1.9 epimeric mixture of **S5a**. ^{1}H NMR (500 MHz, $CDCl_3$): δ 6.80 – 6.68 (m, 1H), 3.50 – 3.39 (m, 2H), 3.24 (s, 1H), 3.23 (s, 1.9H), 2.60 (ddd, J = 15.8, 3.6, 1.6 Hz, 0.7H), 2.57 – 2.48 (m, 1H), 2.48 – 2.37 (m, 1H), 2.34 – 2.15 (m, 2.3H), 1.80 – 1.74 (m, 3H), 1.26 (s, 3H); the data was consistent with that reported in the literature.[9]

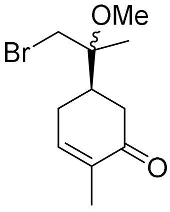

S5a

(1*R*,4*S*,8*R*)-8-methoxy-1,8-dimethylbicyclo[2.2.2]oct-5-en-2-one (S5b1). A solution containing of **S5a** (10.9 g, 41.9 mmol, 1 equiv) in ^{t}BuOH (105 mL) and THF (105 mL) was chilled to 0 °C, and ^{t}BuOK (5.49 g, 49 mmol, 1.17 equiv) was added in portions. Upon completion of the addition of the base, the reaction mixture was stirred at 0 °C for 10 min and then warmed to room temperature and stirred for 60 h. The reaction mixture was diluted in Et_2O, washed successively with 5% HCl aqueous solution and brine, then dried over $MgSO_4$ and concentrated under vacuum. Purification by flash chromatography (5% to 8% EtOAc in hexane) gave 2.51 g (33%) of **S5b1** and 2.69 g (36%) of **S5b2** as light-yellow oils. **S5b1**, ^{1}H NMR (500 MHz, $CDCl_3$): δ 6.48 – 6.41 (m, 1H), 5.86 (d, J = 8.0 Hz, 1H), 3.20 (s, 3H), 2.97 – 2.88 (m, 1H), 2.56 (dd, J = 18.1, 2.1 Hz, 1H), 1.89 (dd, J = 18.2, 3.1 Hz, 1H), 1.78 (d, J = 13.6 Hz, 1H), 1.47 (d, J = 13.6 Hz, 1H), 1.28 (s, 3H), 1.17 (s, 3H). **S5b2,** ^{1}H NMR (500 MHz, $CDCl_3$): δ 6.47 (dd, J = 8.0, 6.3 Hz, 1H), 5.94 (dd, J = 8.0, 0.7 Hz, 1H), 3.18 (s, 3H), 3.02 – 2.99 (m,

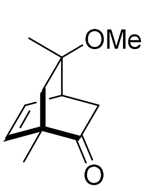

S5b1

1H), 2.14 (dd, J = 18.9, 2.3 Hz, 1H), 2.08 (dd, J = 18.8, 3.3 Hz, 1H), 1.75 (d, J = 14.0 Hz, 1H), 1.55 (d, J = 13.1 Hz, 2H), 1.37 (s, 3H), 1.19 (s, 3H). The data was consistent with the literature.[10]

(1*R*,4*R*,8*R*)-8-methoxy-1,8-dimethylbicyclo[2.2.2]octa-2,5-dien-2-yl trifluoromethanesulfonate (S5c).

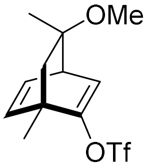

S5c

Under argon, freshly distilled *i*Pr_2NH (1.02 mL, 7.22 mmol, 1.3 equiv) was placed in a 250 mL round bottom flask and THF (33 mL) was added. The solution was cooled at –78 °C and *n*BuLi (2.89 mL, 2.5 M in hexanes, 7.22 mmol, 1.3 equiv) was added dropwise. The light-yellow solution was stirred for 20 min at –78 °C and a solution of ketone **S5b1** (740 mg, 5.55 mmol, 1 eq.) in THF (4 mL) was added dropwise. The yellow reaction mixture was stirred at –78 °C for 1 h. A solution of the $PhNTF_2$ (2.97 g, 8.33 mmol, 1.5 eq.) in THF (5 mL) was then added dropwise over 5 min and the reaction mixture was allowed to warm up to room temperature and stirred overnight. The orange solution was quenched with water and THF was removed in vacuo. DCM (20 mL) was added and the aqueous layer extracted by two times with DCM (2x 15 mL). The combined organic layers were washed with brine (30 mL), dried over $MgSO_4$, filtered and concentrated under vacuum. The crude residue was purified by column chromatography (2 to 4% EtOAc in hexane) to afford 533 mg (49%) of vinyl triflate **S5c** as a light-yellow oil. ^{1}H NMR (500 MHz, $CDCl_3$): δ 6.31 (t, J = 6.6 Hz, 1H), 6.13 (d, J = 6.8 Hz, 1H), 6.10 (dd, J = 7.1, 1.5 Hz, 1H), 3.60 (td, J = 6.7, 1.6 Hz, 1H), 3.16 (s, 3H), 1.78 (d, J = 12.0 Hz, 1H), 1.46 (s, 3H), 1.29 (d, J = 12.1 Hz, 1H), 1.27 (s, 3H); the data was consistent with that reported in the literature.[11]

(1*R*,4*R*,8*R*)-8-methoxy-2-(4-methoxyphenyl)-1,8-dimethylbicyclo[2.2.2]octa-2,5-diene ((*R*,*R*,*R*)-5).

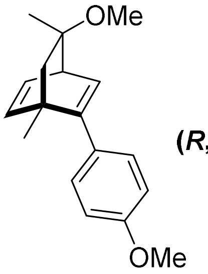

(*R*,*R*,*R*)-5

Under N_2, a Schlenk tube was charged with $Pd(OAc)_2$ (3.6 mg, 0.016 mmol, 5 mol%) and dppf (8.9 mg, 0.016 mmol, 5 mol%). Degassed DMF (0.32 mL) was added and the mixture was stirred 10 min at rt. Another Schlenk tube equipped was charged with K_2CO_3 (88 mg, 0.64 mmol, 2 equiv), vinyl triflate **S5c** (100 mg, 0.32 mmol, 1 equiv) and 4-methoxyphenylboronic acid (98 mg, 0.64 mmol, 2 equiv) under N_2. Degassed DMF (0.32 mL) and the previously prepared catalyst solution were added and the mixture was stirred at 100 °C for 1 h (preheated oil bath). The mixture was cooled to room temperature, diluted with toluene (10 mL) and concentrated under vacuum. The crude residue was purified by silica gel column chromatography (2 % EtOAc in hexane) to afford 10.5 mg (12%) of ligand as a colorless oil. ^{1}H NMR (500 MHz, $CDCl_3$): δ 7.10 – 7.04 (m, 2H), 6.86 – 6.80 (m, 2H), 6.36 (t, J = 6.8 Hz, 1H), 6.15 (dd, J = 7.2, 1.2 Hz, 1H), 6.13 (d, J = 5.9 Hz, 1H), 3.80 (s, 3H), 3.60 (td, J = 6.1, 1.3 Hz, 1H), 3.21 (s, 3H), 1.59 (d, J = 11.9 Hz, 1H), 1.31 (s, 3H), 1.29 (s, 3H), 1.28 (d, J = 10.4 Hz, 1H); the data was consistent with that reported in the literature.[11]

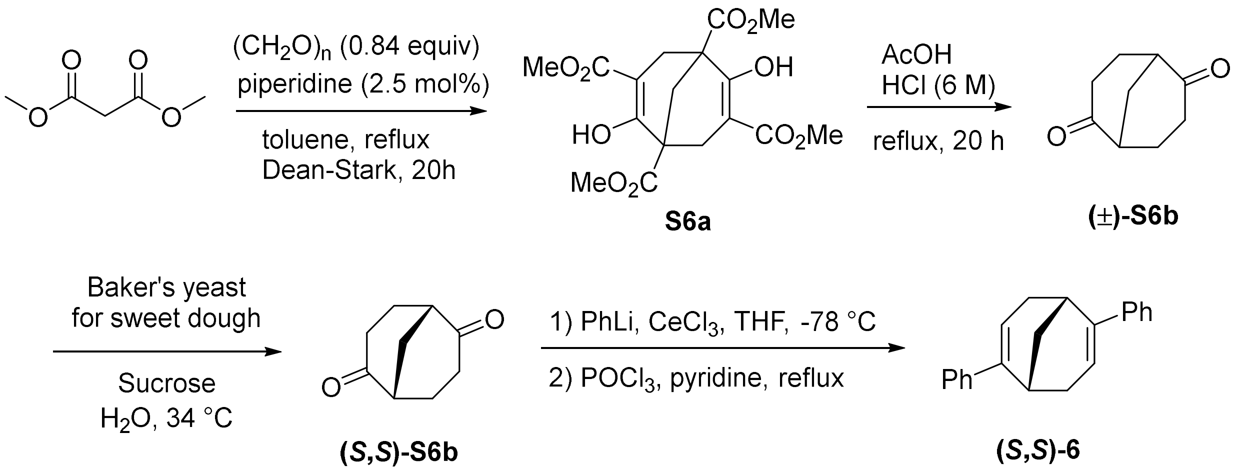

Tetramethyl 2,6-dihydroxybicyclo[3.3.1]nona-2,6-diene-1,3,5,7-tetracarboxylate (S6a). In a 2L flask

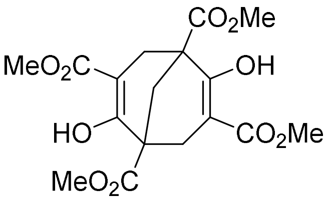

S6a

quipped with a magnetic stirrer bar was charged dimethyl malonate (250 g, 1.89 mol, 1.00 equiv), paraformaldehyde (47.45 g, 1.58 mol, 0.84 equiv) and toluene (375 mL, 5 M). To this solution was added piperidine (4.67 mL, 47.3 mmol, 2.5 mol%) while stirring. The flask was equipped with a condenser and a Dean-Stark trap. The reaction mixture was stirred and heated at reflux, first at 100 °C for 3-4 hours before heating overnight at 120 °C. Water accumulated in the trap reached 90% of its theoretical volume. Toluene was removed under reduce pressure at 60 °C using a rotary evaporator, affording a yellowish and very viscous oil. A NaOMe solution was freshly made using 30.5 g of sodium and dry MeOH (355 mL). The oil was quickly transferred to the 2L flask containing the NaOMe solution and additional MeOH (90 mL) were used to rinse the flask. The mixture was stirred and heated to reflux overnight. A yellowish fine powder precipitated. The suspension was cooled on an ice bath for 1 h and Et_2O (150 mL) was added to complete the precipitation. After additional 30 minutes cooling in an ice bath, the solid was collected by filtration using a Büchner funnel. The filter cake was washed with a 1:1 cold mixture of MeOH/Et_2O (120 mL) yielding a white solid. The solid was dissolved in distilled water (600 mL) and 6 M HCl (approx. 130 mL) was added. Upon acidifying, product precipitated as a white solid, which was collected by filtration. The solid was then dried by crushing into powder on filter paper to furnish 112.3 g (62%) of the compound **S6a**. ^{1}H NMR (500 MHz, $CDCl_3$): δ 12.16 (s, 2H), 3.78 (s, 6H), 3.76 (s, 6H), 2.87 (s, 4H), 2.32 (s, 2H); the data was consistent with that reported in the literature.[12]

Bicyclo[3.3.1]nonane-2,6-dione ((±)-S6b) and resolution to ((1*S*,5*S*)-S6b). A solution of **S6a** (50.0 g, 130

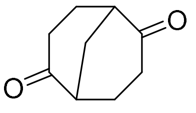

(±)-S6b

mmol, 1 equiv) in glacial AcOH (130 mL, 1 M) was heated under reflux and HCl (6 M, 88 mL) was added dropwise. The mixture was heated overnight (20 h). The solvent was removed under vacuum and the crude residue was dissolved in DCM, washed twice with sat. $NaHCO_3$, brine and dried over $MgSO_4$. Upon concentration, a white solid was obtained. The crude product was washed with ice cold Et_2O to afford 11.52 g (58%) of the **(±)-S6b** as a white solid; ^{1}H NMR (500 MHz, $CDCl_3$): δ 2.77 –2.69 (m, 2H), 2.58 (ddd, J = 17.2, 6.9, 4.0 Hz, 2H), 2.39 (dt, J = 17.2, 9.2 Hz, 2H),

2.28 – 2.17 (m, 2H), 2.15 – 1.97 (m, 4H); the data was consistent with that reported in the literature.[12] **Chiral resolution** - According to a reported procedure,[12] a flask was charged with a solution of **(±)-S6b** (2.00 g, 131 mmol, 1 equiv) and water (40 mL, 0.33 M). Stirring and gentle heating (~30 °C) was necessary to get the substrate dissolved in solution. Under gentle stirring at r.t. Baker's yeast for sweet dough (3.4 g, 170% w/w) was added in small portions, followed by Sucrose (6.66 g, 330% w/w), and the gentle stirring was continued for six days with further addition of sucrose (6.66 g) the second and the third day. The reaction mixture was centrifuged at 5000 rpm for 10 min. The slightly yellow supernatant was decanted and saturated with excess NaCl. The precipitate was extracted with EtOAc (3 × 10 mL). The slightly yellow supernatant was then extracted with $CHCl_3$ (3 × 15 mL). The combined organic phases were concentrated in vacuo yielding a yellowish semi-solid that was suspended in water (4 mL) and subjected to a second fermentation in accordance with the above procedure. After the second workup, enantiomerically pure diketone **(*S*,*S*)-S6b** (270 mg, 27%, >99% ee) was isolated by flash chromatography (30% EtOAc in hexane) as a white solid. Enantiomeric excess was determined by chiral HPLC with Chiralcel IC column, Hexane/IPA 80/20; 0.8 mL/min; 25 °C, t_l = 19.0 min (major), ee > 99%.

(1S,5S)-2,6-diphenylbicyclo[3.3.1]nona-2,6-diene ((*S*,*S*)-6). Anhydrous $CeCl_3$ (843 mg, 2.6 mmol) was suspended in THF (13.1 mL). To this suspension at –78 °C, phenyllithium (1.8 mL, 3.4 mmol, 1.9 M in Bu_2O, 2.6 eq.) was added. After stirring was continued for 1 h, racemic bicyclo[3.3.1]nonane-2,6-dione (200 mg, 1.31 mmol, 1 eq.) in THF (2.6 mL) was added, and the reaction mixture was stirred at –78 °C for 6 h. Water was added to quench the reaction and the organic solvent was removed in vacuo. The aqueous layer was extracted with EtOAc three times. The combined organic layers were dried over $MgSO_4$. Removal of the solvent gave the crude diol. To the crude diol was added pyridine (1.3 mL) and $POCl_3$ (1.20 g, 7.86 mmol, 6 eq.) at room temperature, and the mixture was heated to reflux for 12 h. After cooled to room temperature, the reaction mixture was quenched with water, and extracted with Et_2O. The combined organic layers were washed with 2N NaOH and water, and then dried over $MgSO_4$. Removal of the solvent gave the crude product, which was purified by silica gel column chromatography (2 % DCM in hexane) to afford 134 mg (38% yield for 2 steps) of (*R*,*R*)-**6**. ^{1}H NMR (500 MHz, $CDCl_3$): δ 7.44 –7.39 (m, 4H), 7.35 – 7.30 (m, 4H), 7.26 – 7.21 (m, 2H), 5.98 (dd, J = 5.1, 2.3 Hz, 2H), 3.15 – 3.09 (m, 2H), 2.47 (dd, J = 18.1, 5.3 Hz, 2H), 2.08 (dd, J = 18.4, 5.1 Hz, 2H), 2.00 (t, J = 3.0 Hz, 2H); the data was consistent with that reported in the literature.[13]

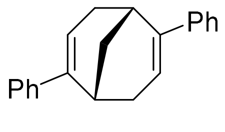

(*S*,*S*)-6

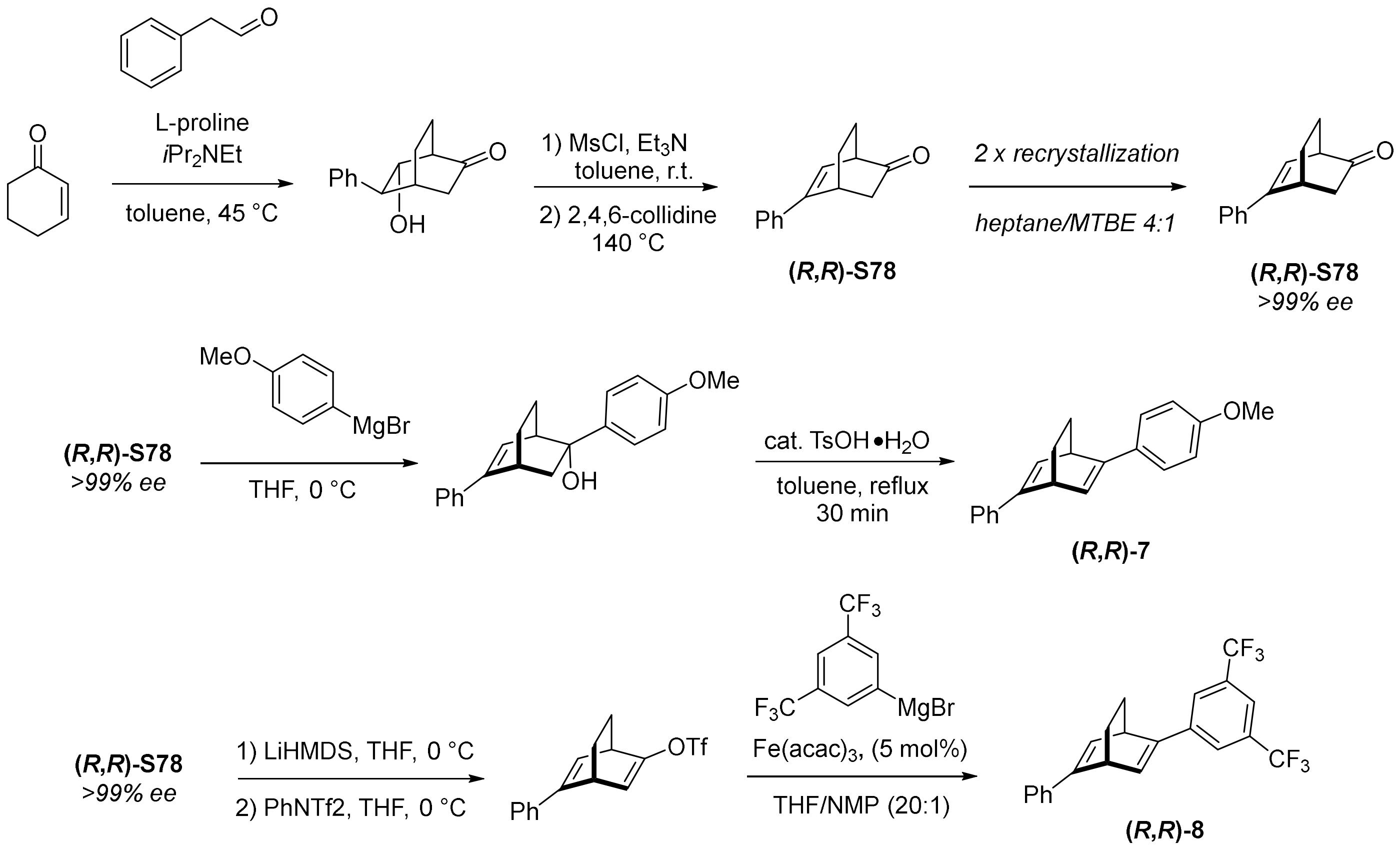

(1*R*,4*R*)-5-phenylbicyclo[2.2.2]oct-5-en-2-one ((*R*,*R*)-S78). Following a reported procedure, L-proline (3.00 g, 26 mmol, 0.25 equiv) and *i*Pr_2NEt (4.53 mL, 26 mmol, 0.25 equiv) were added to a mixture of cyclohexenone (10.7 mL, 104 mmol, 1 equiv) and phenylacetaldehyde (13.7 g, 114 mmol, 1.1 equiv.) in toluene (70 mL, analytical reagent grade) at room temperature. The mixture was stirred at 45 °C for 4 days. The suspension was then cooled to 20 °C, stirred at 20 °C for 20 min and filtered. The filter cake was washed with water (3 × 30 mL) and toluene (3 × 30 mL). The resulting white solid was dried by sucking air through the filter for 10 min and then crushed on absorbing paper to complete the drying. The cyclized product (10.0 g, 44%) was suspended in dry toluene (18 mL) at 10-20 °C in an oven-dried round-bottom flask under argon, freshly distilled Et_3N (9.67 mL, 69.4 mmol, 1.5 equiv) was added before MsCl (4.65 mL, 60.1 mmol, 1.3 equiv). After stirring at this temperature for 30 min, the mixture was washed with water (2 × 10 mL) and concentrated to dryness under reduced pressure to afford crude mesylate intermediate as yellow oil that solidified at r.t. The crude residue (13.11 g) was then dissolved in 2,4,6-collidine (8.3 mL) and stirred at 140 °C for 1.5 h. HCl (2M, 10 mL) and hexane (20 mL) were added, and the layers were separated. The organic layer was washed with 2M HCl (2 x 10 mL) and water (20 mL), dried over $MgSO_4$ and filtered. The filtrate was evaporated to dryness at 50 °C under reduced pressure to afford **(*R*,*R*)-S78** (4.9 g, 53%, 70:30 *e.r.*) as a light-red oil. A sample (1.0 g) of that material was purified by column chromatography (20% EtOAc in hexane), but the purified material remains an orange oil. After 4 days in the freezer at -20 °C, the oil solidified to a white solid. Crystals were obtained by adding a mixture of heptane/MTBE (4:1, approx. 3 vol), heating the mixture to 70 °C in a flask equipped with a condenser. Upon

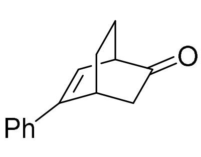

(*R*,*R*)-S78

full dissolution of the compound, the heating and stirring were stopped, the mixture was left cooling down to room temperature slowly by letting the flask on the heating block overnight. Long and colorless crystals were obtained (332 mg, 33%), enantiopure by chiral HPLC measurement. This crystallization procedure was repeated on a non-purified batch (5.19 g) with a seeding a 40 °C whilst the mixture was cooling down. This step afforded crystals with ~80% e.e., which were recrystallized again to give 1.59 g (31%) of enantiopure compound as colorless crystals. Enantiomeric excess was determined by chiral HPLC with Chiralcel AD-H column, Hexane/IPA 80/20; 1mL/min; 25 °C, t_1 = 6.7 min (major), ee > 99%. ^{1}H NMR (500 MHz, $CDCl_3$): δ 7.47 –7.42 (m, 2H), 7.39 –7.34 (m, 2H), 7.32 –7.27 (m, 1H), 6.44 (dd, J = 6.7, 2.2 Hz, 1H), 3.53 (hex, J = 2.7 Hz, 1H), 3.32 – 3.28 (m, 1H), 2.20 – 2.16 (m, 2H), 2.02 – 1.95 (m, 1H), 1.90 – 1.83 (m, 1H), 1.76 – 1.62 (m, 2H); the data was consistent with that reported in the literature.[14]

(1*R*,4*R*)-2-(4-methoxyphenyl)-5-phenylbicyclo[2.2.2]octa-2,5-diene ((*R*,*R*)-7). Mg turnings (88 mg, 3.6 mmol, 1.2 equiv) were added to a Schlenk tube which was then heated (heat gun) under vacuum for 2 min with vigorous stirring. To the cooled flask was added anhydrous THF (1.2 mL) and I_2 (1 crystal). 4-methoxybromobenzene (561 mg, 3 mmol, 1 equiv) was then added neat dropwise on the suspension of Mg in THF.

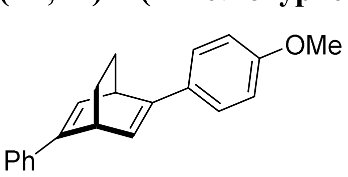

(*R*,*R*)-7

The mixture was then heated to reflux for 2 hours. Upon cooling down to room temperature, concentration was determined to be 1.1 M by titration with I_2. Under argon, this Grignard solution (1.51 mL, 1.1 M, 2 equiv) was added dropwise to a solution of enantiopure ketone (*R*,*R*)-**S78** (150 mg, 0.756 mmol, 1 equiv) in THF (1.5 mL) cooled in an ice-bath. The reaction was allowed to warm to room temperature and was stirred overnight. The mixture was cooled to 0 °C and quenched with water, EtOAc was added and the layers were separated. The aqueous layer was extracted twice with EtOAc and the combined organic layers were washed with water and brine, dried over $MgSO_4$, filtered and concentrated under vaccum. The crude residue was dissolved in toluene (15 mL), before *p*-TsOH•H_2O (15 mg, 0.08 mmol, 0.1 equiv) was added. The resulting mixture was stirred at reflux for 30 min. Solvent was then removed under *vacuo* and the residue was purified by silica gel column chromatography (5% EtOAc in hexane) to yield 99 mg (46%) of ligand (*R*,*R*)-**7** as a white solid. ^{1}H NMR (500 MHz, $CDCl_3$): δ 7.45 (d, J = 7.7 Hz, 2H), 7.39 (d, J = 8.6 Hz, 2H), 7.33 (t, J = 7.6 Hz, 2H), 7.22 (t, J = 7.3 Hz, 1H), 6.88 (d, J = 8.6 Hz, 2H), 6.63 (d, J = 5.2 Hz, 1H), 6.53 (d, J = 5.2 Hz, 1H), 4.20 (t, J = 6.7 Hz, 2H), 3.82 (s, 3H), 1.54 (s, 4H); the data was consistent with that reported in the literature.[14]

(1*R*,4*R*)-2-(3,5-bis(trifluoromethyl)phenyl)-5-phenylbicyclo[2.2.2]octa-2,5-diene ((*R*,*R*)-8). Mg turnings (172 mg, 7.2 mmol, 1.2 equiv) were added to a Schlenk tube which was then heated (heat gun) under vacuum for 2 min with vigorous stirring. To the cooled flask was added anhydrous THF (5 mL) and I_2 (1 crystal). Then, 3,5-di(trifluoromethyl)bromobenzene (1.76 g, 6 mmol, 1 equiv) was added neat dropwise on the suspension of Mg in THF at room temperature. The reaction is

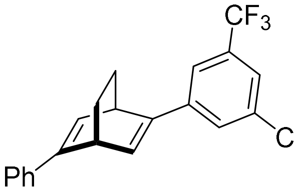

(*R*,*R*)-8

initiated after few drops added (solution became colorless then light grey), and the tube was cooled with a 20

°C water bath and the slow addition was continued. After stirring for 20 min, the concentration was determined to be 0.75 M by titration with I_2. Separately, and following a reported procedure,[15] an oven-dried tube was charged with LiHMDS (2.0 mL, 2.0 mmol, 1 M in THF, 2 equiv) and anhydrous THF (14 mL) and the resulting solution was cooled to 0 °C. A solution of enantiopure ketone (*R*,*R*)-**S78** (200 mg, 1.0 mmol, 1 equiv) in THF (3.0 mL) was then added dropwise and the mixture was allowed to stir for 1 h at 0 °C. A solution of $PhNTf_2$ (714 mg, 2 mmol, 2 equiv) was slowly added to the mixture and the reaction was stirred for 2 hours at 0 °C. The reaction mixture was quenched with chilled water, extracted twice with ether, then washed with brine, dried over $MgSO_4$ and concentrated under vacuum. The crude triflate was dissolved in THF (1 mL) and added to $Fe(acac)_3$ (18 mg, 0.05 mmol, 5 mol%) in THF (20 mL) and NMP (1 mL) an oven-dried flask under argon. The reaction flask was cooled to -10 °C in a salted ice-bath before a solution of the Grignard reagent (3 mL, 0.75 M, 2 mmol, 2 equiv) prepared previously was slowly added. The mixture was allowed to warm to r.t. and was stirred for 4 h. Then, chilled water was added, the aqueous layer was extracted twice with Et_2O, washed with brine, dried over $MgSO_4$ and concentrated. The residue was purified by silica gel column chromatography (100% hexane) to yield 164 mg (42%) of ligand (*R*,*R*)**-8** as a white solid. ^1H NMR (500 MHz, $CDCl_3$): δ 7.83 (s, 2H), 7.71 (s, 1H), 7.44 (d, J = 7.4 Hz, 2H), 7.35 (t, J = 7.7 Hz, 2H), 7.26 – 7.22 (m, 1H), 6.84 (dd, J = 6.5, 2.0 Hz, 1H), 6.65 (dd, J = 6.4, 1.9 Hz, 1H), 4.30 (dd, J = 6.3, 1.8 Hz, 1H), 4.23 (dd, J = 6.3, 2.0 Hz, 1H), 1.65 – 1.55 (m, 4H); the data was consistent with that reported in the literature.[16]

4. Representative procedure with chiral catalyst $[Ir(coe)_2Cl]_2$/(*R*,*R*)-3

An oven-dried Radley vial was taken into a glovebox and was charged with $[Ir(coe)_2Cl]_2$ (4.5 mg, 0.05 mmol, 2.5 mol%) and (*R*,*R*)-**3** (2.6 mg, 0.01 mmol, 5 mol%). The vial was fitted with a septum and taken out from the glovebox. Then, 1,2-DCE (9 mL) was added (the solvent had been freshly degassed by argon bubbling for 15 min) and the vial was placed in a Radleys Carousel 12 Chemistry Reaction System and was heated to reflux (temperature of metal block set at 80 °C) for 20 min. The reaction mixture changed from light-yellow to light-red during this period. The heating was stopped and the vial was left cooling down to room temperature before a solution of sulfoxonium ylide **1a** (50 mg, 0.2 mmol, 1 equiv) in degassed 1,2-DCE (3 mL) was slowly added to the reaction mixture over 3 h (syringe pump 1 mL/h). Upon completion of the addition, the reaction mixture was allowed to stir for 14 h at room temperature. The solvent was evaporated under vacuum. The crude residue was purified by column chromatography (20% EtOAc in Hexane) to yield 30.9 mg (89%) of lactone **(–)-2a** as a white solid.

5. Representative procedure with chiral catalyst $[((R,R)\text{-}3)IrCl]_2$

Synthesis of $[((R,R)\text{-}3)IrCl]_2$

A J-Young Schlenk tube was charged with $[Ir(coe)_2Cl]_2$ (90 mg, 0.1 mmol, 1.0 equiv) in an argon-filled glove box. Outside the glove box, degassed hexane (1.0 mL) and (*R*,*R*)-**3** (52 mg, 0.2 mmol, 2.0 equiv). The tube was sealed and placed in an oil bath (50 °C) and stirred for 48 hours. The reaction was cooled to room

temperature. The red precipitate obtained was isolated by filtration and washed with a small amount of hexane to yield 68 mg (70%) of [((*R,R*)-**3**)IrCl]$_2$ as a bright red solid. ^{1}H NMR (500 MHz, $CDCl_3$): δ 7.49 – 7.42 (m, 8H), 7.33 – 7.24 (m, 12H),* 4.71 (d, J = 5.9 Hz, 4H), 3.28 (dd, J = 6.3, 1.1 Hz, 4H), 1.13 – 1.03 (m, 8H). The signal at 7.33 – 7.24 cannot be integrated precisely due to overlap with residual $CHCl_3$ of the deuterated solvent. $^{13}C\{^1H\}$ NMR (126 MHz, $CDCl_3$): δ 139.7 (4C), 128.3 (8C), 126.7 (4C), 126.6 (8C), 49.2 (4C), 44.6 (4C), 30.4 (4C), 28.5 (4C). HRMS (ESI-): *m/z* calculated for $C_{20}H_{18}ClIr$ $[M+Cl]^-$: 521.0420, found: 521.0396 (accurate mass could only be obtained on the monomeric diene-iridium chloride complex).

Asymmetric cyclopropanation on 1 mmol scale

An oven-dried 100 mL two-necked flask was charged under argon with pre-made iridium diene catalyst [((*R,R*)-**3**)IrCl]$_2$ (24 mg, 0.025 mmol, 2.5 mol%) and 1,2-DCE (45 mL) (the solvent had been freshly degassed by argon bubbling for 15 min). A solution of sulfoxonium ylide **1a** (252 mg, 1.0 mmol, 1 equiv) in degassed 1,2-DCE (15 mL) was slowly added to the reaction mixture over 15 h (syringe pump 1 mL/h). Upon completion of the addition, the reaction mixture was allowed to stir for 27 h at room temperature. The solvent was evaporated under vacuum. The crude residue was purified by column chromatography (20% EtOAc in Hexane) to yield 158 mg (91%) of lactone **(–)-2a** as a white solid.

(1*S*,5*R*,6*R*)-6-Phenyl-3-oxabicyclo[3.1.0]hexan-2-one ((–)-**2a**). Racemic product: 32.9 mg (94%). White solid; m.p.: 85-86 °C. ^{1}H NMR (500 MHz, $CDCl_3$): δ 7.35 – 7.28 (m, 2H), 7.28 – 7.22 (m, 1H), 7.10 – 7.04 (m, 2H), 4.47 (dd, J = 9.5, 4.7 Hz, 1H), 4.42 (d, J = 9.5 Hz, 1H), 2.53 (dtd, J = 5.6, 4.7, 0.7 Hz, 1H), 2.38 – 2.30 (m, 1H). $^{13}C\{^1H\}$ NMR (126 MHz, $CDCl_3$): δ 175.0, 137.2, 128.8 (2C), 127.2, 126.0 (2C), 69.8, 29.4, 27.5, 26.2. HRMS (ESI+): *m/z* calculated for $C_{11}H_{10}O_2$ $[M+H]^+$: 175.0754, found: 175.0753. Enantioenriched product: 30.9 mg (89%) (on 0.2 mmol scale) and 158 mg (91%) (on 1.0 mmol scale). Enantiomeric excess (e.e.) was determined by chiral HPLC with Chiralcel OD column, Hexane/IPA 80/20; 1 mL/min; 25 °C: t_1 = 13.6 min (minor), t_2 = 14.6 min (major); e.e. = 90% (on 0.2 mmol scale) and e.e. = 93% (on 1.0 mmol scale). Recrystallisation from EtOAc/hexane (1:1) in 65% yield gave a sample with 99% e.e. $[\alpha]_D^{23}$= -131.9° (*c* 0.26, $CHCl_3$). The absolute configuration of the major enantiomer was established by comparison with literature data for the (1*R*,5*S*,6*S*) enantiomer: $[\alpha]_D^{23}$= +130° (*c* 0.26, $CHCl_3$).[17]

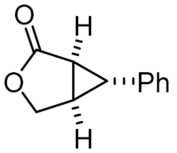

(–)-2a

(1*S*,5*R*,6*R*)-6-(4-fluorophenyl)-3-oxabicyclo[3.1.0]hexan-2-one ((–)-2b). Racemic product: 32.7 mg (85%) after purification by flash chromatography (20 to 30% EtOAc in hexane). White solid; m.p.: 69-70 °C. ^{1}H NMR (500 MHz, $CDCl_3$): δ 7.08 – 6.97 (m, 4H), 4.47 (dd, J = 9.5, 4.8 Hz, 1H), 4.42 (d, J = 9.5 Hz, 1H), 2.50 (dtd, J = 5.6, 4.8, 0.7 Hz, 1H), 2.35 – 2.27 (m, 2H). $^{13}C\{^1H\}$ NMR (126 MHz, $CDCl_3$): δ 174.7, 161.9 (d, J = 246.0 Hz), 132.8, 127.6 (d, J = 8.2 Hz, 2C), 115.6 (d, J = 21.8 Hz, 2C), 69.6, 28.6, 27.3, 25.9. ^{19}F NMR (471 MHz, $CDCl_3$): δ -115.0. HRMS (ESI+): *m/z* calculated for $C_{11}H_9FO_2$ $[M+Na]^+$: 215.0479, found: 215.0476. Enantioenriched

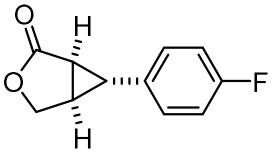

(–)-2b

product: 26.9 mg (70%). Enantiomeric excess (e.e.) was determined by chiral HPLC with Chiralcel AD-H column, Hexane/IPA 80/20; 1 mL/min; 25 °C: t_1 = 8.8 min (minor), t_2 = 9.7 min (major); e.e. = 89%. $[\alpha]_D^{25}$= -87.7° (*c* 0.17, $CHCl_3$). The absolute configuration of the major enantiomer was assigned by analogy with (–)-**2a** and (–)-**2c**.

(1*S*,5*R*,6*R*)-6-(4-methoxyphenyl)-3-oxabicyclo[3.1.0]hexan-2-one ((–)-2c). Racemic product: 40.3 mg

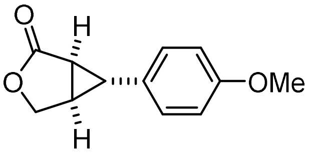

(–)-2c

(98%) after purification by flash chromatography (20 to 30% EtOAc in hexane). Brown solid; m.p.: 90-91 °C. ^{1}H NMR (500 MHz, $CDCl_3$): δ 7.03 – 6.97 (m, 2H), 6.88 – 6.82 (m, 2H), 4.46 (dd, *J* = 9.4, 4.8 Hz, 1H), 4.40 (d, *J* = 9.4 Hz, 1H), 3.79 (s, 3H), 2.52 – 2.45 (m, 1H), 2.30 (t, *J* = 3.4 Hz, 1H), 2.27 (ddd, *J* = 6.0, 2.8, 0.7 Hz, 1H). ^{13}C{^{1}H} NMR (126 MHz, $CDCl_3$): δ 175.2, 158.9, 129.1, 127.2 (2C), 114.2 (2C), 69.8, 55.4, 29.0, 27.2, 25.8. HRMS (ESI+): *m/z* calculated for $C_{12}H_{12}O_3$ $[M+H]^+$: 205.0859, found: 205.0848. Enantioenriched product: 30.2 mg (87%). Enantiomeric excess (e.e.) was determined by chiral HPLC with Chiralcel AD-H column, Hexane/IPA 80/20; 1 mL/min; 25 °C: t_1 = 10.2 min (minor), t_2 = 11.3 min (major); e.e. = 70%. $[\alpha]_D^{25}$= -80.3° (*c* 0.45, $CHCl_3$). The absolute configuration of the major enantiomer was established by comparison with literature data: $[\alpha]_D^{23}$= -43.1° (*c* 0.5, $CHCl_3$).[18]

(1*S*,5*R*,6*R*)-6-(1-methyl-1H-pyrazol-4-yl)-3-oxabicyclo[3.1.0]hexan-2-one ((–)-2d). Racemic product:

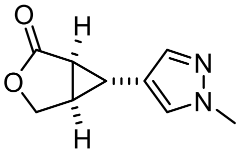

(–)-2d

29.2 mg (82%) after purification by flash chromatography (EtOAc). White solid; m.p.: 74-75 °C. ^{1}H NMR (500 MHz, $CDCl_3$): δ 7.27 (s, 1H), 7.21 (s, 1H), 4.42 (dd, *J* = 9.4, 4.7 Hz, 1H), 4.38 (d, *J* = 9.4 Hz, 1H), 3.86 (s, 3H), 2.40 (dtd, *J* = 5.6, 4.8, 0.8 Hz, 1H), 2.22 – 2.16 (m, 2H). ^{13}C{^{1}H} NMR (126 MHz, $CDCl_3$): δ 174.9, 137.1, 128.1, 118.2, 69.5, 39.0, 26.8, 25.4, 20.6. HRMS (ESI+): *m/z* calculated for $C_9H_{10}N_2O_2$ $[M+H]^+$: 179.0815, found: 179.0813. Enantioenriched product: 13.6 mg (38%). Enantiomeric excess (e.e.) was determined by chiral HPLC with Chiralcel AD-H column, Hexane/IPA 80/20; 1 mL/min; 25 °C: t_1 = 14.2 min (minor), t_2 = 15.9 min (major); e.e. = 52%. $[\alpha]_D^{23}$= -42.3° (*c* 0.42, $CHCl_3$). The absolute configuration of the major enantiomer was assigned by analogy with (–)-**2a** and (–)-**2c**.

(1*S*,5*R*,6*R*)-6-((*E*)-prop-1-en-1-yl)-3-oxabicyclo[3.1.0]hexan-2-one ((–)-2e). Racemic product: 26.8 mg

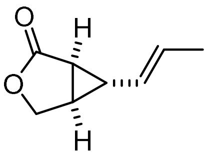

(–)-2e

(97%) after purification by flash chromatography (20 to 40% Et_2O in hexane). Colorless oil. ^{1}H NMR (500 MHz, $CDCl_3$): δ 5.70 – 5.59 (m, 1H), 5.07 – 4.96 (m, 1H), 4.34 (dd, *J* = 9.4, 4.8 Hz, 1H), 4.27 (d, *J* = 9.4 Hz, 1H), 2.18 (dt, *J* = 5.2, 4.2 Hz, 1H), 2.03 (dd, *J* = 6.0, 2.7 Hz, 1H), 1.83 – 1.76 (m, 1H), 1.67 (dd, *J* = 6.6, 1.6 Hz, 3H). ^{13}C{^{1}H} NMR (126 MHz, $CDCl_3$): δ 175.3, 127.7, 127.0, 69.4, 28.2, 25.0, 24.4, 17.8. HRMS (ESI+): *m/z* calculated for $C_8H_{10}O_2$ $[M+H]^+$: 139.0754, found: 139.0767. Enantioenriched product: 13.6 mg (38%). Enantiomeric excess (e.e.) was determined by chiral HPLC with Chiralcel AD-H column, Hexane/IPA 80/20; 1 mL/min; 25 °C: t_1 = 14.5

min (minor), t_2 = 16.1 min (major); e.e. = 69%. $[\alpha]_D^{23}$= -75.8° (*c* 0.27, $CHCl_3$). The absolute configuration of the major enantiomer was established by comparison with literature data: $[\alpha]_D^{23}$= -87.6° (*c* 1.14, $CHCl_3$).[18]

(1*S*,5*R*,6*S*)-6-Phenyl-3-oxabicyclo[3.1.0]hexan-2-one ((+)-2f). Racemic product: 25.9 mg (74%) after purification by flash chromatography (20% EtOAc in hexane). White solid; m.p.: 64-65 °C. ^{1}H NMR (500 MHz, $CDCl_3$): δ 7.39 – 7.27 (m, 5H), 4.37 (ddd, *J* = 9.8, 3.6, 1.7 Hz, 1H), 4.06 (dd, *J* = 9.8, 0.6 Hz, 1H), 2.78 (t, *J* = 8.4 Hz, 1H), 2.62 – 2.56 (m, 2H). $^{13}C\{^1H\}$ NMR (126 MHz, $CDCl_3$): δ 174.8, 132.3, 129.4 (2C), 128.9, 127.8 (2C), 65.8, 26.3, 23.9, 23.5. HRMS (ESI+): *m/z* calculated for $C_{11}H_{10}O_2$ $[M+Na]^+$: 197.0573, found 197.0573. Enantioenriched product: 29.9 mg (86%). Enantiomeric excess (e.e.) was determined by chiral HPLC with Chiralcel OD column, Hexane/IPA 80/20; 1 mL/min; 25 °C: t_1 = 9.8 min (minor), t_2 = 10.8 min (major); e.e. = 90%. Recrystallised from EtOAc/hexane (1:1) in 62% yield to give a sample with 99% e.e. $[\alpha]_D^{25}$= +72.7° (*c* 0.19, $CHCl_3$). The absolute configuration of the major enantiomer was established by comparison with literature data: $[\alpha]_D^{23}$= +85.6° (*c* 1.09, $CHCl_3$).[19]

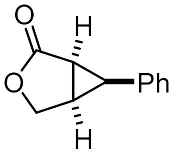

(+)-2f

(1*S*,5*R*,6*S*)-6-(4-fluorophenyl)-3-oxabicyclo[3.1.0]hexan-2-one ((+)-2g). Racemic product: 29.9 mg (78%) after purification by flash chromatography (10 to 20% EtOAc in hexane). White solid; m.p.: 93-94 °C. ^{1}H NMR (500 MHz, $CDCl_3$): δ 7.32 –7.27 (m, 2H), 7.07 – 7.01 (m, 2H), 4.38 (dd, *J* = 9.9, 4.7 Hz, 1H), 4.03 (d, *J* = 9.9 Hz, 1H), 2.73 (t, *J* = 8.4 Hz, 1H), 2.62 – 2.54 (m, 2H). $^{13}C\{^1H\}$ NMR (126 MHz, $CDCl_3$): δ 174.6, 162.3 (d, *J* = 246.7 Hz), 131.1 (d, *J* = 8.2 Hz, 2C), 128.0 (d, *J* = 3.3 Hz), 116.0 (d, *J* = 21.6 Hz, 2C), 65.7, 25.4, 24.0, 23.5. ^{19}F NMR (471 MHz, $CDCl_3$): δ -114.0. HRMS (ESI+): *m/z* calculated for $C_{11}H_9FO_2$ $[M+Na]^+$: 215.0479, found: 215.0476. Enantioenriched product: 28.8 mg (75%). Enantiomeric excess (e.e.) was determined by chiral HPLC with Chiralcel AD-H column, Hexane/IPA 95/5; 1 mL/min; 25 °C: t_1 = 19.6 min (minor), t_2 = 22.7 min (major); e.e. = 90%. $[\alpha]_D^{25}$= +77.1 (*c* 0.33, $CHCl_3$). The absolute configuration of the major enantiomer was assigned by analogy with (+)-**2f**.

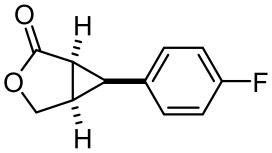

(+)-2g

(1*S*,5*R*,6*S*)-6-(4-methoxyphenyl)-3-oxabicyclo[3.1.0]hexan-2-one ((+)-2h). Racemic product: 31.8 mg (78%) after purification by flash chromatography (20 to 30% EtOAc in hexane). White solid; m.p.: 84-85 °C. ^{1}H NMR (500 MHz, $CDCl_3$): δ 7.25 – 7.21 (m, 2H), 6.90 – 6.85 (m, 2H), 4.36 (dd, *J* = 9.8, 4.7 Hz, 1H), 4.05 (d, *J* = 9.8 Hz, 1H), 3.79 (s, 3H), 2.71 (t, *J* = 8.4 Hz, 1H), 2.59 – 2.51 (m, 2H). $^{13}C\{^1H\}$ NMR (126 MHz, $CDCl_3$): δ 175.0, 159.1, 130.5 (2C), 124.1, 114.4 (2C), 65.8, 55.2, 25.5, 24.1, 23.6. HRMS (ESI+): *m/z* calculated for $C_{12}H_{12}O_3$ $[M+Na]^+$: 227.0679, found: 227.0675. Enantioenriched product: 33.7 mg (83%). Enantiomeric excess (e.e.) was determined by chiral HPLC with Chiralcel AD-H column, Hexane/IPA 80/20; 1 mL/min; 25 °C: t_1 = 9.0 min (minor), t_2 = 10.3 min (major); e.e. = 90%. $[\alpha]_D^{25}$= +71.4° (*c* 0.33, $CHCl_3$). The absolute configuration of the major enantiomer was assigned by analogy with (+)-**2f**.

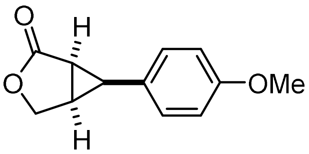

(+)-2h

(1*R*,5*S*,6*R*)-6-methyl-6-(4-methylpent-3-en-1-yl)-3-oxabicyclo[3.1.0]hexan-2-one ((–)-2i). Racemic product: 33 mg (85%) after purification by flash chromatography (5 to 20% EtOAc in Hexane). Colorless oil. ^{1}H NMR (500 MHz, $CDCl_3$): δ 5.11 – 5.01 (m, 1H), 4.37 (dd, J = 9.9, 5.5 Hz, 1H), 4.14 (d, J = 9.9 Hz, 1H), 2.13 – 2.06 (m, 2H), 2.06 – 2.02 (m, 1H), 1.96 (dd, J = 6.4, 0.8 Hz, 1H), 1.68 (s, 3H), 1.61 (s, 3H), 1.37 (ddd, J = 13.9, 9.3, 6.7 Hz, 1H), 1.28 (ddd, J = 13.8, 9.5, 6.9 Hz, 1H), 1.17 (s, 3H). ^{13}C{^{1}H} NMR (126 MHz, $CDCl_3$): δ 174.9, 132.4, 123.2, 66.4, 39.3, 29.8, 29.3, 26.7, 25.7, 24.9, 17.6, 11.7. HRMS (ESI+): *m/z* calculated for $C_{12}H_{18}O_2$ [M+H$^+$]: 195.1380; found: 195.1376. Enantioenriched product: 34.4 mg (89%). Enantiomeric excess (e.e.) was determined by chiral HPLC with Chiralcel OD column, Hexane/IPA 98/2; 1 mL/min; 25 °C: t_1 = 10.2 min (minor), t_2 = 11.3 min (major); e.e. = 91%. $[\alpha]_D^{23}$= -87.7 (*c* 0.17, $CHCl_3$). The absolute configuration of the major enantiomer was established by comparison with literature data for the (1*S*,5*R*,6*S*) enantiomer: $[\alpha]_D^{23}$= +56.1° (*c* 1.44, $CHCl_3$).[17]

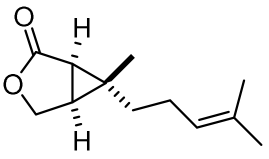

(–)-2i

(1*R*,5*S*,6*S*)-6-methyl-6-(4-methylpent-3-en-1-yl)-3-oxabicyclo[3.1.0]hexan-2-one ((–)-2j). Racemic product: 33 mg (85%) after purification by flash chromatography (5 to 20% EtOAc in Hexane). Colorless oil. ^{1}H NMR (500 MHz, $CDCl_3$): δ 5.12 (t, J = 6.7 Hz, 1H), 4.35 (dd, J = 9.9, 5.7 Hz, 1H), 4.15 (d, J = 10.0 Hz, 1H), 2.14 (q, J = 7.8 Hz, 2H), 2.06 (t, J = 5.9 Hz, 1H), 1.96 (d, J = 6.3 Hz, 1H), 1.68 (s, 3H), 1.61 (s, 3H), 1.48 (dt, J = 15.9, 8.1 Hz, 1H), 1.40 (dt, J = 15.9, 8.1 Hz, 1H), 1.16 (s, 3H). ^{13}C{^{1}H} NMR (126 MHz, $CDCl_3$): δ 174.8, 132.4, 123.3, 66.5, 30.82, 30.76, 28.4, 26.9, 25.7, 24.9, 22.6, 17.6. HRMS (ESI+): *m/z* calculated for $C_{12}H_{18}O_2$ [M+H$^+$]: 195.1380; found: 195.1377. Enantioenriched product: 37.2 mg (96%). Enantiomeric excess (e.e.) was determined by chiral HPLC with Chiralcel AD-H column, Hexane/IPA 98/2; 1 mL/min; 25 °C: t_1 = 12.3 min (minor), t_2 = 14.5 min (major); e.e. = 90%. $[\alpha]_D^{23}$= -46.7° (*c* 0.23, $CHCl_3$). The absolute configuration of the major enantiomer was established by comparison with literature data for the (1*S*,5*R*,6*R*) enantiomer: $[\alpha]_D^{23}$= +40.2° (*c* 1.74, $CHCl_3$).[17]

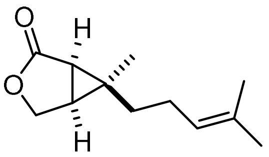

(–)-2j

(1*S*,5*R*,6*R*)-6-phenethyl-3-oxabicyclo[3.1.0]hexan-2-one ((–)-2k). Racemic product: 66 mg (82%) after purification by flash chromatography (20% EtOAc in Hexane). Note: the reaction was conducted on **1k** (112 mg, 0.4 mmol, 1 equiv). Orange oil. ^{1}H NMR (500 MHz, $CDCl_3$): δ 7.33 – 7.27 (m, 2H), 7.24 – 7.19 (m, 1H), 7.17 (dd, J = 7.8, 0.9 Hz, 2H), 4.26 (dd, J = 9.2, 4.9 Hz, 1H), 4.16 (d, J = 9.3 Hz, 1H), 2.83 – 2.69 (m, 1H), 1.95 (dt, J = 5.1, 4.4 Hz, 1H), 1.86 (ddd, J = 5.9, 2.7, 0.7 Hz, 1H), 1.76 – 1.59 (m, 2H), 1.20 (tdd, J = 6.8, 3.8, 2.9 Hz, 1H). ^{13}C{^{1}H} NMR (126 MHz, $CDCl_3$): δ 175.8, 140.9, 128.5 (2C), 128.4 (2C), 126.2, 69.4, 35.0, 33.0, 25.8, 24.1, 23.8. HRMS (ESI+): *m/z* calculated for $C_{13}H_{14}O_2$ [M+H]$^+$: 203.1067, found: 203.1068. Enantioenriched product: 16.5 mg (41%). Note: the reaction was conducted on **1k** (66 mg, 0.2 mmol, 1 equiv) with a slow addition over 9 h (syringe pump 0.33 mL/h) and then stirred for 96 h. Enantiomeric excess (e.e.) was determined by chiral HPLC with Chiralcel OD column, Hexane/IPA 80/20; 1 mL/min; 25 °C: t_1 = 10.4 min (minor), t_2 = 11.9 min

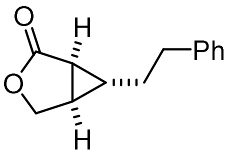

(–)-2k

(major); e.e. = 77%. $[\alpha]_D^{23}$= –14.0° (*c* 0.20, $CHCl_3$). The absolute configuration of the major enantiomer was assigned by analogy with (–)-**2a** and (–)-**2c**.

(3aS,3bS,6aR)-tetrahydro-1H-cyclopenta[1,3]cyclopropa[1,2-c]furan-3(3aH)-one ((+)-2l). Racemic product: 19.4 mg (70%) after purification by flash chromatography (10 to 30% EtOAc in Hexane). Orange oil. ^{1}H NMR (500 MHz, $CDCl_3$): δ 4.38 – 4.34 (m, 2H), 1.99 (dd, J = 12.3, 7.7 Hz, 1H), 1.95 – 1.83 (m, 4H), 1.81 – 1.72 (m, 1H), 1.64 – 1.60 (m, 1H), 1.21 – 1.08 (m, 1H). ^{13}C{^{1}H} NMR (126 MHz, $CDCl_3$): δ 176.3, 69.9, 38.6, 29.0, 27.2, 26.5, 25.3, 21.0. HRMS (ESI+): *m/z* calculated for $C_8H_{10}O_2$ [M+H$^+$]: 139.0754, found: 139.0761. Enantioenriched product: 9.3 mg (34%). Enantiomeric excess (e.e.) was determined by chiral HPLC with Chiralcel AD-H column, Hexane/IPA 90/10; 1 mL/min; 25 °C: t_1 = 8.5 min (minor), t_2 = 9.4 min (major); e.e. = 79%. $[\alpha]_D^{23}$= +27.5° (*c* 0.05, $CHCl_3$). The absolute configuration is assumed.

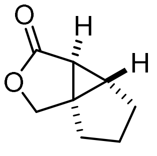

(+)-2l

(1*R*,5*R*,6*R*)-6-phenylbicyclo[3.1.0]hexan-2-one ((–)-2m). Racemic product: 33.9 mg (98%) after purification by flash chromatography (10% EtOAc in hexane). White solid; m.p.: 66-67 °C. ^{1}H NMR (500 MHz, $CDCl_3$): δ 7.31 – 7.26 (m, 2H), 7.23 – 7.18 (m, 1H), 7.08 – 7.02 (m, 1H), 2.42 – 2.35 (m, 2H), 2.34 – 2.11 (m, 4H), 2.06 (ddd, J = 7.2, 3.6, 2.1 Hz, 1H). ^{13}C{^{1}H} NMR (126 MHz, $CDCl_3$): δ 213.2, 138.9, 128.5 (2C), 126.6, 125.9 (2C), 39.0, 32.4, 30.73, 30.66, 23.3. HRMS (ESI+): *m/z* calculated for $C_{12}H_{12}O$ [M+H]$^+$: 173.0966, found: 173.0959. Enantioenriched product: 34.2 mg (98%). Enantiomeric excess (e.e.) was determined by chiral HPLC with Chiralcel AD-H column, Hexane/IPA 80/20; 1 mL/min; 25 °C: t_1 = 7.2 min (minor), t_2 = 8.0 min (major); e.e. = 93%. $[\alpha]_D^{23}$= –87.7 (*c* 0.17, $CHCl_3$). The absolute configuration of the major enantiomer was established by comparison with literature data: $[\alpha]_D^{23}$= -111° (*c* 0.53, $CHCl_3$).[20]

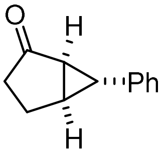

(–)-2m

(1*S*,5*R*,6*R*)-3-benzyl-6-(4-methoxyphenyl)-3-azabicyclo[3.1.0]hexan-2-one ((–)-2n). Racemic product: 50.7 mg (86%) after purification by flash chromatography (2% MeOH in DCM). White solid; m.p.: 101-102 °C. ^{1}H NMR (500 MHz, $CDCl_3$): δ 7.37 – 7.32 (m, 2H), 7.32 – 7.27 (m, 1H), 7.25 – 7.21 (m, 2H), 7.00 – 6.95 (m, 2H), 6.83 – 6.78 (m, 2H), 4.41 (d, J = 14.8 Hz, 1H), 4.37 (d, J = 14.8 Hz, 1H), 3.78 (s, 3H), 3.50 (dd, J = 10.6, 5.9 Hz, 1H), 3.33 (dd, J = 10.5, 1.4 Hz, 1H), 2.21 (ddd, J = 6.4, 2.7, 1.7 Hz, 1H), 2.04 (td, J = 6.1, 3.7 Hz, 1H), 2.00 (t, J = 3.2 Hz, 1H).^{13}C{^{1}H} NMR (126 MHz, $CDCl_3$): δ 173.5, 158.4, 136.7, 130.9, 128.7 (2C), 128.2 (2C), 127.6, 127.1 (2C), 113.9 (2C), 55.3, 49.0, 46.3, 30.3, 29.3, 20.9. HRMS (ESI+): *m/z* calculated for $C_{19}H_{19}NO_2$ [M+H]$^+$: 294.1489, found: 294.1475. Enantioenriched product: 50.8 mg (87%). Enantiomeric excess (e.e.) was determined by chiral HPLC with Chiralcel AD-H column, Hexane/IPA 80/20; 1 mL/min; 25 °C: t_1 = 12.8 min (minor), t_2 = 17.4 min (major); e.e. = 61%. $[\alpha]_D^{23}$= -45.7° (*c* 0.44, $CHCl_3$). The absolute configuration of the major enantiomer was assigned by analogy with (–)-**2a** and (–)-**2c**.

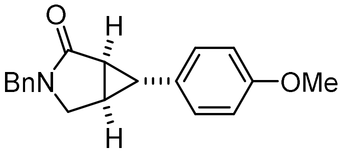

(–)-2n

(1*R*,6*R*,7*R*)-7-phenyl-3-oxabicyclo[4.1.0]heptan-2-one ((–)-2o). Racemic product: 12 mg (32%) after

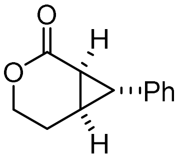

(–)-2o

purification by flash chromatography (20% EtOAc in Hexane). Colorless oil. ^{1}H NMR (500 MHz, $CDCl_3$): δ 7.35 – 7.27 (m, 2H), 7.26 – 7.21 (m, 1H), 7.14 – 7.06 (m, 2H), 4.35 (ddt, J = 12.1, 6.2, 1.3 Hz, 1H), 4.27 (td, J = 12.6, 3.6 Hz, 1H), 2.93 (t, J = 4.4 Hz, 1H), 2.32 – 2.22 (m, 1H), 2.20 – 2.16 (m, 1H), 2.16 – 2.09 (m, 2H). $^{13}C\{^{1}H\}$ NMR (126 MHz, $CDCl_3$): δ 169.4, 138.0, 128.7 (2C), 127.0, 126.2 (2C), 64.5, 26.6, 24.9, 23.6, 20.4. HRMS (ESI+): *m/z* calculated for $C_{12}H_{12}O_2$ $[M+H]^+$: 189.0910; found: 189.0907. Enantioenriched product: 14.6 mg (39%). Note: the reaction was conducted on **1o** with a slow addition over 9 h (syringe pump 0.33 mL/h) and then stirred for 96 h. Enantiomeric excess (e.e.) was determined by chiral HPLC with Chiralcel AD-H column, Hexane/IPA 80/20; 1 mL/min; 25 °C: t_1 = 8.8 min (minor), t_2 = 10.0 min (major); e.e. = 98%. $[\alpha]_D^{23}$= –126.3° (*c* 0.28, $CHCl_3$). The absolute configuration of the major enantiomer was assigned by analogy with (–)-**2a** and (–)-**2c**.

Table S2. Representative examples of asymmetric cyclopropanation of *E* and *Z* allyl diazo acetates.

Catalyst	Substrate	Product	Reference
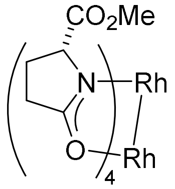	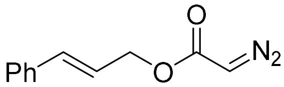	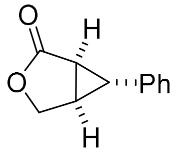 78% yield 68% e.e.	17
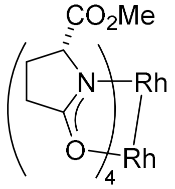	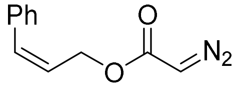	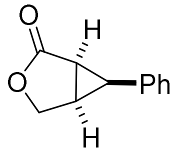 70% yield 94% e.e.	17
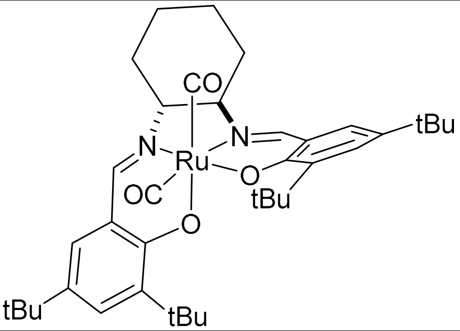	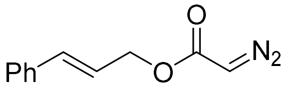	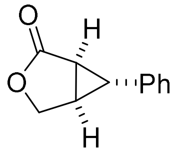 91% yield 98% e.e.	21
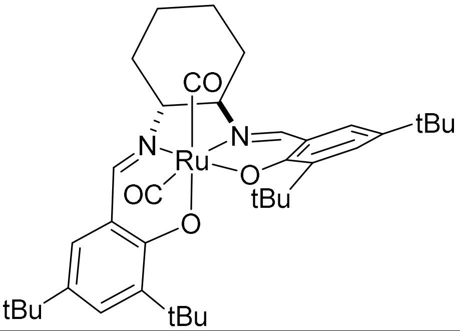	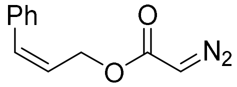	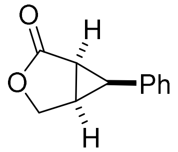 48% yield 51% e.e.	21
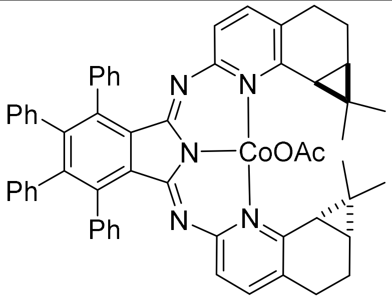	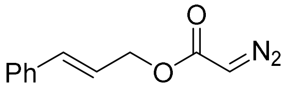	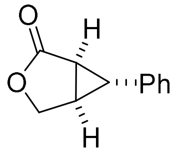 89% yield 93% e.e.	22
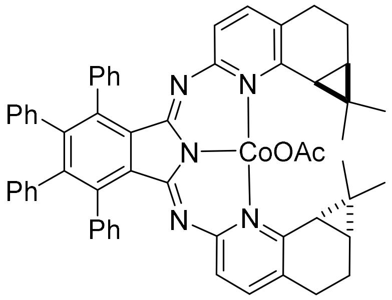	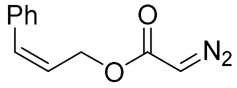	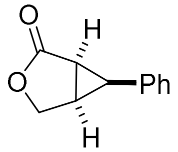 42% yield 65% e.e.	22

Synthesis of cyclopropane derivative (±)-9

(1*S*,2*R*,3*R*)-2-(hydroxymethyl)-3-(1-methyl-1*H*-pyrazol-4-yl)-*N*-(pyridin-2-yl)cyclopropane-1-

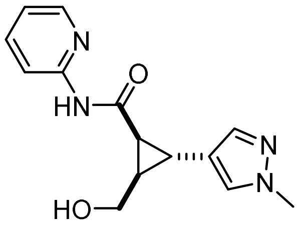

(±)-**9**

carboxamide ((±)-9). Following a reported procedure,[23] a solution of LiHMDS (0.34 ml, 0.34 mmol, 1M in THF, 3 equiv) was added to an oven-dried Schlenk tube charged with lactone (±)-**2d** (20 mg, 0.11 mmol, 1 equiv) and 2-aminopyridine (32 mg, 0.34 mmol, 3 equiv). Toluene (1.2 mL) was added with vigorous stirring at room temperature, and the reaction mixture was stirred at room temperature overnight. Then the reaction mixture was quenched with a saturated aqueous NH_4Cl solution (3 mL), diluted with EtOAc (20 mL), and the organic layer was washed with water (5 mL), brine (5 mL), and dried over $MgSO_4$, before filtration and evaporation under vacuum. Purification by silica gel column chromatography (0 to 20% EtOAc in hexane) gave amide (±)-**9** (21.3 mg (71%)) as a brown thick oil. ^{1}H NMR (500 MHz, $CDCl_3$): δ 8.59 (br s, 1H (NH)), 8.25 (d, J = 4.7 Hz, 1H), 8.17 (d, J = 8.4 Hz, 1H), 7.76 – 7.69 (m, 1H), 7.29 (s, 1H), 7.21 (s, 1H), 7.05 (dd, J = 6.8, 5.4 Hz, 1H), 4.13 (dd, J = 12.0, 4.2 Hz, 1H), 3.96 (dd, J = 12.0, 7.7 Hz, 1H), 3.86 (s, 3H), 2.72 – 2.65 (m, 1H), 1.96 – 1.88 (m, 1H), 1.84 (dd, J = 8.5, 5.0 Hz, 1H). ^{13}C{^{1}H} NMR (126 MHz, $CDCl_3$): δ 170.4, 151.0, 147.4, 138.7, 137.2, 128.1, 120.8, 119.9, 114.2, 59.5, 39.0, 32.4, 31.3, 20.0. HRMS (ESI+): *m/z* calculated for $C_{14}H_{16}N_4O_2$ $[M+H]^+$: 273.1346, found 273.1346.

X-Ray crystallography

Preparation of X-ray suitable crystals

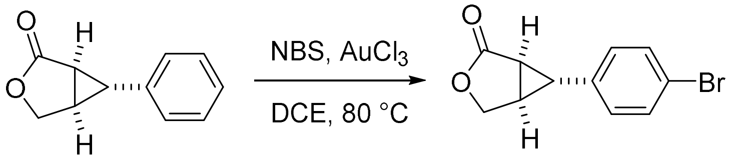

(–)-**2a** (–)-**SI-2a-Br**

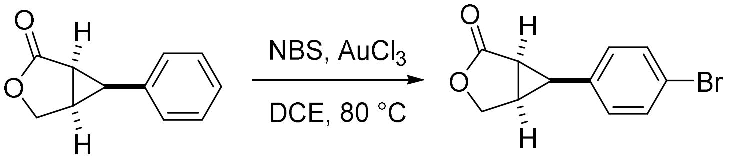

(+)-**2f** (+)-**SI-2f-Br**

(1*S*,5*R*,6*R*)-6-(4-bromophenyl)-3-oxabicyclo[3.1.0]hexan-2-one ((–)-SI-2a-Br). Adapting a reported procedure,[24] a flame-dried Schlenk tube was charged with N-bromosuccinimide (22 mg, 0.19 mmol, 1.1 equiv), $AuCl_3$ (1.0 mg, 3.3 µmol, 2 mol%) and dry DCE (0.5 mL). Lactone **(–)-2a** (30 mg, 0.17 mmol, 1 equiv) was then added and the mixture stirred for 3 days at 80 °C. The solution was then concentrated under vacuum and the residue was purified by silica gel column (10 to 20% EtOAc in hexane) to yield 24.3 mg (56%) of **(–)-SI-2a-Br** as light-brown solid. m.p.: 119-123 °C. ^{1}H NMR (500 MHz, $CDCl_3$) δ 7.47 – 7.41 (m, 2H), 6.97 – 6.92 (m, 2H), 4.47 (dd, J = 9.5, 4.7 Hz, 1H), 4.41 (d, J = 9.5 Hz, 1H), 2.51 (dd, J = 10.3, 4.4 Hz, 1H), 2.32 (dd, J = 6.1, 2.8 Hz, 1H), 2.29 (t, J = 3.3 Hz, 1H). ^{13}C{^{1}H} NMR (126 MHz, $CDCl_3$): δ 174.6, 136.2, 131.9 (2C), 127.6 (2C), 121.1, 69.6, 28.8, 27.4, 26.1. HRMS (ESI$^+$): *m/z* calculated for $C_{11}H_9BrO_2$ [M+Na]$^+$: 274.9678, found 274.9674. $[\alpha]_D^{19}$ = –118° (*c* 0.27, $CHCl_3$). Crystals suitable for X-ray analysis were obtained after recrystallisation by dilution of the sample in minimum amount of EtOAc and slow evaporation of that solvent at room temperature. The X-Ray crystal structure of the enantiomer of this compound has previously been determined.[25]

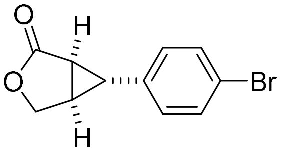

(–)-**SI-2a-Br**

(1*S*,5*R*,6*S*)-6-(4-bromophenyl)-3-oxabicyclo[3.1.0]hexan-2-one ((+)-SI-2f-Br). This compound was obtained (18.4 mg (63%)) from lactone **(+)-2f** (20 mg, 0.12 mmol, 1 equiv) by following the procedure described for the preparation of **(–)-SI-2a-Br**. Light-brown solid; m.p.: 100-104 °C. ^{1}H NMR (500 MHz, $CDCl_3$): δ 7.48 (d, J = 8.3 Hz, 2H), 7.20 (d, J = 8.3 Hz, 2H), 4.38 (dd, J = 9.9, 4.2 Hz, 1H), 4.02 (d, J = 9.9 Hz, 1H), 2.71 (t, J = 8.4 Hz, 1H), 2.62 – 2.55 (m, 2H). ^{13}C{^{1}H} NMR (126 MHz, $CDCl_3$): δ 174.5, 132.2 (2C), 131.3, 131.2 (2C), 122.0, 65.7, 25.6, 23.9, 23.5. HRMS (ESI$^+$): *m/z* calculated for $C_{11}H_9BrO_2$ [M+Na]$^+$: 274.9678, found 274.9671. $[\alpha]_D^{19}$ = +91.3° (*c* 0.25, $CHCl_3$). Crystals suitable for X-ray analysis were obtained after

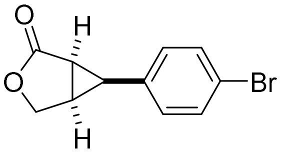

(+)-**SI-2f-Br**

recrystallisation by dilution of the sample in minimum amount of EtOAc and slow evaporation of that solvent at room temperature.

X-ray crystallography method and data

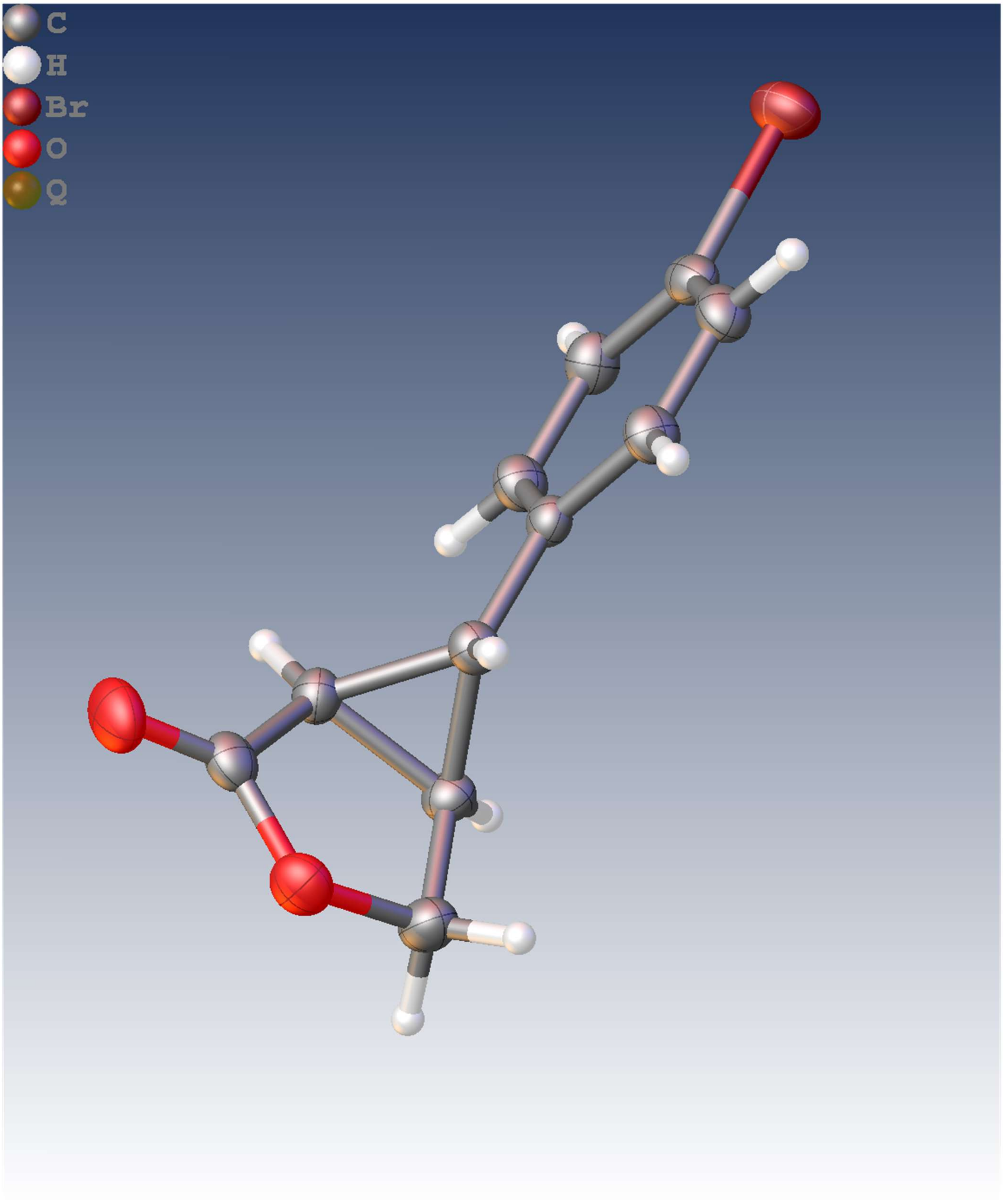

Figure S1. Thermal ellipsoid plot of **(–)-SI-2a-Br**. Ellipsoids drawn at 50% probability.

Single crystals of **(–)-SI-2a-Br** were submitted for X-ray structural determination. A suitable crystal was selected and mounted onto a MiTeGen tip using Parabar oil and placed on a Bruker Venture D8 diffractometer. The crystal was kept at 200.0 K during data collection. Using Olex2,[26] the structure was solved with the XT structure solution program[27] using Intrinsic Phasing and refined with the XL[28] refinement package using Least Squares minimisation. Absolute structure determination was carried out via anomalous dispersion, yielding a Flack parameter: -0.012(6) and a Hooft parameter: -0.012(6).[29]

Table S3. Crystal data and structure refinement for **(–)-SI-2a-Br**.

Identification code	LV_03_071_0m
Empirical formula	$C_{11}H_9O_2Br$
Formula weight	253.09
Temperature/K	200.0
Crystal system	monoclinic
Space group	$P2_1$
a/Å	5.6614(3)
b/Å	9.7492(5)
c/Å	8.9751(4)
α/°	90
β/°	97.263(2)
γ/°	90
Volume/Å^3	491.40(4)
Z	2
ρ_{calc}g/cm^3	1.711
μ/mm^{-1}	4.150
F(000)	252.0
Crystal size/mm^3	0.24 × 0.1 × 0.06
Radiation	MoKα (λ = 0.71073)
2Θ range for data collection/°	4.576 to 52.856
Index ranges	$-7 \leq h \leq 6$, $-12 \leq k \leq 12$, $-11 \leq l \leq 11$
Reflections collected	5710
Independent reflections	1975 [R_{int} = 0.0185, R_{sigma} = 0.0206]
Data/restraints/parameters	1975/1/127
Goodness-of-fit on F^2	1.054
Final R indexes [I>=2σ (I)]	R_1 = 0.0224, wR_2 = 0.0549
Final R indexes [all data]	R_1 = 0.0232, wR_2 = 0.0554
Largest diff. peak/hole / e Å^{-3}	0.27/-0.43
Flack parameter	-0.012(6)

Table S4. Fractional Atomic Coordinates (×10^4) and Equivalent Isotropic Displacement Parameters ($Å^2$×10^3) for **(–)-SI-2a-Br**. U_{eq} is defined as 1/3 of the trace of the orthogonalised U_{IJ} tensor.

Atom	*x*	*y*	*z*	U(eq)
Br1	1423.7(6)	7134.5(5)	11326.1(3)	38.53(13)
O1	9798(5)	2683(3)	5062(3)	41.9(7)
O2	8607(4)	4365(3)	3447(3)	33.8(6)
C11	3004(6)	5169(4)	7487(4)	27.0(7)
C6	5064(6)	5832(3)	7152(4)	21.8(6)
C3	8354(6)	3555(4)	4637(4)	28.2(7)
C9	2878(6)	6626(4)	9608(4)	26.9(7)
C8	4897(6)	7301(5)	9315(4)	29.7(7)
C7	5990(6)	6904(4)	8080(4)	27.4(8)
C5	5045(6)	5082(4)	4346(4)	25.2(7)
C1	6333(6)	5436(3)	5866(4)	23.7(6)
C2	6207(6)	3950(3)	5306(4)	25.1(7)
C10	1898(6)	5571(4)	8709(4)	28.1(7)
C4	6550(7)	5245(4)	3079(4)	32.2(8)

Table S5. Anisotropic Displacement Parameters ($Å^2$×10^3) for **(–)-SI-2a-Br**. The Anisotropic displacement factor exponent takes the form: $-2\pi^2[h^2a^{*2}U_{11}+2hka^*b^*U_{12}+\ldots]$.

Atom	U_{11}	U_{22}	U_{33}	U_{23}	U_{13}	U_{12}
Br1	38.8(2)	49.1(2)	28.51(17)	-4.2(2)	7.28(12)	9.2(2)
O1	38.4(14)	38.6(14)	48.0(16)	-7.6(12)	2.4(12)	15.1(12)
O2	31.6(14)	37.8(15)	33.7(14)	-1.8(11)	11.0(11)	3.3(11)
C11	27.0(17)	25.1(16)	28.4(16)	-3.3(13)	1.5(13)	-2.0(13)
C6	21.7(15)	20.0(14)	23.3(15)	1.3(12)	1.4(12)	2.9(12)
C3	27.1(17)	25.8(16)	30.7(17)	-7.2(13)	0.3(14)	-0.2(14)
C9	27.5(17)	30.0(16)	22.5(16)	1.2(12)	1.0(13)	7.5(13)
C8	31.6(15)	27(2)	29.0(14)	-5.5(16)	-2.8(12)	0.2(17)
C7	25.8(15)	26(2)	29.8(15)	-2.9(14)	1.4(12)	-4.0(13)
C5	24.3(17)	27.2(17)	23.4(16)	1.6(13)	0.3(13)	2.1(14)
C1	22.3(16)	23.6(16)	25.3(16)	-1.2(13)	2.9(12)	-1.1(13)
C2	25.2(17)	22.0(16)	27.7(17)	0.0(13)	1.9(14)	1.2(13)
C10	24.7(16)	25.6(17)	34.2(18)	1.0(14)	4.6(14)	-1.2(13)
C4	36.5(19)	33.6(19)	26.9(17)	3.8(14)	5.7(15)	5.0(16)

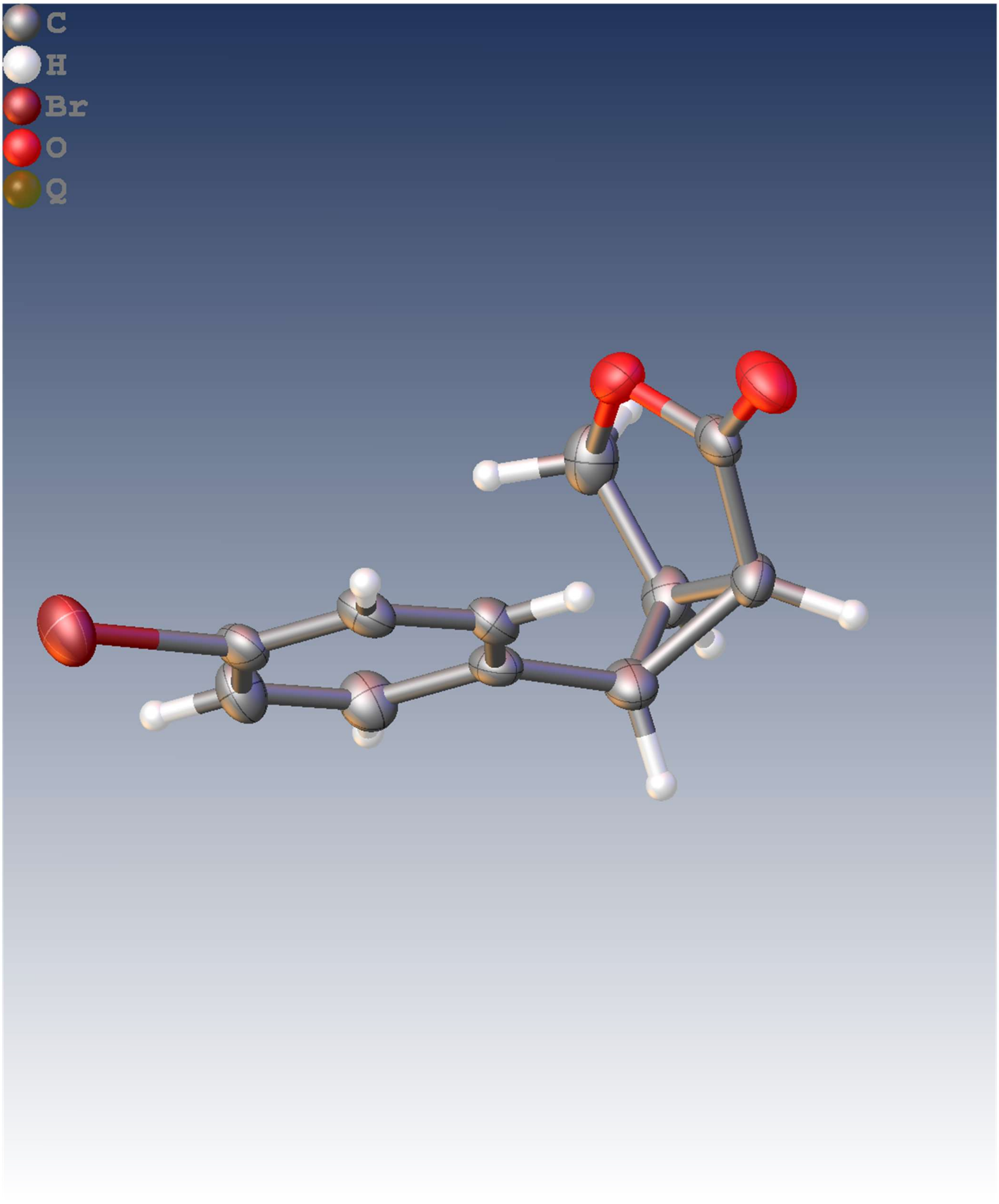

Figure S2. Thermal ellipsoid plot of **(+)-SI-2f-Br**. Ellipsoids drawn at 50% probability.

Single crystals of **(+)-SI-2f-Br** were submitted for X-ray structural determination. A suitable crystal was selected and mounted on a MiTeGen tip using Parabar oil and placed on a Bruker Venture D8 diffractometer. The crystal was kept at 200.02 K during data collection. Using Olex2,[26] the structure was solved with the

olex2.solve structure solution program[30] using Charge Flipping and refined with the SHELXL[27] refinement package using Least Squares minimisation. Absolute structure determination was carried out via anomalous dispersion, yielding a Flack parameter: -0.001(8) and a Hooft parameter: 0.023(6).[29]

Table S6. Crystal data and structure refinement for **(+)-SI-2f-Br**.

Identification code	LV_03_057_0m
Empirical formula	$C_{11}H_9BrO_2$
Formula weight	253.09
Temperature/K	200.02
Crystal system	monoclinic
Space group	$P2_1$
a/Å	7.7934(6)
b/Å	5.9246(4)
c/Å	10.5160(8)
α/°	90
β/°	93.823(3)
γ/°	90
Volume/Å^3	484.47(6)
Z	2
ρ_{calc}g/cm^3	1.735
μ/mm^{-1}	4.210
F(000)	252.0
Crystal size/mm^3	0.45 × 0.09 × 0.02
Radiation	MoKα (λ = 0.71073)
2Θ range for data collection/°	5.238 to 52.788
Index ranges	-9 ≤ h ≤ 9, -7 ≤ k ≤ 7, -13 ≤ l ≤ 12
Reflections collected	6514
Independent reflections	1974 [R_{int} = 0.0295, R_{sigma} = 0.0294]
Data/restraints/parameters	1974/1/127
Goodness-of-fit on F^2	1.061
Final R indexes [I>=2σ (I)]	R_1 = 0.0289, wR_2 = 0.0636
Final R indexes [all data]	R_1 = 0.0331, wR_2 = 0.0654
Largest diff. peak/hole / e Å^{-3}	0.68/-0.38
Flack parameter	-0.001(8)

Table S7. Fractional Atomic Coordinates (×104) and Equivalent Isotropic Displacement Parameters (Å2×103) for **(+)-SI-2f-Br**. Ueq is defined as 1/3 of the trace of the orthogonalized UIJ tensor.

**Atom**	***x***	***y***	***z***	**U(eq)**
Br1	8794.8(6)	-619.4(9)	9325.8(4)	41.28(16)
O2	2644(4)	2663(6)	5136(3)	32.7(8)
O1	463(5)	1230(6)	6146(4)	40.0(9)
C6	4364(5)	4139(10)	7746(4)	25.1(9)
C5	2716(6)	6419(8)	5896(5)	30.5(11)

Table S7. Fractional Atomic Coordinates (×104) and Equivalent Isotropic Displacement Parameters (Å2×103) for **(+)-SI-2f-Br**. Ueq is defined as 1/3 of the trace of the orthogonalized UIJ tensor.

**Atom**	***x***	***y***	***z***	**U(eq)**
C3	1377(6)	2821(9)	5955(4)	27.6(10)
C4	3402(6)	4847(9)	4935(5)	35.7(13)
C11	6090(6)	4744(9)	7700(4)	32.6(13)
C1	2997(6)	5762(8)	7281(5)	30.2(10)
C10	7397(6)	3343(8)	8175(4)	31.8(11)
C2	1350(6)	5068(7)	6519(5)	27.2(11)
C8	5291(6)	665(8)	8795(4)	27.9(10)
C7	3988(6)	2077(8)	8306(4)	27.3(10)
C9	6985(6)	1329(8)	8711(4)	28.0(10)

Table S8. Anisotropic Displacement Parameters ($Å^2×10^3$) for **(+)-SI-2f-Br**. The Anisotropic displacement factor exponent takes the form: $-2\pi^2[h^2a^{*2}U_{11}+2hka^*b^*U_{12}+\ldots]$.

**Atom**	**U_{11}**	**U_{22}**	**U_{33}**	**U_{23}**	**U_{13}**	**U_{12}**
Br1	34.5(2)	40.4(3)	47.2(3)	7.5(3)	-10.34(17)	1.3(3)
O2	32.6(18)	30.6(19)	34.4(18)	-6.9(15)	-1.2(15)	7.6(15)
O1	34.2(19)	31(2)	53(2)	-1.8(17)	-10.5(16)	-4.9(16)
C6	29.5(19)	20(3)	25.6(18)	-4(2)	-3.7(15)	-1(2)
C5	27(2)	22(2)	42(3)	5(2)	-1(2)	3.1(19)
C3	23(2)	26(3)	33(2)	-1(2)	-8.9(19)	2(2)
C4	31(2)	39(4)	37(2)	10(2)	3.7(18)	4(2)
C11	33(2)	26(4)	38(2)	4(2)	-0.5(18)	-4(2)
C1	30(2)	20(2)	39(3)	-4(2)	-4(2)	-0.9(19)
C10	24(2)	35(3)	36(3)	5(2)	-2.2(19)	-5.4(19)
C2	21(2)	23(3)	38(2)	-1.4(18)	0.8(19)	2.8(16)
C8	32(2)	24(2)	26(2)	0.9(18)	-5.9(18)	-5.4(19)
C7	25(2)	29(2)	27(2)	-3.6(18)	-3.4(18)	-4.8(18)
C9	30(2)	29(3)	24(2)	-2.7(19)	-8.5(18)	1(2)

DFT calculations

1. Computational methods

All density functional theory (DFT) calculations were conducted with Gaussian 16.[31] Geometry optimizations of all intermediates and transition states were performed at the B3LYP[32] level of theory with a def2-SVP[33] basis set including Grimme's D3 dispersion corrections.[34] Based on the optimized structures, vibrational frequencies were calculated at the same level of theory to evaluate zero-point vibrational energy (ZPVE) and thermal corrections at 298 K. The single-point energies were computed with a M06[35] functional and def2-TZVPP[33,36] basis set, including solvation energy corrections. The solvation energies were evaluated by a self-consistent reaction field (SCRF) using SMD model.[37] Extensive conformational searches for intermediates and transition states were conducted to ensure that the lowest energy conformers were located. Intrinsic reaction coordinate (IRC) calculations of the transition states were performed to verify their locations in the free energy surface. The Hirshfeld charges of intermediates were analyzed at the B3LYP-D3/def2-SVP level of theory based on the optimized structures.

Independent Gradient Model (IGM)[38] analysis was performed with Multiwfn[39] software package, using high quality grid option to generate files for further plotting, and the visualization of IGM analysis results are presented with VMD[40] visualization software. The 3D diagrams of molecules were generated using CYLview.[41]

As it is known that in solution energies for association/dissociation processes are overestimated/underestimated, in order to adjust the Gibbs free energies from 1 atm to 1 mol/L, a correction of $RT\ln(c_s/c_g)$ is added to the energies of all species.[42] c_s is the standard molar concentration in solution (1 mol/L), c_g is the standard molar concentration in the gas phase (0.0446 mol/L), and R is the gas constant.

2. Details of distortion-interaction analysis

We performed the distortion-interaction analysis[43], [44] on transition states of Ir/(*R*,*R*)-**3**-mediated cyclopropanation (**TS1–TS4**), in order to reveal the origins that are responsible for the selectivity. Here, we use **TS1** as an example to shows the protocol of distortion-interaction analysis. The optimized structure of **TS1** is separated into two fragments, the Ir/(*R*,*R*)-**3** catalyst fragment and substrate fragment. The energies of these distorted fragments were computed at the M06 level of theory with def2-TZVPP basis set, without the inclusion of solvation energy corrections. Comparing the energies of the distorted fragments in the transition state (**TS1**) and the reference points (separately optimized catalyst and substrate fragments), we obtained the distortion energies of the catalyst and substrate ($\Delta E_{\text{dist(cat)}}$ and $\Delta E_{\text{dist(sub)}}$). The interaction energy, ΔE_{int}, is the difference between the total distortion energy and the electronic reaction barrier, $\Delta E_{\text{int}} = \Delta E^{\ddagger} - (\Delta E_{\text{dist-cat}} + \Delta E_{\text{dist-sub}})$.

The origins for the distortion of substrate were examined through IGM analysis.[37] As shown in Figure S3, a favorable C–H•••π interaction (purple dashed circle) exists in both **TS1** and **TS2**. However, maintaining this interaction in **TS2**, brings two C–H bonds in close alignment and the distance between the two hydrogen atoms is reduced to 2.25 Å, which in turn produces significant steric hindrance (red dashed circle). Accordingly, large distortions in the substrate are generated. A similar situation is found **TS3** and **TS4** (Figure S4): both **TS3** and **TS4** have favorable C–H•••π interaction (purple dashed circle), but closed shell repulsion (red dashed circle) between C–H bonds causes a great distortion in the substrate.

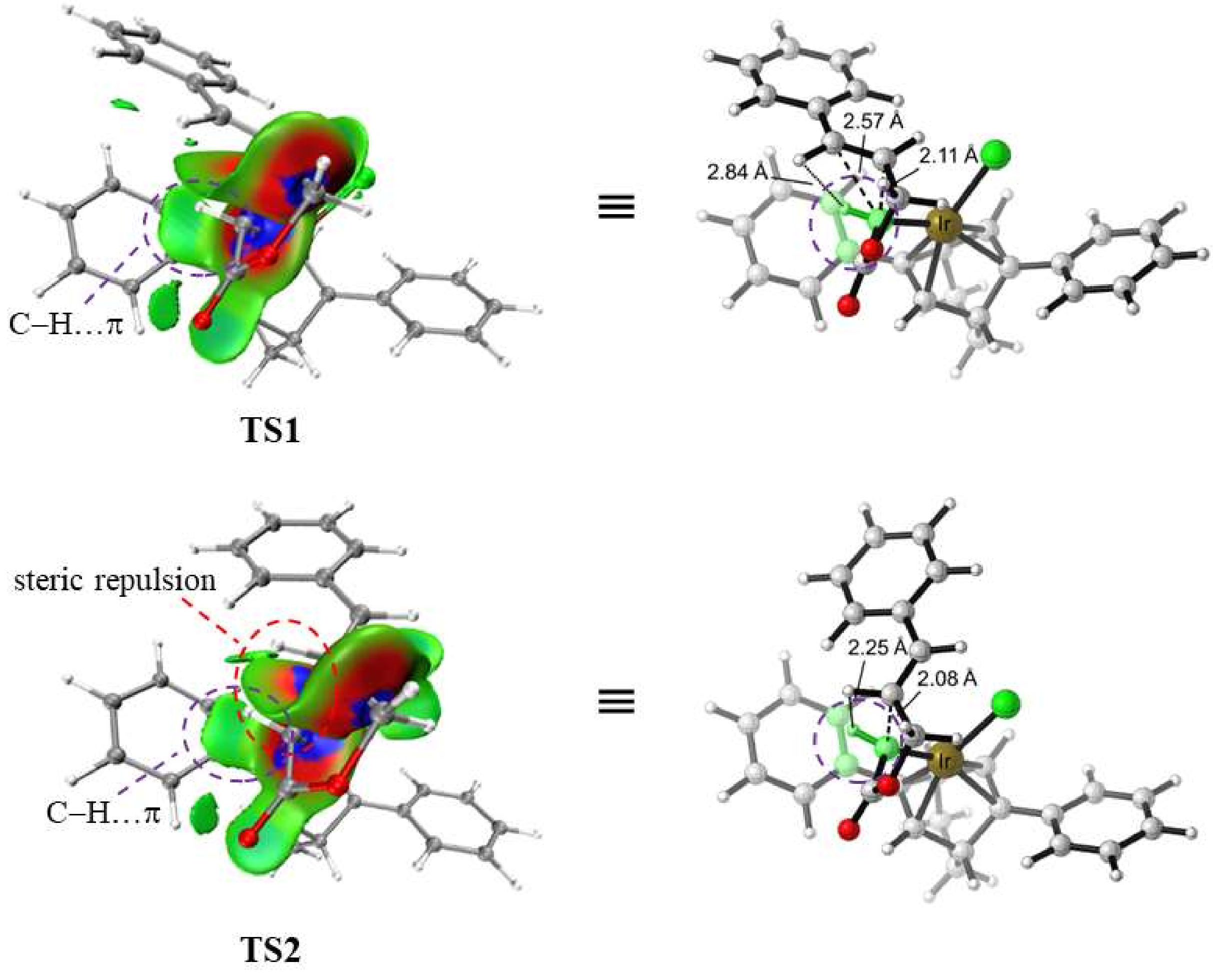

Figure S3. NCI isosurfaces of transition states **TS1** and **TS2** (blue: strong attraction; green: weak interaction; red: steric effect).

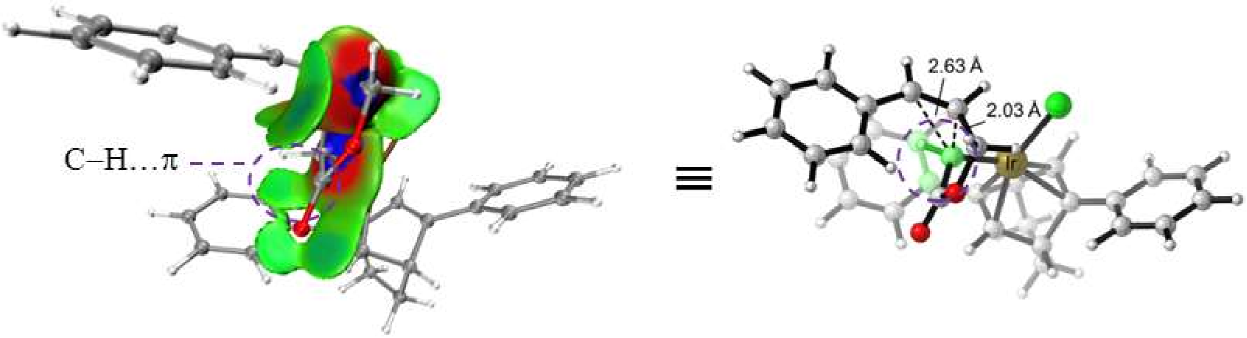

TS3

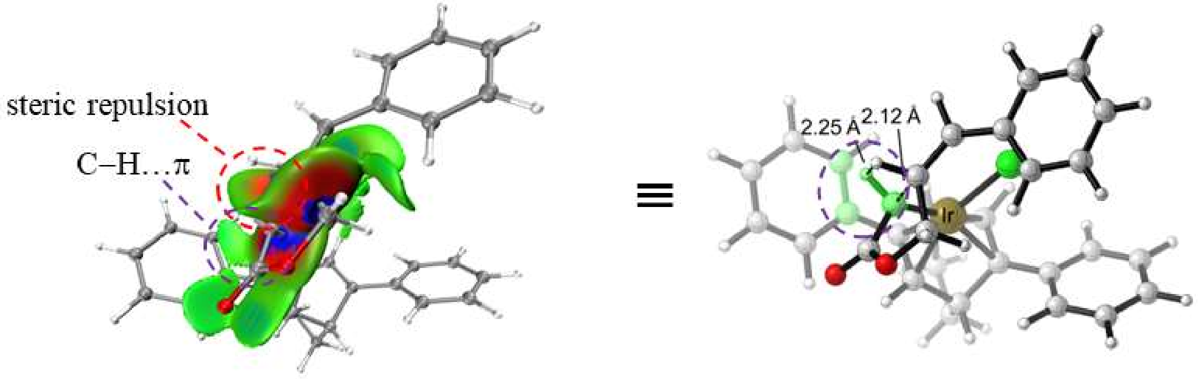

TS4

Figure S4. NCI isosurfaces of transition states **TS3** and **TS4** (blue: strong attraction; green: weak interaction; red: steric effect).

3. Free energy diagrams of product formation

Figures S5 and S6 are the free energy diagrams for major and minor enantiomer formation based on *E* olefin (**1a**), and Figures S7 and S8 are the free energy diagrams for major and minor enantiomer formation based on *Z* olefin (**1f**). As shown in Figure S5, based on **TS1**, the reaction follows a concerted mechanism, directly forming major enantiomer **(-)-2a**. If the reaction occurs via **TS2** (Figure S6), it follows a stepwise mechanism, first forming intermediate **III**, and then after second C−C bond formation (via **TS5**), the minor enantiomer (**(+)-2a**) is generated. Since **TS5** is lower in energy than **TS2**, the competition between **TS1** and **TS2** determines the stereoselectivity. Similar situation also applies to *Z* olefin, and the competition between **TS3** and **TS4** determines the enantioselectivity (Figures S7 and S8).

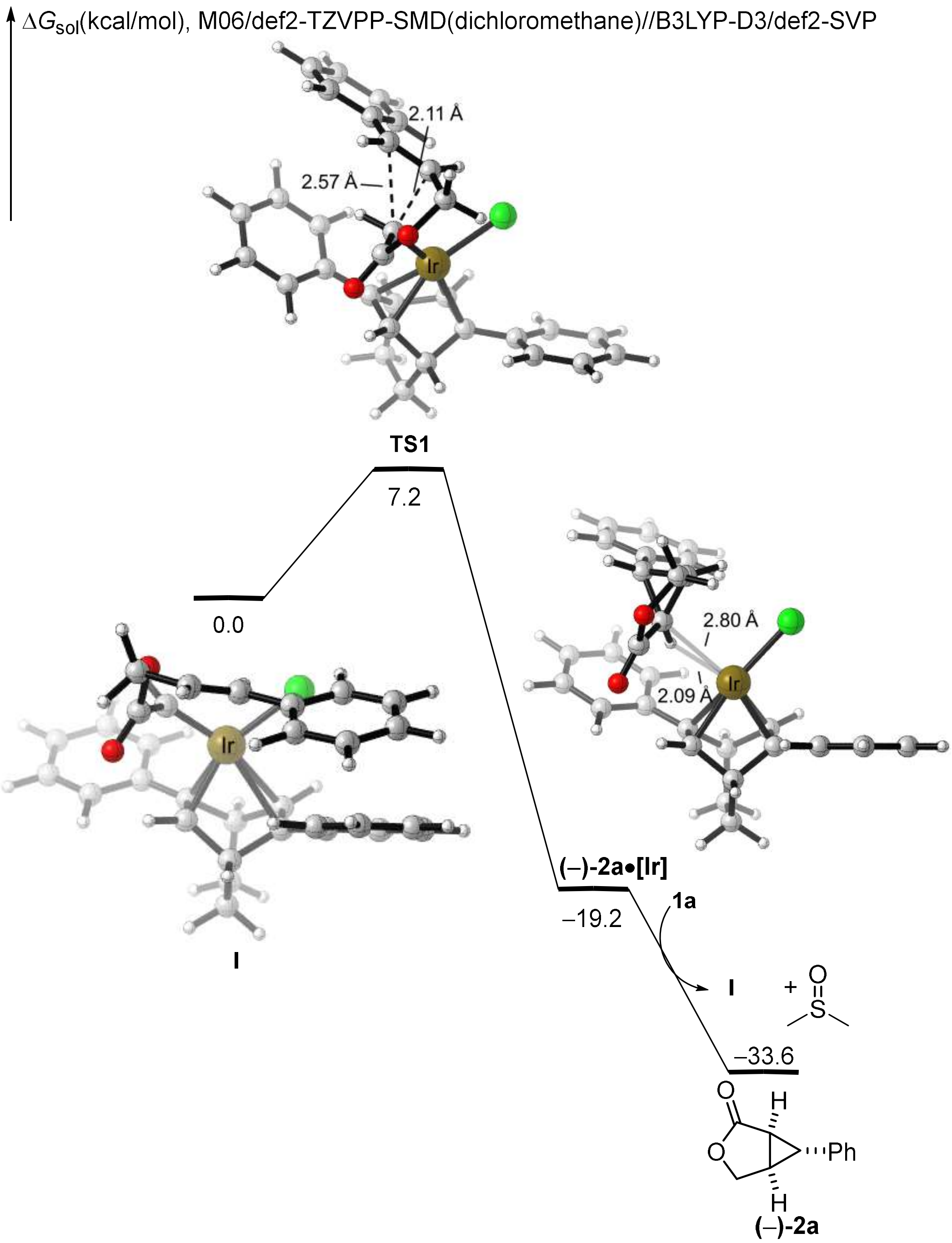

Figure S5. DFT-computed free energy changes of Ir/(*R*,*R*)-**3**-catalyzed cyclopropanation to form (–)-**2a**.

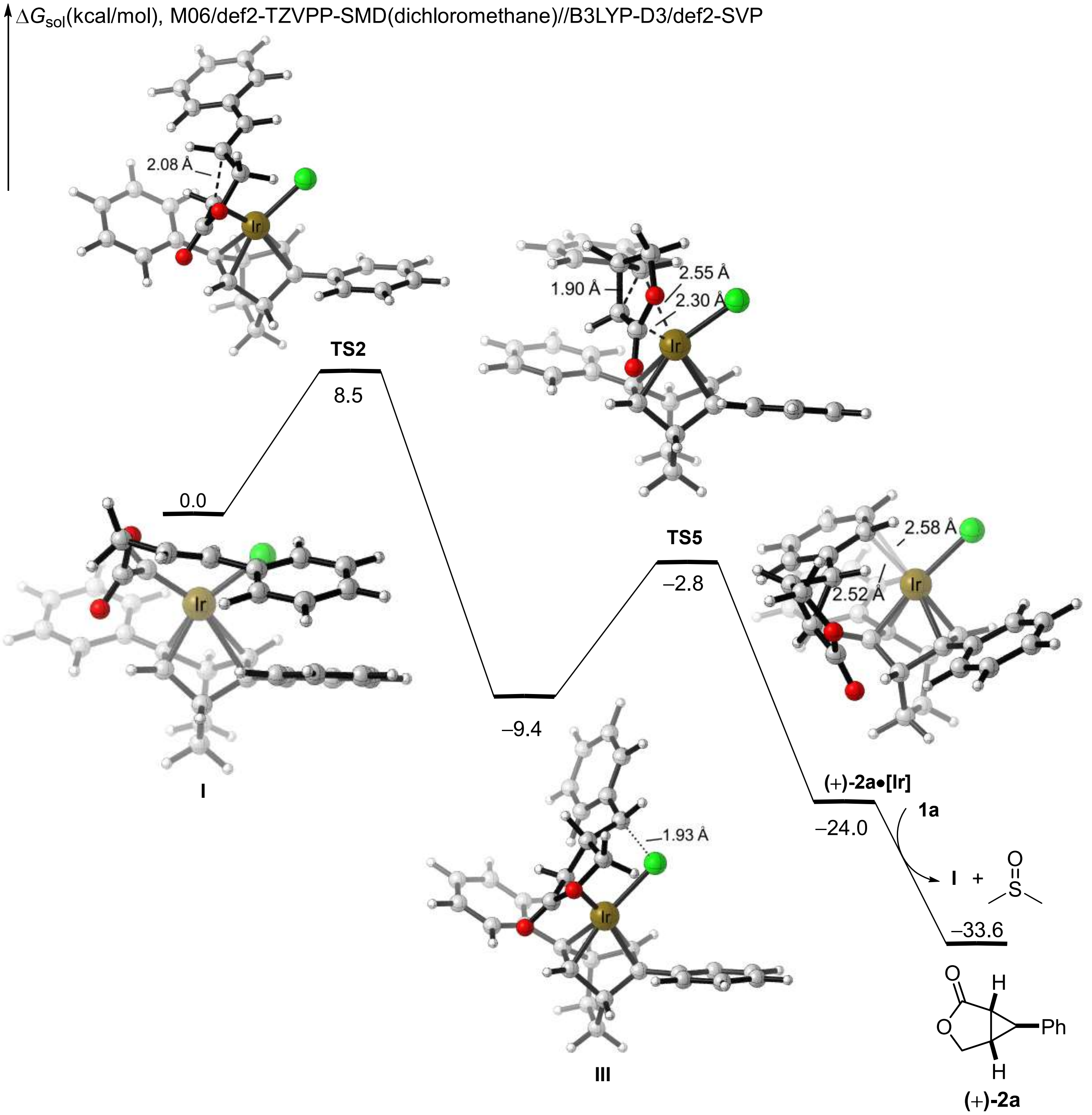

Figure S6. DFT-computed free energy changes of Ir/(*R*,*R*)-**3**-catalyzed cyclopropanation to form (+)-**2a**.

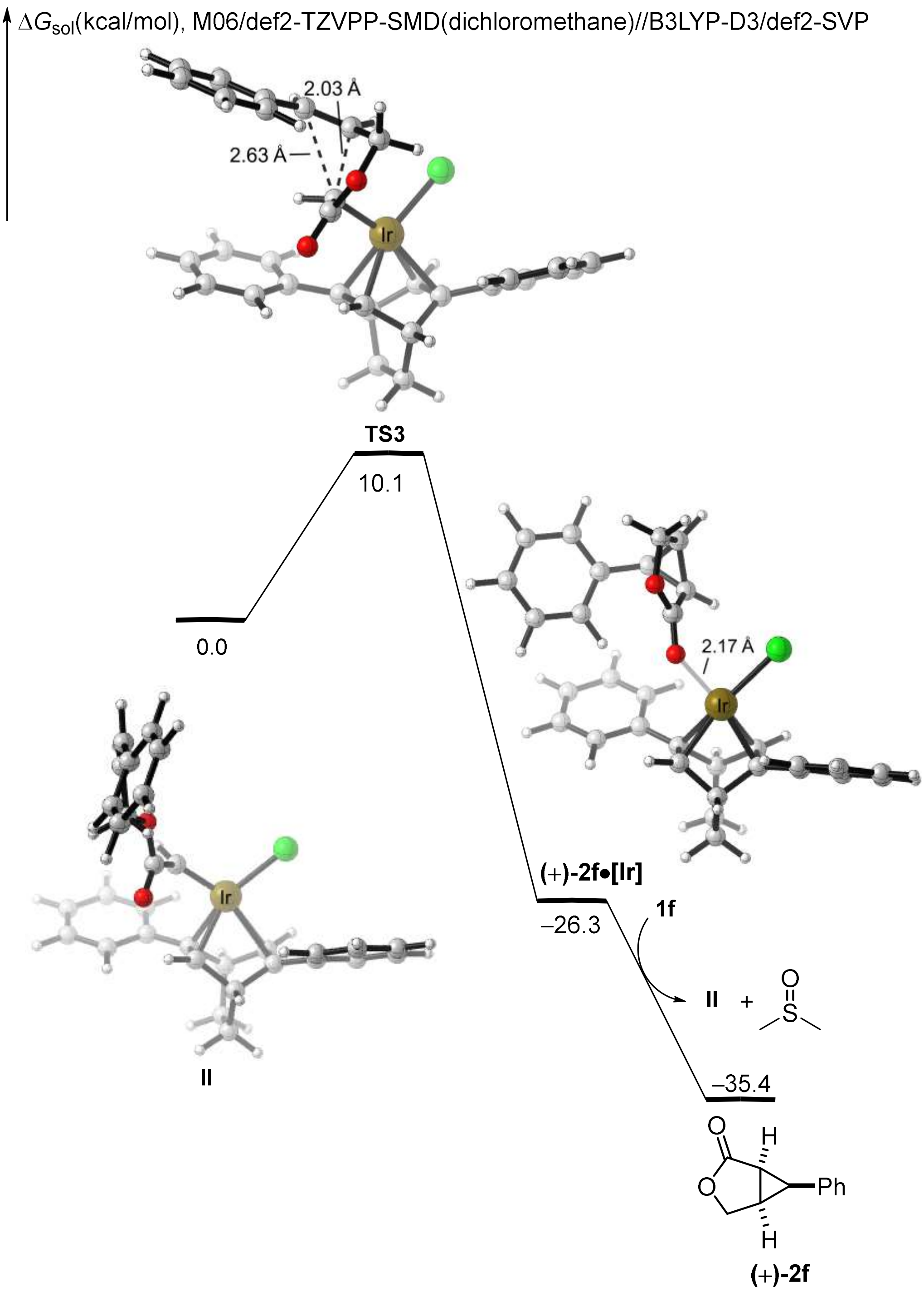

Figure S7. DFT-computed free energy changes of Ir/(*R*,*R*)-**3**-catalyzed cyclopropanation to form (+)-**2f**.

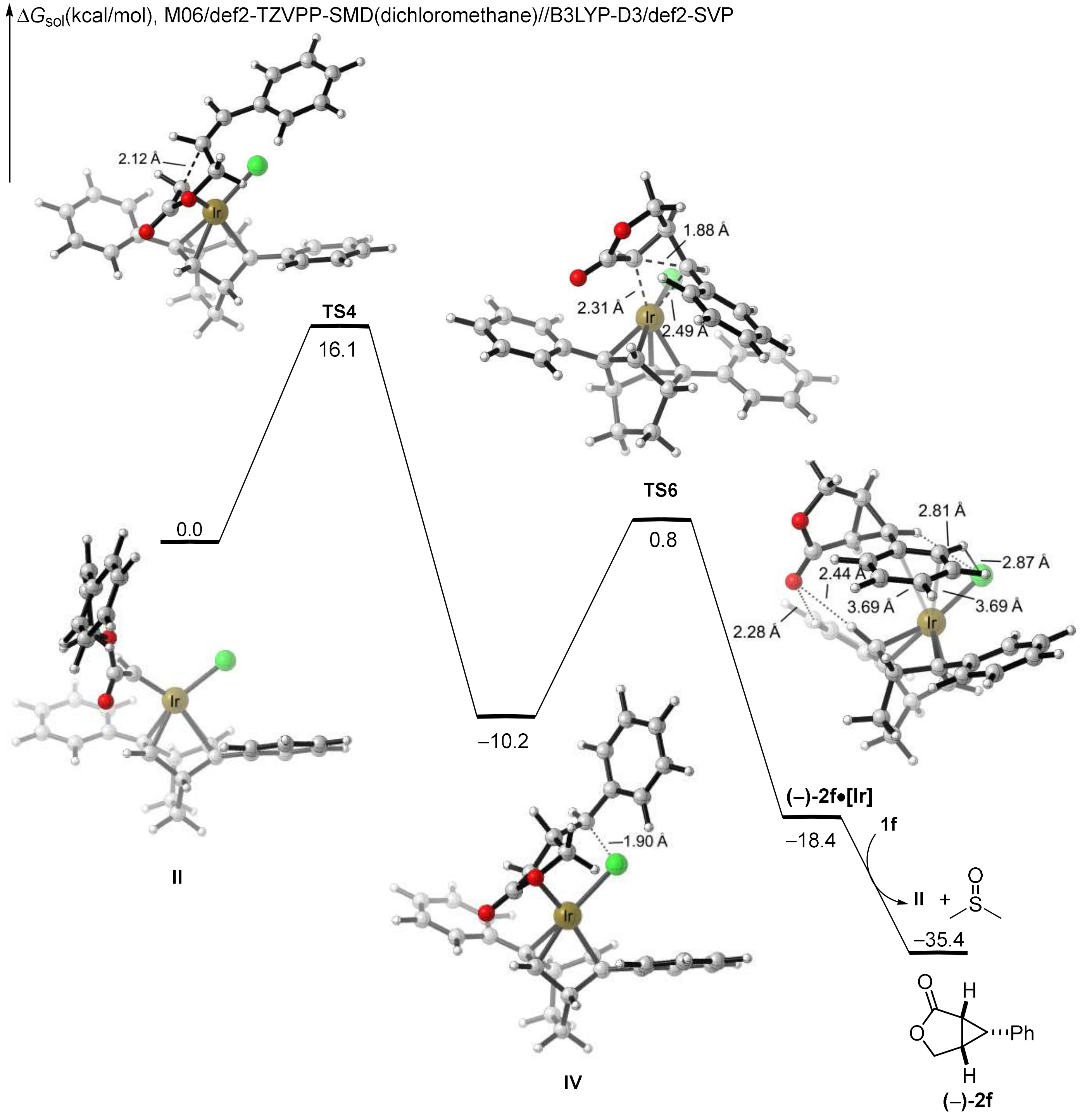

Figure S8. DFT-computed free energy changes of Ir/(*R*,*R*)-**3**-catalyzed cyclopropanation to form (–)-**2f**.

For the minor enantiomer ((+)-**2a** or (-)-**2f**) formation, the reaction undergoes a two-step mechanism (Figures S2 and S4). After the formation of the first carbon-carbon bond, the benzylic carbon has a positive charge (0.097 e), which is stabilized through an electrostatic interaction between benzylic carbon and chloride ligand. As shown in Figures S2 and S4, in **III** and **IV**, there is, and the C−Cl bond distances are 1.93 and 1.90 Å, respectively. Hirshfeld charges in **III** and **IV** were calculated (Table S8), which indicated that a partial positive charge is mostly delocalised between the benzylic carbon atom, the iridium atom (0.239 e (**III**) and 0.232 e (**IV**)), and the benzene ring (0.025 e (**III**), 0.039 e (**IV**)).

Table S9. Hirshfeld charges (in e) of the intermediates calculated at the B3LYP-D3/def2-SVP level of theory based on the optimized structures (atom 8 is benzylic carbon).

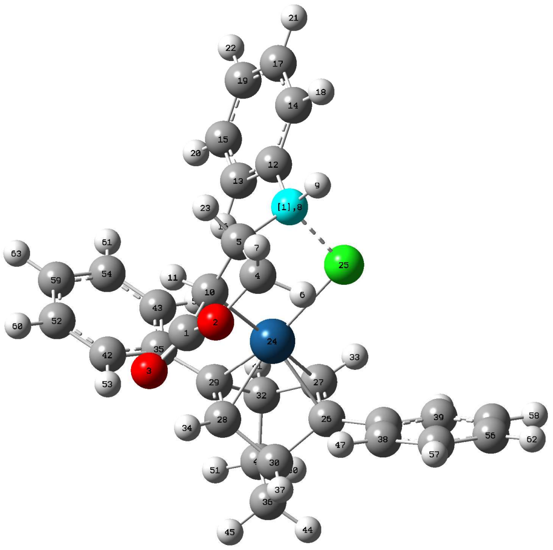

III

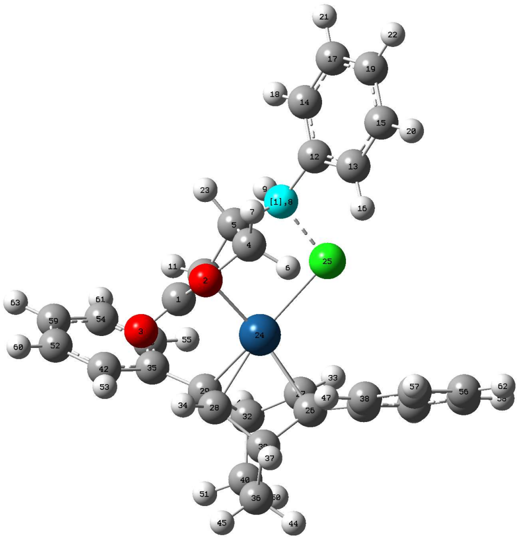

IV

Structure	Atom label	Element name	Charge	Structure	Atom label	Element name	Charge
	1	C	0.244687		1	C	0.243972
	2	O	-0.225027		2	O	-0.22684
	3	O	-0.336143		3	O	-0.335623
	4	C	0.136746		4	C	0.13104
	5	C	0.003673		5	C	0.003118
	8	C	0.097025		8	C	0.097394
	10	C	-0.125752		10	C	-0.124086
	12	C	-0.02099		12	C	-0.018478
	13	C	0.010398		13	C	0.008558
III	14	C	0.005622	IV	14	C	0.009566
	15	C	0.009638		15	C	0.01425
	17	C	0.006843		17	C	0.01195
	19	C	0.013248		19	C	0.013596
	24	Ir	0.239233		24	Ir	0.231668
	25	Cl	0.011088		25	Cl	0.027408
	26	C	-0.03831		26	C	-0.040086
	27	C	-0.042281		27	C	-0.045833
	28	C	-0.029007		28	C	-0.026578
	29	C	-0.033542		29	C	-0.032307

30	C	0.018611	30	C	0.017443
31	C	-0.010108	31	C	-0.010142
32	C	0.01274	32	C	0.012877
35	C	-0.006698	35	C	-0.007585
36	C	0.016377	36	C	0.015832
38	C	0.008087	38	C	0.006176
39	C	-0.001948	39	C	-0.001919
40	C	0.013635	40	C	0.013691
42	C	0.020846	42	C	0.020506
43	C	-0.000772	43	C	-0.002594
46	C	0.002392	46	C	0.000184
48	C	-0.000239	48	C	-0.000021
52	C	0.008666	52	C	0.006383
54	C	-0.00608	54	C	-0.007937
56	C	-0.001303	56	C	-0.00251
59	C	-0.001419	59	C	-0.003115

4. Tables of energies

Table S10. Energies in Scheme 3 of manuscript, and Figures S1–S4 of supporting information. Zero-point vibrational energy (*ZPVE*), thermal correction to enthalpy (*TCH*), thermal correction to Gibbs free energy (*TCG*), energies (*E*), enthalpies (*H*), and Gibbs free energies (*G*) (in Hartree) of the structures calculated at the M06/def2-TZVPP-SMD(dichloromethane)//B3LYP-D3/def2-SVP level of theory**.**

Structures	*ZPVE*	*TCH*	*TCG*	*E*	*H*	*G*	Imaginary Frequency
**TS1**	0.507178	0.538319	0.443432	-1912.707695	-1912.169376	-1912.264263	165.5*i*
**TS2**	0.506721	0.537879	0.441967	-1912.704006	-1912.166127	-1912.262039	264.6*i*
**TS3**	0.507596	0.538567	0.443305	-1912.701946	-1912.163379	-1912.258641	271.7*i*
**TS4**	0.506630	0.537637	0.442374	-1912.691424	-1912.153787	-1912.249050	278.3*i*
**I**	0.506780	0.539013	0.441164	-1912.716823	-1913.255836	-1912.275659	
**(–)-2a·[Ir]**	0.509510	0.540690	0.445035	-1912.751313	-1912.210623	-1912.306278	
**1a**	0.264046	0.282890	0.214859	-1128.599444	-1128.316554	-1128.384585	
**DMSO**	0.078461	0.085126	0.050076	-553.176287	-553.091161	-553.126211	
**(–)-2a/(+)-2a**	0.184860	0.195873	0.147037	-575.461965	-575.266092	-575.314928	
**III**	0.510666	0.540987	0.448219	-1912.738933	-1912.197946	-1912.290714	
**TS5**	0.509246	0.539504	0.447937	-1912.728037	-1912.188533	-1912.280100	169.5*i*
**(+)-2a·[Ir]**	0.510448	0.541265	0.447790	-1912.761633	-1912.220368	-1912.313843	
**II**	0.506204	0.538839	0.435304	-1912.710083	-1912.171244	-1912.274779	

**(+)-2f·[Ir]**	0.510362	0.540975	0.446866	-1912.763603	-1912.222628	-1912.316737	
**1f**	0.264309	0.282922	0.215870	-1128.596219	-1128.313297	-1128.380349	
**(+)-2f/(–)-2f**	0.185157	0.195961	0.148817	-575.462402	-575.266441	-575.313585	
**IV**	0.510540	0.540907	0.446779	-1912.737789	-1912.196882	-1912.291010	
**TS6**	0.508536	0.538844	0.447128	-1912.720692	-1912.181848	-1912.273564	272.5*i*
**(–)-2f·[Ir]**	0.510717	0.541833	0.446294	-1912.750466	-1912.208633	-1912.304172	

Table S11. Energies in Table 2 of manuscript. Energies (*E*) (in Hartree) of the structures calculated at the M06/def2-TZVPP//B3LYP-D3/def2-SVP level of theory.

Structures	*E*
**((*R*,*R*)-3)IrCl**	-1337.233269
**Carbene 1**	-575.302853
**Catalyst fragment in TS1**	-1337.212291
**Substrate fragment in TS1**	-575.320239
**TS1**	-1912.664691
**Catalyst fragment in TS2**	-1337.211198
**Substrate fragment in TS2**	-575.301209
**TS2**	-1912.660182
**Carbene 2**	-575.299782
**Catalyst fragment in TS3**	-1337.212526
**Substrate fragment in TS3**	-575.31914
**TS3**	-1912.657323
**Catalyst fragment in TS4**	-1337.212216
**Substrate fragment in TS4**	-575.293723
**TS4**	-1912.647162

5. Cartesian coordinates of the DFT-optimized structures

TS1

C	0.99286300	-0.61865400	1.12006900
H	1.91748200	-0.11843800	0.80392300
C	0.93067300	-0.71135500	2.61718300
O	0.92098200	-1.95441800	3.14196100
O	0.88874400	0.26272100	3.32948200
C	0.83394900	-3.02251300	2.18813100
C	1.51580800	-2.65258500	0.89369000

H	-0.22701600	-3.23995000	1.98740000
H	1.29182700	-3.89903800	2.67126900
C	2.85474800	-2.35762500	0.80691300
H	0.93941100	-2.86266800	-0.01116400
H	3.44396900	-2.32227500	1.73189500
C	3.53356600	-1.92670000	-0.40396800
C	2.83888900	-1.72575600	-1.62164400
C	4.91187000	-1.62330100	-0.35475000
C	3.51395300	-1.24937800	-2.74295800
H	1.76532200	-1.91767700	-1.68375200
C	5.58166900	-1.15124100	-1.48193700
H	5.45468500	-1.75638400	0.58511600
C	4.88396700	-0.96262000	-2.68048900
H	2.96443500	-1.09283100	-3.67446300
H	6.64871400	-0.92260200	-1.42603600
H	5.40589400	-0.58744400	-3.56454300
Ir	-0.56563400	-0.13999800	0.01733400
Cl	-1.05167000	-2.16234900	-1.09986200
C	-2.59494400	0.87115700	-0.36697100
C	-1.73171100	1.13258600	-1.43574500
C	-0.90479500	1.67878300	1.10478700
C	-0.03992000	1.96513800	0.00491500
C	-2.38112900	1.87221700	0.77877200
C	-3.77135600	-0.02760700	-0.41805400
C	-0.84211200	2.35361300	-1.24552500
H	-1.84200100	0.65126600	-2.40970000
H	-0.55496500	1.69319000	2.13897700
C	1.40491400	2.29738900	0.12326600
C	-2.63899100	3.31164300	0.26058900
H	-3.01629200	1.64140300	1.64164300
C	-4.24018800	-0.65339500	0.75187800
C	-4.43396200	-0.29244000	-1.62871800
C	-1.70192800	3.60793900	-0.93964400
H	-0.18971600	2.51587100	-2.11166200
C	1.94974900	2.80267600	1.31527400
C	2.27776300	2.01683700	-0.94607000
H	-3.69806600	3.40543500	-0.02721800

H	-2.46431900	4.02109800	1.08470200
C	-5.33413800	-1.51730900	0.71170200
H	-3.71806900	-0.48567600	1.69635200
C	-5.53017900	-1.15638300	-1.66984200
H	-4.09451000	0.19230400	-2.54617900
H	-2.27948600	3.87552000	-1.83845700
H	-1.03066700	4.45405400	-0.72345200
C	3.32563900	3.01925800	1.43438600
H	1.29562000	3.00921400	2.16309800
C	3.65134300	2.22450100	-0.82497200
H	1.87849400	1.57656400	-1.86228500
C	-5.98505100	-1.77237800	-0.50043600
H	-5.67634800	-2.00162000	1.62988700
H	-6.03388600	-1.34689300	-2.62106400
C	4.18170200	2.72965400	0.36807400
H	3.73019300	3.40927800	2.37188000
H	4.31195100	1.96881800	-1.65675900
H	-6.84266700	-2.44914800	-0.53269000
H	5.25847000	2.88904100	0.46723600

TS2

C	0.72975800	0.26548300	2.85559100
O	0.61543200	-0.79710900	3.68150300
O	0.56031900	1.38873600	3.26677400
C	0.92180200	-2.06137300	3.08221700
C	1.91990800	-1.85251800	1.97533800
H	0.00188200	-2.51468100	2.67544000
H	1.31122100	-2.69384700	3.89576200
C	2.01178700	-2.71460400	0.89892800
H	1.30407900	-3.54209200	0.83323500
C	1.04903400	-0.05115700	1.42204400
H	1.98259600	0.41987600	1.08613300
C	2.93163700	-2.58099000	-0.21364100
C	3.84467800	-1.50734400	-0.33105700
C	2.87179400	-3.52478900	-1.26326500
C	4.67025600	-1.39529200	-1.44621500
H	3.90173900	-0.74449600	0.44669400
C	3.70261100	-3.41368900	-2.37572100

H	2.14386500	-4.33750300	-1.20335300
C	4.60596500	-2.34858100	-2.47094000
H	5.36351600	-0.55416500	-1.52133100
H	3.64028800	-4.15270300	-3.17813700
H	5.25396400	-2.25597400	-3.34617200
H	2.82433100	-1.30540300	2.25988900
Ir	-0.42606000	0.00281400	0.12972000
Cl	-0.66321500	-2.28349800	-0.28263100
C	-2.48597000	0.68913700	-0.69301900
C	-1.55198300	0.69042300	-1.72825100
C	-0.97667900	2.02198600	0.58619900
C	-0.03471900	2.04055700	-0.48771800
C	-2.42659000	1.99251100	0.11761700
C	-3.59421600	-0.28207200	-0.54208100
C	-0.74643100	1.97757200	-1.84836100
H	-1.54688300	-0.06382500	-2.51787700
H	-0.72595600	2.36562900	1.59165400
C	1.37134700	2.51229000	-0.37714000
C	-2.71660900	3.19347100	-0.82114800
H	-3.12153700	1.97359500	0.96506200
C	-4.09708700	-0.59569600	0.73427800
C	-4.15720100	-0.92337600	-1.65866500
C	-1.69621700	3.19616800	-1.98966800
H	-0.03342200	1.92845500	-2.67952800
C	1.79160600	3.33599000	0.68141200
C	2.33740800	2.06774500	-1.30031300
H	-3.75026900	3.11732900	-1.19409400
H	-2.65213300	4.12420900	-0.23584500
C	-5.12761300	-1.52242500	0.88854100
H	-3.64790500	-0.13247600	1.61548000
C	-5.18989900	-1.85105500	-1.50512900
H	-3.79071200	-0.68299200	-2.65879400
H	-2.20737500	3.14256900	-2.96381200
H	-1.09324200	4.11801100	-1.99158400
C	3.13362300	3.70367400	0.81132900
H	1.06682500	3.67616000	1.42162600
C	3.67711400	2.43600000	-1.17189300

H	2.04023500	1.38913100	-2.10261500
C	-5.67957400	-2.15434500	-0.23152600
H	-5.49705400	-1.76038700	1.88936700
H	-5.61735700	-2.33637300	-2.38645200
C	4.08253400	3.25808000	-0.11381300
H	3.43863300	4.34200800	1.64453200
H	4.40969200	2.07210400	-1.89730100
H	-6.48775600	-2.88029000	-0.11114300
H	5.13154600	3.54723200	-0.01033000

TS3

C	1.17320400	-0.30737500	-0.04594400
H	1.84241800	0.48611400	-0.40277600
C	1.54672300	-0.78287200	1.32851100
O	1.62450900	-2.12759300	1.46755300
O	1.71987200	-0.05432700	2.27517100
C	1.46362200	-2.85143600	0.23971400
C	1.86959000	-1.99717000	-0.93899900
H	0.40619600	-3.13096600	0.11886600
H	2.07653500	-3.76197700	0.32883400
C	3.14616600	-1.55267300	-1.24921900
H	1.16150100	-2.03447200	-1.77235600
H	3.20658300	-1.04441600	-2.21843600
C	4.40308800	-1.55953000	-0.52670300
C	4.61008700	-2.12709400	0.75612400
C	5.51659500	-0.96200900	-1.17177800
C	5.87092100	-2.09770000	1.34771600
H	3.78313900	-2.56393200	1.31060600
C	6.77355800	-0.93779700	-0.57638000
H	5.37810500	-0.51381500	-2.15930700
C	6.95629100	-1.50847200	0.68884600
H	6.00658400	-2.53422800	2.34007600
H	7.61404500	-0.47150800	-1.09597800
H	7.94098300	-1.48960700	1.16254000
Ir	-0.76481600	-0.01774000	-0.37191300
Cl	-1.26065800	-1.79028700	-1.84160400
C	-2.93935500	0.37805300	0.26401000
C	-2.62431400	1.16201100	-0.85034400

C	-1.00561800	1.25487000	1.33470500
C	-0.70308800	2.07868600	0.20751800
C	-2.49894200	1.06005700	1.56635600
C	-3.84533000	-0.79385100	0.25848900
C	-1.98974000	2.50592100	-0.51649100
H	-3.00969900	0.94402500	-1.84861000
H	-0.30203600	1.10779100	2.15610100
C	0.57194500	2.81978400	0.01644100
C	-3.21196900	2.43105100	1.70072500
H	-2.68943300	0.42590400	2.43990200
C	-3.68257600	-1.82289100	1.20423800
C	-4.87137600	-0.91693200	-0.69340600
C	-2.89360600	3.30435400	0.45914300
H	-1.77883700	3.08250800	-1.42408200
C	1.48350600	3.00158800	1.07237500
C	0.92505400	3.30876200	-1.25605600
H	-4.29578900	2.26398900	1.80455600
H	-2.87013900	2.91855400	2.62730300
C	-4.51804200	-2.93920500	1.19709000
H	-2.86817600	-1.76036600	1.92933400
C	-5.70959200	-2.03390600	-0.70074000
H	-5.02169400	-0.12416200	-1.42892900
H	-3.81568900	3.59976000	-0.06590900
H	-2.37361700	4.23216100	0.74561900
C	2.70199500	3.65121000	0.86203700
H	1.25210800	2.60952500	2.06225900
C	2.14111200	3.96076700	-1.46587800
H	0.25074200	3.14947700	-2.10004300
C	-5.53681800	-3.04941700	0.24397300
H	-4.36857300	-3.73306900	1.93346600
H	-6.50417700	-2.10964000	-1.44758400
C	3.03675800	4.13694800	-0.40549600
H	3.39540900	3.77570500	1.69774800
H	2.39345300	4.32803100	-2.46421800
H	-6.19212800	-3.92415100	0.23769900
H	3.99008300	4.64657000	-0.56734500

TS4

C	0.77648400	-1.98169600	1.97263400
O	-0.29962900	-2.19480100	2.76017100
O	1.88641400	-1.95169900	2.44650100
C	-1.54938500	-2.20009200	2.07126300
C	-1.35026700	-2.77914400	0.69844400
H	-1.92506500	-1.16597100	1.99236000
H	-2.24113500	-2.78908400	2.69562300
C	-2.25165500	-2.71857100	-0.35447400
H	-1.91879000	-3.23603900	-1.26007000
C	0.49891400	-1.75081200	0.51463300
H	1.04079200	-2.46911600	-0.11947600
C	-3.63921500	-2.27374200	-0.41977300
C	-4.36738200	-1.73080500	0.66273600
C	-4.31914600	-2.44395000	-1.64845500
C	-5.70425600	-1.36601000	0.51307700
H	-3.89594600	-1.58220100	1.63098100
C	-5.65383700	-2.07968900	-1.79588900
H	-3.77389900	-2.85594000	-2.50146900
C	-6.35489700	-1.53485100	-0.71362600
H	-6.24437900	-0.94401300	1.36421500
H	-6.15144100	-2.21687600	-2.75909100
H	-7.40266700	-1.24526900	-0.82524200
H	-0.70144700	-3.66199100	0.66355800
Ir	0.42587400	0.08866700	-0.16348800
Cl	-1.65582800	-0.03557600	-1.18136800
C	0.61401700	2.38789700	-0.04997800
C	1.08895000	1.94910700	-1.28710100
C	2.07146000	0.83518500	1.01408800
C	2.57010100	0.39700400	-0.24861600
C	1.69829700	2.31212400	1.03421900
C	-0.66466600	3.10518000	0.16720000
C	2.55464500	1.53933200	-1.27976300
H	0.53720200	2.09433700	-2.21787300
H	2.30137400	0.30414100	1.93974300
C	3.45046300	-0.78340700	-0.45879600
C	2.90930100	3.18533100	0.61176000

H	1.31176100	2.61702800	2.01372000
C	-1.34327400	2.99253000	1.39491400
C	-1.23947400	3.89271800	-0.84460700
C	3.44167100	2.70219400	-0.76282000
H	2.88176300	1.19378800	-2.26739300
C	4.28610900	-1.27025600	0.55866400
C	3.44605400	-1.44916900	-1.69928300
H	2.59472500	4.24020200	0.56975900
H	3.68776000	3.11129200	1.38732100
C	-2.55893600	3.64349100	1.60233700
H	-0.92659000	2.35754400	2.17980100
C	-2.45625500	4.54607500	-0.63736100
H	-0.72244500	4.00305000	-1.79992000
H	3.43269400	3.51840200	-1.50225700
H	4.48134000	2.34648900	-0.68603400
C	5.09562000	-2.38831700	0.34198300
H	4.29374700	-0.77817500	1.53124600
C	4.25189900	-2.56703100	-1.91592900
H	2.77636300	-1.10005300	-2.48921100
C	-3.12123600	4.42418300	0.58610800
H	-3.07565800	3.53549400	2.55955000
H	-2.88562400	5.15684500	-1.43577300
C	5.08328900	-3.04063800	-0.89377000
H	5.73673000	-2.75373300	1.14822000
H	4.22867600	-3.07464000	-2.88390700
H	-4.07385000	4.93503700	0.74785700
H	5.71608900	-3.91617400	-1.06063300

Intermediate I

O	-0.93034000	2.67028100	1.65133500
C	-0.71488700	2.70068600	0.46073500
O	0.14400100	3.53374100	-0.14866300
H	-2.19668100	2.18634000	-1.10590100
C	1.09615700	4.23710900	0.66304700
C	2.40905600	3.50970400	0.72900700
H	0.67973500	4.39219800	1.67190600
H	1.22029000	5.22630000	0.19094700
C	2.70126600	2.39342600	0.04211100

H	3.15650700	3.98760100	1.37315200
H	1.93999600	1.97577500	-0.62395500
C	3.98448000	1.67049000	0.04938700
C	4.27270700	0.79689800	-1.01722200
C	4.94521300	1.81324300	1.07091200
C	5.49297400	0.12211600	-1.08177600
H	3.51810200	0.63860600	-1.79062300
C	6.16011900	1.12922500	1.01153800
H	4.73226000	2.45422200	1.92993600
C	6.44398600	0.28629400	-0.07074500
H	5.69314400	-0.55073800	-1.91933400
H	6.89006300	1.25046400	1.81646900
H	7.39667900	-0.24777700	-0.11613900
Cl	0.82309200	-0.11917200	-2.24547200
C	-1.05186000	-2.42166500	-0.50400000
C	-2.59656700	-0.77542900	0.35025500
C	-0.78616400	-1.67201500	1.77025400
C	1.32609000	-2.41964300	0.39696700
C	-2.50046400	-2.25067300	-0.07180500
H	-0.78367400	-2.85029600	-1.47125400
H	-1.70092500	0.39272800	2.00926900
C	-3.86638600	-0.02185600	0.16348600
C	-1.74032300	-2.78849200	2.28260500
H	-0.04492000	-1.40395800	2.53118300
C	1.82135300	-3.50738200	-0.34242500
C	2.24161400	-1.57665400	1.05435400
C	-2.77649800	-3.12931400	1.18041200
H	-3.19482700	-2.48313900	-0.88740500
C	-4.40632500	0.76619800	1.19414700
C	-4.53971200	-0.06299100	-1.07147700
H	-1.14405100	-3.67194300	2.55877900
H	-2.23654700	-2.43382500	3.19942700
C	3.19474100	-3.74996300	-0.41516500
H	1.12317700	-4.17704100	-0.84929000
C	3.61210100	-1.81422600	0.97564800
H	1.87842700	-0.69638900	1.58883600
H	-2.71932200	-4.19077600	0.89398100

H	-3.80513200	-2.94042400	1.52553500
C	-5.58249000	1.49427700	0.99469700
H	-3.90202200	0.81103900	2.16106500
C	-5.71472600	0.66239600	-1.27049500
H	-4.11630900	-0.64578500	-1.89288100
C	4.09266900	-2.90509000	0.24369900
H	3.56446700	-4.60371800	-0.98891200
H	4.31026100	-1.12796000	1.45885300
C	-6.24197400	1.44512000	-0.23660900
H	-5.98676800	2.10079200	1.80921900
H	-6.21867400	0.62311300	-2.23963300
H	5.16840400	-3.08513700	0.17773900
H	-7.16192000	2.01435400	-0.39171700
C	-1.60943900	-0.46592700	1.34062700
C	-0.12427000	-2.15134600	0.47670100
C	-1.34993200	1.77944300	-0.52508700
Ir	-0.81709900	0.01149800	-0.59130300

Intermediate (-)-2a·[Ir]

C	1.51498900	-1.35909400	1.05143200
H	1.28490900	-0.41265300	0.52878000
C	1.20926600	-1.47468200	2.50994100
O	1.19550900	-2.79313200	2.84316000
O	1.00285400	-0.60137400	3.30911700
C	1.20352300	-3.64035700	1.67972200
C	1.56807100	-2.75814600	0.49643400
H	0.19588400	-4.06665700	1.54951200
H	1.92256200	-4.45492000	1.85600500
C	2.84777600	-2.00078200	0.60173700
H	1.12891900	-2.98708000	-0.47691600
H	3.52577100	-2.27008400	1.41961300
C	3.52271100	-1.41605700	-0.59397500
C	2.83120900	-1.15569100	-1.79110800
C	4.88356400	-1.08132500	-0.51519300
C	3.49214700	-0.57436500	-2.87531000
H	1.77030500	-1.39945300	-1.88955400
C	5.54374500	-0.50500900	-1.60291000
H	5.43046800	-1.26879000	0.41282400

C	4.84922700	-0.24759000	-2.78822800
H	2.93787100	-0.37835600	-3.79669500
H	6.60412100	-0.25247700	-1.52139400
H	5.36310300	0.20440300	-3.64047700
Ir	-0.65740400	-0.05695100	-0.15356500
Cl	-0.95641600	-1.89045400	-1.61424800
C	-2.58471600	0.83638100	-0.06829300
C	-1.78042600	1.40800500	-1.11664400
C	-0.87573900	1.36489700	1.43650600
C	-0.08912300	1.94641100	0.40026000
C	-2.37475500	1.58180600	1.26013100
C	-3.77532600	-0.01935700	-0.29604400
C	-0.97084100	2.62169600	-0.66681800
H	-2.00168400	1.23278100	-2.17359700
H	-0.46053500	1.07196700	2.40336700
C	1.37930100	2.16548800	0.48268300
C	-2.73073000	3.07878200	1.10123300
H	-2.94723500	1.11982800	2.07320600
C	-4.18328100	-0.93691600	0.68965400
C	-4.51517200	0.05921400	-1.48729100
C	-1.87442400	3.70473100	-0.03068000
H	-0.37532400	3.03298400	-1.48992200
C	2.05442300	2.13888400	1.71732100
C	2.14480100	2.30242600	-0.69235800
H	-3.80691400	3.16949100	0.88339600
H	-2.55317900	3.58881200	2.06087100
C	-5.29535000	-1.75295200	0.48827200
H	-3.60031800	-1.03030100	1.60875400
C	-5.63286600	-0.75439100	-1.68769200
H	-4.22059800	0.76960700	-2.26210700
H	-2.51212400	4.14607500	-0.81316300
H	-1.23456400	4.51410600	0.35475300
C	3.44594000	2.24418400	1.77126400
H	1.49265900	2.00763800	2.64204300
C	3.53424500	2.40558100	-0.63707300
H	1.64972300	2.28079500	-1.66516000
C	-6.02625700	-1.66372700	-0.70222200

H	-5.59084100	-2.46795100	1.26027500
H	-6.19895600	-0.67684900	-2.61943300
C	4.19198900	2.37700100	0.59644200
H	3.94933300	2.21473400	2.74103100
H	4.10810700	2.47873500	-1.56301000
H	-6.89839200	-2.30302800	-0.86079800
H	5.28170300	2.44532800	0.63913100

Substrate 1a

C	-2.76374400	-0.80753800	0.03388200
C	-1.67210700	-0.30231900	-0.56325500
H	-2.65777600	-1.76066200	0.56700000
H	-1.71021700	0.65669200	-1.09111800
C	-4.11730300	-0.23021900	0.06563900
C	-5.12104700	-0.88237700	0.80807300
C	-4.46625900	0.95601700	-0.61279400
C	-6.41930500	-0.37199800	0.87777400
H	-4.87420100	-1.80551400	1.34040000
C	-5.76113800	1.46711800	-0.54353400
H	-3.71627800	1.48380000	-1.20573200
C	-6.74581900	0.80677200	0.20237300
H	-7.17882900	-0.89814600	1.46195800
H	-6.00759700	2.38813000	-1.07846600
H	-7.76067100	1.20924500	0.25305400
C	-0.34270100	-0.98271600	-0.58573000
H	-0.07158500	-1.29680100	-1.60997700
H	-0.33551100	-1.89410300	0.03643700
O	0.63914600	-0.05171600	-0.11164200
C	1.93636200	-0.44622700	-0.21553600
O	2.24364700	-1.53015100	-0.69755100
C	2.82268100	0.53924500	0.34418900
H	2.46951100	1.53225500	0.62114700
S	4.46617500	0.41775600	0.05823900
O	5.09912100	0.73427800	-1.24389700
C	4.99375800	-1.24406100	0.52574200
H	6.06693900	-1.30459500	0.30271800
H	4.76496700	-1.42151200	1.58416500
H	4.40032500	-1.90841300	-0.11655700

C	5.20149400	1.49488800	1.30828700
H	4.90618500	1.17860400	2.31608100
H	6.28780400	1.45230000	1.15474300
H	4.83357000	2.50581300	1.08637300

DMSO

S	-0.26320100	0.43935900	0.00000000
O	1.10898000	1.06637100	0.00000000
C	-0.26320100	-0.79214100	1.36845200
H	-1.18893400	-1.38726900	1.35845600
H	0.62847200	-1.42666800	1.25548800
H	-0.19065100	-0.21357900	2.29982800
C	-0.26320100	-0.79214100	-1.36845200
H	-1.18893400	-1.38726900	-1.35845600
H	-0.19065100	-0.21357900	-2.29982800
H	0.62847200	-1.42666800	-1.25548800

(–)-2a

C	2.49978000	-1.54068500	-0.16948900
C	1.16234900	-1.12041900	0.42203900
C	1.28190400	0.36811200	0.64730300
H	3.08862500	-2.13185200	0.55219700
H	2.40186900	-2.11706600	-1.10323400
H	0.67635900	-1.76948400	1.15390600
H	0.87125300	0.90586200	1.50294200
C	2.63574100	0.76005100	0.13602700
O	3.18300700	1.82264700	0.19646300
O	3.20895000	-0.33014700	-0.46303700
C	0.34220800	-0.19074400	-0.41606900
H	0.67157800	-0.05197100	-1.45529000
C	-1.13801500	-0.06013000	-0.21584600
C	-1.94909700	-1.18295100	0.00351200
C	-1.74164300	1.20660700	-0.28083600
C	-3.33241300	-1.04484600	0.15851400
H	-1.49457700	-2.17601000	0.04803900
C	-3.12239100	1.34696300	-0.12713400
H	-1.11652800	2.08816600	-0.44834200
C	-3.92244000	0.22021900	0.09425600
H	-3.95054700	-1.93005100	0.32932600

H	-3.57662700	2.33989000	-0.17750000
H	-5.00296300	0.32946400	0.21688700

Intermediate III

C	0.55535900	0.12957200	2.88930300
O	0.10528400	-0.97084900	3.58003200
O	0.45976000	1.23191600	3.37333100
C	0.31721700	-2.15702500	2.82520600
C	1.38529800	-1.79569500	1.77374500
H	-0.62812800	-2.45575100	2.33842200
H	0.63314800	-2.96021100	3.50862000
C	1.37468300	-2.69969200	0.55418800
H	1.20134200	-3.74776100	0.83441800
C	1.10844800	-0.29266200	1.56719300
H	1.99938700	0.30285600	1.33542800
C	2.52996400	-2.59442100	-0.39645200
C	2.95400300	-1.36120700	-0.92591800
C	3.22203700	-3.76089200	-0.75887900
C	4.05253200	-1.30343600	-1.78298900
H	2.41418600	-0.44521500	-0.68636500
C	4.32573600	-3.70086000	-1.61467500
H	2.89686500	-4.72739900	-0.36310000
C	4.74479200	-2.47044600	-2.12740300
H	4.36863700	-0.33542100	-2.17949500
H	4.85669300	-4.61797600	-1.88152100
H	5.60730300	-2.42040500	-2.79687700
H	2.38899900	-1.93604700	2.21908400
Ir	-0.39353500	0.09629700	0.07237000
Cl	-0.24712300	-2.29334700	-0.40896500
C	-2.37363600	0.72646200	-0.69153400
C	-1.43315100	0.83238500	-1.73405100
C	-0.95533900	2.03749200	0.67133800
C	0.00140300	2.14755700	-0.39184800
C	-2.39905100	1.99542800	0.18018500
C	-3.43515000	-0.30722000	-0.61036700
C	-0.69732200	2.16846600	-1.76277800
H	-1.43475700	0.17005600	-2.60354700
H	-0.72934500	2.31658700	1.70254700

C	1.41497200	2.58019500	-0.22423500
C	-2.73240900	3.23211900	-0.69073900
H	-3.10104900	1.89613300	1.01671900
C	-3.91181400	-0.73542300	0.64441500
C	-3.96718400	-0.90704300	-1.76542200
C	-1.69320500	3.35400600	-1.83536800
H	0.01630400	2.20645100	-2.59260000
C	1.90502900	3.01641500	1.02237500
C	2.32488200	2.49257100	-1.29636600
H	-3.75411600	3.12596600	-1.08893900
H	-2.72692100	4.12962700	-0.05242700
C	-4.88223000	-1.73301500	0.73916300
H	-3.49085800	-0.29878300	1.55312700
C	-4.93897900	-1.90671200	-1.67064600
H	-3.62513200	-0.57651600	-2.74852600
H	-2.18541100	3.34699100	-2.82083000
H	-1.12945200	4.29791000	-1.76401300
C	3.25059500	3.34973200	1.18483000
H	1.24477500	3.04295900	1.88877400
C	3.67077500	2.82773500	-1.13295500
H	1.98631100	2.13552500	-2.27023600
C	-5.40054000	-2.32433100	-0.41891900
H	-5.23279500	-2.05560800	1.72302700
H	-5.34304800	-2.35692500	-2.58128600
C	4.14172000	3.26064500	0.11011700
H	3.60600700	3.67570100	2.16571700
H	4.35455100	2.74903900	-1.98263400
H	-6.16188400	-3.10501900	-0.34509300
H	5.19467300	3.52250300	0.24150500

TS5

C	-0.60905900	-1.93709900	2.08299600
O	-0.98527100	-3.20102100	1.79614200
O	-1.17787700	-1.25820600	2.89947700
C	-0.07089900	-3.85306300	0.90376600
C	1.15829300	-2.93352800	0.78830100
H	-0.57933600	-3.99002200	-0.06240800
H	0.18055000	-4.83169700	1.33645900

C	1.23974000	-2.09202700	-0.42239900
H	0.49318100	-2.30388000	-1.18822800
C	0.66419800	-1.61182000	1.32289400
H	1.39263500	-0.97481000	1.82690000
C	2.50336500	-1.52094000	-0.91477700
C	3.69447600	-1.51989200	-0.16739900
C	2.53630400	-1.05302000	-2.24419000
C	4.89285500	-1.10650800	-0.74905200
H	3.69089300	-1.84041700	0.87600900
C	3.73609000	-0.64569100	-2.82416300
H	1.60576700	-1.02780200	-2.81664600
C	4.92108200	-0.68096700	-2.08055200
H	5.80930700	-1.10771600	-0.15518400
H	3.74891000	-0.30169400	-3.86137800
H	5.86323300	-0.36231600	-2.53339800
H	2.09273000	-3.33475900	1.19719800
Ir	-0.43520500	-0.19644900	-0.12390500
Cl	-1.69467300	-1.74075300	-1.46467500
C	-2.04068900	1.29824300	-0.13042300
C	-1.10427800	1.58528200	-1.15302600
C	-0.24244100	1.28080900	1.39410000
C	0.70409700	1.61405700	0.38387600
C	-1.61001300	1.91807800	1.20567300
C	-3.43222400	0.83330400	-0.33600200
C	0.04637900	2.47953600	-0.70688600
H	-1.34489800	1.44850500	-2.21043400
H	0.03501800	0.93273300	2.38992500
C	2.17293500	1.64649800	0.60991400
C	-1.48801000	3.45635300	1.05629800
H	-2.29322600	1.63923600	2.01537000
C	-4.05170200	0.02288300	0.63374700
C	-4.15302500	1.18180900	-1.48835000
C	-0.49072100	3.79381100	-0.08281800
H	0.72640800	2.68357600	-1.54098800
C	2.72362100	1.41119600	1.88675800
C	3.06372600	1.97107000	-0.43004600
H	-2.48622600	3.87382500	0.85019400

H	-1.15309900	3.87969600	2.01626100
C	-5.35957300	-0.42259900	0.45203500
H	-3.48343600	-0.29408300	1.51198200
C	-5.46581300	0.73802900	-1.66784700
H	-3.68584500	1.81499800	-2.24582100
H	-0.97470100	4.39378300	-0.86979600
H	0.36147200	4.38212600	0.29273500
C	4.09834300	1.50097200	2.11274900
H	2.06638500	1.18642500	2.72802200
C	4.43582900	2.07750000	-0.20220500
H	2.68351000	2.13197600	-1.43907700
C	-6.07275800	-0.06490900	-0.69870100
H	-5.82236500	-1.06375500	1.20646300
H	-6.01614600	1.02139000	-2.56883200
C	4.96367300	1.84482500	1.07038400
H	4.49222900	1.32073400	3.11653500
H	5.09931000	2.33068300	-1.03240500
H	-7.09827100	-0.41585600	-0.84027800
H	6.03865000	1.92915000	1.24808700

Intermediate (+)-2a·[Ir]

C	3.22680300	2.53454500	1.25570500
O	4.18437100	3.10644300	0.47602800
O	3.48570600	1.78621400	2.15894900
C	3.64534600	4.12746200	-0.37885900
C	2.13043700	3.98893300	-0.30887100
H	4.05157400	3.97546800	-1.39091700
H	3.97636500	5.11053300	-0.00493900
C	1.58811400	2.63896600	-0.68355400
H	2.28614300	1.93954100	-1.15642700
C	1.88271900	2.98287900	0.78034500
H	1.06204300	3.00900700	1.49765500
C	0.16871300	2.42865800	-1.08024200
C	-0.88963900	3.17325000	-0.55493800
C	-0.12843700	1.36885700	-1.97430900
C	-2.22417200	2.86007000	-0.86857700
H	-0.69132200	4.00090600	0.13155500
C	-1.46834600	1.03756000	-2.26874100

H	0.67843900	0.87750500	-2.52050200
C	-2.51974000	1.79022300	-1.70206100
H	-3.03337700	3.44585100	-0.42806400
H	-1.67419200	0.27942200	-3.02721300
H	-3.55676200	1.52609100	-1.91606100
H	1.50770300	4.87752600	-0.43799300
Ir	-0.51190600	-0.74321700	-0.66329300
Cl	0.07841800	-1.85102500	-2.69191400
C	0.86444900	-1.56556100	0.80612000
C	-0.29079300	-2.40080000	0.60244400
C	-0.59838000	0.20292700	1.26244200
C	-1.73753100	-0.63137700	1.09175100
C	0.59816000	-0.50648000	1.88457700
C	2.24165300	-1.93139600	0.38590300
C	-1.44778300	-2.07386200	1.54157000
H	-0.21002000	-3.39100300	0.14721700
H	-0.68552600	1.29113300	1.30009800
C	-3.13262500	-0.13777100	0.95962400
C	0.22272700	-1.18266800	3.22619900
H	1.43884400	0.18341100	2.00996900
C	3.36092300	-1.27029400	0.92677400
C	2.46924600	-2.96026100	-0.55241800
C	-1.01023500	-2.10313200	3.02603100
H	-2.30858700	-2.72972900	1.35934900
C	-3.56587900	1.01504400	1.63328900
C	-4.04717700	-0.81730100	0.13448400
H	1.09077300	-1.75175400	3.59468300
H	0.01608100	-0.39798300	3.97118100
C	4.65876000	-1.62258300	0.54502000
H	3.23992400	-0.46734700	1.65175200
C	3.76304900	-3.30976200	-0.93086800
H	1.62428700	-3.45634300	-1.02850000
H	-0.78060100	-3.14233900	3.31019300
H	-1.85661600	-1.77894800	3.65267000
C	-4.87105400	1.48925700	1.47228600
H	-2.86968100	1.54561100	2.28640400
C	-5.34907900	-0.34378300	-0.03089700

H	-3.71218500	-1.70490200	-0.40853400
C	4.86724200	-2.64359400	-0.38388000
H	5.50603600	-1.08698800	0.98061000
H	3.91138400	-4.10242200	-1.66869800
C	-5.76583000	0.81545600	0.63559000
H	-5.19150700	2.38872100	2.00491900
H	-6.04202300	-0.87726100	-0.68673200
H	5.88145400	-2.91790900	-0.68589400
H	-6.78549400	1.18732600	0.50684800

(+)-2a

C	-1.28189200	0.36804600	0.64726500
C	-1.16234600	-1.12050100	0.42182800
H	-0.87108800	0.90559400	1.50296000
H	-0.67649200	-1.76966700	1.15370200
O	-3.20900200	-0.33003600	-0.46311700
C	-0.34220500	-0.19075400	-0.41609300
H	-0.67131700	-0.05180900	-1.45538400
C	1.13804200	-0.06012500	-0.21581500
C	1.74166600	1.20656600	-0.28106200
C	1.94910600	-1.18293800	0.00377000
C	3.12243900	1.34693700	-0.12736000
H	1.11659600	2.08812200	-0.44875300
C	3.33240000	-1.04481900	0.15877000
H	1.49455500	-2.17597300	0.04848400
C	3.92245500	0.22024500	0.09427400
H	3.57667600	2.33985600	-0.17791700
H	3.95055100	-1.92997800	0.32977700
H	5.00297900	0.32948400	0.21690900
C	-2.63576200	0.76004500	0.13615700
O	-3.18304700	1.82261700	0.19687200
C	-2.49981200	-1.54062600	-0.16975600
H	-3.08864900	-2.13183700	0.55188900
H	-2.40196400	-2.11689900	-1.10357900

Intermediate II

O	-1.51345800	-1.14157700	1.20626000
C	-1.43850800	-1.23799600	0.00030300
O	-2.50306200	-1.37195600	-0.80538000

H	-0.06272700	-2.14232800	-1.43334700
C	-3.79757700	-1.33231500	-0.17393000
C	-4.82304500	-1.48323300	-1.25228400
H	-3.90445800	-0.38829900	0.38448400
H	-3.84430600	-2.14790600	0.57117500
C	-6.06581600	-0.96840500	-1.27897900
H	-4.48415000	-2.06980900	-2.11241800
H	-6.64824700	-1.14575500	-2.19020600
C	-6.74958900	-0.15235300	-0.26044900
C	-6.50400000	-0.27279400	1.12271600
C	-7.71450600	0.78741800	-0.67929100
C	-7.17010200	0.53965600	2.04343300
H	-5.81500000	-1.03424200	1.49074700
C	-8.37636000	1.60161300	0.23936100
H	-7.93503100	0.88370700	-1.74609800
C	-8.10254700	1.48496500	1.60677800
H	-6.96562400	0.42472700	3.11103100
H	-9.11214100	2.32971400	-0.11183700
H	-8.62273100	2.11947300	2.32872800
Cl	0.88677800	1.54277800	-2.35763700
C	-0.17676700	-1.24833800	-0.79393700
Ir	1.17579200	0.00148800	-0.65183500
C	1.62171600	-0.47522300	1.38875500
C	2.71672600	1.52900400	0.61892400
C	3.47287800	0.75200900	-0.22900500
C	2.44308100	-1.28662500	0.54041700
C	2.33119900	0.77435700	1.89347200
H	0.77623800	-0.88087600	1.94793400
C	2.42588500	2.96579700	0.43304300
C	3.81199900	-0.62807900	0.30933800
H	3.90797700	1.13397100	-1.15409200
C	2.28711800	-2.75846900	0.37803900
C	3.63475800	0.35950300	2.63456600
H	1.68097700	1.37309900	2.54099300
C	3.33753700	3.80659900	-0.22898500
C	1.21800000	3.51687300	0.89872300
C	4.52024300	-0.49014300	1.68620900

H	4.42007000	-1.20050700	-0.40069600
C	1.64933500	-3.53796400	1.35777100
C	2.75722500	-3.39655300	-0.78470100
H	3.36108900	-0.20762200	3.53793100
H	4.16246000	1.26601200	2.96956800
C	3.05121100	5.15951500	-0.41895900
H	4.28613900	3.39877000	-0.58432800
C	0.93002600	4.86738000	0.70520000
H	0.48094600	2.86966600	1.37853500
H	4.69397100	-1.49736200	2.09638700
H	5.50699300	-0.02465000	1.53864400
C	1.48244700	-4.91416700	1.17823800
H	1.28069900	-3.06361000	2.26914200
C	2.59268100	-4.77024200	-0.96352500
H	3.22609400	-2.79818300	-1.56958400
C	1.84658900	5.69461900	0.04708400
H	3.77376200	5.79980900	-0.93129100
H	-0.01990800	5.27503700	1.05974600
C	1.95339700	-5.53612100	0.01874800
H	0.98437300	-5.50341500	1.95257200
H	2.95788600	-5.24583900	-1.87743600
H	1.62111300	6.75335700	-0.10375400
H	1.82309300	-6.61219500	-0.12085900

Intermediate (+)-2f·[Ir]

C	-2.02669900	-1.87580400	1.04374400
H	-1.26296400	-1.66954300	1.79314400
C	-1.60566500	-1.80632200	-0.37898500
O	-2.30422900	-2.65171000	-1.13258400
O	-0.75670400	-1.08530600	-0.88576000
C	-3.10471900	-3.54400200	-0.32383400
C	-3.01988100	-3.01695800	1.09985500
H	-2.67480200	-4.55301000	-0.41409000
H	-4.12341300	-3.53628100	-0.73902500
C	-3.50346500	-1.60737500	1.30564100
H	-3.01625200	-3.73387900	1.92359200
H	-3.68968700	-1.35338100	2.35564100
C	-4.43879200	-0.94214900	0.33803700

C	-5.76970500	-1.39042300	0.27255400
C	-4.03816600	0.11297600	-0.49190100
C	-6.67957900	-0.79940500	-0.60615500
H	-6.09047500	-2.21302100	0.91877000
C	-4.94718700	0.70033900	-1.37727000
H	-3.01504800	0.48869900	-0.44393200
C	-6.26707300	0.24786700	-1.43818800
H	-7.71118900	-1.15866100	-0.64553000
H	-4.61503000	1.52295700	-2.01388700
H	-6.97524700	0.70911300	-2.13129500
Ir	0.98349300	-0.16755100	0.01910500
Cl	1.36779900	-2.28683500	1.02827100
C	2.95232500	0.63312200	-0.08672500
C	2.33778600	0.96969800	1.15727700
C	1.11592300	1.57286300	-1.20080300
C	0.51186000	1.91791500	0.04590200
C	2.63924600	1.67526100	-1.17802100
C	4.10137500	-0.29846800	-0.23026400
C	1.56777300	2.28458900	1.10953500
H	2.67276600	0.53687100	2.10409400
H	0.56244100	1.59046200	-2.14477500
C	-0.89659700	2.35899000	0.22059400
C	3.11787800	3.07792400	-0.73362900
H	3.08096600	1.38707100	-2.14012100
C	4.26675700	-1.04088600	-1.41331800
C	5.04572300	-0.45503500	0.79651500
C	2.45220100	3.45469600	0.61716400
H	1.09967100	2.51581300	2.07461600
C	-1.58345100	3.05421300	-0.78964500
C	-1.56521300	2.12223900	1.43802700
H	4.21623600	3.06842300	-0.64604900
H	2.86508500	3.80949600	-1.51769900
C	5.34231400	-1.91511300	-1.56369200
H	3.52022400	-0.95188400	-2.20610400
C	6.12630000	-1.32860800	0.64640300
H	4.93734500	0.11879400	1.71926100
H	3.21111900	3.68023600	1.38344900

H	1.82463800	4.35458100	0.51392200
C	-2.88806900	3.51413600	-0.58302000
H	-1.07950100	3.25673800	-1.73710500
C	-2.86715200	2.57823700	1.64349400
H	-1.05248900	1.55675900	2.22008100
C	6.27859100	-2.06175500	-0.53327000
H	5.44739000	-2.49333400	-2.48541000
H	6.85296200	-1.43596400	1.45609000
C	-3.53366500	3.28217800	0.63410700
H	-3.40078000	4.06225400	-1.37804100
H	-3.37013200	2.37721300	2.59292900
H	7.12170800	-2.74761700	-0.64989300
H	-4.55546100	3.63517200	0.79167500

Substrate 1f

C	-2.98854300	-1.74808900	-0.27848600
C	-1.65920200	-1.75993900	-0.48883800
H	-3.48187500	-2.72477800	-0.21492900
H	-1.16463700	-2.72860300	-0.61688400
C	-3.88504300	-0.59628000	-0.07781300
C	-5.05463300	-0.77284300	0.69128400
C	-3.64919800	0.67917300	-0.63159000
C	-5.93044700	0.28567700	0.93116400
H	-5.26853400	-1.75872800	1.11384200
C	-4.52837700	1.73860300	-0.39456000
H	-2.79162400	0.84064100	-1.28547100
C	-5.66786900	1.55044900	0.39292100
H	-6.82486500	0.12357800	1.53850500
H	-4.32518300	2.71654700	-0.83872500
H	-6.35439300	2.38089500	0.57620600
C	-0.72675500	-0.59055700	-0.55627200
H	-1.06507800	0.25128800	0.07125400
H	-0.63791500	-0.19136000	-1.58411600
O	0.55705900	-1.04354800	-0.11805200
C	1.56763200	-0.13795500	-0.20156800
O	1.38630300	0.98925200	-0.64715400
H	2.89169200	-1.73396800	0.57266300
C	2.79108200	-0.67646800	0.32976100

S	4.22757100	0.13617800	0.05769300
O	4.92237400	0.16058800	-1.25105300
C	4.01019800	1.84841700	0.58627900
H	3.73743700	1.87649300	1.64878800
H	4.95590700	2.36313400	0.37225300
H	3.18634000	2.22246800	-0.03611800
C	5.35978700	-0.57266900	1.27381700
H	6.32533900	-0.07064800	1.12843200
H	4.96803600	-0.44507200	2.29025600
H	5.45052500	-1.63618100	1.01442900

(+)-2f

C	-1.87338900	-0.10182400	-0.98060000
H	-2.52892800	0.08387900	-1.83286800
C	-1.94130400	0.87660800	0.15932500
O	-1.65560700	0.22845400	1.32246700
O	-2.19935000	2.04589800	0.11640100
C	-1.57456700	-1.19119800	1.14853200
C	-1.62171900	-1.45268200	-0.35073200
H	-2.43003500	-1.66008600	1.66215400
H	-0.64057400	-1.53780300	1.61842400
C	-0.54275800	-0.80729200	-1.17882200
H	-2.11421400	-2.36045600	-0.70719400
H	-0.44515600	-1.24340700	-2.18056900
C	0.75724100	-0.32585500	-0.60379000
C	1.67755500	-1.26168600	-0.10400000
C	1.09783600	1.03468000	-0.58667400
C	2.91197100	-0.84778300	0.40258200
H	1.42007400	-2.32491500	-0.11506500
C	2.33192700	1.45025300	-0.07814200
H	0.38614500	1.77412500	-0.96062200
C	3.24193500	0.51158700	0.41658800
H	3.61759700	-1.58753200	0.78961700
H	2.58075400	2.51437700	-0.06605600
H	4.20562000	0.83815400	0.81563400

Intermediate IV

C	0.13057000	-1.67322200	2.19604000
O	-1.02580300	-1.33928600	2.86520300

O	1.15363800	-1.85222500	2.81098000
C	-2.09525100	-1.14782200	1.94699500
C	-1.66935100	-1.90832800	0.67956300
H	-2.20482800	-0.06831200	1.73958900
H	-3.02661600	-1.51931500	2.39757800
C	-2.36317900	-1.51059600	-0.61384900
H	-1.97116400	-2.12311700	-1.43594200
C	-0.13960100	-1.73078900	0.72850500
H	0.43303300	-2.54268800	0.25785500
C	-3.86774300	-1.52723500	-0.61170400
C	-4.63215900	-0.53790500	0.02886500
C	-4.52723100	-2.60720000	-1.21890200
C	-6.02393200	-0.63582100	0.06863000
H	-4.13622500	0.32525100	0.47598600
C	-5.92039100	-2.70908600	-1.17139100
H	-3.94413200	-3.37845300	-1.73002400
C	-6.67229900	-1.72367900	-0.52637500
H	-6.60625300	0.14446600	0.56481500
H	-6.41848000	-3.55785700	-1.64642000
H	-7.76204900	-1.79807400	-0.49346100
H	-1.93213500	-2.97711200	0.80830900
Ir	0.51609200	0.12793800	-0.14765700
Cl	-1.73645800	0.21843200	-1.08836600
C	1.17520500	2.23339700	-0.15204200
C	1.56024900	1.63881800	-1.37091100
C	2.24981200	0.47812000	1.00391800
C	2.64400100	-0.12938600	-0.23213900
C	2.22632000	2.00258300	0.94826300
C	0.07796200	3.22185700	-0.00215900
C	2.91282200	0.93672400	-1.30992900
H	1.10351300	1.90071800	-2.32883300
H	2.31794400	-0.04374000	1.96081400
C	3.15151400	-1.52013400	-0.37277800
C	3.59801600	2.56852100	0.50364600
H	1.90989700	2.43244900	1.90623700
C	-0.63247000	3.31127300	1.21145000
C	-0.31113300	4.05718300	-1.06395100

C	4.02525100	1.90386900	-0.83088900
H	3.17225800	0.48931500	-2.27569500
C	3.50244200	-2.29309500	0.74944200
C	3.25285000	-2.11567100	-1.64543000
H	3.51697800	3.66208100	0.39701900
H	4.33721200	2.37632400	1.29719300
C	-1.69860900	4.19947200	1.35392900
H	-0.36033700	2.65189700	2.03895300
C	-1.37993800	4.94638700	-0.92197200
H	0.23776000	4.01771400	-2.00726700
H	4.20271000	2.66008900	-1.61200100
H	4.96264800	1.33724800	-0.71233600
C	3.94128000	-3.61005500	0.60056300
H	3.39849500	-1.87825400	1.75021900
C	3.69329200	-3.43157500	-1.79411500
H	2.96112900	-1.55030600	-2.53251100
C	-2.07896600	5.02120900	0.28622200
H	-2.23987500	4.24812100	2.30251700
H	-1.66352500	5.58971700	-1.75918600
C	4.04289400	-4.18606700	-0.66949700
H	4.20183400	-4.19114700	1.48887800
H	3.75999000	-3.87106500	-2.79296300
H	-2.91360000	5.71804600	0.39747400
H	4.38852300	-5.21694600	-0.78241900

TS6

C	-2.24441300	2.19955400	-0.27563400
O	-2.10146800	3.55854800	-0.18281100
O	-3.08577900	1.62560700	0.35206700
C	-1.13799800	4.06632600	-1.10809600
C	-0.43808400	2.85324700	-1.70308000
H	-0.45434400	4.73956400	-0.56783300
H	-1.66345400	4.64167700	-1.88720900
C	0.54477300	2.07679800	-0.90954300
H	1.26146500	1.58302900	-1.56694900
C	-1.24862100	1.64808500	-1.26118900
H	-1.66385400	0.98801100	-2.03935700
C	1.10794800	2.36347600	0.43301400

C	0.43469600	2.97607400	1.50859800
C	2.45202600	1.97969900	0.62274600
C	1.08752800	3.18315500	2.72638400
H	-0.60612100	3.28019700	1.42136100
C	3.10397500	2.20158800	1.83365700
H	2.98446500	1.47679900	-0.18675300
C	2.42133000	2.80031800	2.89746900
H	0.54218800	3.64980300	3.55055300
H	4.14301100	1.88465100	1.94715700
H	2.92410000	2.96619200	3.85350000
H	-0.19300800	2.92077700	-2.76698900
Ir	-0.13666800	-0.31618300	-0.77304500
Cl	0.57963800	-0.34467900	-3.09091200
C	1.35265300	-1.43169900	0.35888300
C	0.47368300	-2.31048400	-0.35716000
C	-0.52933200	-0.43080900	1.34505200
C	-1.39372900	-1.33538500	0.67159100
C	0.79828300	-1.06554800	1.74283600
C	2.80865400	-1.32116900	0.06694000
C	-0.74396000	-2.71161300	0.46132100
H	0.83631600	-2.93280900	-1.17951200
H	-0.90961400	0.45727400	1.85521000
C	-2.87484000	-1.25240500	0.61519100
C	0.57703700	-2.34097100	2.59392200
H	1.43579200	-0.33360200	2.25145500
C	3.73256900	-1.17438100	1.11881000
C	3.30816400	-1.39413700	-1.24836600
C	-0.35046100	-3.32238600	1.82899200
H	-1.41025800	-3.37604900	-0.10257500
C	-3.61340000	-0.92934300	1.76204100
C	-3.55915100	-1.56188100	-0.57134200
H	1.55137900	-2.80622300	2.81257500
H	0.13601300	-2.04979800	3.56053200
C	5.10526000	-1.09855800	0.86854400
H	3.38311400	-1.11980700	2.15041900
C	4.67817000	-1.30681500	-1.49728100
H	2.61213400	-1.47108300	-2.08460200

H	0.14838200	-4.28920100	1.65669500
H	-1.26845800	-3.53040100	2.40134300
C	-5.00852700	-0.90256800	1.72182800
H	-3.08556600	-0.68527900	2.68675200
C	-4.95275200	-1.52088800	-0.61664700
H	-2.98430200	-1.81007900	-1.46818900
C	5.58565000	-1.15799600	-0.44205200
H	5.80105900	-0.98969900	1.70474400
H	5.03898400	-1.34943300	-2.52820400
C	-5.68201400	-1.19151100	0.53164800
H	-5.57293300	-0.64408400	2.62149600
H	-5.47376600	-1.74558300	-1.55099900
H	6.65820500	-1.08886900	-0.64068200
H	-6.77409900	-1.16022600	0.49752300

Intermediate (–)-2f·[Ir]

C	-1.93922200	3.20363600	0.85655300
O	-1.55968200	4.50591000	0.92663600
O	-2.40353500	2.62900200	1.80677700
C	-1.10107600	5.00649500	-0.34096800
C	-1.15457800	3.84243000	-1.31802200
H	-0.08320600	5.40453300	-0.19883900
H	-1.76779200	5.82644600	-0.64971700
C	-0.26343200	2.65320900	-1.05987000
H	-0.20816900	2.00969500	-1.94300600
C	-1.69632500	2.67681700	-0.52396400
H	-2.40754300	1.93304900	-0.89058400
C	1.02470500	2.60914400	-0.29174700
C	1.21992800	3.09524200	1.01390800
C	2.12269400	2.01589500	-0.94655900
C	2.47118700	2.99554500	1.63273500
H	0.40851600	3.55390200	1.57547600
C	3.36879200	1.91987900	-0.32879800
H	1.98298400	1.58898800	-1.94159000
C	3.55054400	2.41223400	0.96609700
H	2.59515500	3.37846100	2.64921400
H	4.19202000	1.42536800	-0.84872600
H	4.52271500	2.32349300	1.45664000

H	-1.47798300	4.04519300	-2.34207100
Ir	-0.20695300	-0.84012600	-0.76501300
Cl	0.36333800	-0.52176900	-3.01732500
C	1.25720200	-1.46604100	0.64739900
C	0.43774500	-2.56833300	0.17901000
C	-0.68969500	-0.39771700	1.26801200
C	-1.49241000	-1.49312000	0.83008200
C	0.64040900	-0.80831000	1.88517100
C	2.70899900	-1.36793500	0.34475400
C	-0.77751800	-2.83275100	1.05843000
H	0.86892100	-3.36298000	-0.43995400
H	-1.10086800	0.59406600	1.46664400
C	-2.93667200	-1.39627700	0.49691400
C	0.44803700	-1.81785500	3.04009000
H	1.21096100	0.07709600	2.19131000
C	3.61951600	-1.01874000	1.35704600
C	3.21447400	-1.65444900	-0.93833800
C	-0.39122700	-3.02508800	2.54444600
H	-1.36044200	-3.68362200	0.68825700
C	-3.69809300	-0.27195900	0.87307000
C	-3.57056200	-2.41218000	-0.24668900
H	1.43273200	-2.14656700	3.40838200
H	-0.05245300	-1.30467600	3.87609400
C	4.99231600	-0.96667100	1.10179600
H	3.25828400	-0.78288900	2.35900600
C	4.58326600	-1.58932900	-1.19560300
H	2.52336400	-1.88316200	-1.75164100
H	0.17124400	-3.96700000	2.64553000
H	-1.31326200	-3.13824100	3.13607700
C	-5.04517300	-0.17337600	0.51668000
H	-3.24297500	0.53683500	1.44535300
C	-4.91617100	-2.31028800	-0.60104200
H	-2.99841300	-3.28245700	-0.57387900
C	5.48068800	-1.24748600	-0.17622700
H	5.68127700	-0.69595000	1.90603400
H	4.95231500	-1.79931000	-2.20271600
C	-5.66166500	-1.18957800	-0.21985300

H	-5.61613500	0.70673700	0.82408900
H	-5.38405000	-3.10853000	-1.18296500
H	6.55321700	-1.19643500	-0.38025800
H	-6.71618500	-1.10982800	-0.49600000

(–)-2f

C	1.94122800	0.87668300	0.15961700
O	1.65551200	0.22820600	1.32248600
O	2.19930400	2.04597800	0.11705300
C	1.57473100	-1.19150600	1.14814800
C	1.62184600	-1.45248000	-0.35114300
H	0.64086900	-1.53835700	1.61811200
H	2.43038800	-1.66025300	1.66156000
C	0.54280000	-0.80694500	-1.17897000
H	0.44520600	-1.24292100	-2.18078300
C	1.87339900	-0.10146100	-0.98066500
H	2.52888700	0.08432400	-1.83295400
C	-0.75725300	-0.32578200	-0.60383900
C	-1.09810700	1.03471700	-0.58688500
C	-1.67730800	-1.26169900	-0.10379500
C	-2.33220200	1.45013300	-0.07829200
H	-0.38656000	1.77420900	-0.96100900
C	-2.91176700	-0.84794300	0.40284300
H	-1.41967300	-2.32488800	-0.11474000
C	-3.24200200	0.51134400	0.41663700
H	-2.58124900	2.51420600	-0.06629000
H	-3.61721900	-1.58779600	0.78999000
H	-4.20571500	0.83780500	0.81569900
H	2.11434600	-2.36016000	-0.70784000

((R,R)-3)IrCl

Ir	-0.03682500	-0.79236900	-0.12177500
Cl	0.71985300	-2.94611600	0.22140800
C	1.29470300	0.83911300	0.06384500
C	0.47738900	0.68553100	1.24748200
C	-0.64900900	0.97238300	-1.17377900
C	-1.45255200	0.83288900	-0.00537400
C	0.62573400	1.77548400	-0.95207000
C	2.75404700	0.56873500	0.02136100

C	-0.80145100	1.50949200	1.21205500
H	0.89775400	0.33917200	2.19692200
H	-1.04884300	0.82231700	-2.18093700
C	-2.89046000	0.46008300	-0.01626100
C	0.34895900	3.16353700	-0.32976200
H	1.22053800	1.85779100	-1.86995300
C	3.37180600	0.20880500	-1.19096400
C	3.54903500	0.67188000	1.17515900
C	-0.52556800	3.00700200	0.94247000
H	-1.40024000	1.36744200	2.11987800
C	-3.71621400	0.75738900	-1.11447600
C	-3.45670300	-0.21213900	1.08439700
H	1.31143500	3.64540800	-0.09443100
H	-0.15127000	3.79721600	-1.07902000
C	4.74156400	-0.04297000	-1.24638200
H	2.76000000	0.09457200	-2.08881300
C	4.92345400	0.42483000	1.11905800
H	3.09218200	0.96005000	2.12419000
H	-0.02768400	3.44507200	1.82222000
H	-1.48984500	3.52728100	0.82938700
C	-5.06583800	0.39483600	-1.11333400
H	-3.30153700	1.29175400	-1.97196400
C	-4.80298000	-0.57631900	1.08475800
H	-2.82163500	-0.47562700	1.93364900
C	5.52398800	0.06585600	-0.09031600
H	5.20208100	-0.33398600	-2.19382900
H	5.52713000	0.51410700	2.02582700
C	-5.61431900	-0.27306500	-0.01488900
H	-5.69293100	0.63975900	-1.97452900
H	-5.22108100	-1.10608600	1.94446600
H	6.59791800	-0.13214500	-0.13326400
H	-6.66943700	-0.55782700	-0.01487900

Carbene 1

C	-0.50247000	-0.66907300	-0.16650700
C	0.55517400	0.11011300	-0.44814700
H	-0.31719800	-1.73701200	0.00400000
H	0.44003900	1.18630400	-0.61247600

C	-1.91231000	-0.26422100	-0.05789000
C	-2.87382800	-1.23361000	0.28738800
C	-2.35375100	1.05663600	-0.27952900
C	-4.22438400	-0.90066200	0.41167900
H	-2.55322000	-2.26461100	0.46220200
C	-3.70106400	1.39018000	-0.15508700
H	-1.63655100	1.83284500	-0.55456600
C	-4.64383500	0.41373500	0.19132000
H	-4.95122400	-1.67101800	0.68142500
H	-4.02138800	2.42026100	-0.33117500
H	-5.69975100	0.67870400	0.28680800
C	1.94565200	-0.41004700	-0.57751400
H	2.35792900	-0.23865100	-1.58627500
H	2.00654600	-1.48973100	-0.36764200
O	2.78691000	0.30752600	0.37420000
C	4.08590400	0.15357100	0.24178800
O	4.68130200	-0.56262700	-0.57177600
H	5.57521800	0.26620100	1.69119700
C	5.13389700	0.86296500	0.87035100

Carbene 2

C	0.73243000	1.69140700	-0.40113500
C	-0.60175400	1.55475900	-0.50622000
H	1.12353400	2.71396600	-0.45036200
H	-1.20800500	2.45136100	-0.67116400
C	1.75138400	0.64894000	-0.18666300
C	2.91939200	0.97790200	0.53207000
C	1.62816600	-0.66519900	-0.68184500
C	3.90388000	0.02275400	0.78370300
H	3.04472300	1.99758200	0.90685400
C	2.61648700	-1.62080400	-0.43380700
H	0.77284400	-0.93689600	-1.30259700
C	3.75353700	-1.28407300	0.30604100
H	4.79535200	0.29872200	1.35271400
H	2.50131100	-2.63229800	-0.83147600
H	4.52582400	-2.03280100	0.49896900
C	-1.39386800	0.29138600	-0.40162700
H	-0.90031200	-0.46848800	0.22443600

H	-1.58680800	-0.16883100	-1.38699600
O	-2.67174200	0.63780800	0.19637400
C	-3.56793100	-0.32291900	0.25419800
O	-3.45922500	-1.47934200	-0.16974500
H	-5.64446500	-0.19149900	0.23342900
C	-4.80443400	-0.34391100	0.93747900

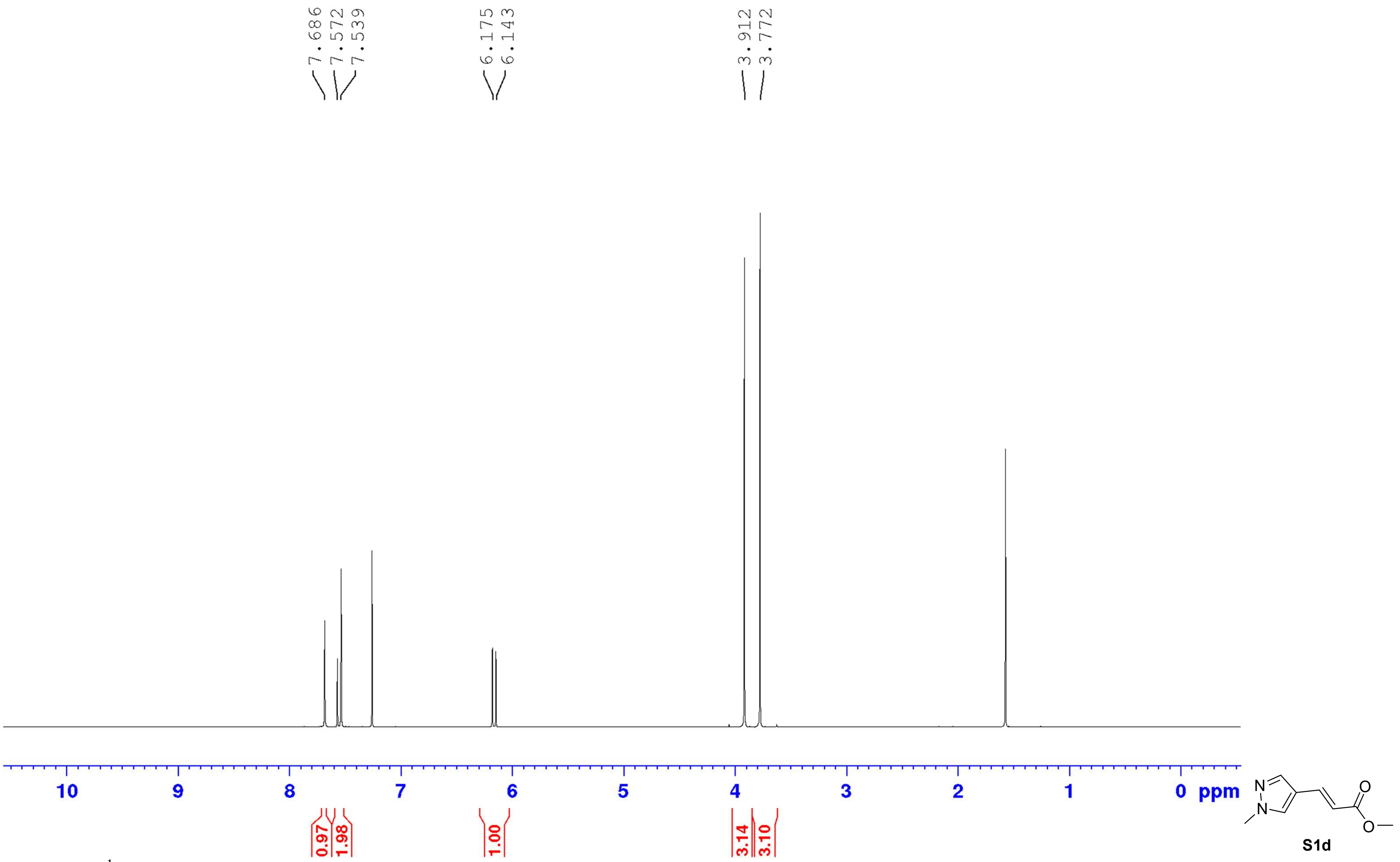

Figure S9. ^{1}H NMR (500 MHz, $CDCl_3$) of **S1d**

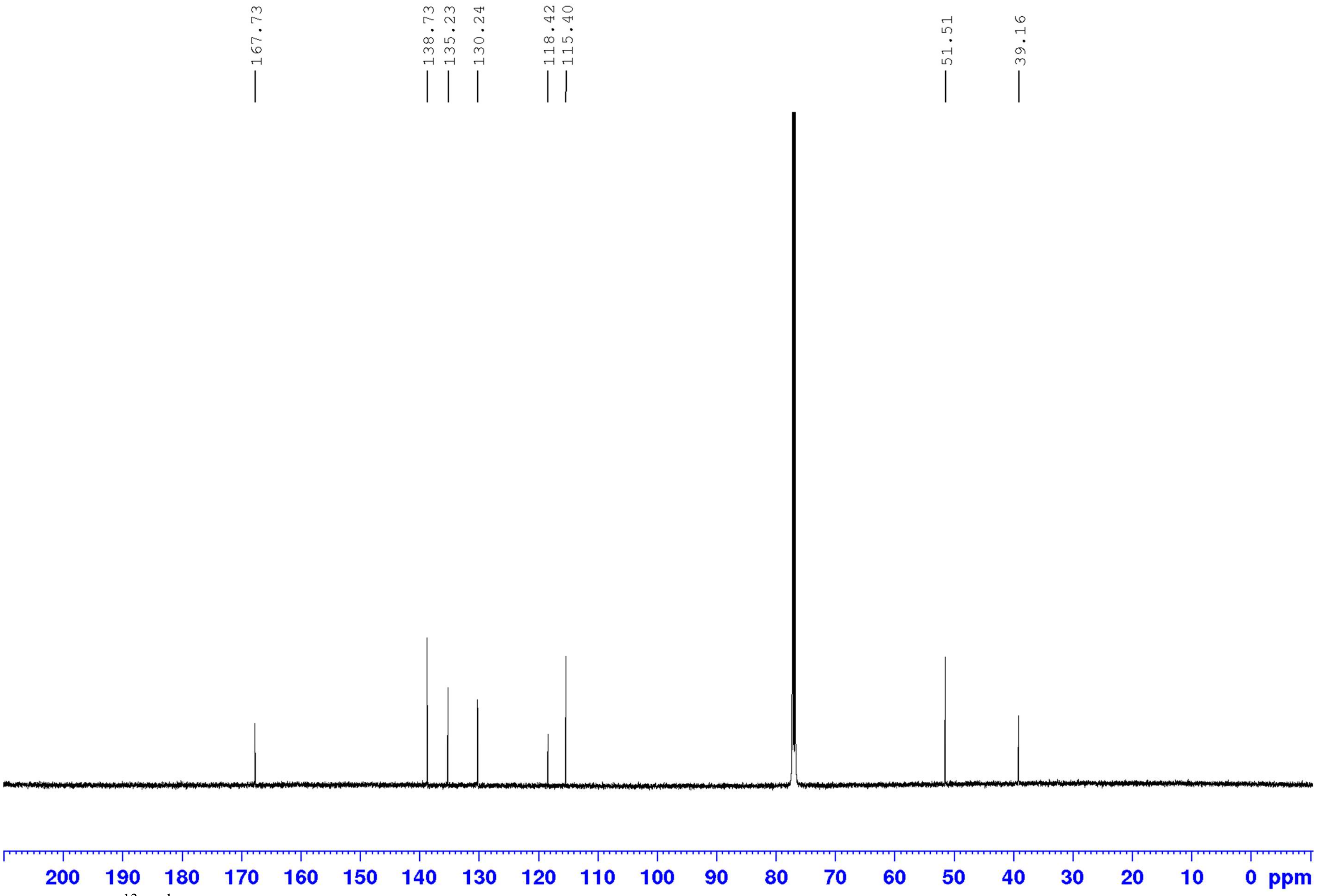

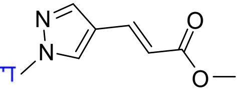

S1d

Figure S10. $^{13}C\{^{1}H\}$ NMR (126 MHz, $CDCl_3$) of **S1d**

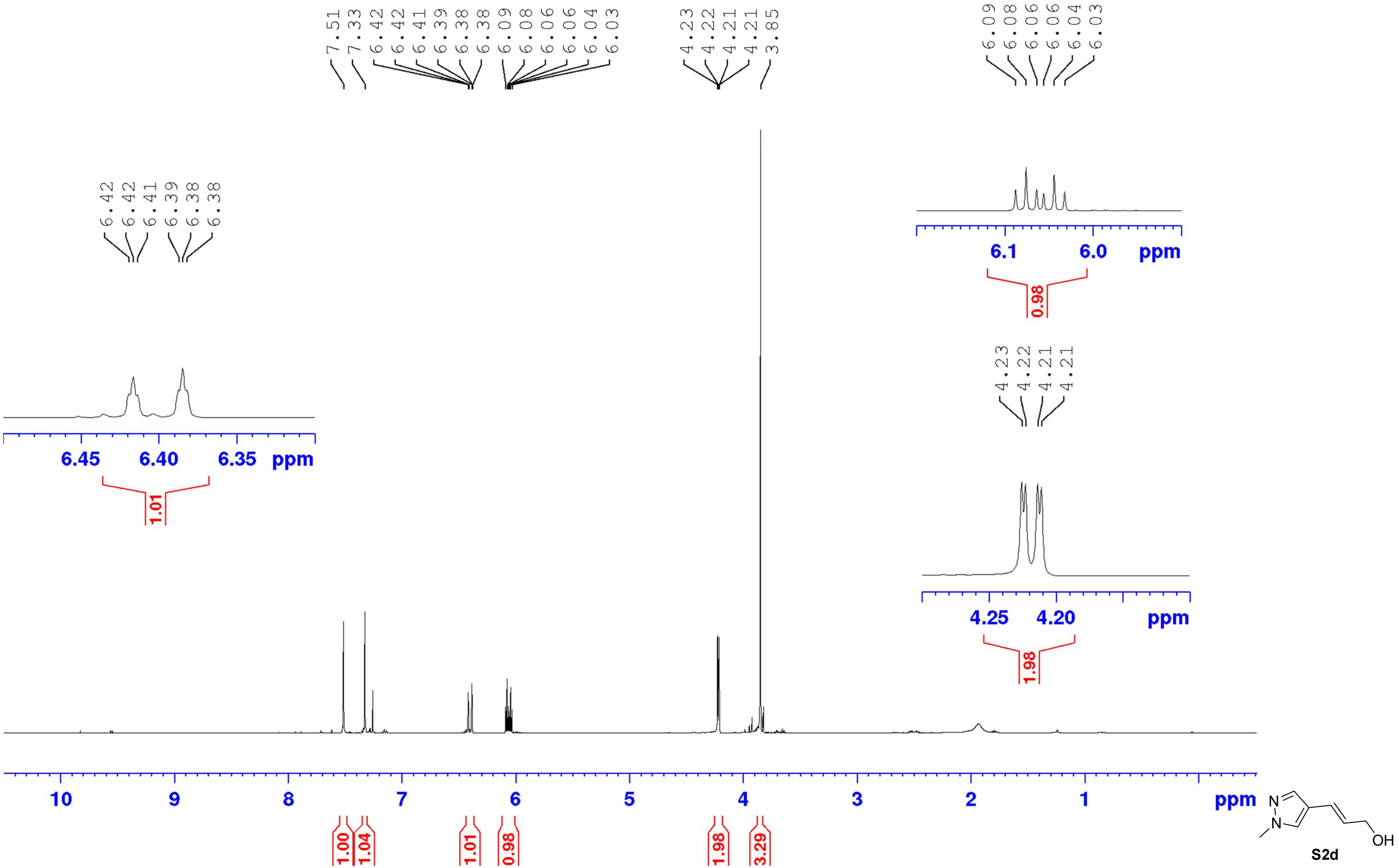

Figure S11. ^{1}H NMR (500 MHz, $CDCl_3$) of **S2d**

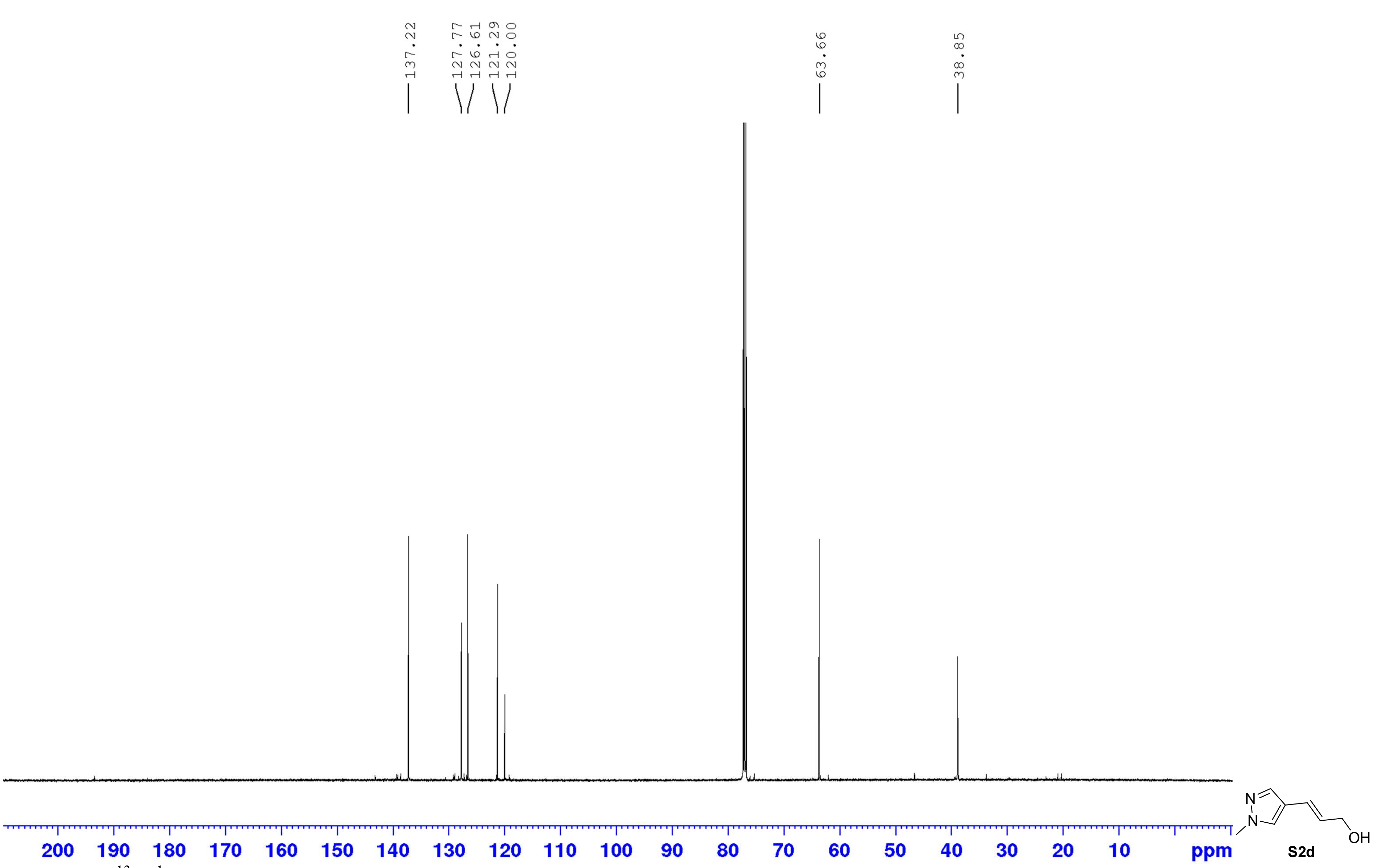

Figure S12. $^{13}C\{^{1}H\}$ NMR (126 MHz, $CDCl_3$) of **S2d**

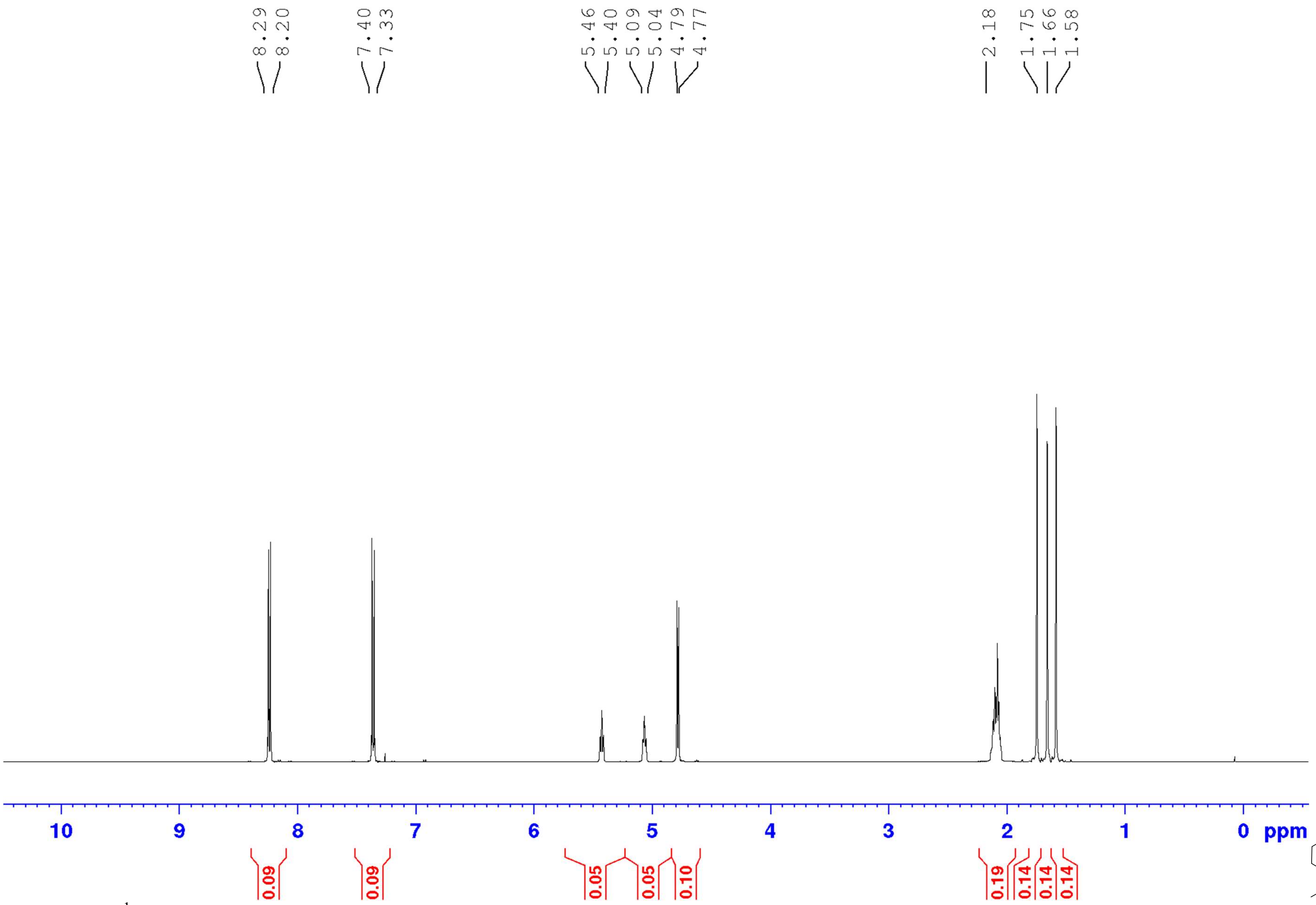

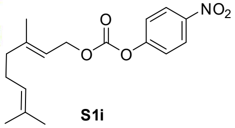

S1i

Figure S13. ^{1}H NMR (500 MHz, $CDCl_3$) of **S1i**

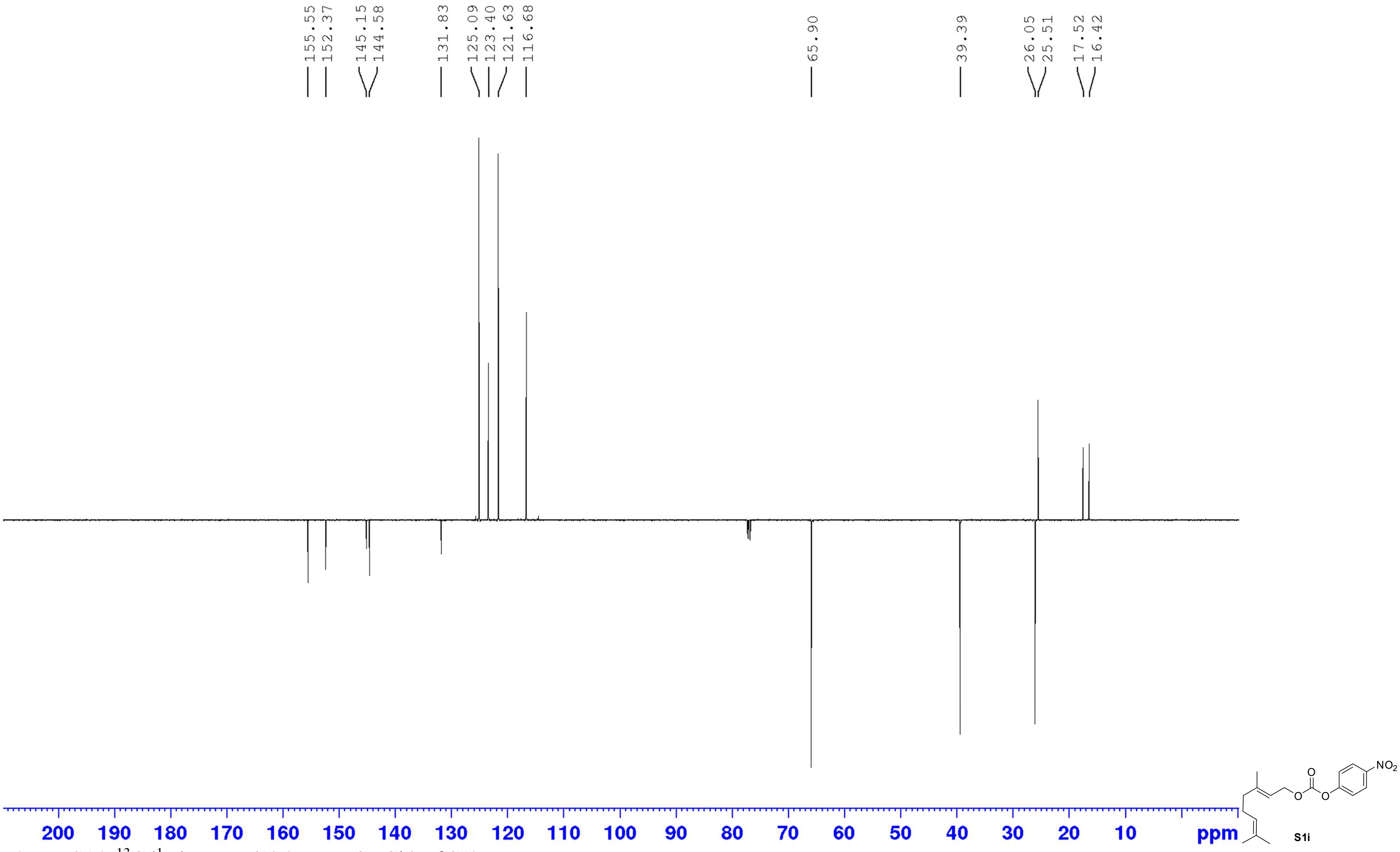

Figure S14. $^{13}C\{^{1}H\}$ NMR (126 MHz, $CDCl_3$) of **S1i**

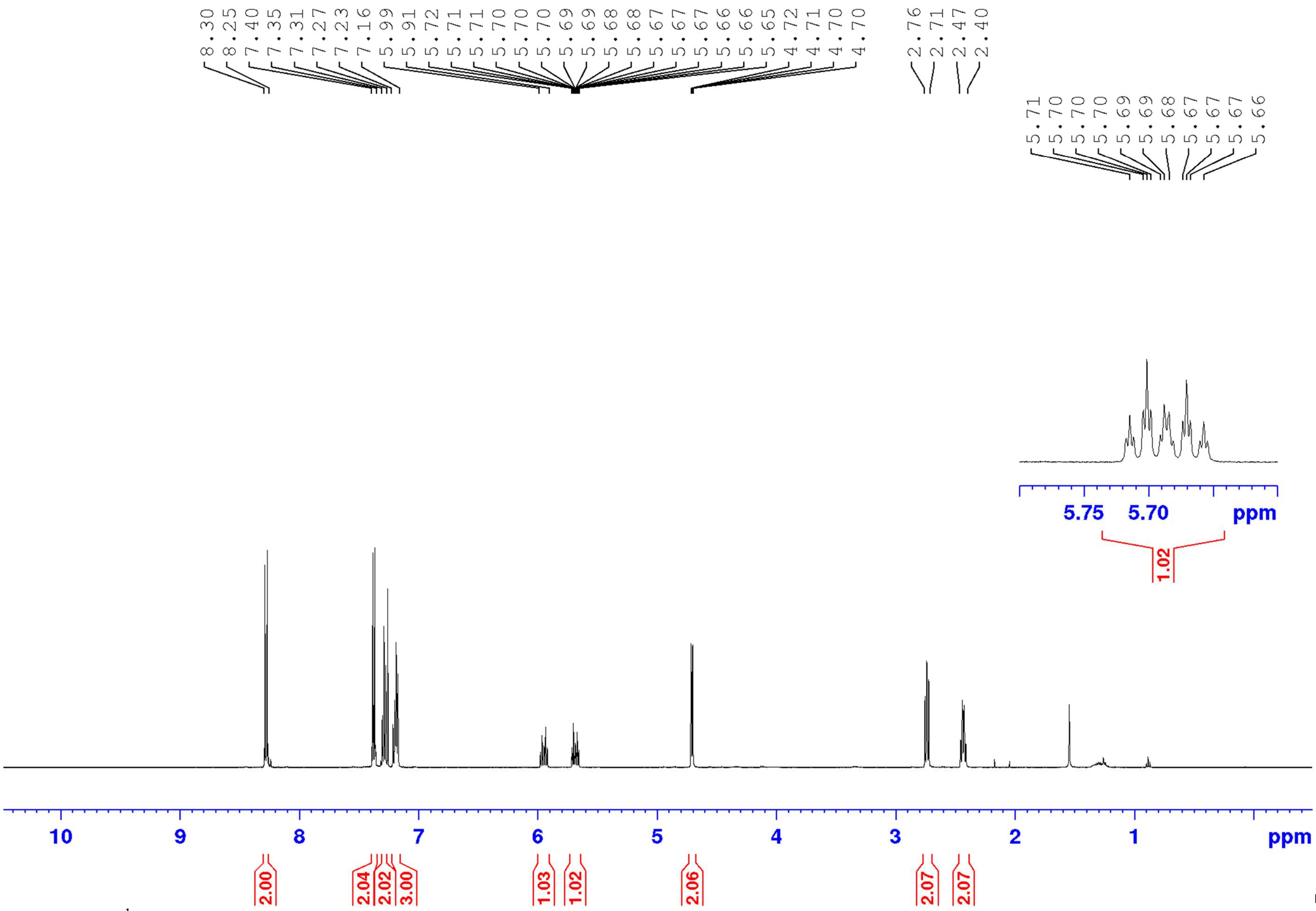

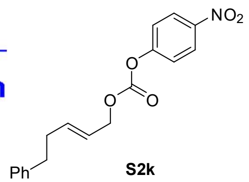
S2k

Figure S15. ^{1}H NMR (500 MHz, $CDCl_3$) of **S2k**

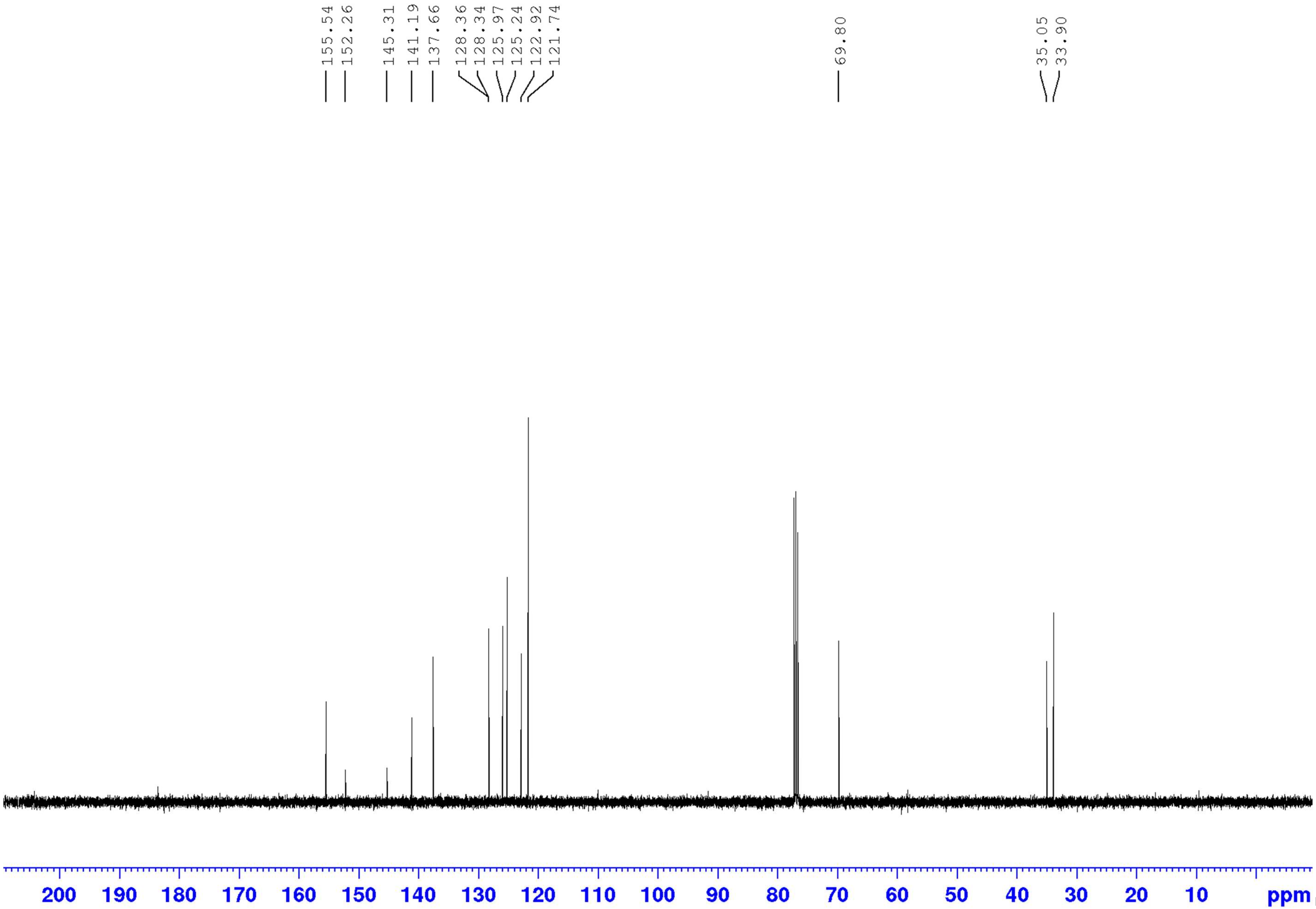

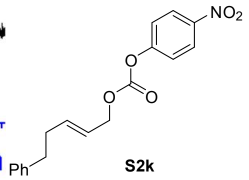

S2k

Figure S16. $^{13}C\{^{1}H\}$ NMR (126 MHz, $CDCl_3$) of **S2k**

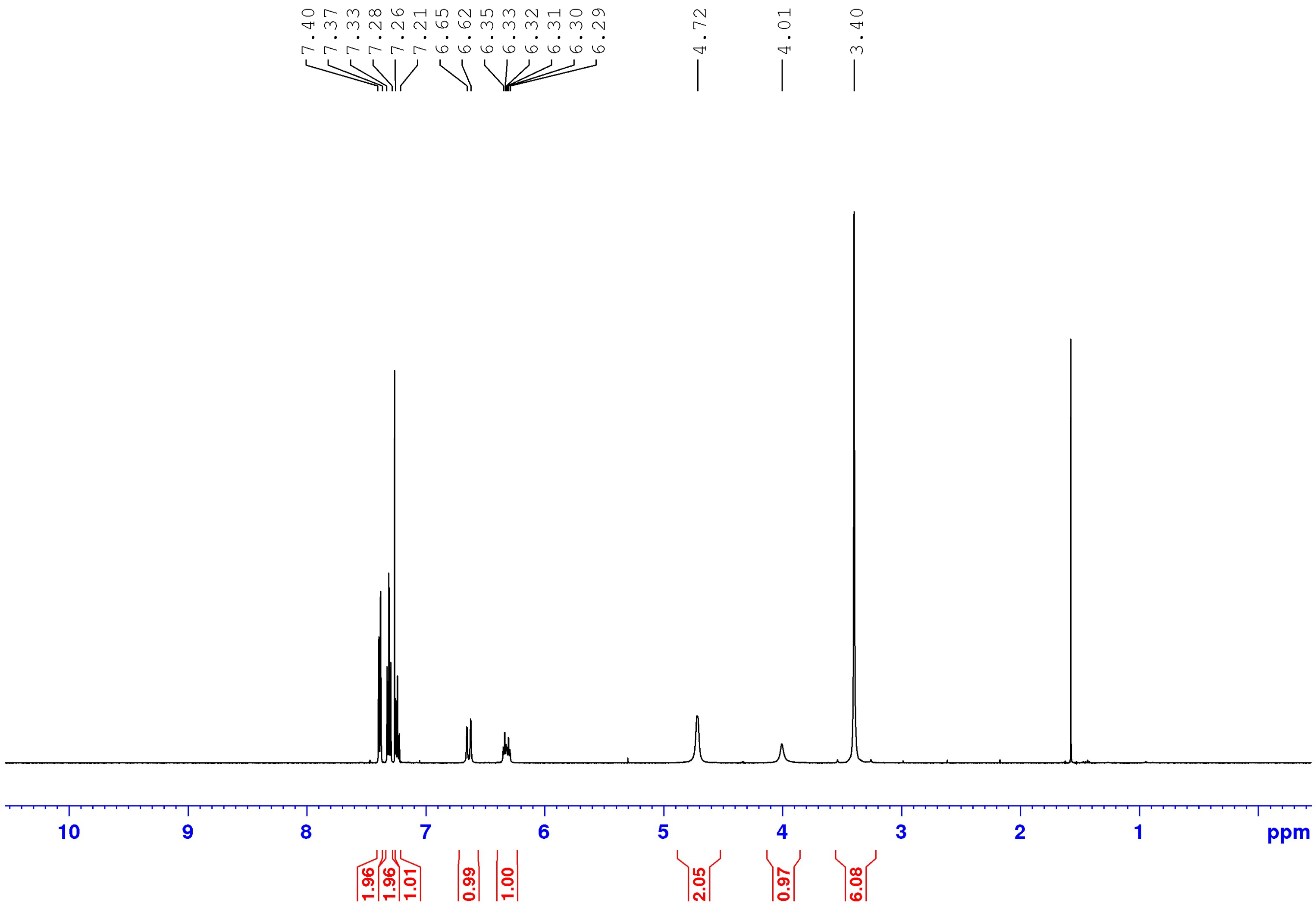

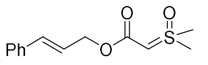

1a

Figure S17. ^{1}H NMR (500 MHz, $CDCl_3$) of **1a**

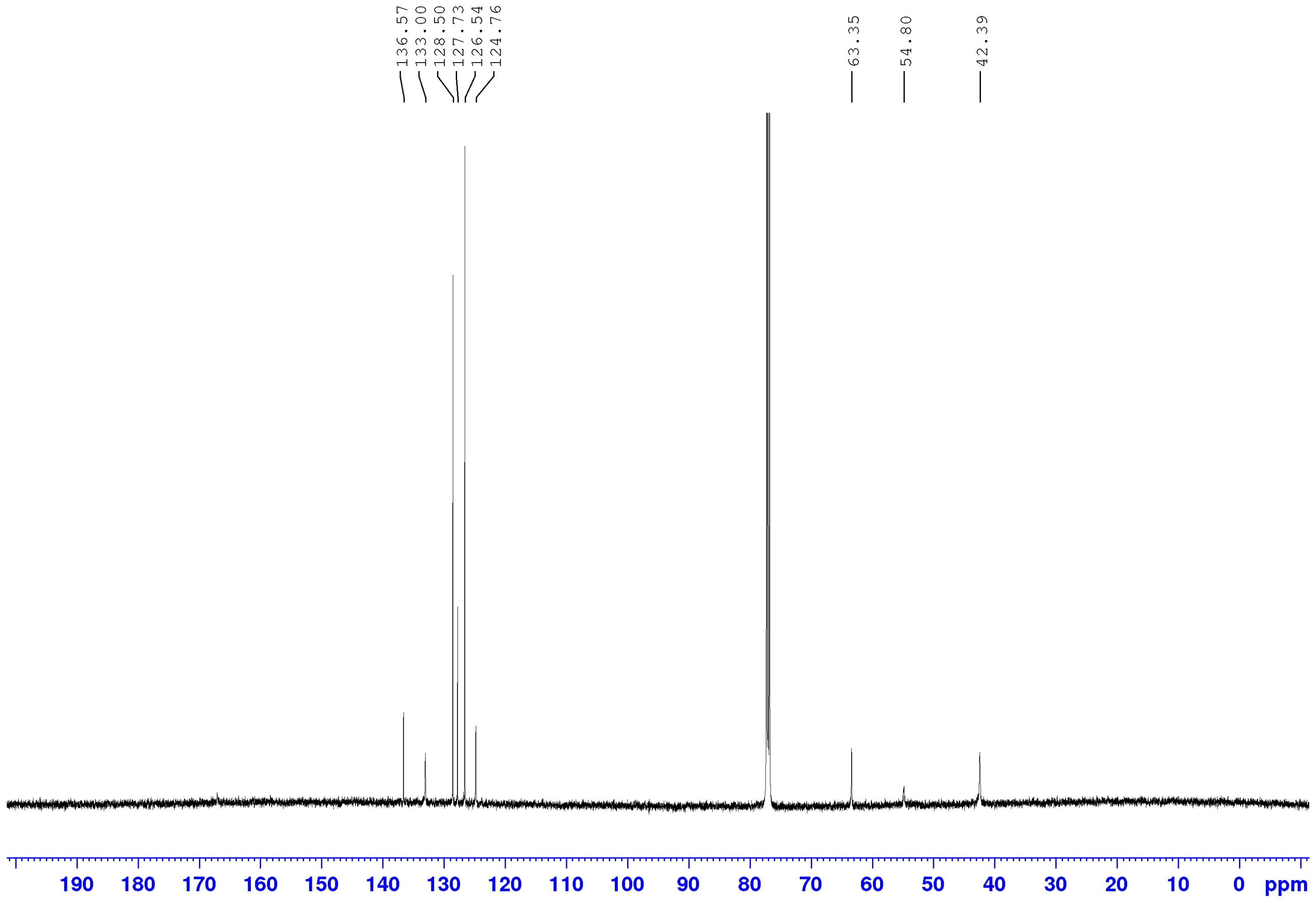

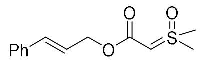

1a

Figure S18. $^{13}C\{^{1}H\}$ NMR (126 MHz, $CDCl_3$) of **1a**

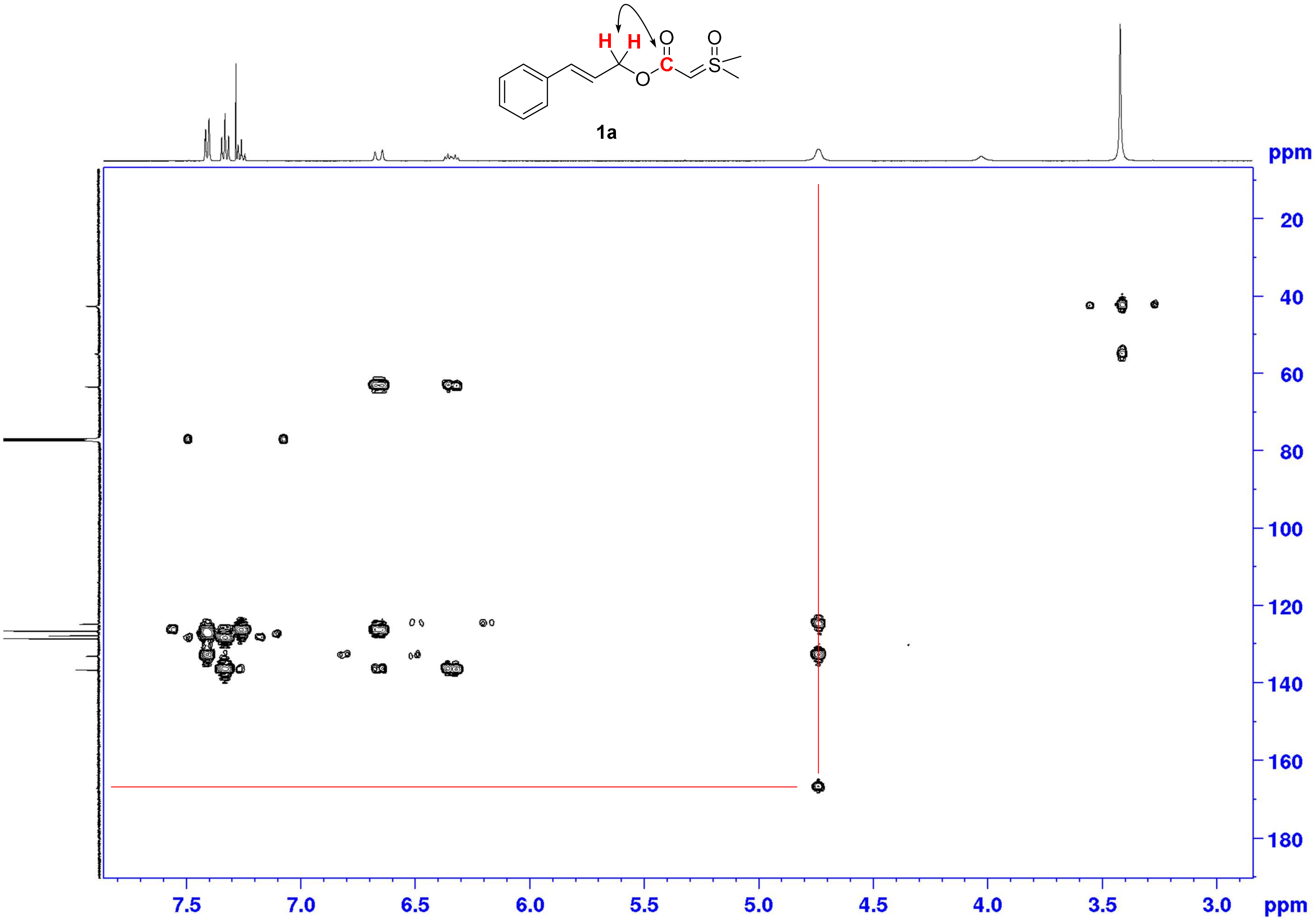

Figure S19. HMBC of **1a**

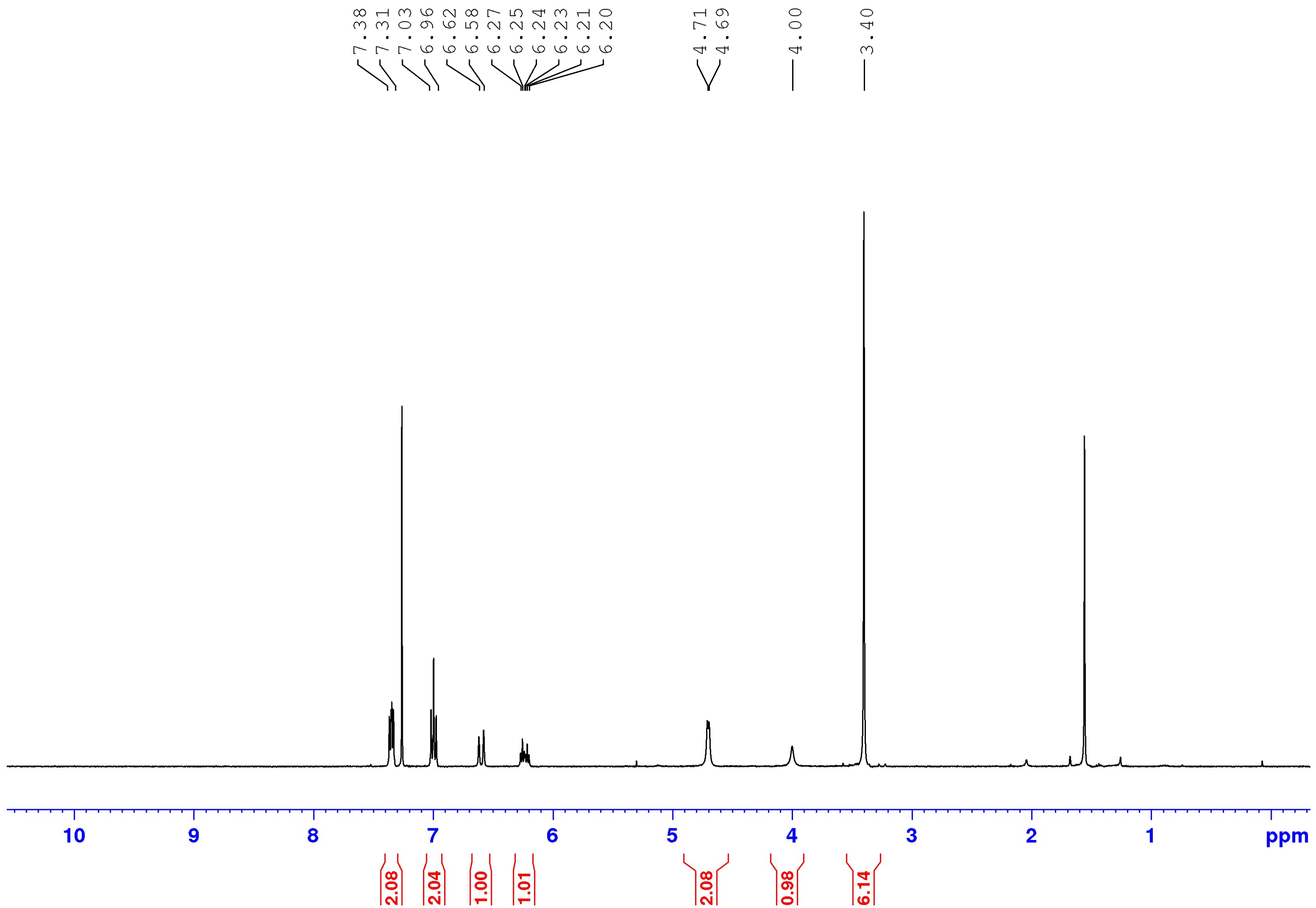

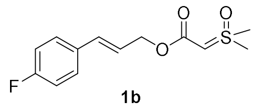

1b

Figure S20. ^{1}H NMR (500 MHz, $CDCl_3$) of **1b**

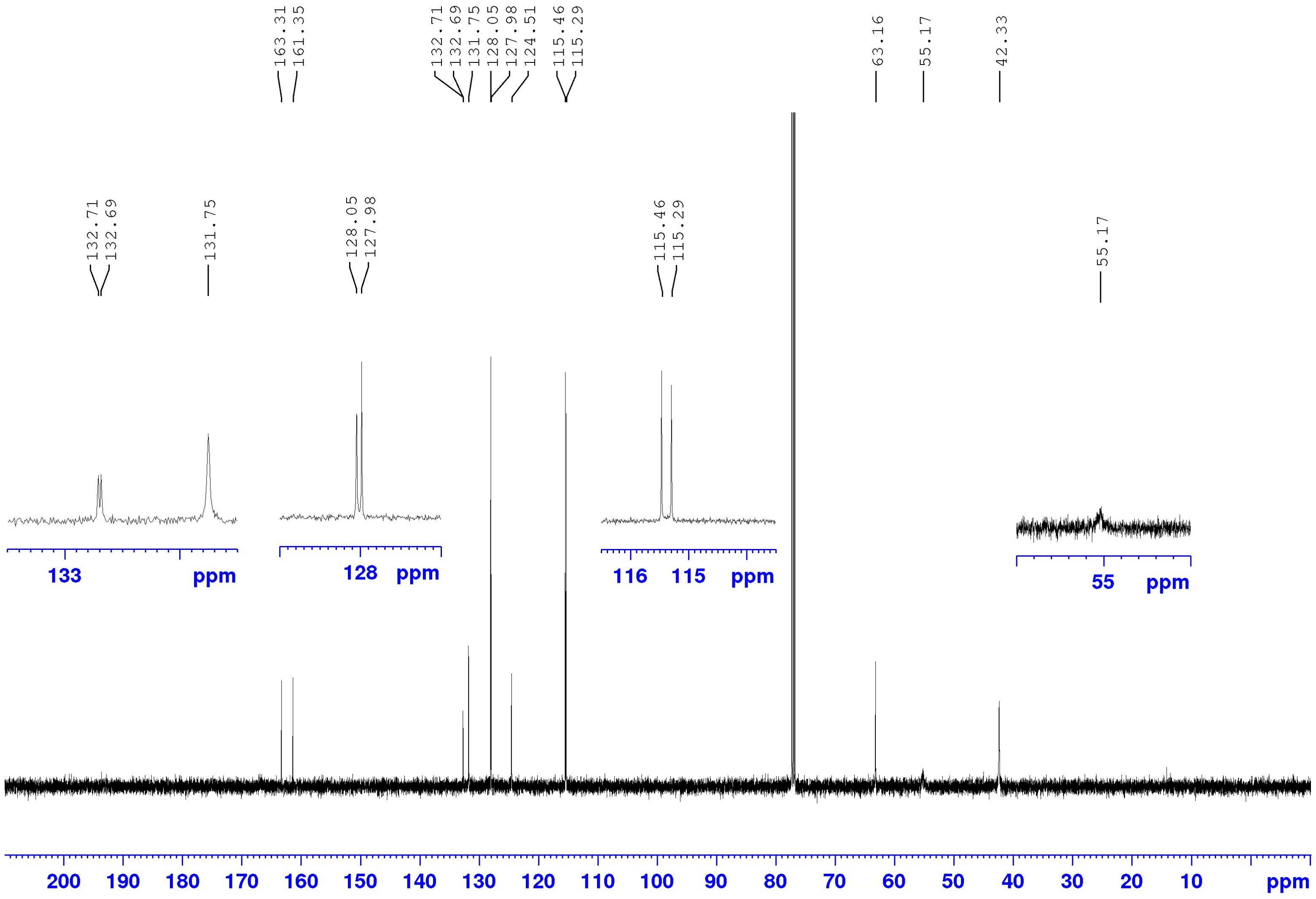

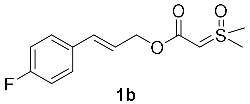

1b

Figure S21. $^{13}C\{^{1}H\}$ NMR (126 MHz, $CDCl_3$) of **1b**

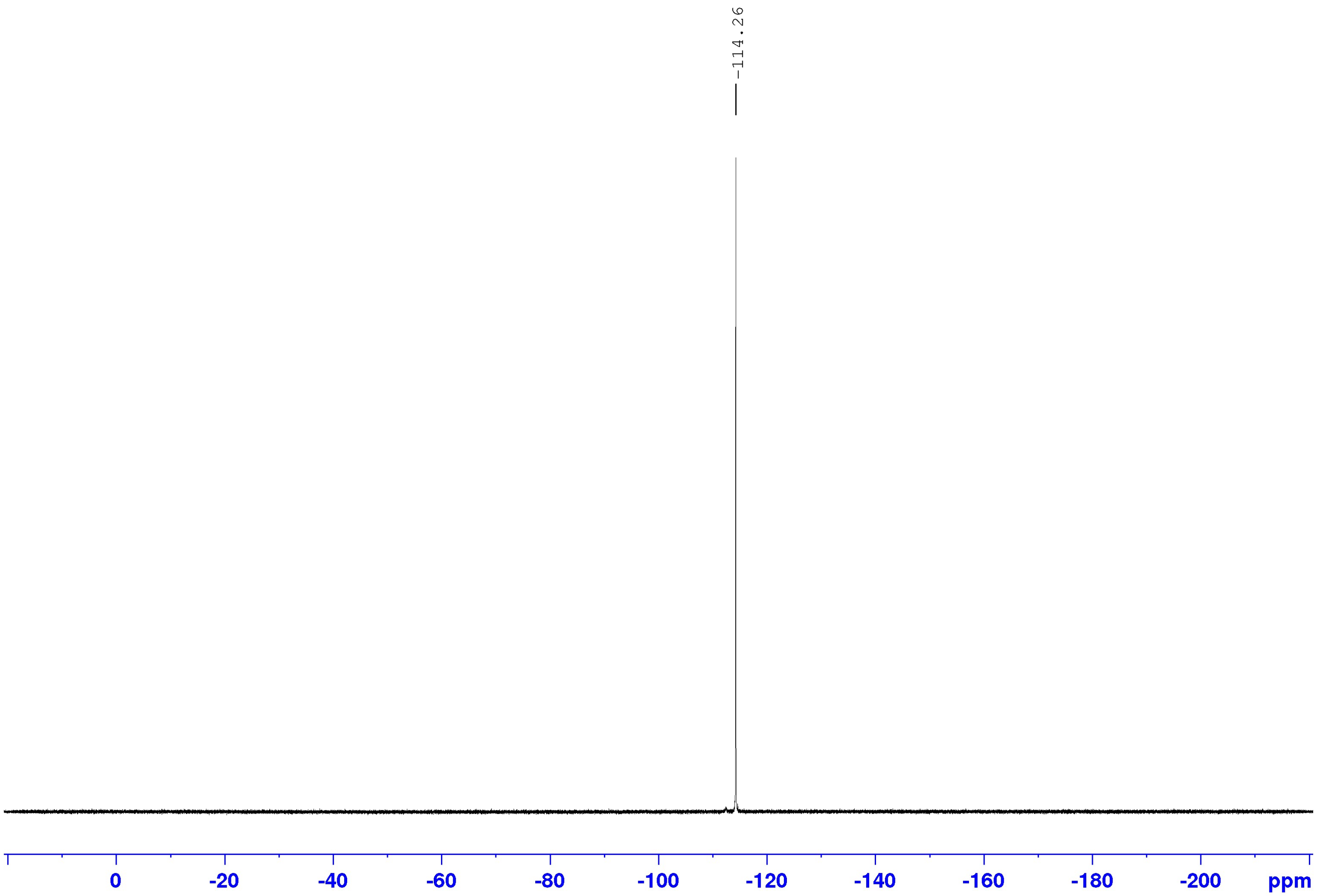

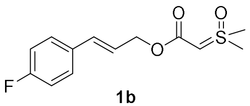

1b

Figure S22. ^{19}F NMR (470MHz, $CDCl_3$) of **1b**

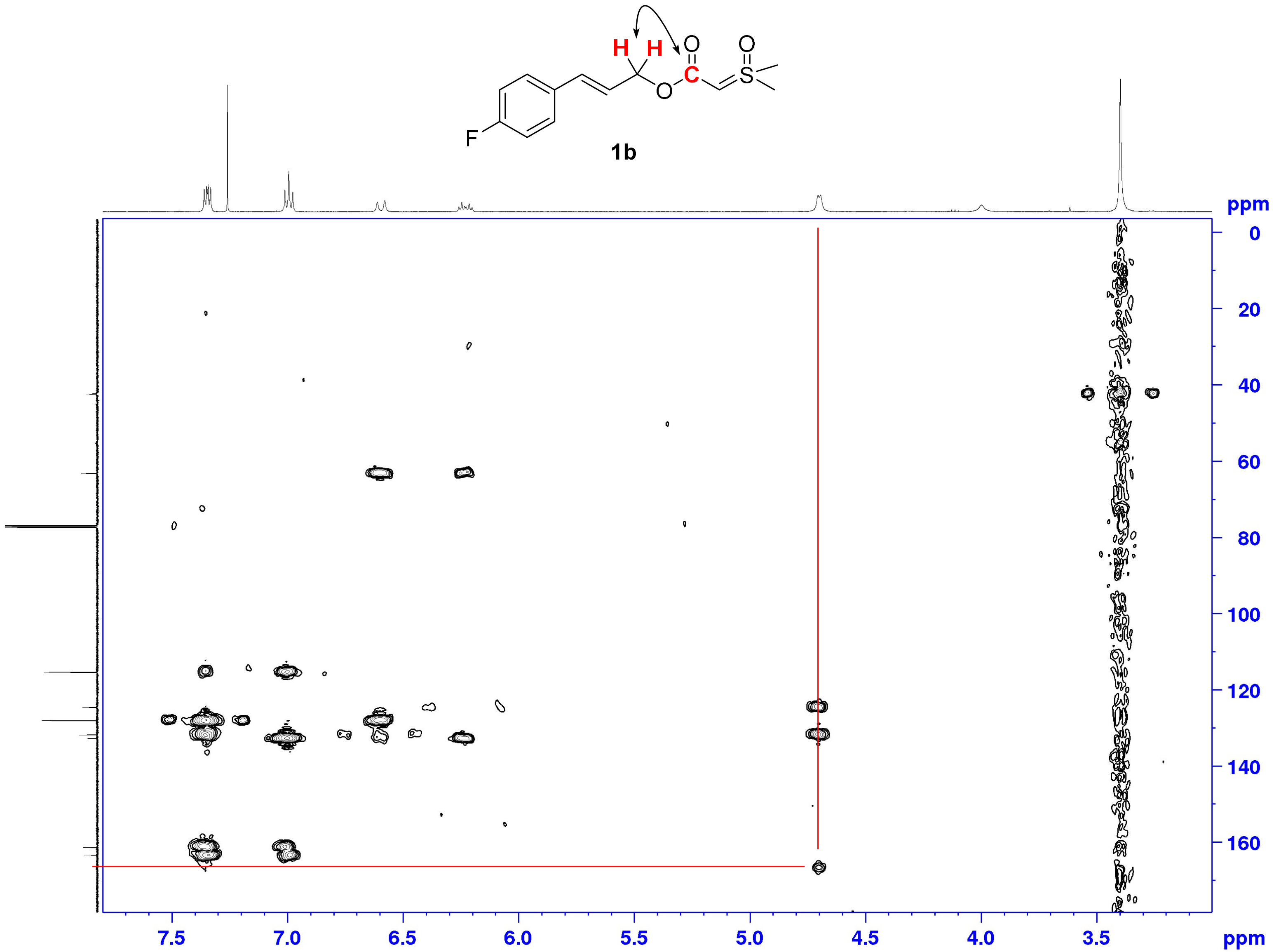

Figure S23. HMBC of **1b**

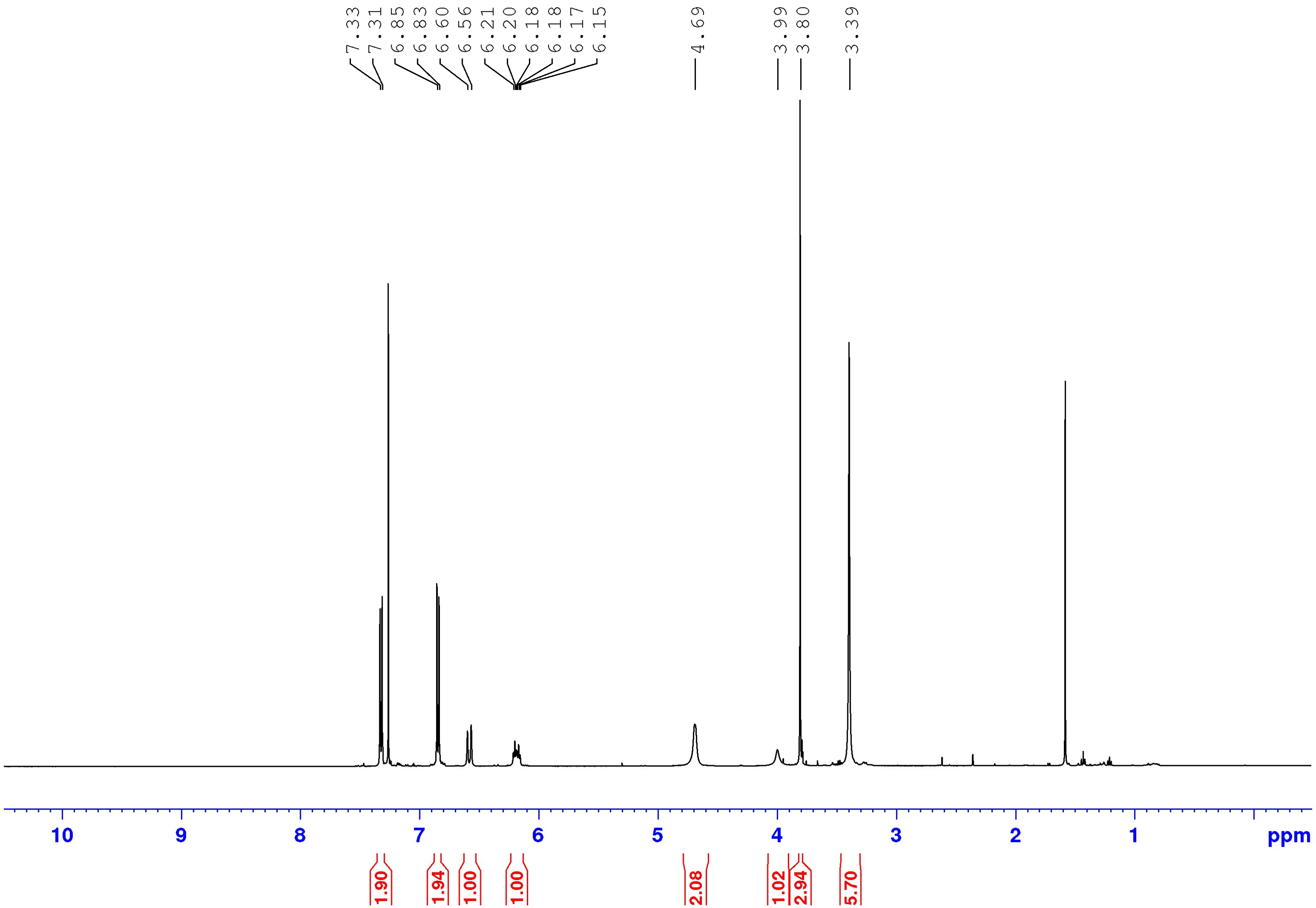

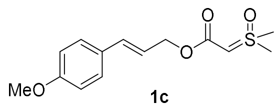

1c

Figure S24. ^{1}H NMR (500 MHz, $CDCl_3$) of **1c**

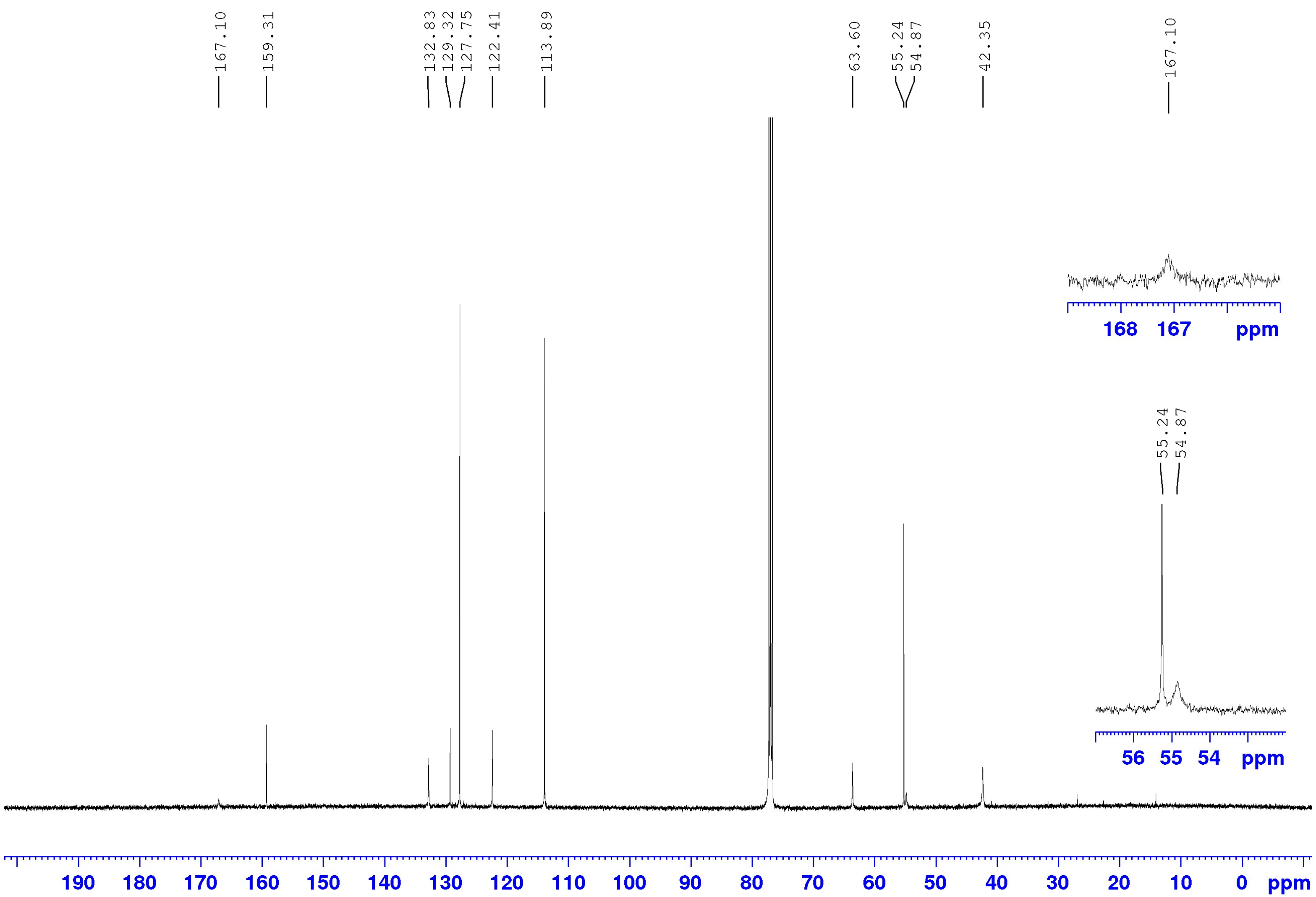

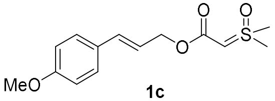

1c

Figure S25. $^{13}C\{^{1}H\}$ NMR (126 MHz, $CDCl_3$) of **1c**

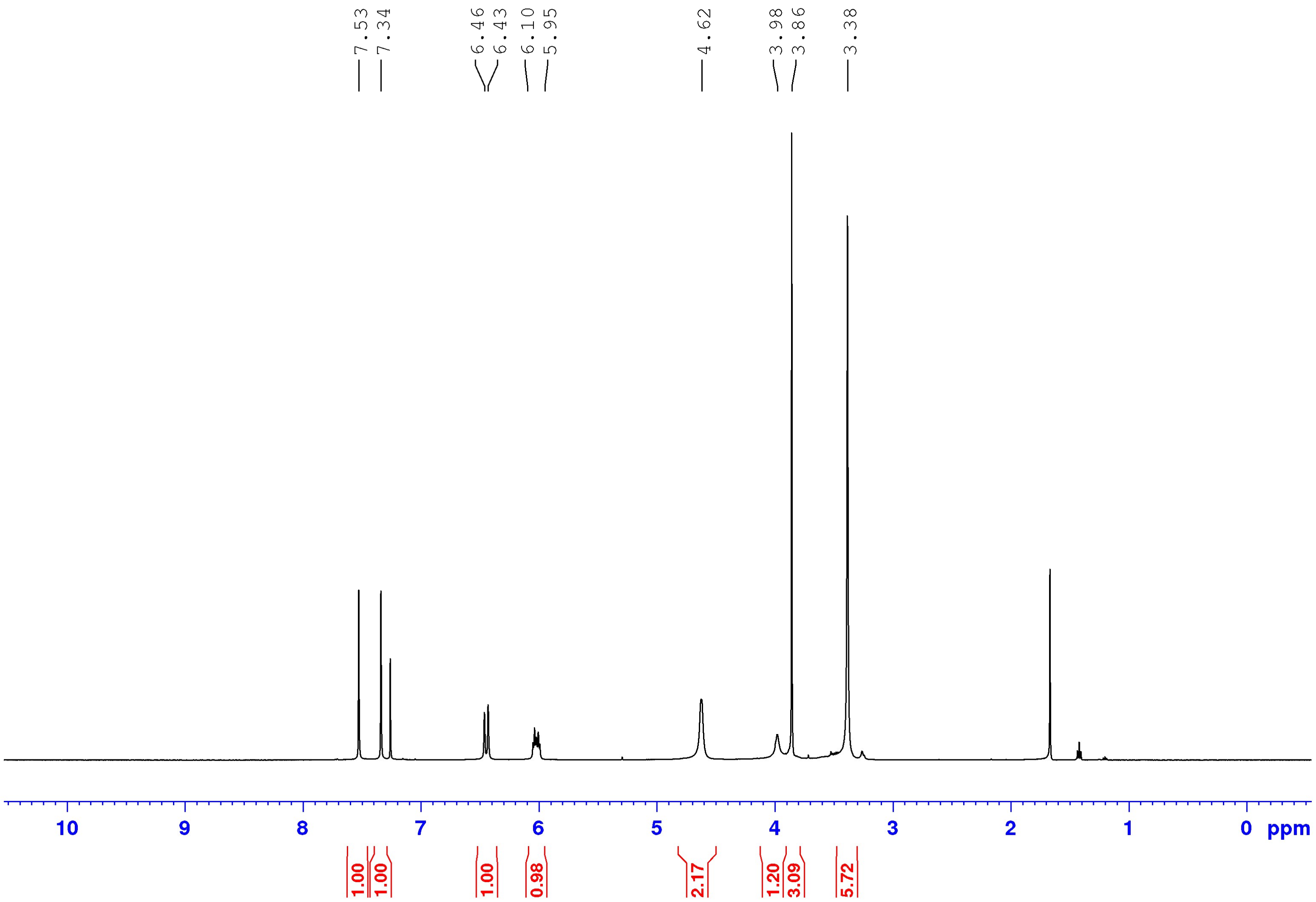

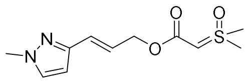

1d

Figure S26. ^{1}H NMR (500 MHz, $CDCl_3$) of **1d**

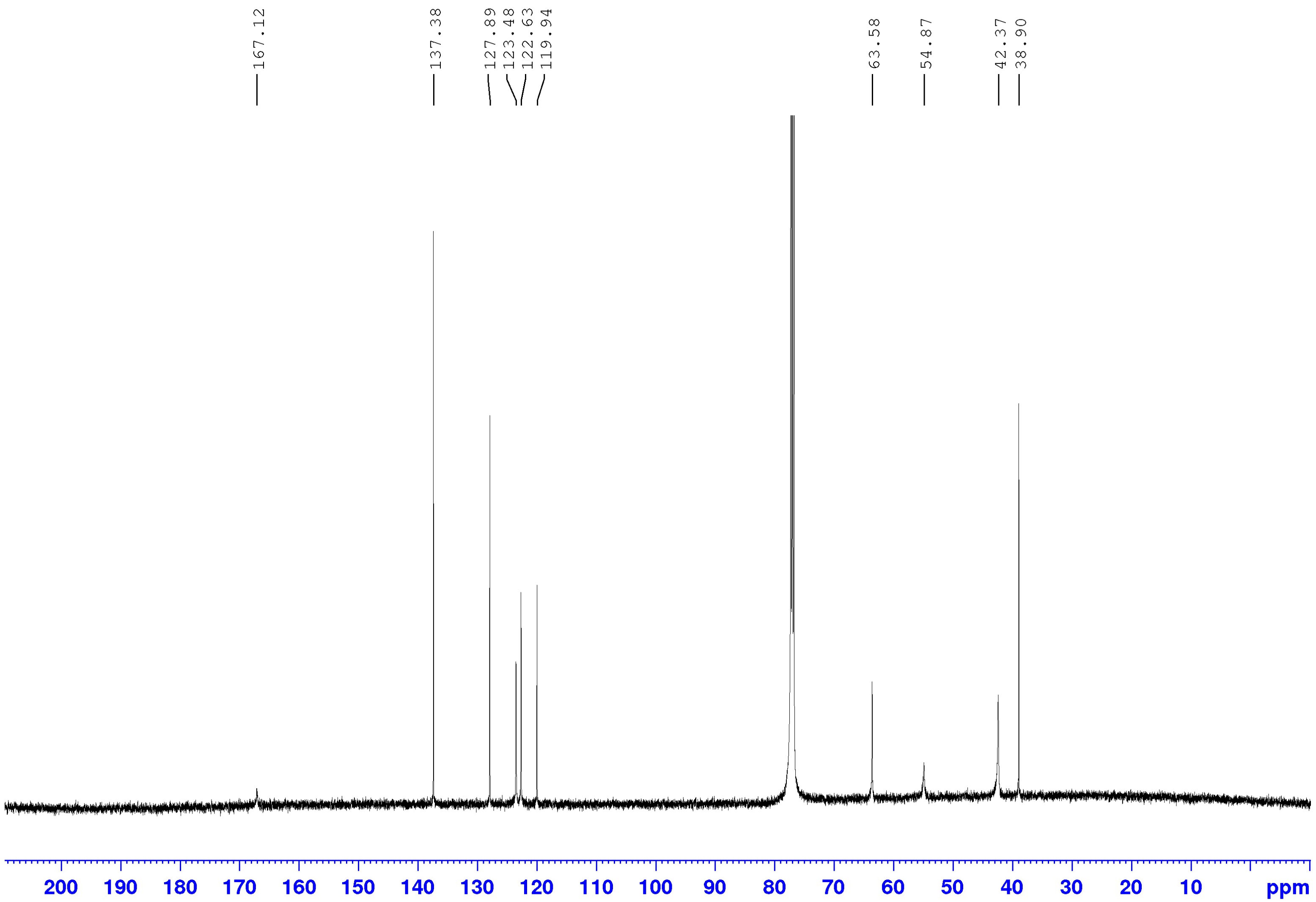

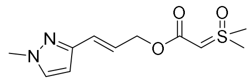

1d

Figure S27. $^{13}C\{^1H\}$ NMR (126 MHz, $CDCl_3$) of **1d**

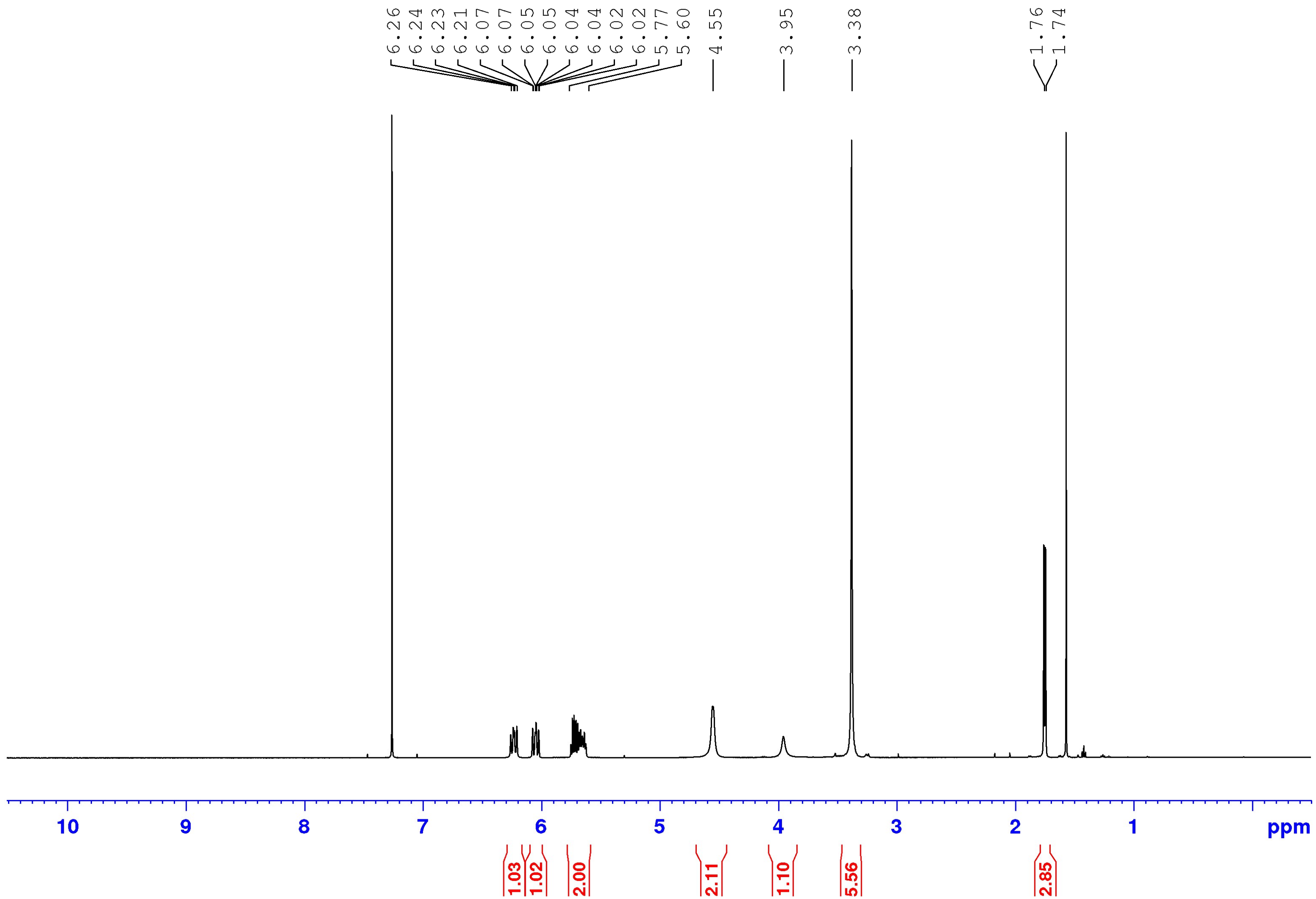

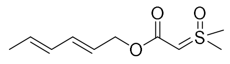

1e

Figure S28. ^{1}H NMR (500 MHz, $CDCl_3$) of **1e**

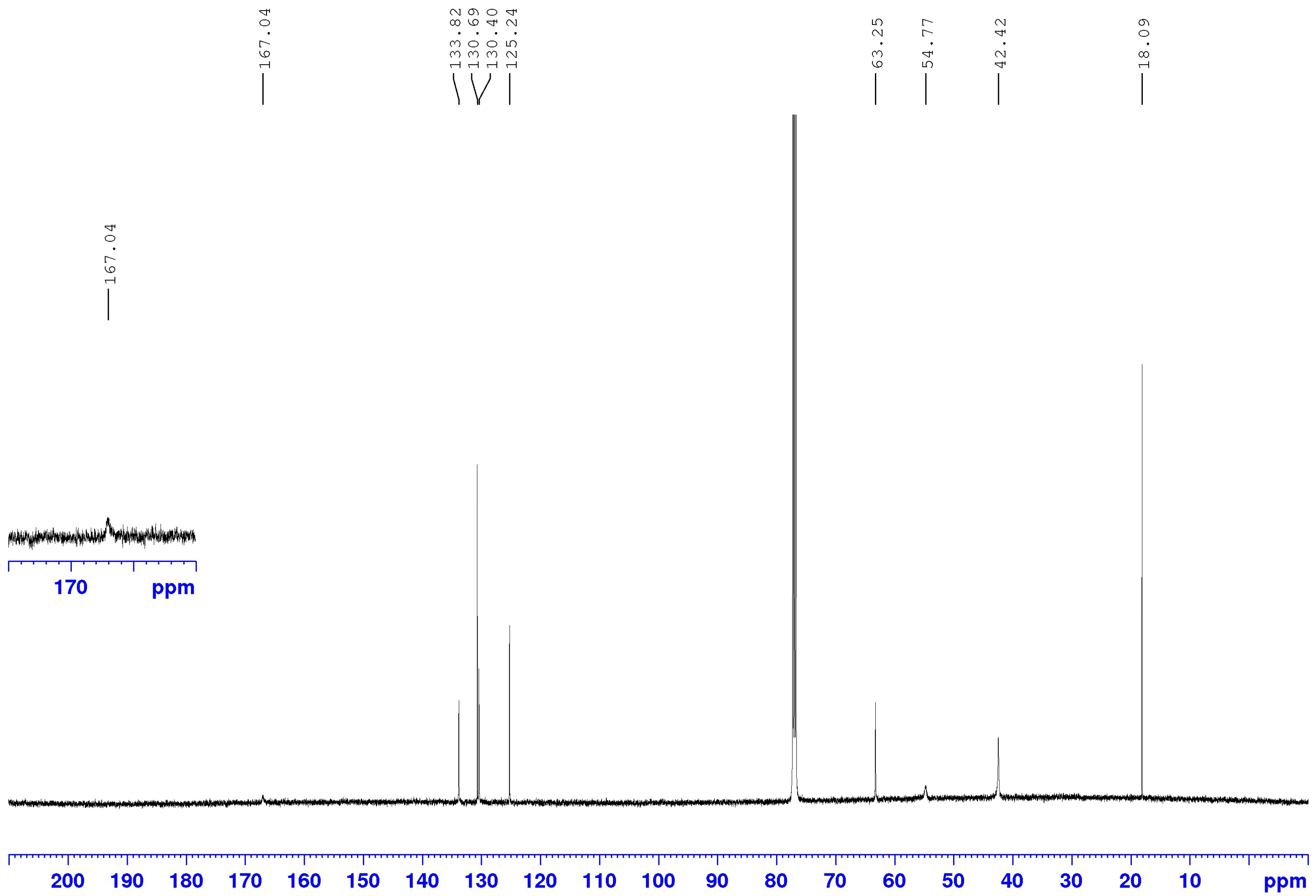

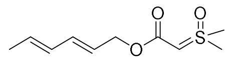

1e

Figure S29. $^{13}C\{^{1}H\}$ NMR (126 MHz, $CDCl_3$) of **1e**

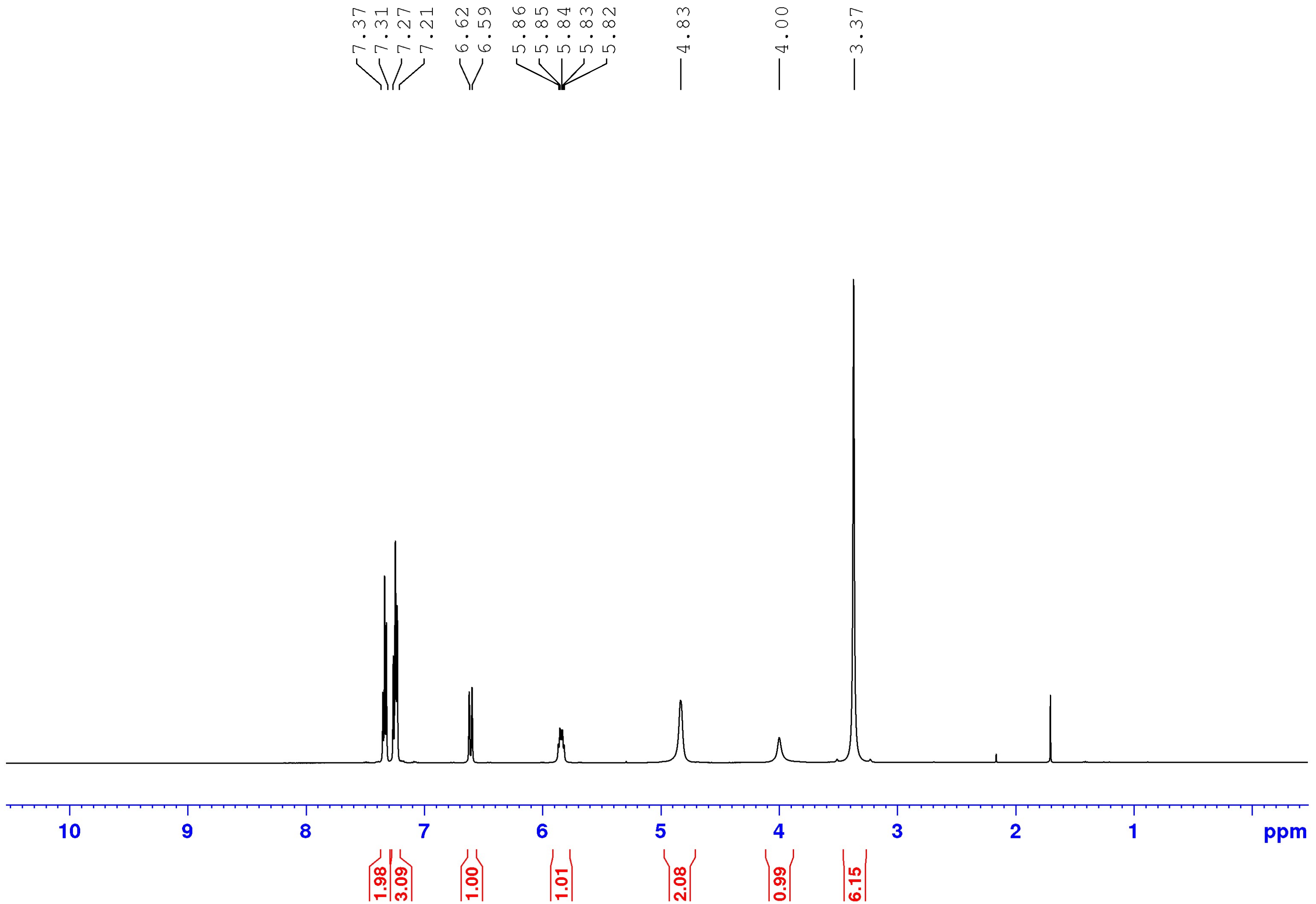

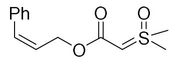

1f

Figure S30. ^{1}H NMR (500 MHz, $CDCl_3$) of **1f**

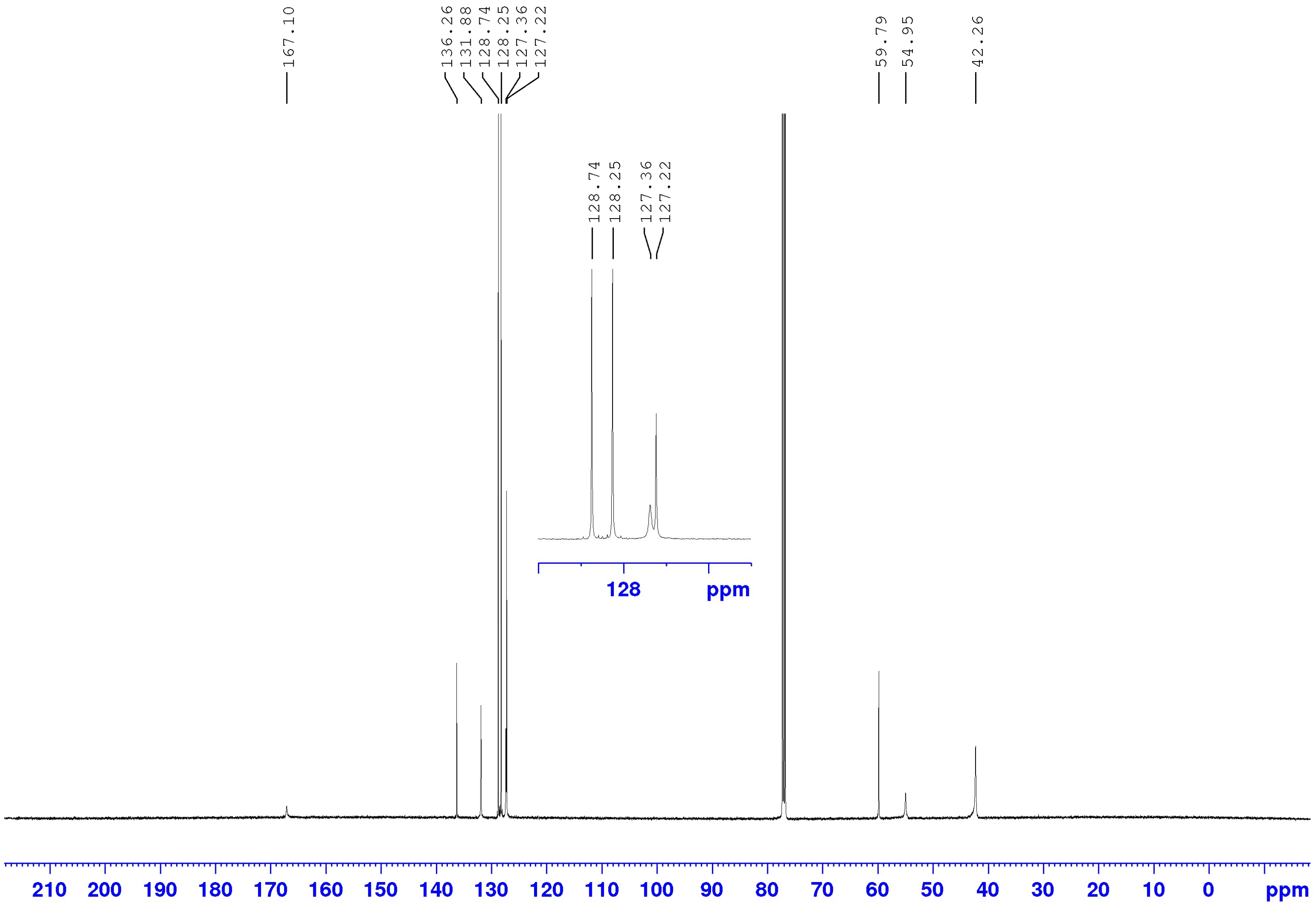

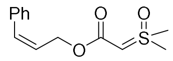

1f

Figure S31. $^{13}C\{^{1}H\}$ NMR (126 MHz, $CDCl_3$) of **1f**

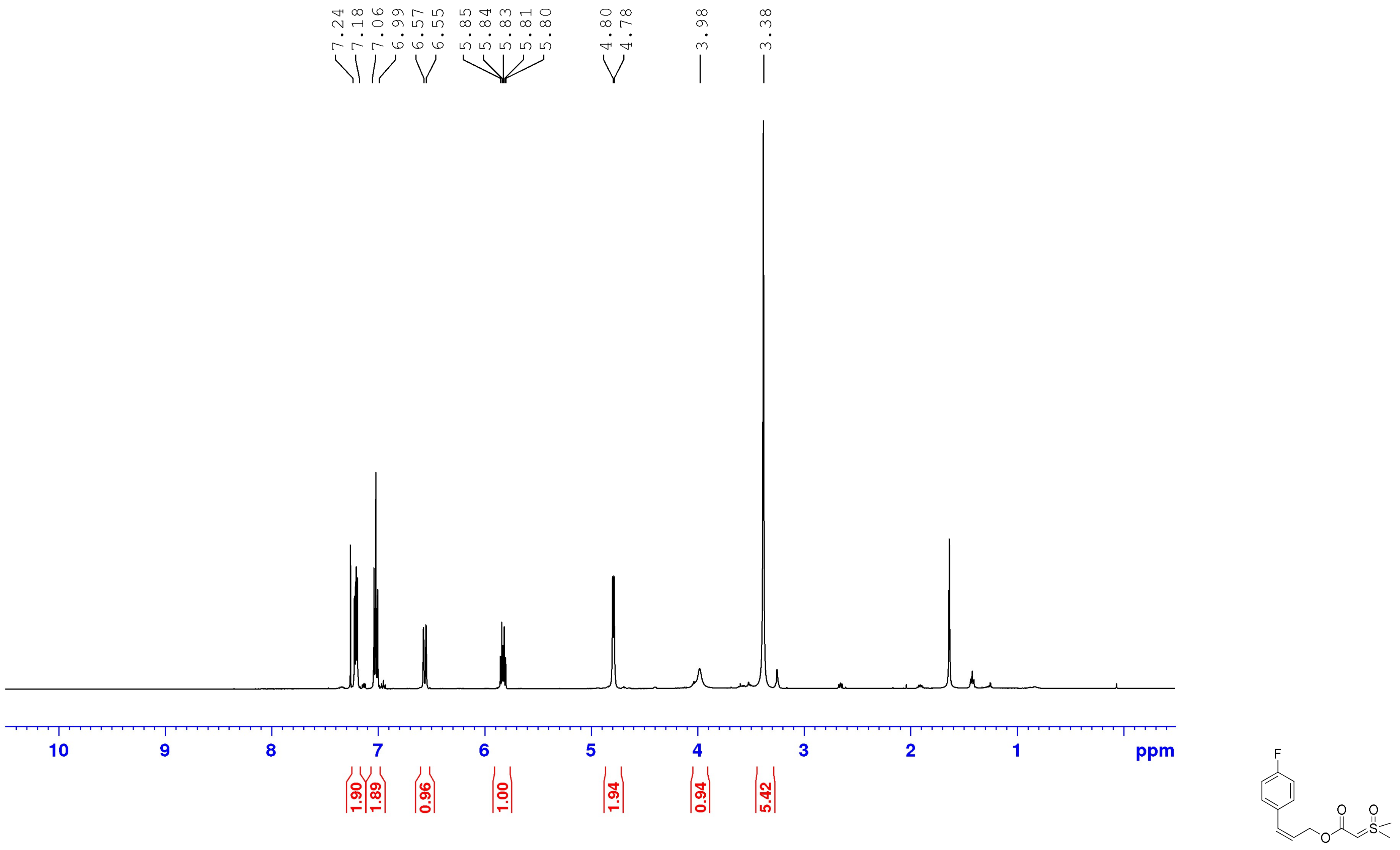

1g

Figure S32. ^{1}H NMR (500 MHz, $CDCl_3$) of **1g**

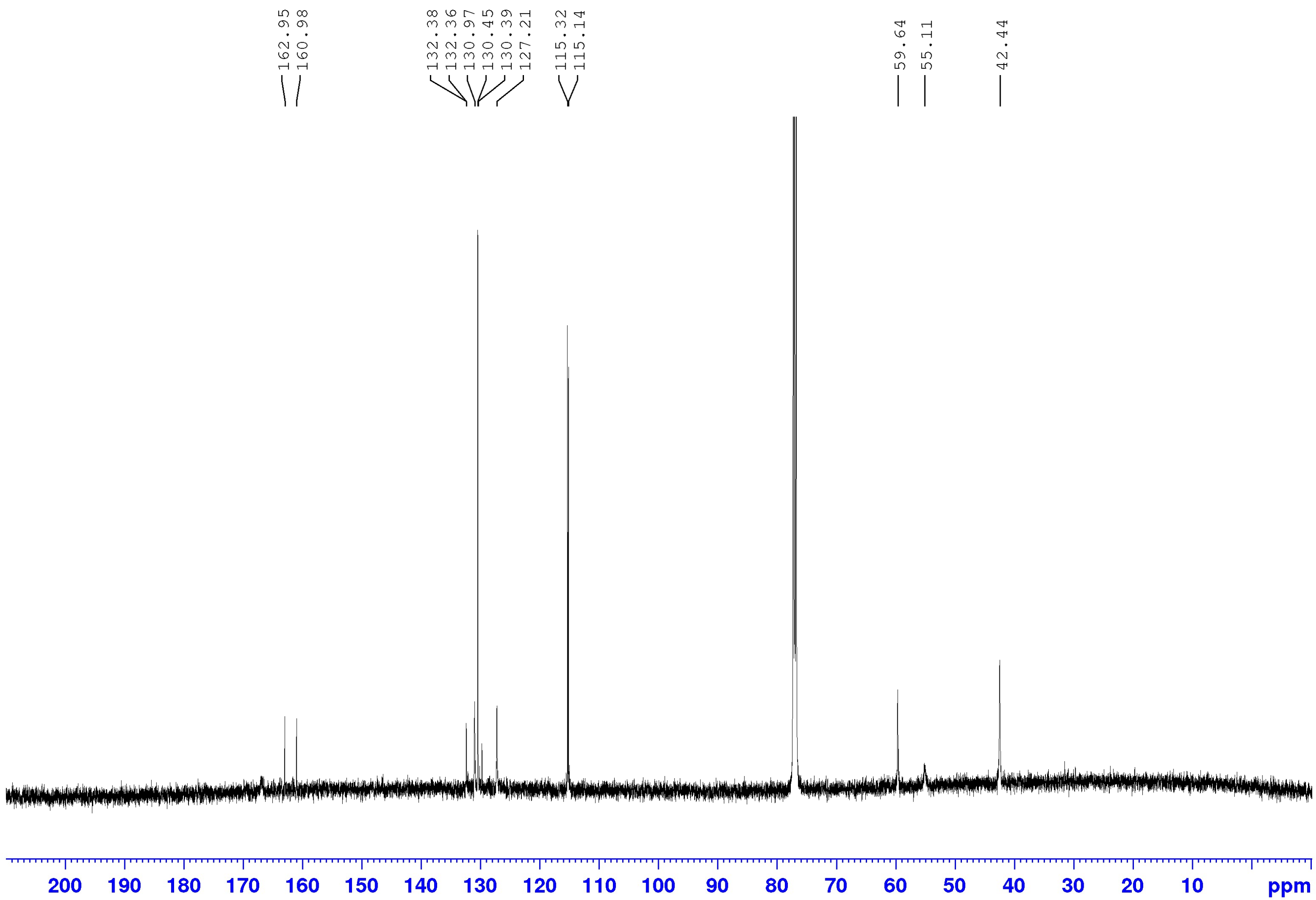

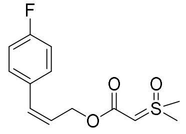

1g

Figure S33. $^{13}C\{^{1}H\}$ NMR (126 MHz, $CDCl_3$) of **1g**

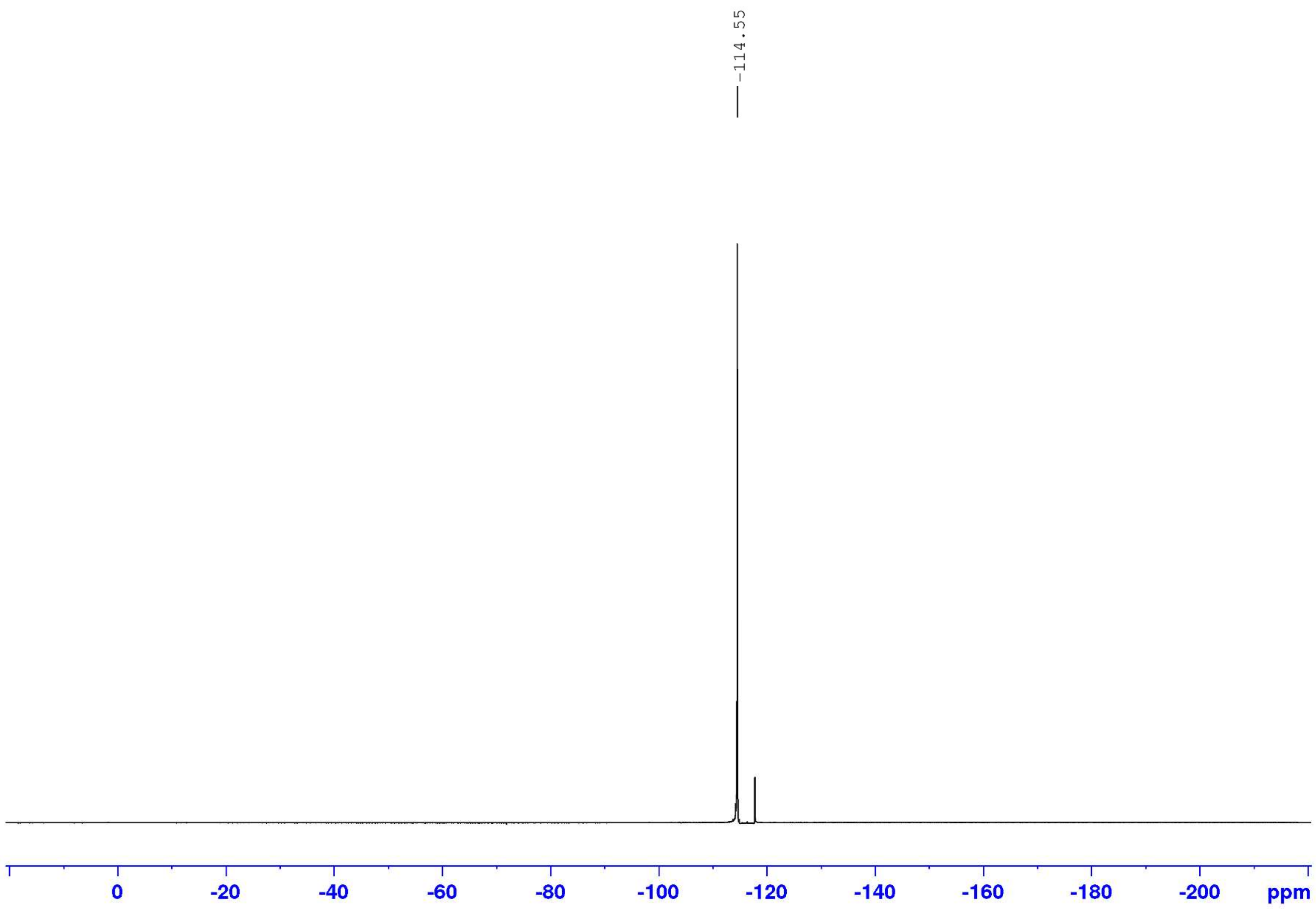

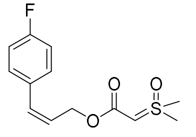

1g

Figure S34. $^{19}F\{^{1}H\}$ NMR (470 MHz, $CDCl_3$) of **1g**

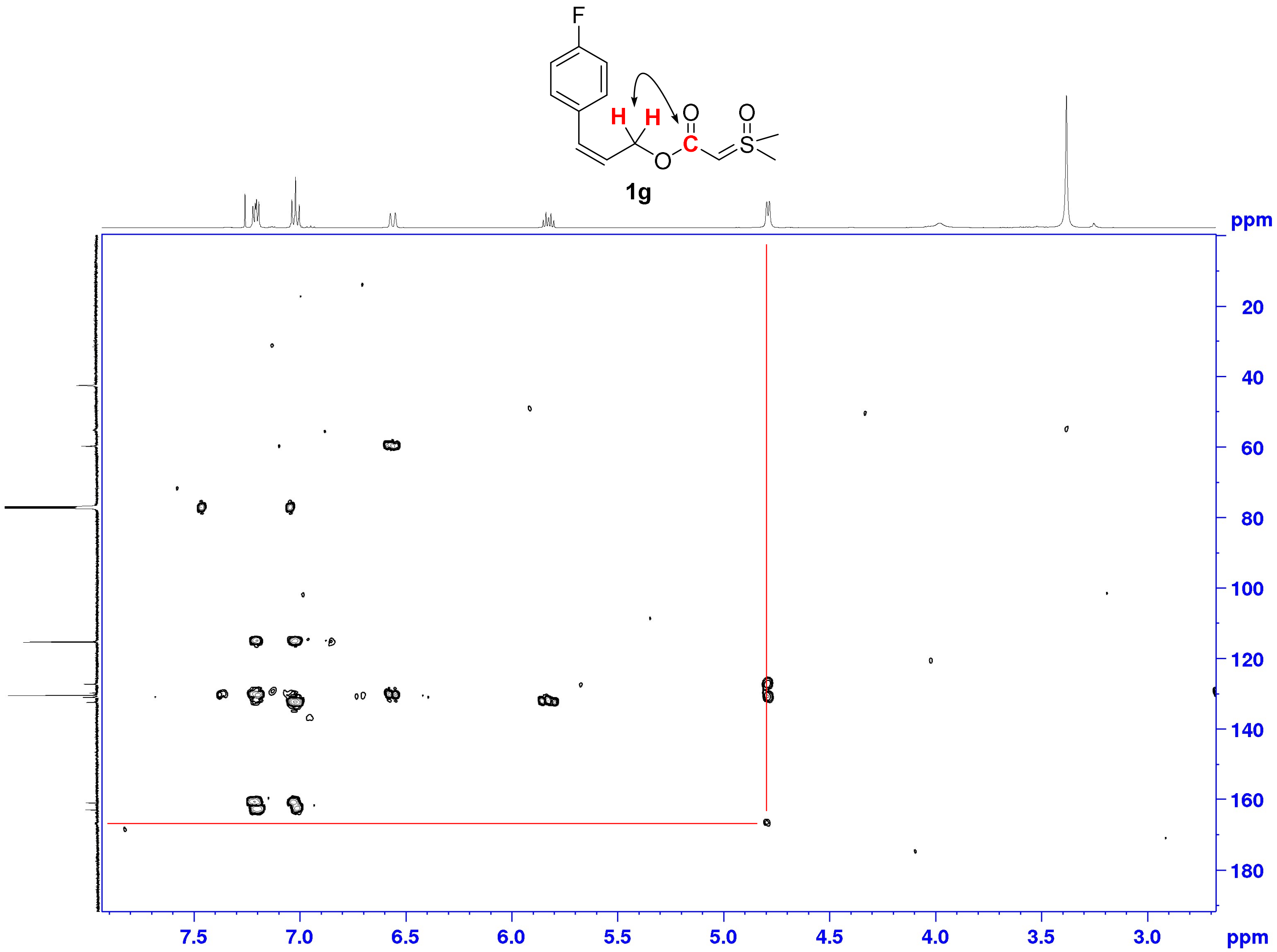

Figure S35. HMBC of **1g**

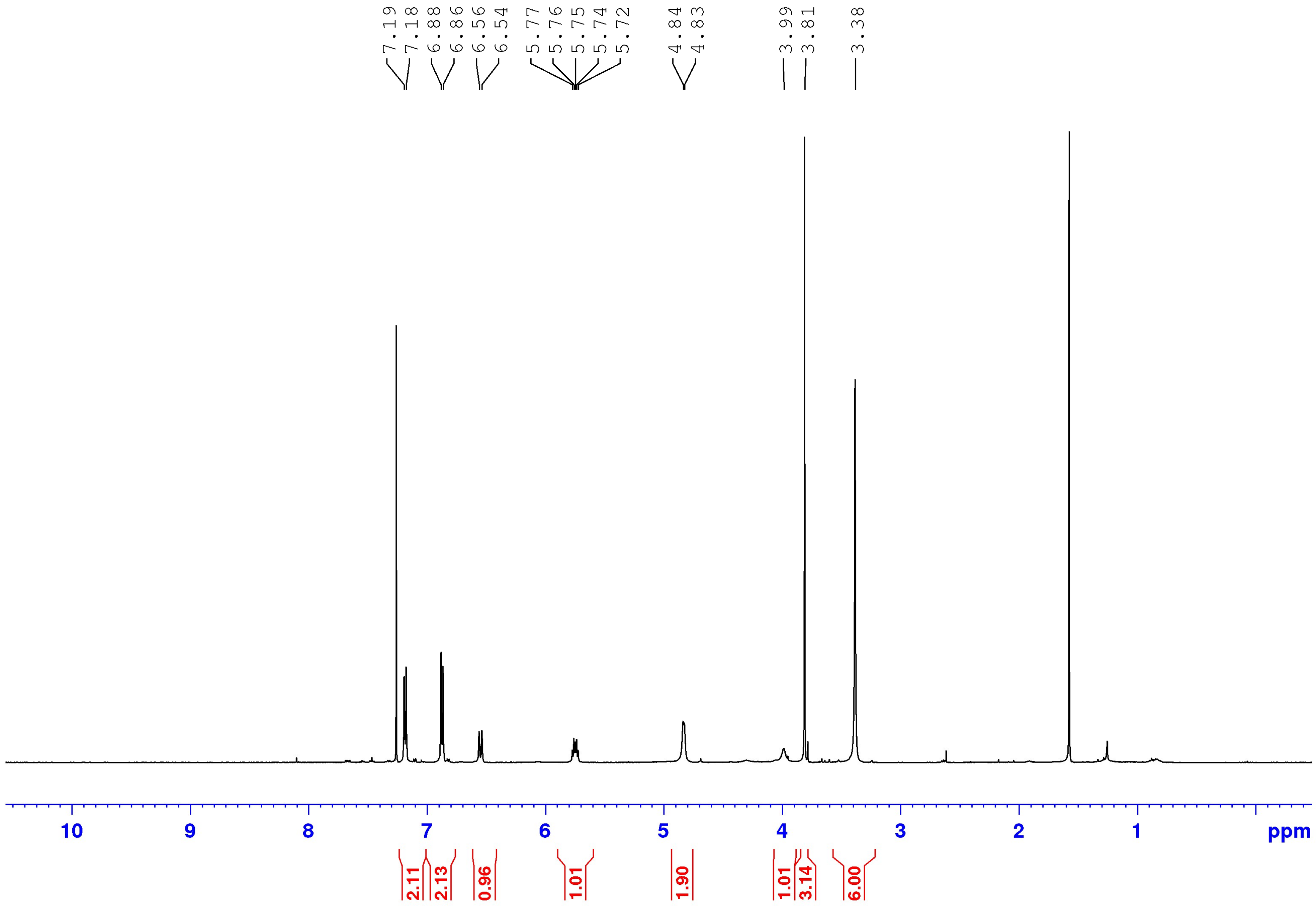

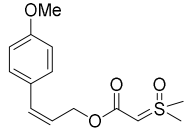

1h

Figure S36. ^{1}H NMR (500 MHz, $CDCl_3$) of **1h**

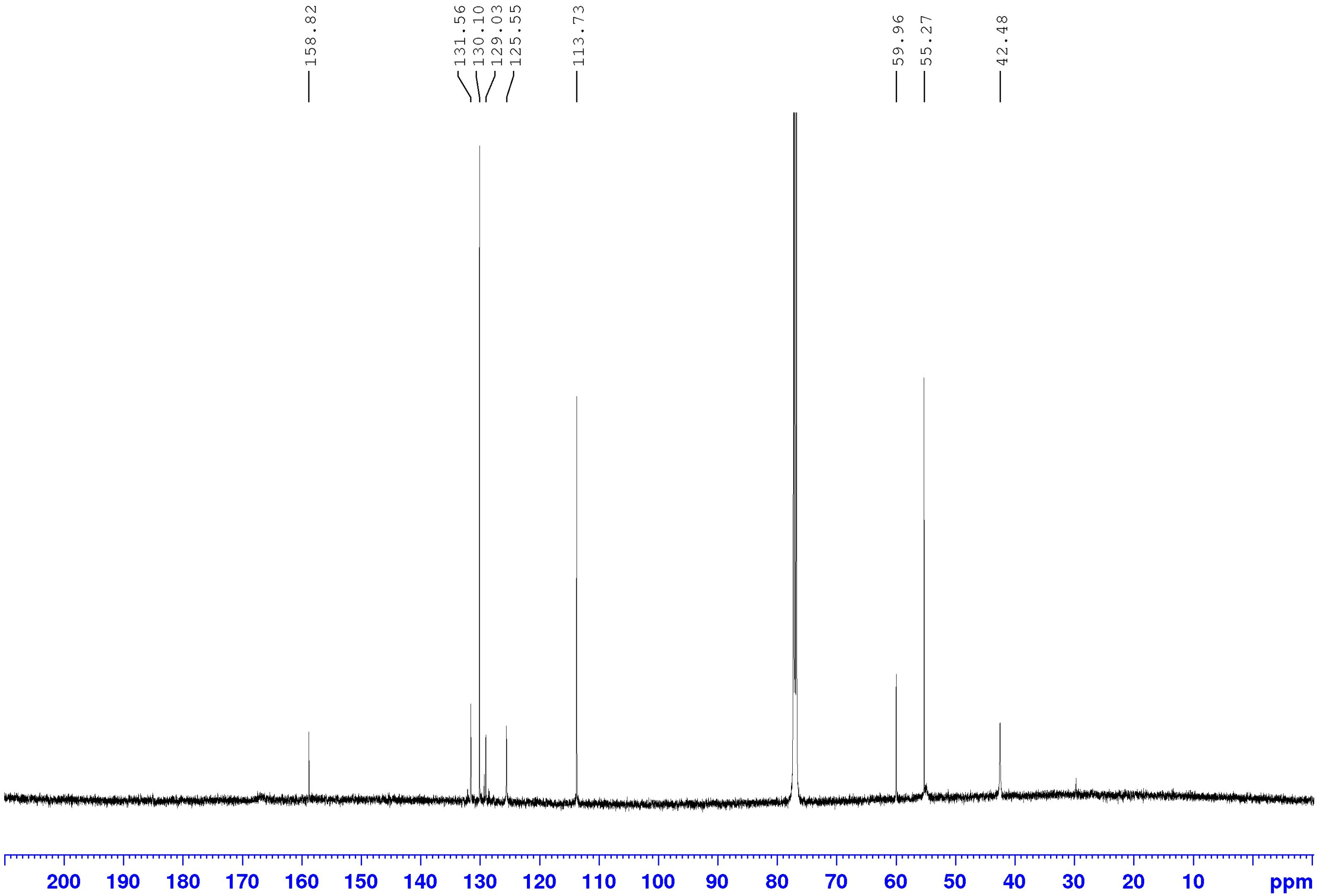

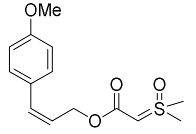

1h

Figure S37. $^{13}C\{^{1}H\}$ NMR (126 MHz, $CDCl_3$) of **1h**

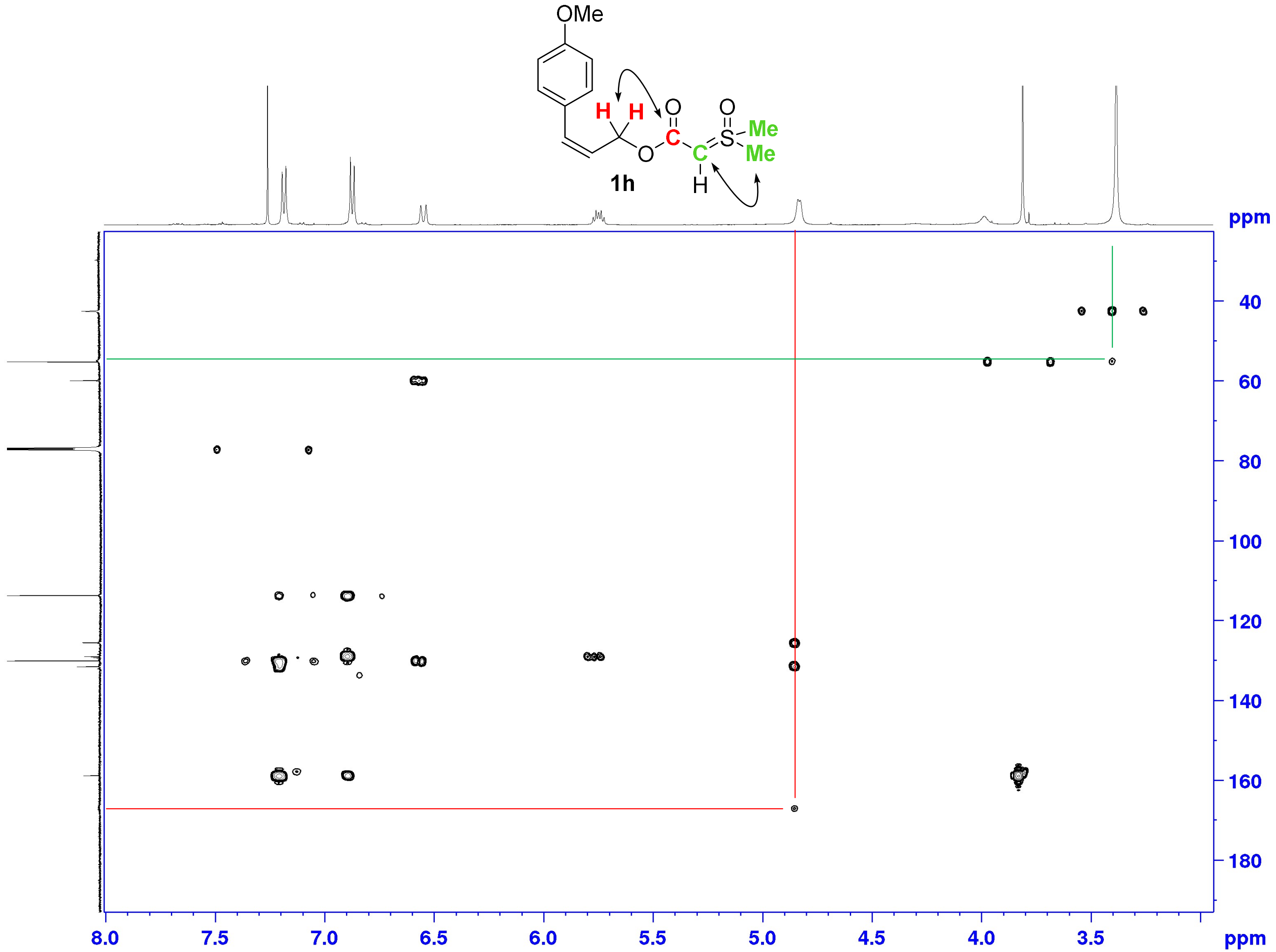

Figure S38. HMBC of **1h**

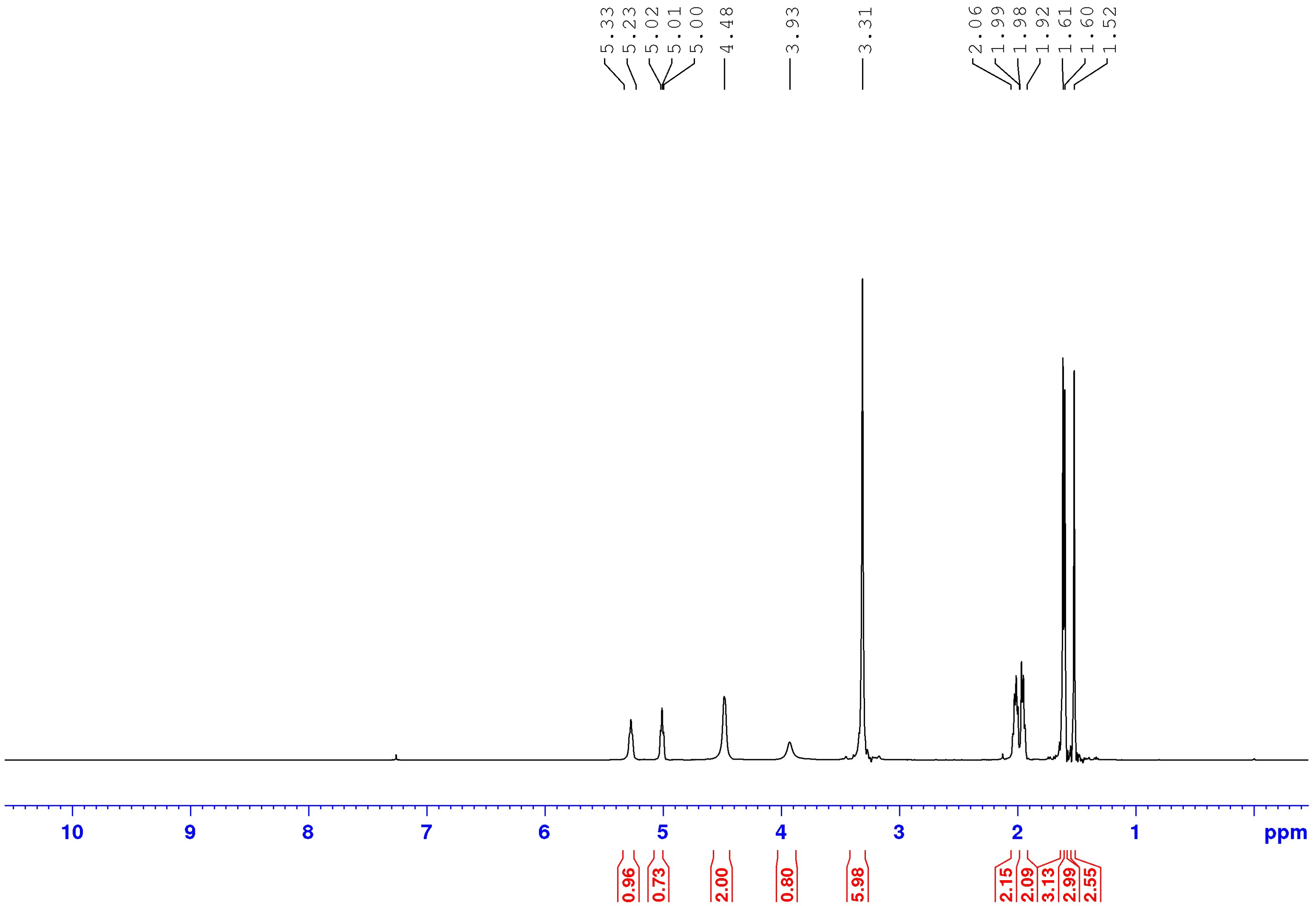

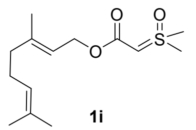

1i

Figure S39. ^{1}H NMR (500 MHz, $CDCl_3$) of **1i**

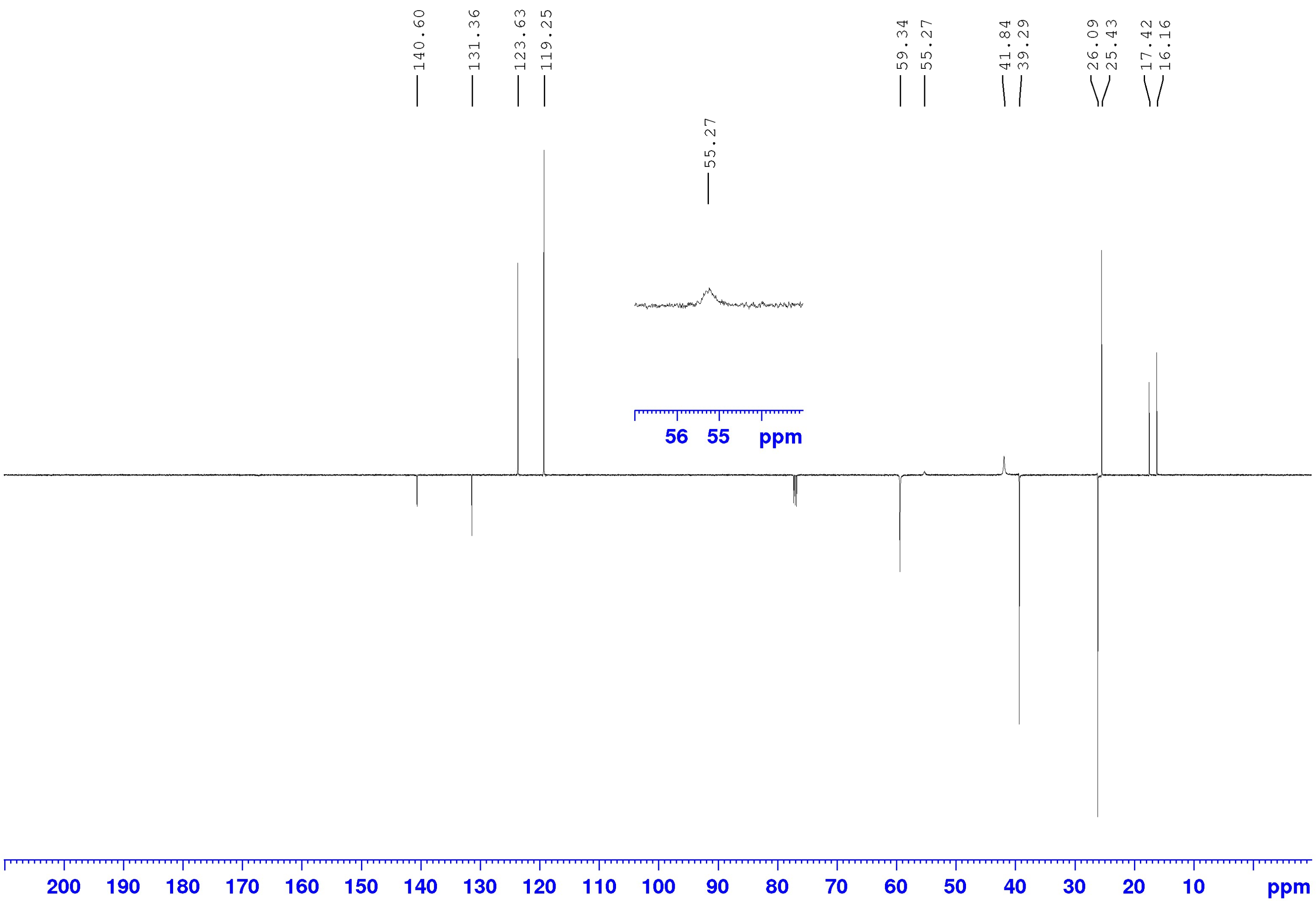

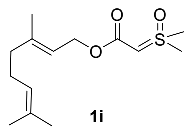

1i

Figure S40. $^{13}C\{^1H\}$ NMR (126 MHz, $CDCl_3$) of **1i**

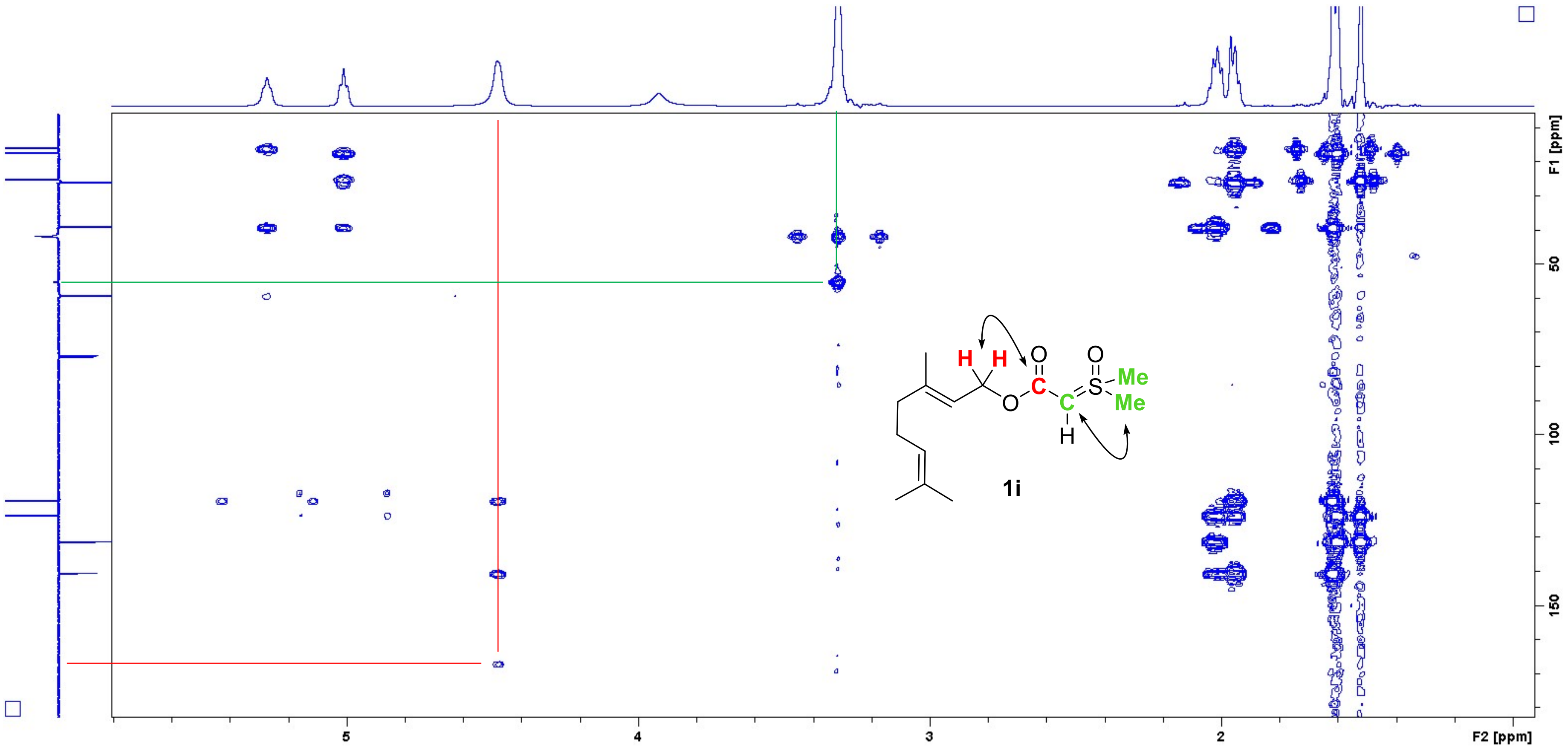

Figure S41. HMBC of **1i**

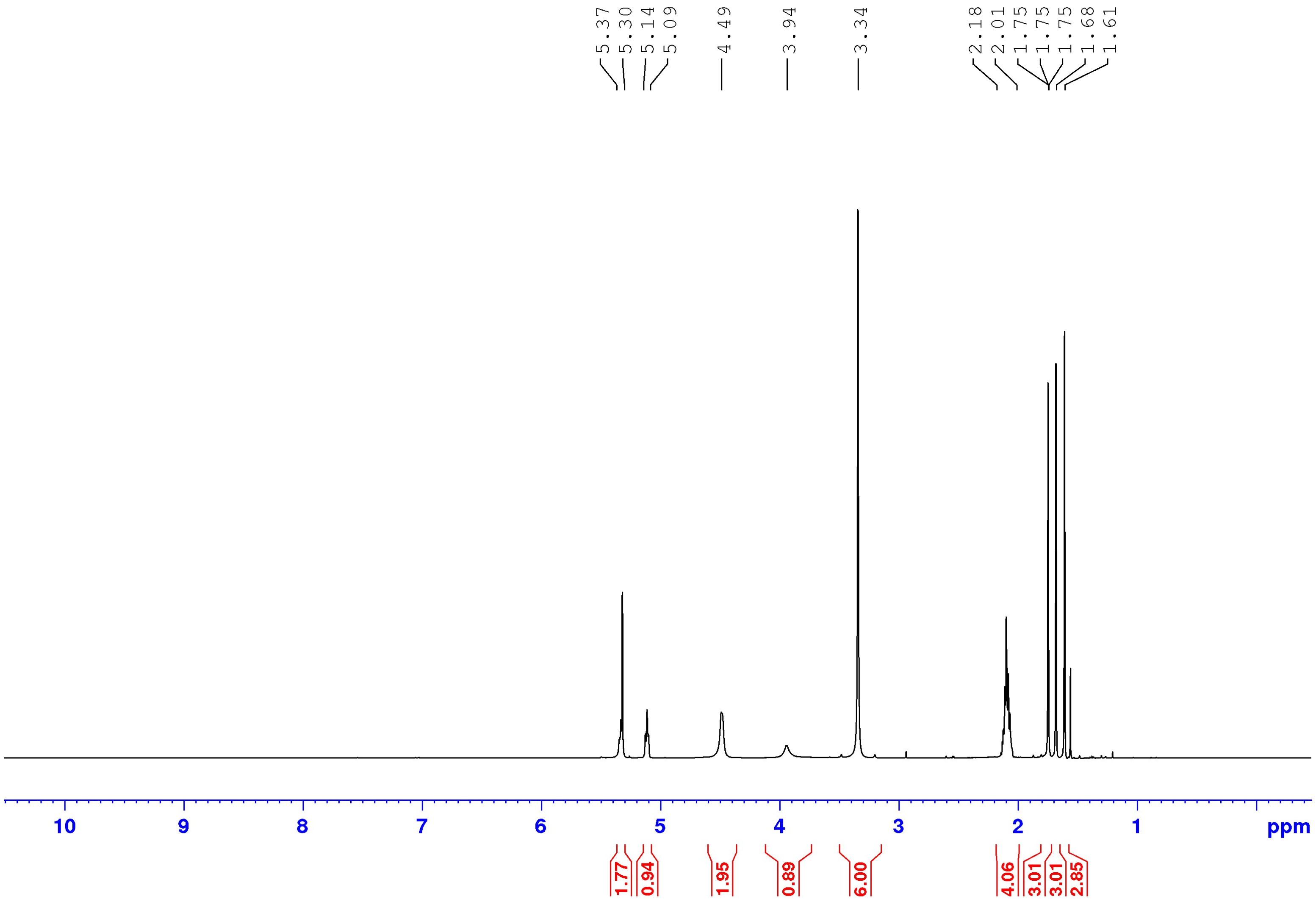

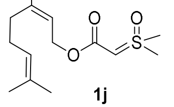

1j

Figure S42. ^{1}H NMR (500 MHz, CD_2Cl_2) of **1j**

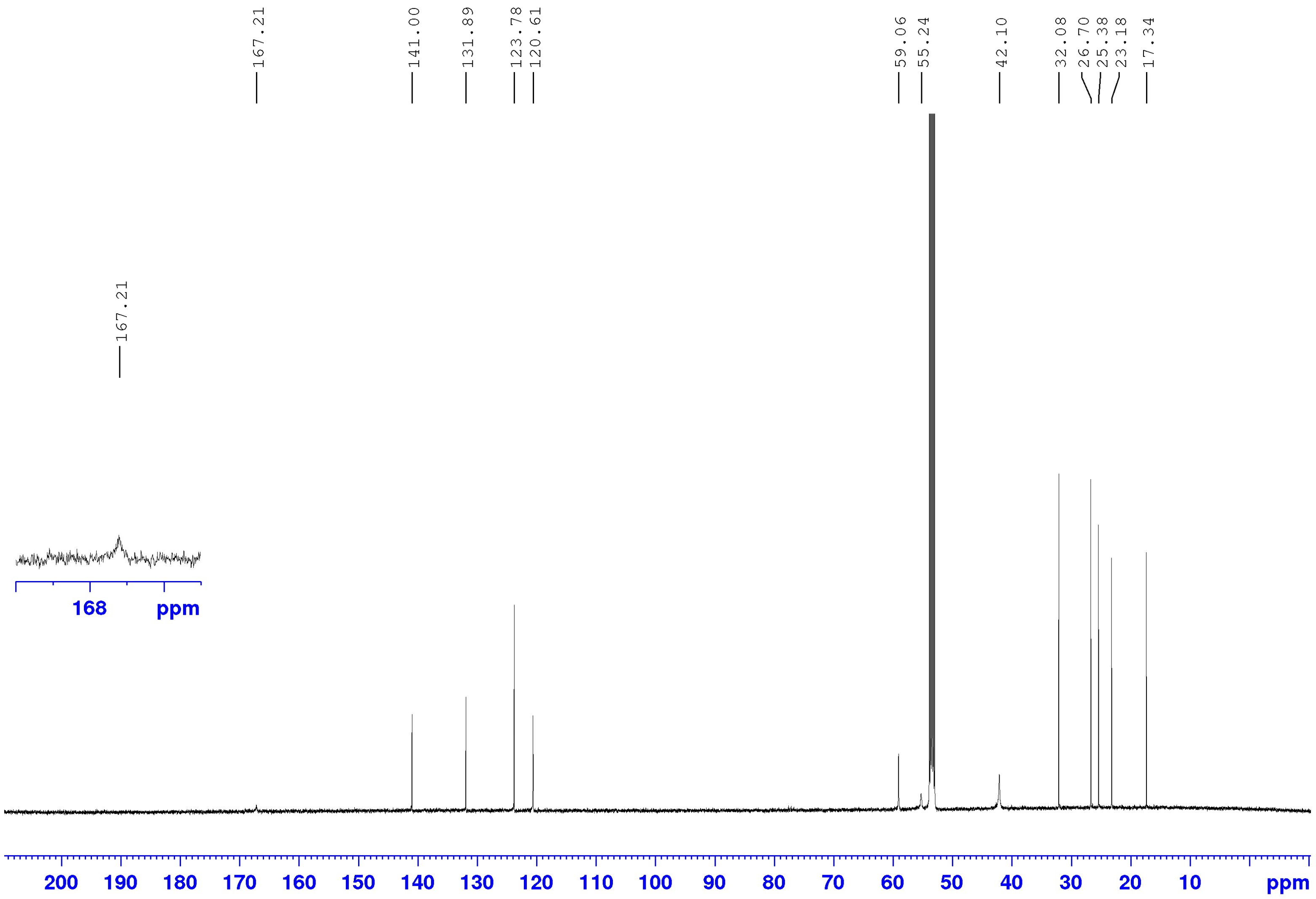

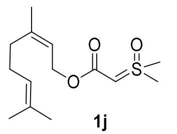

1j

Figure S43. $^{13}C\{^{1}H\}$ NMR (126 MHz, CD_2Cl_2) of **1j**

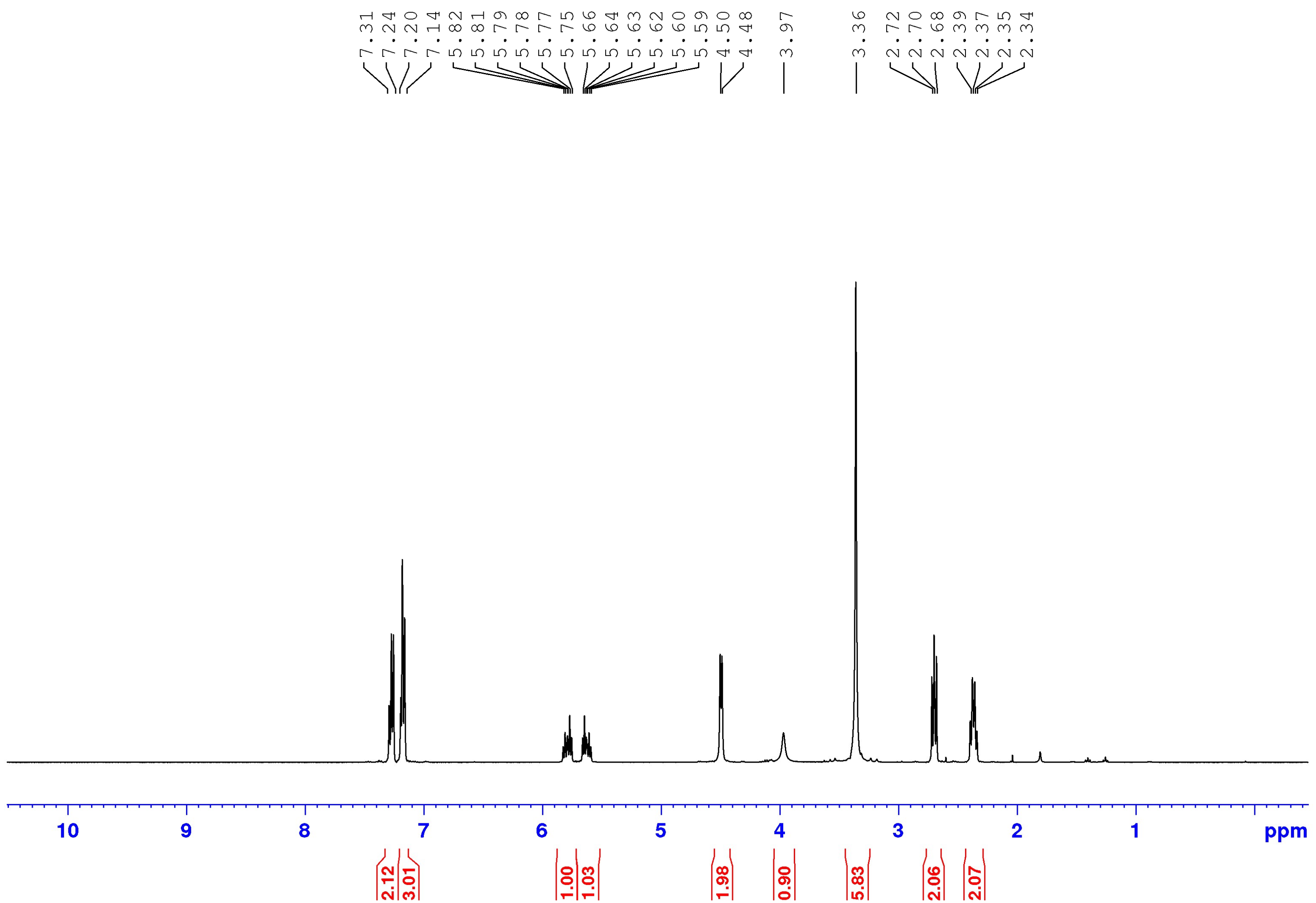

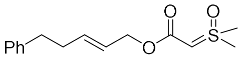

1k

Figure S44. ^{1}H NMR (500 MHz, $CDCl_3$) of **1k**

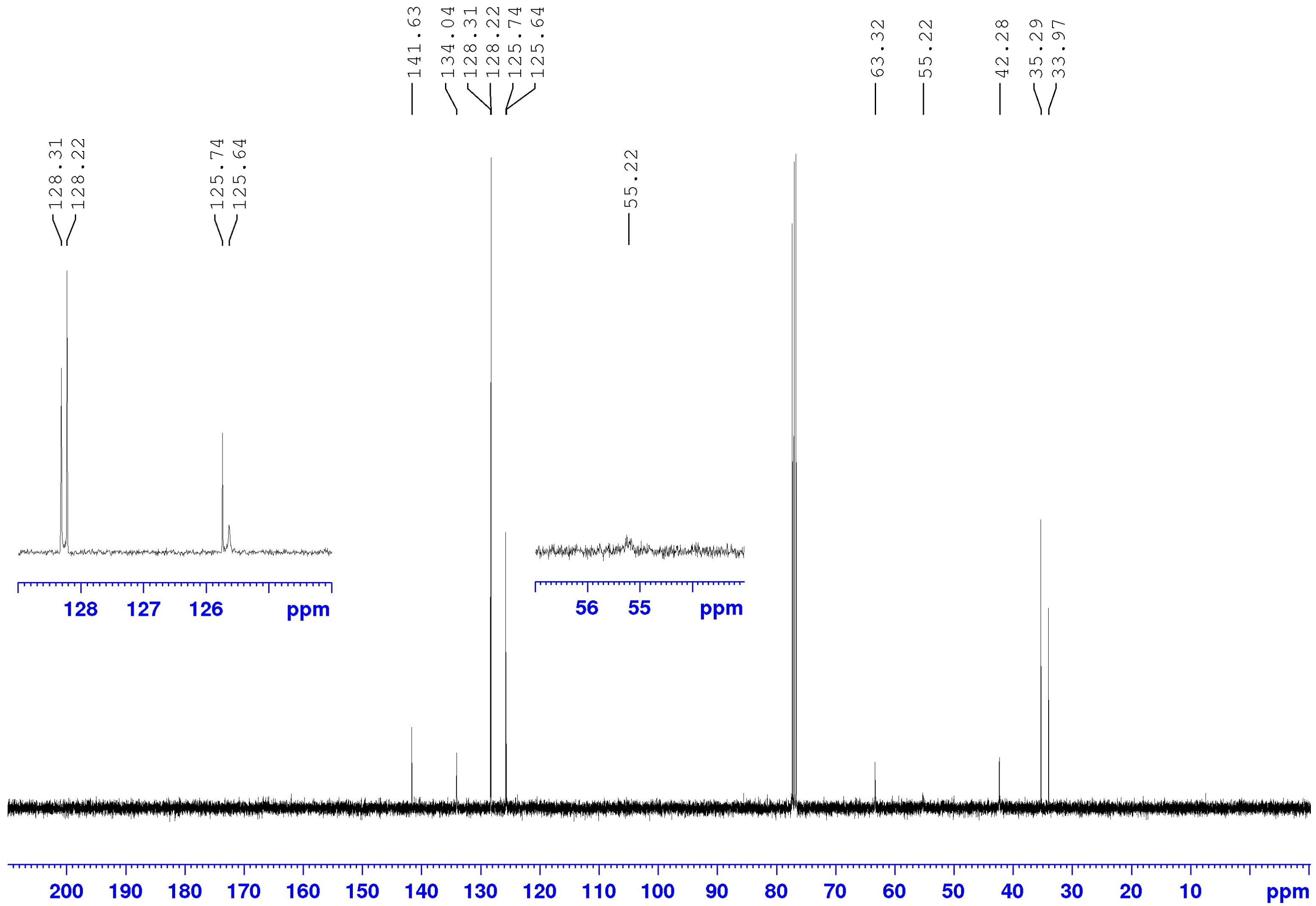

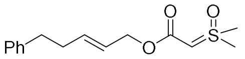

1k

Figure S45. $^{13}C\{^1H\}$ NMR (126 MHz, $CDCl_3$) of **1k**

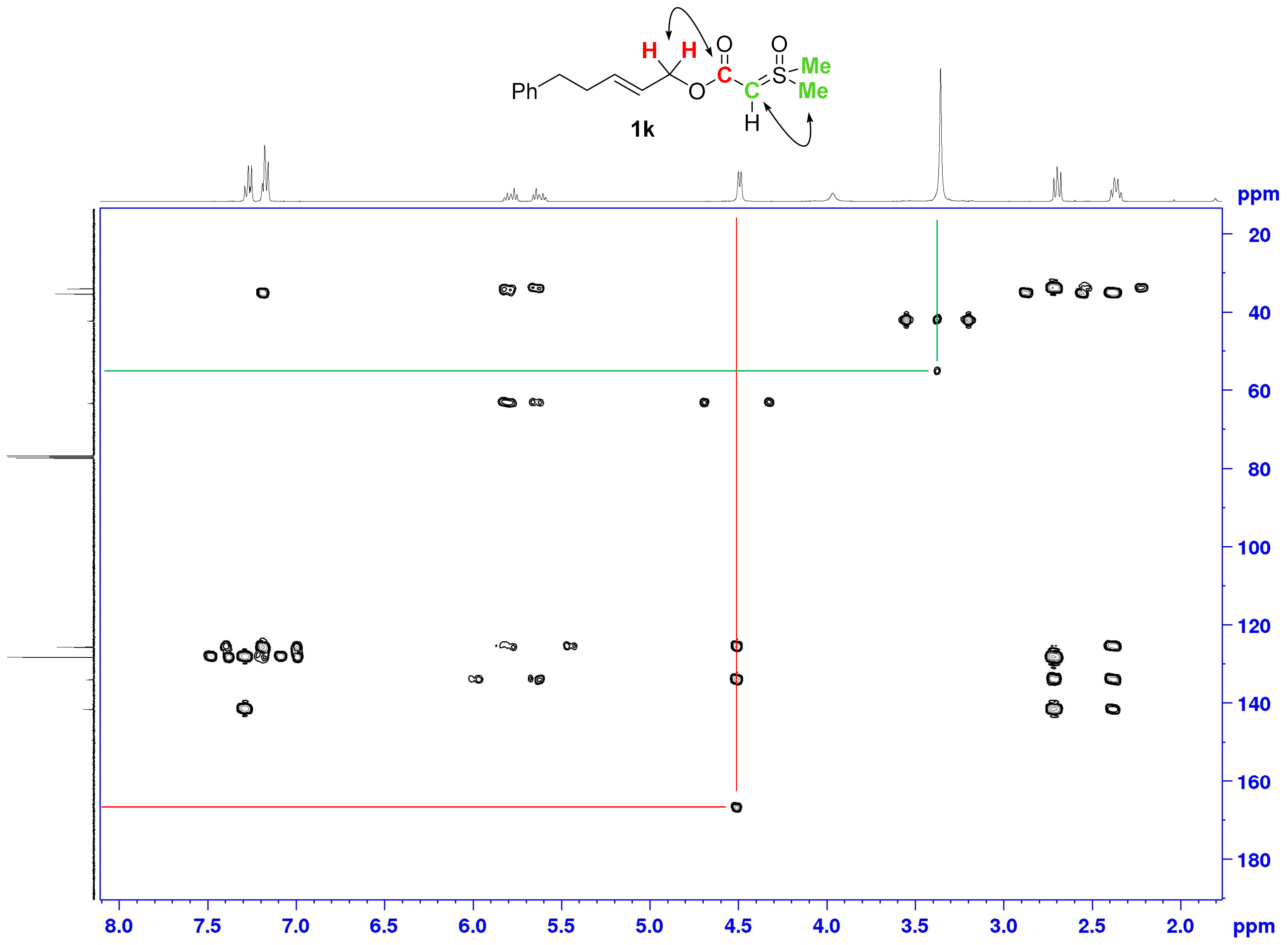

Figure S46. HMBC of **1k**

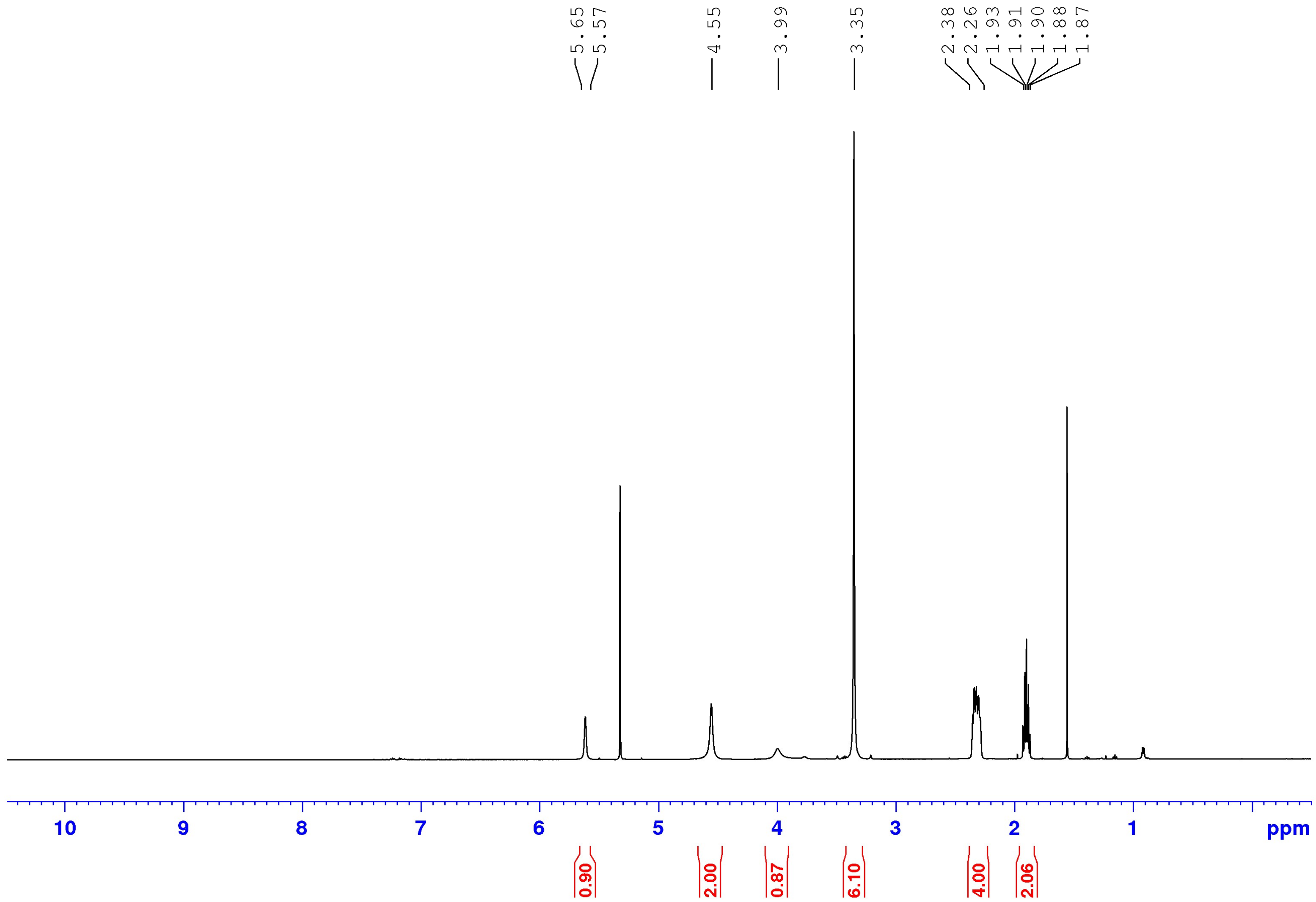

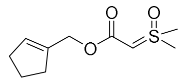

1l

Figure S47. ^{1}H NMR (500 MHz, CD_2Cl_2) of **1l**

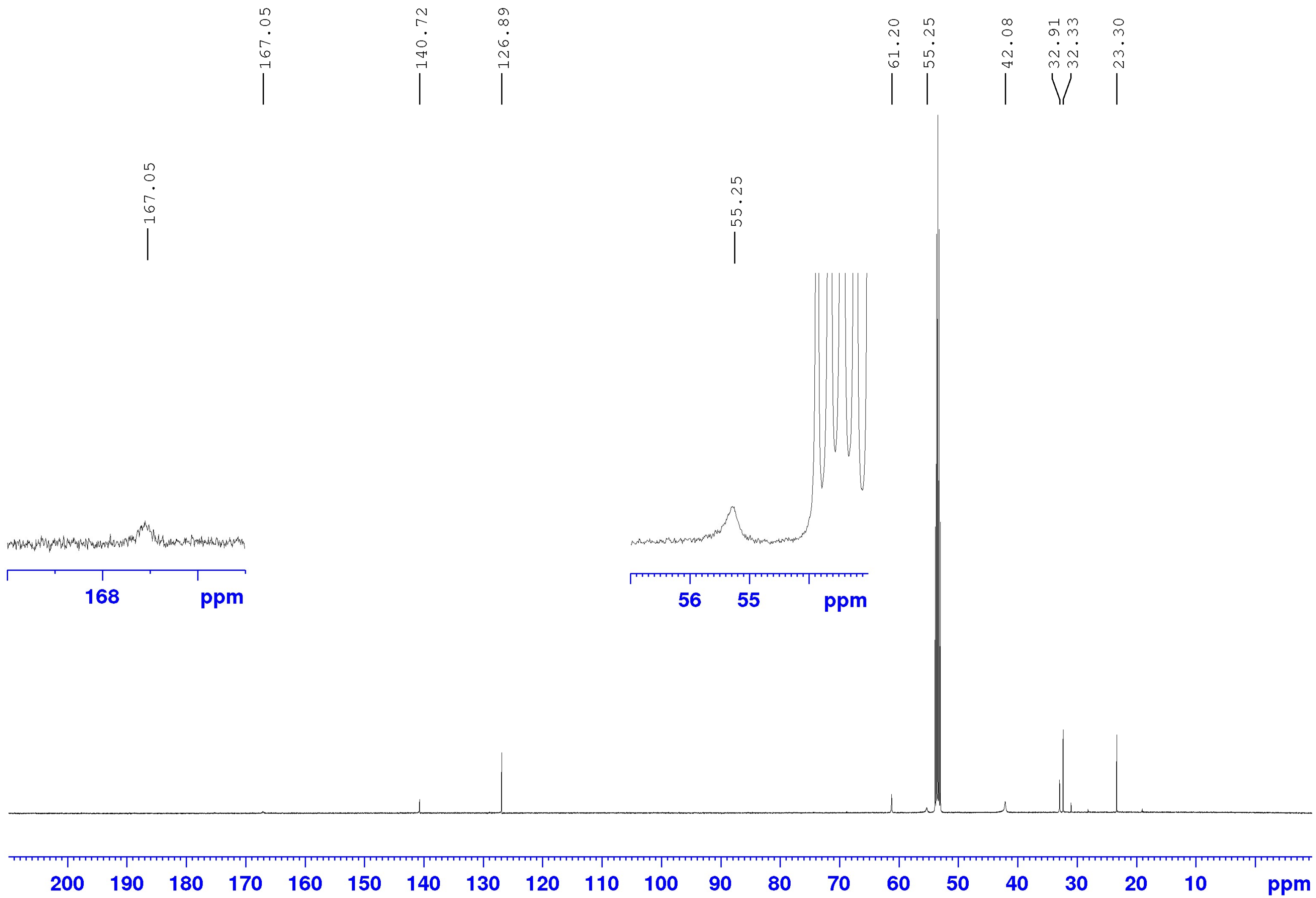

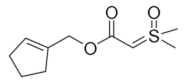

1l

Figure S48. $^{13}C\{^{1}H\}$ NMR (126 MHz, CD_2Cl_2) of **1l**

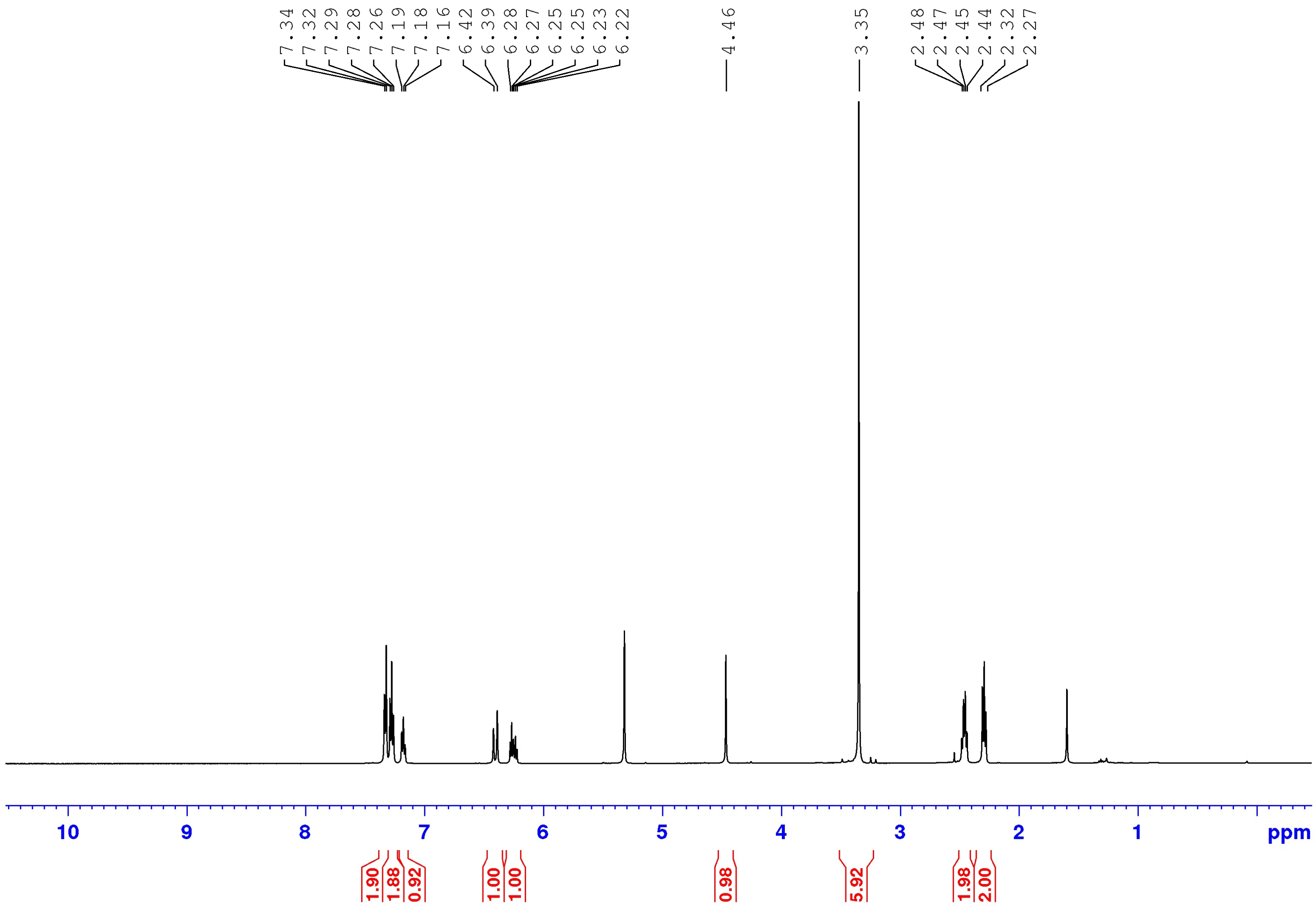

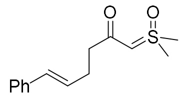

1m

Figure S49. ^{1}H NMR (500 MHz, CD_2Cl_2) of **1m**

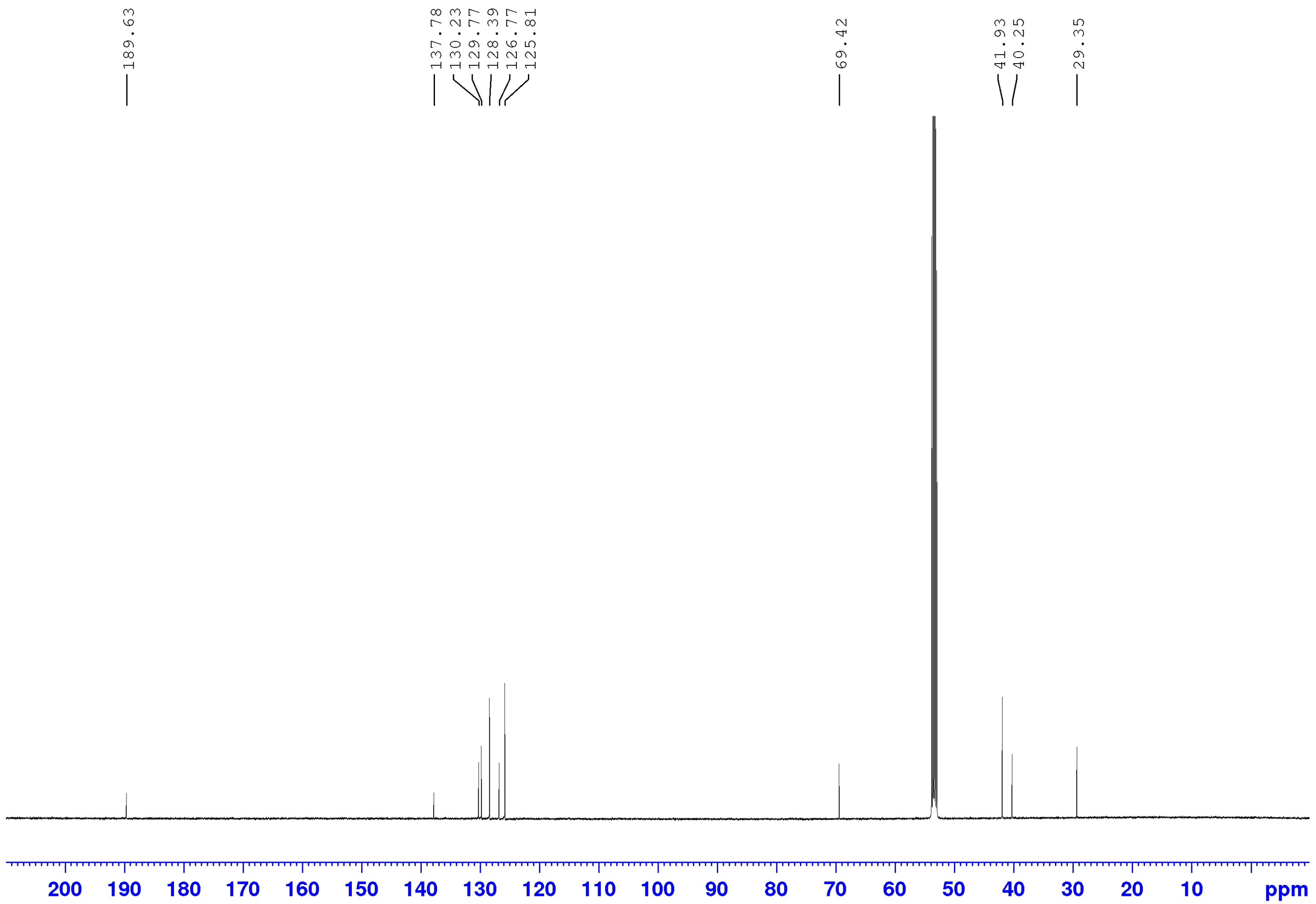

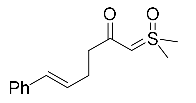

1m

Figure S50. $^{13}C\{^{1}H\}$ NMR (126 MHz, CD_2Cl_2) of **1m**

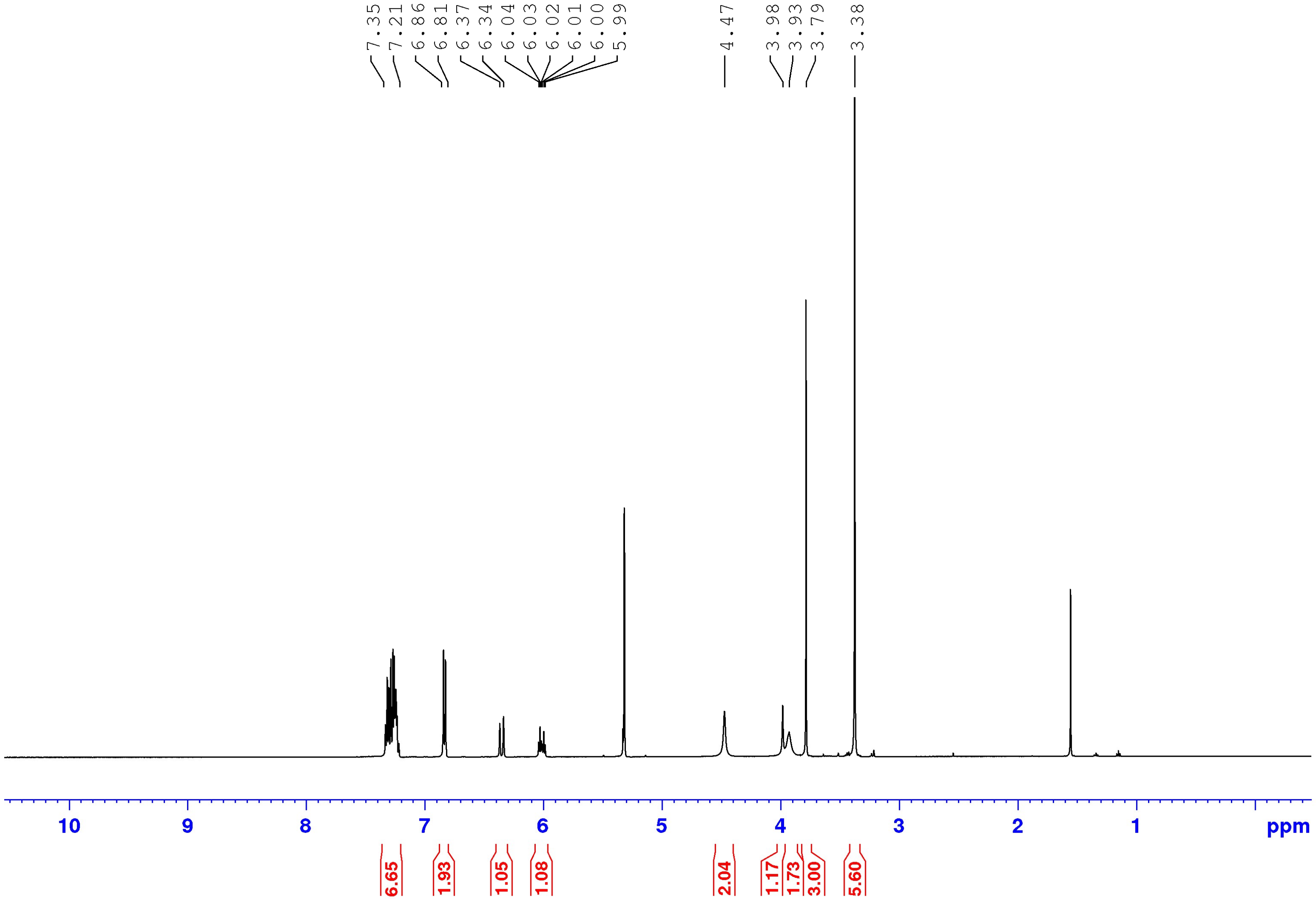

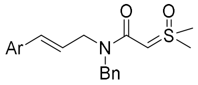

1n (Ar = 4-MeO-C_6H_4)

Figure S51. ^{1}H NMR (500 MHz, CD_2Cl_2) of **1n**

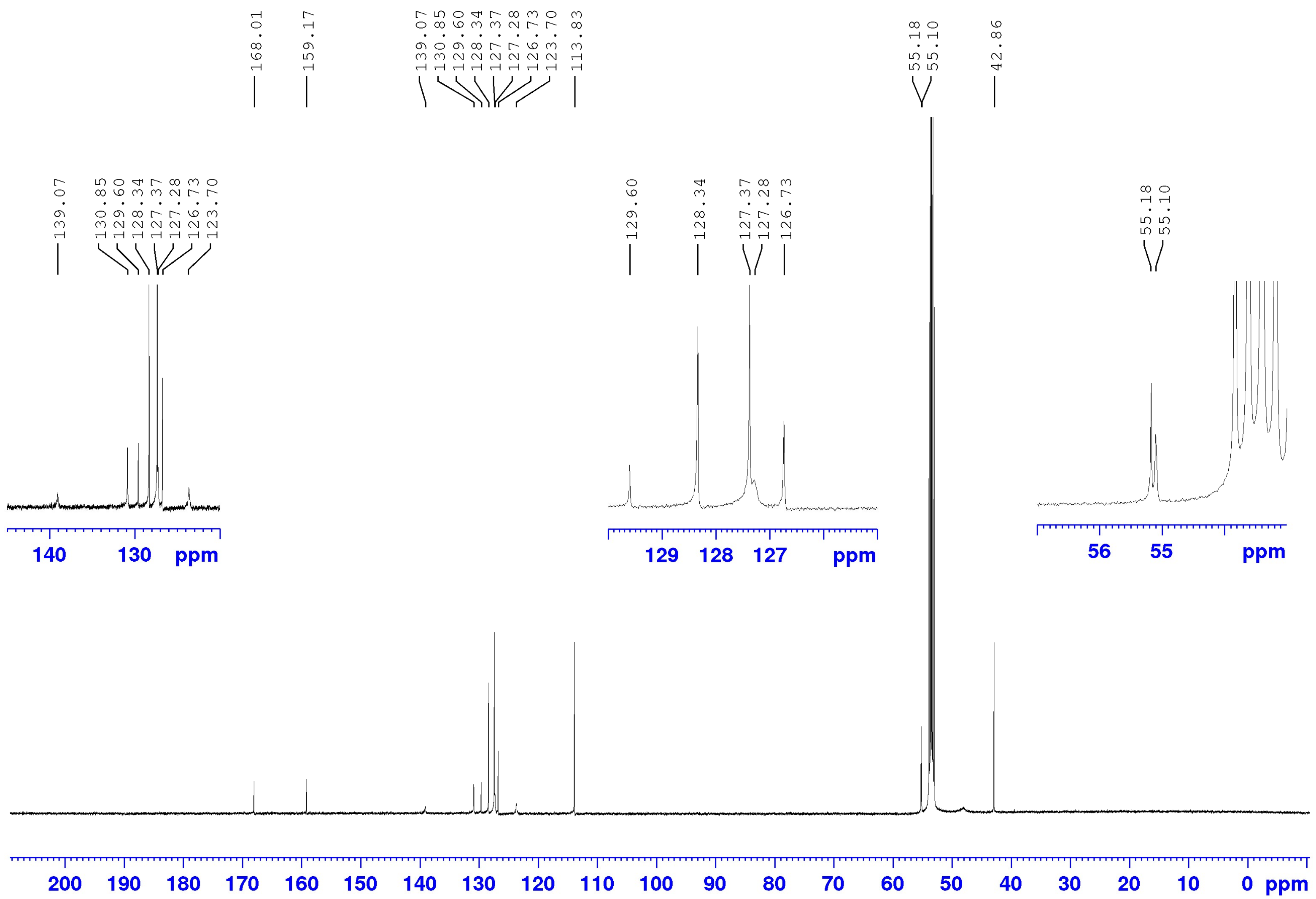

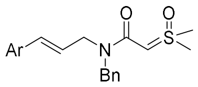

1n (Ar = 4-MeO-C_6H_4)

Figure S52. $^{13}C\{^1H\}$ NMR (126 MHz, CD_2Cl_2) of **1n**

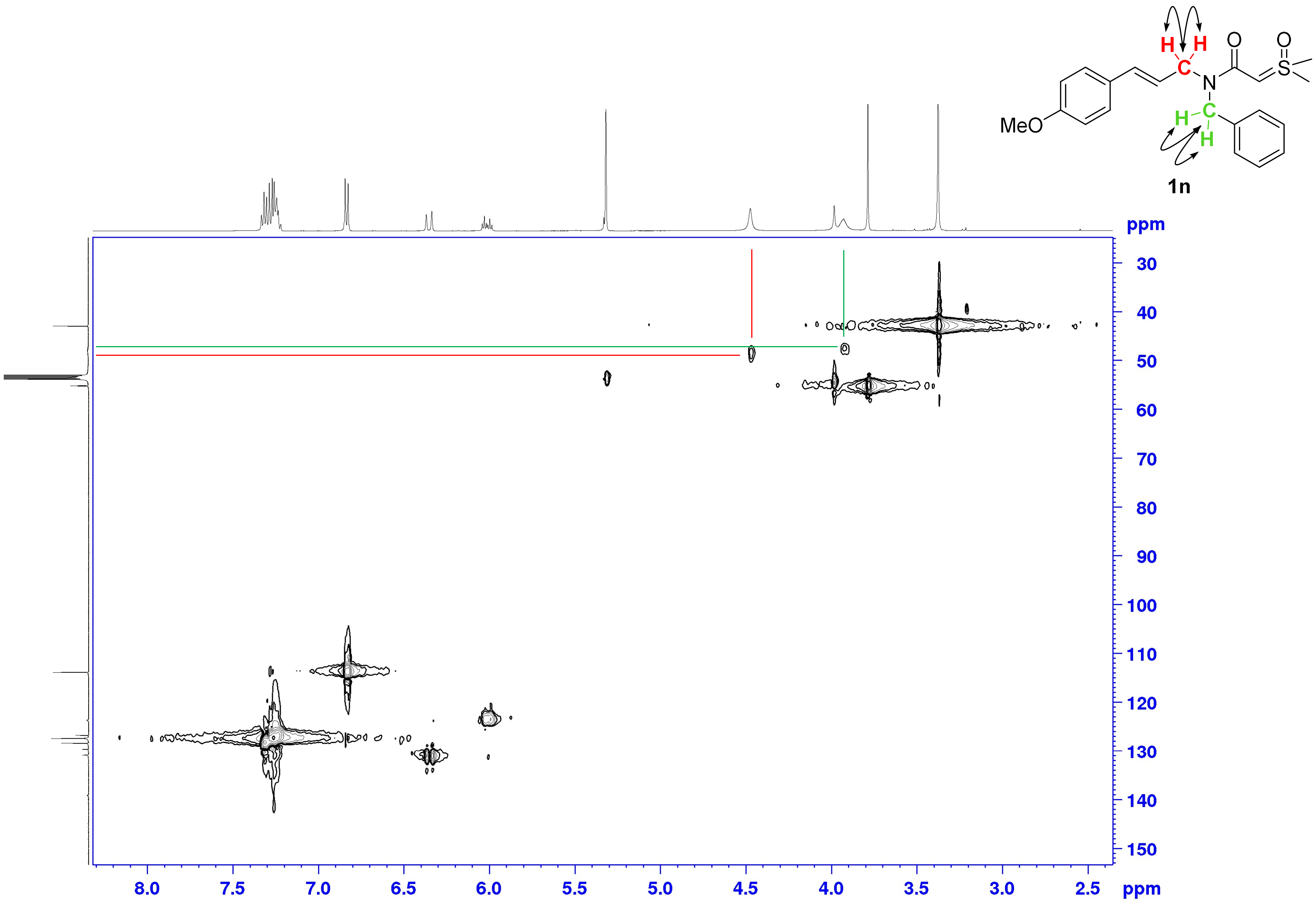

Figure S53. HSQC of **1n**

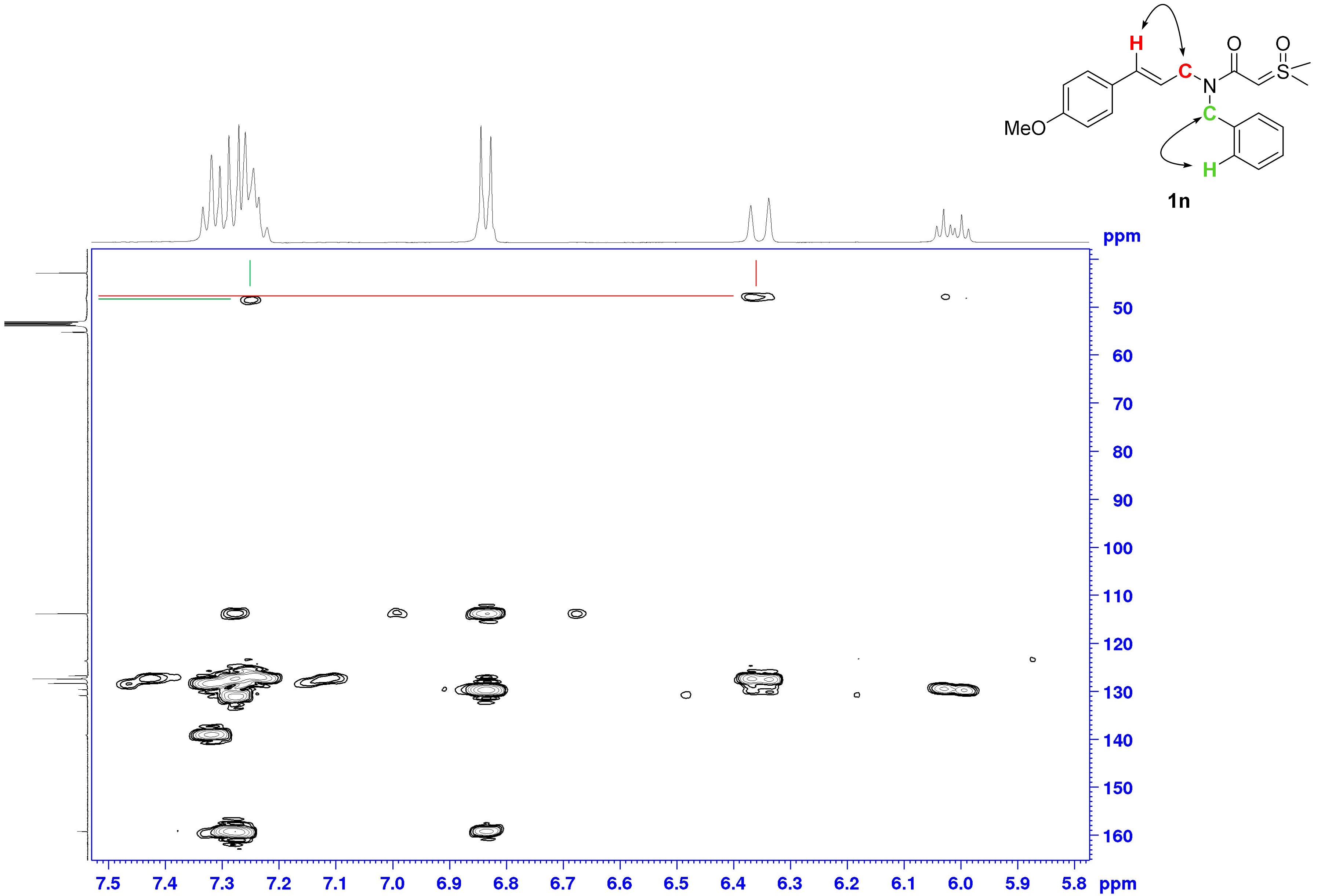

Figure S54. HMBC of **1n**

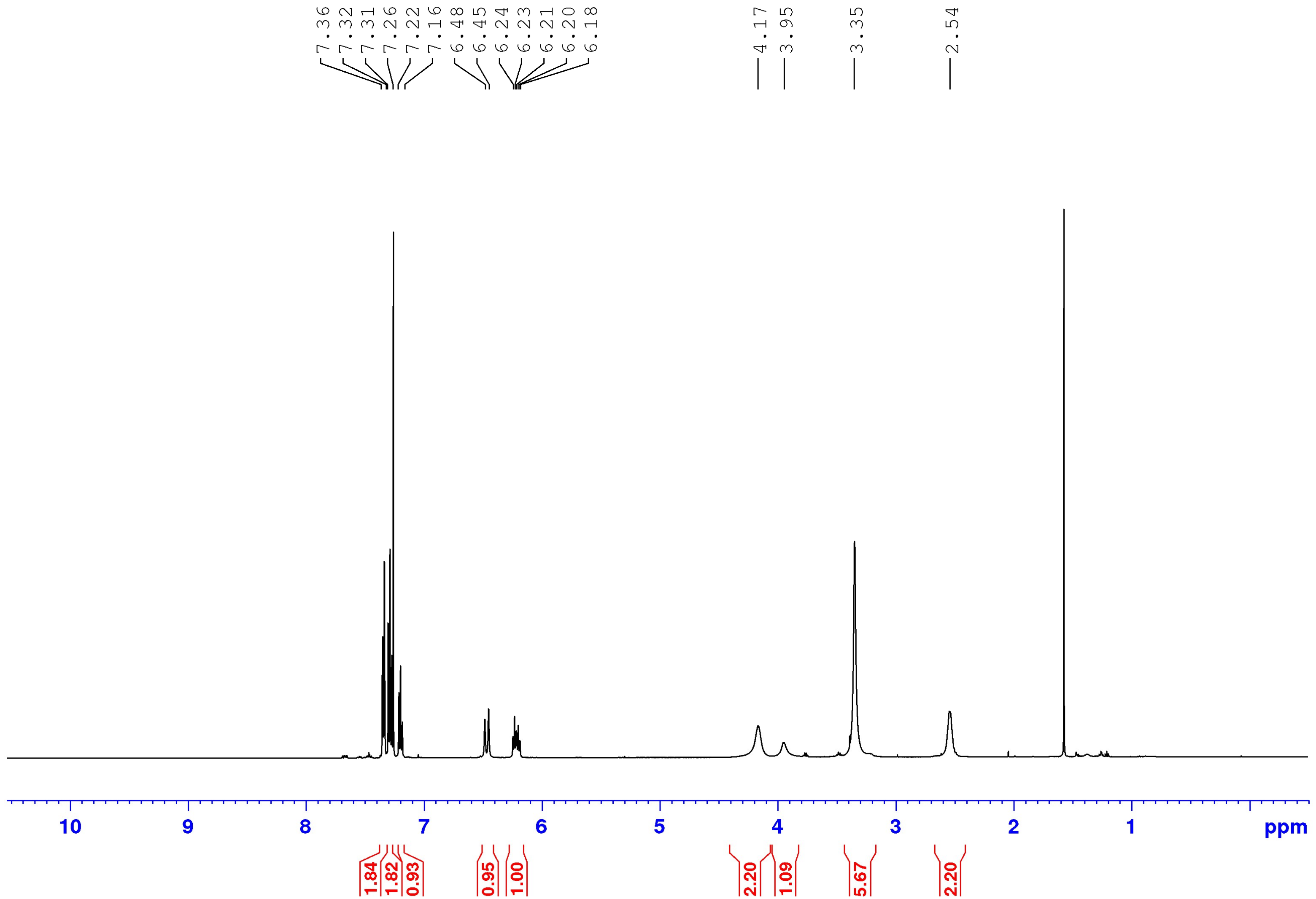

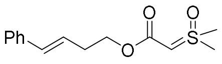

1o

Figure S55. ^{1}H NMR (500 MHz, $CDCl_3$) of **1o**

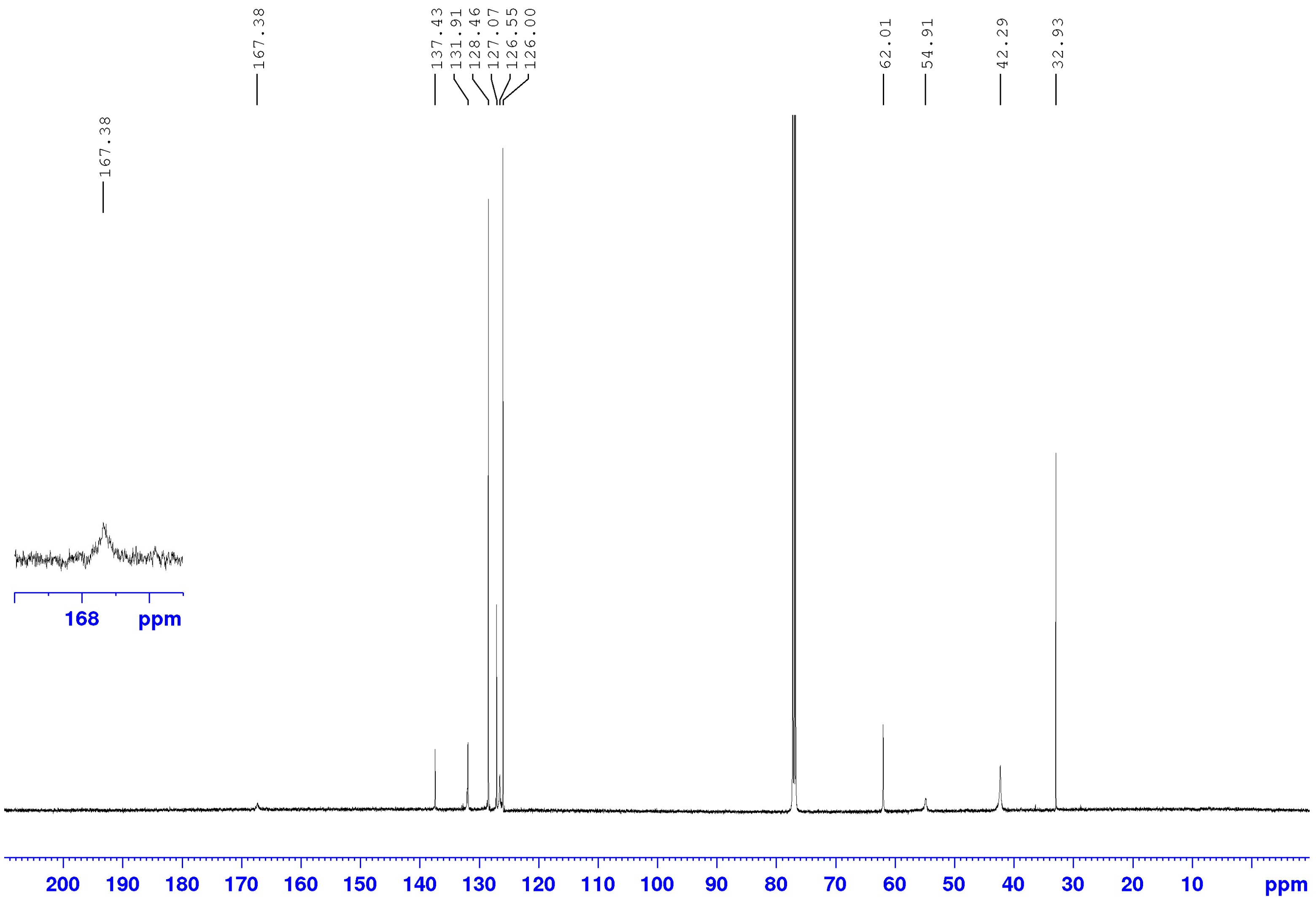

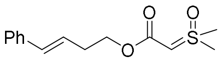

1o

Figure S56. $^{13}C\{^1H\}$ NMR (126 MHz, $CDCl_3$) of **1o**

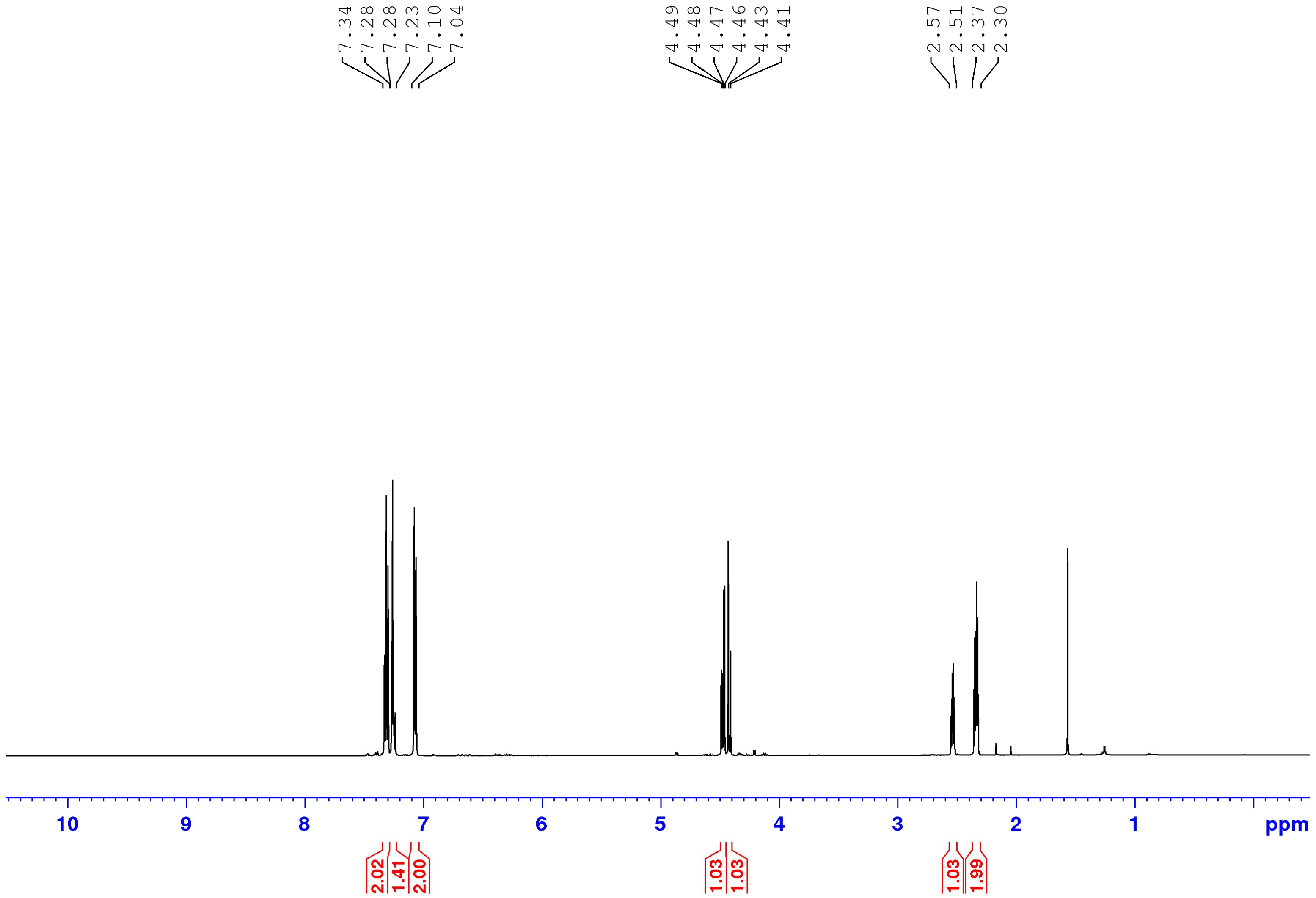

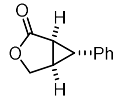

(–)-2a

Figure S57. ^{1}H NMR (500 MHz, $CDCl_3$) of **2a**

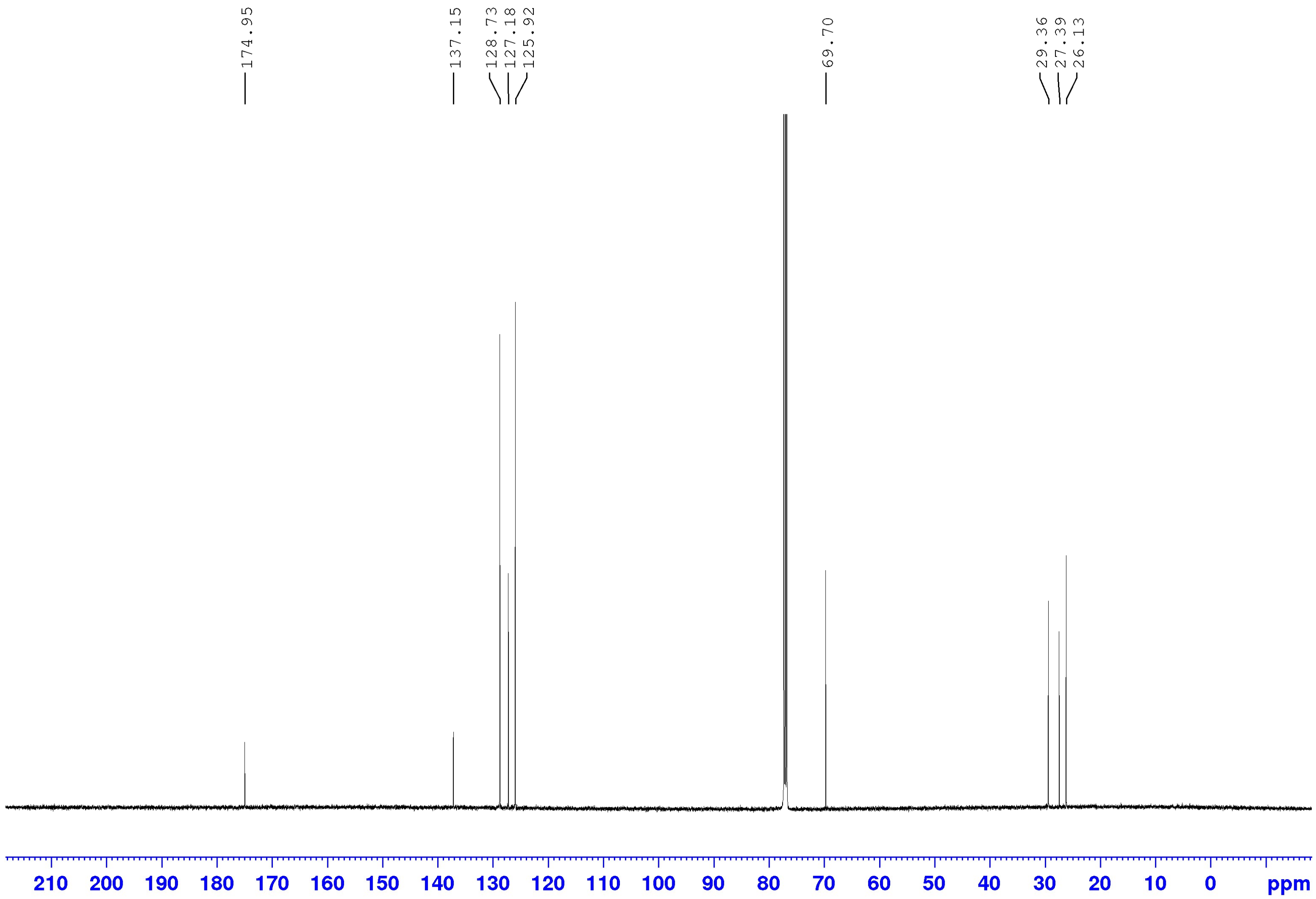

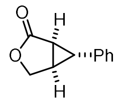

(–)-2a

Figure S58. $^{13}C\{^{1}H\}$ NMR (126 MHz, $CDCl_3$) of **2a**

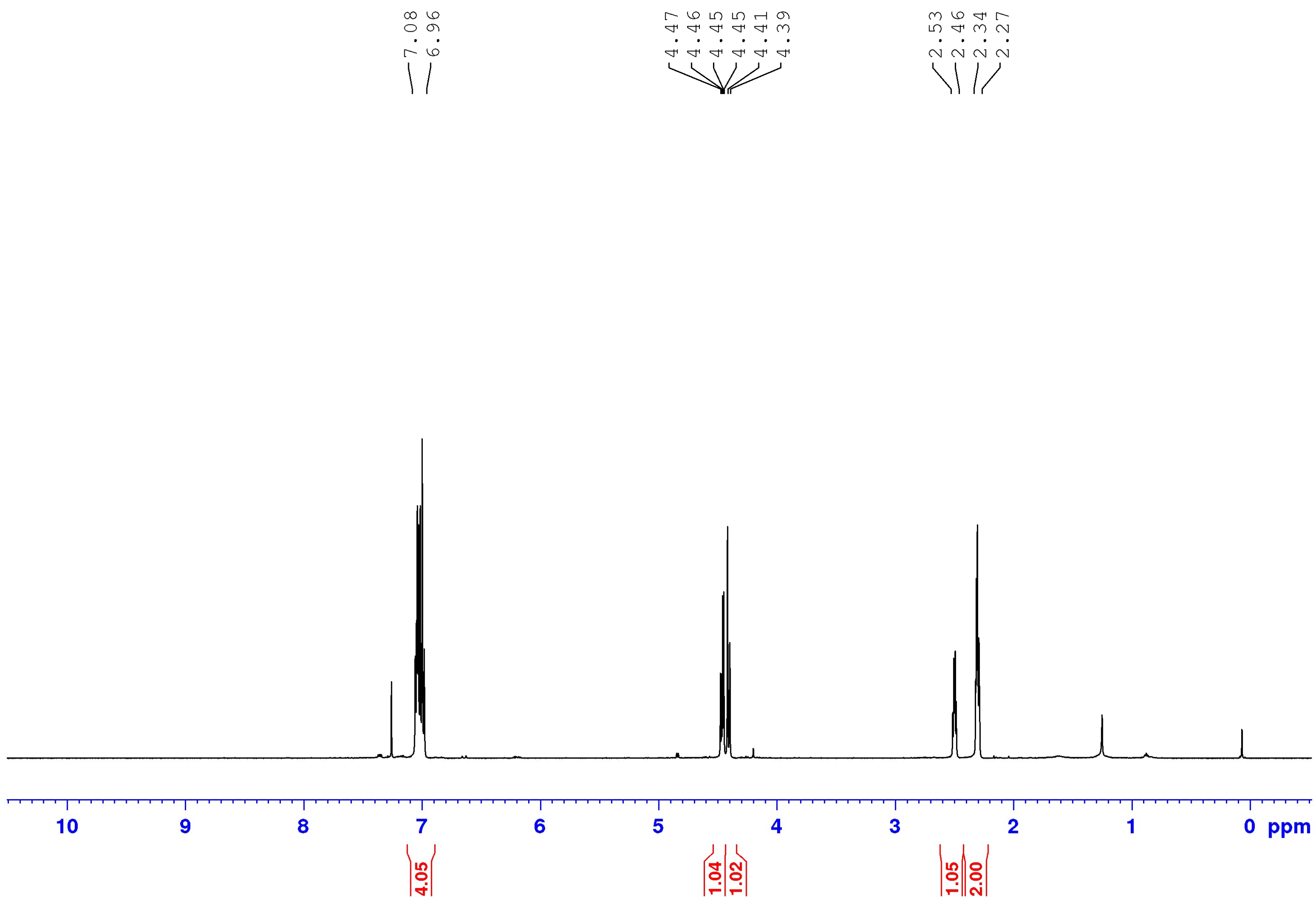

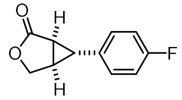

(−)-2b

Figure S59. ^{1}H NMR (500 MHz, $CDCl_3$) of **2b**

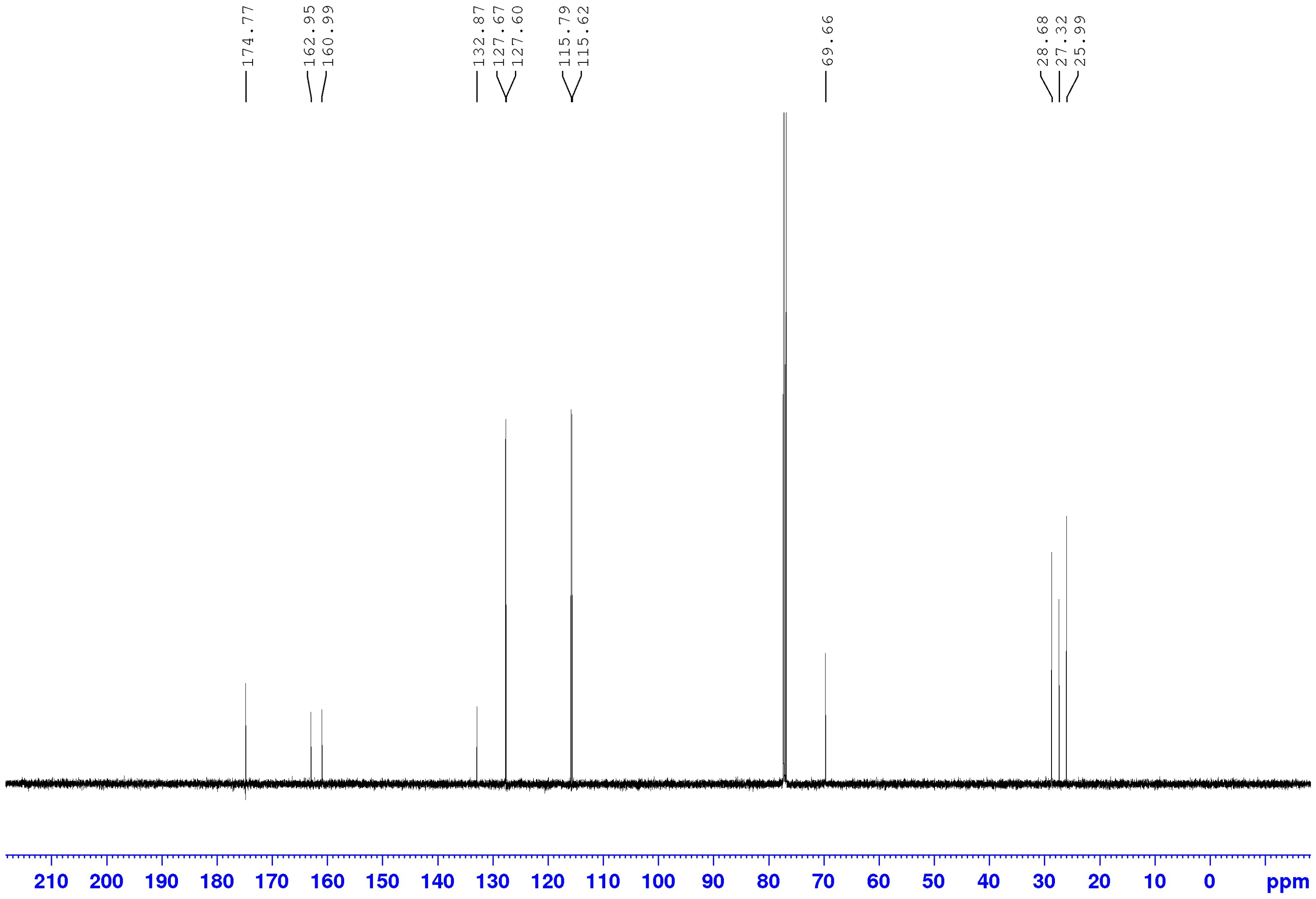

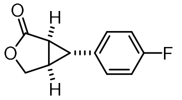

(–)-2b

Figure S60. $^{13}C\{^{1}H\}$ NMR (126 MHz, $CDCl_3$) of **2b**

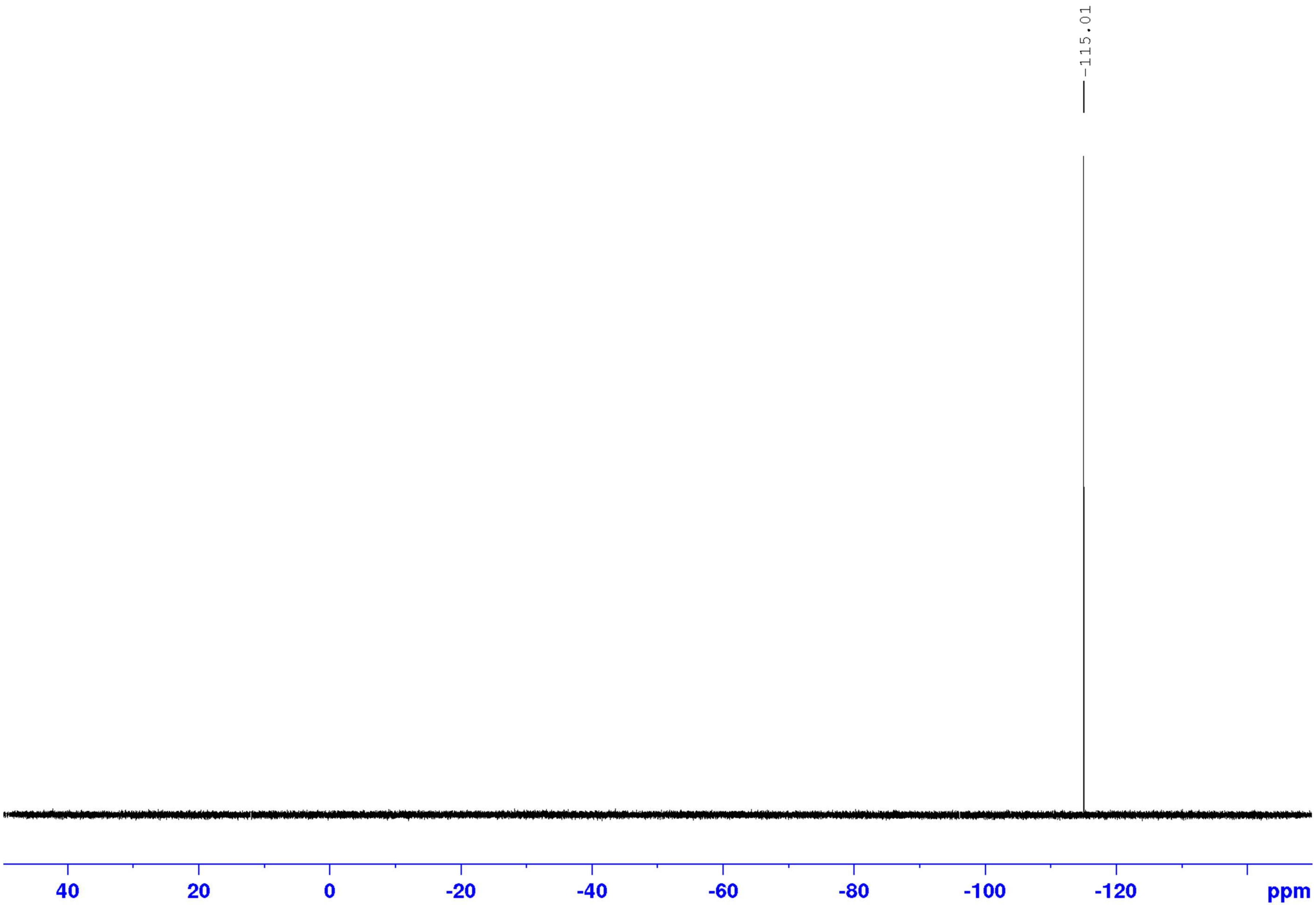

Figure S61. $^{19}F\{^{1}H\}$ NMR (470MHz, $CDCl_3$) of **2b**

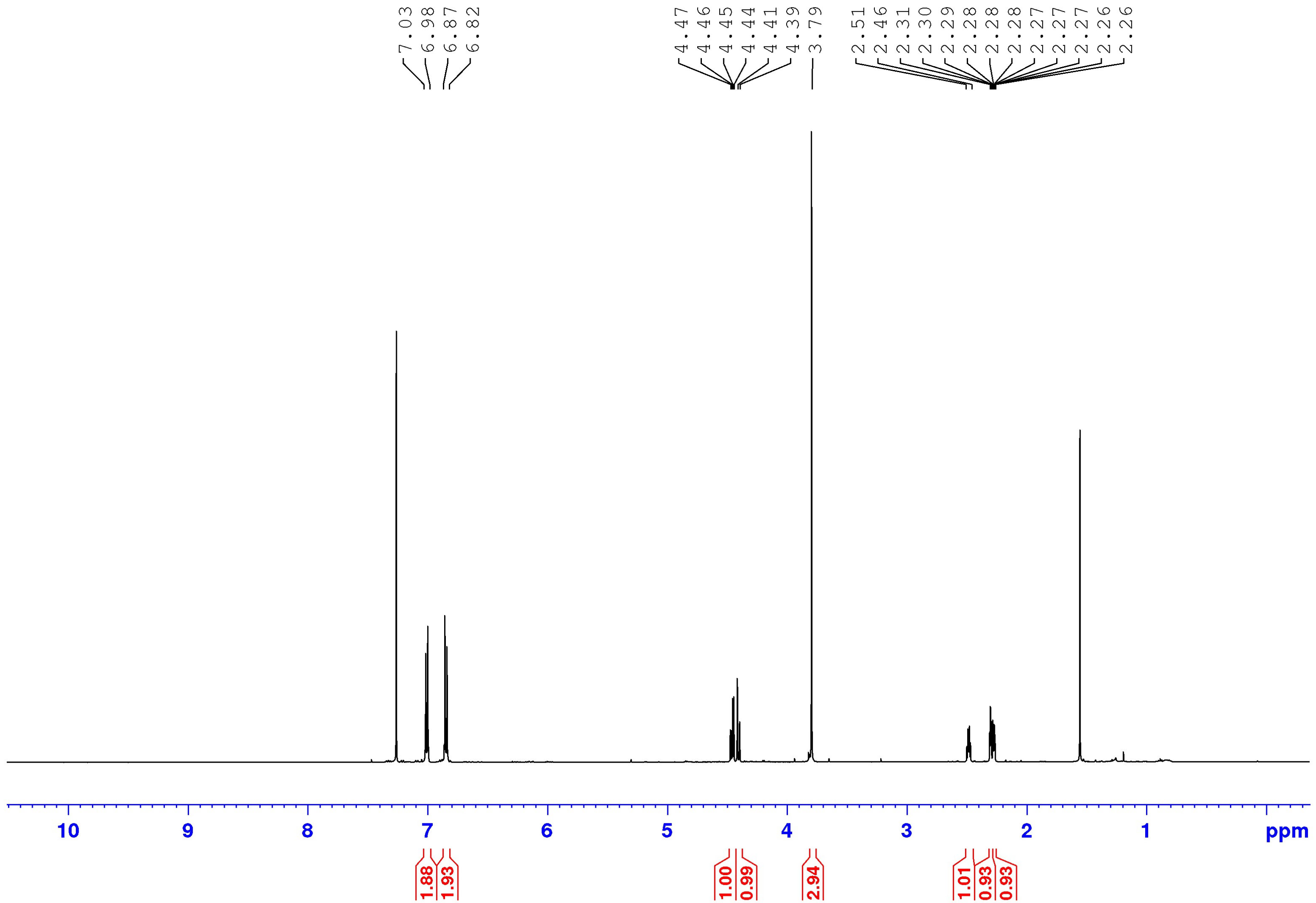

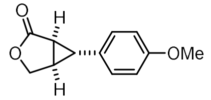

(–)-2c

Figure S62. ^{1}H NMR (500 MHz, $CDCl_3$) of **2c**

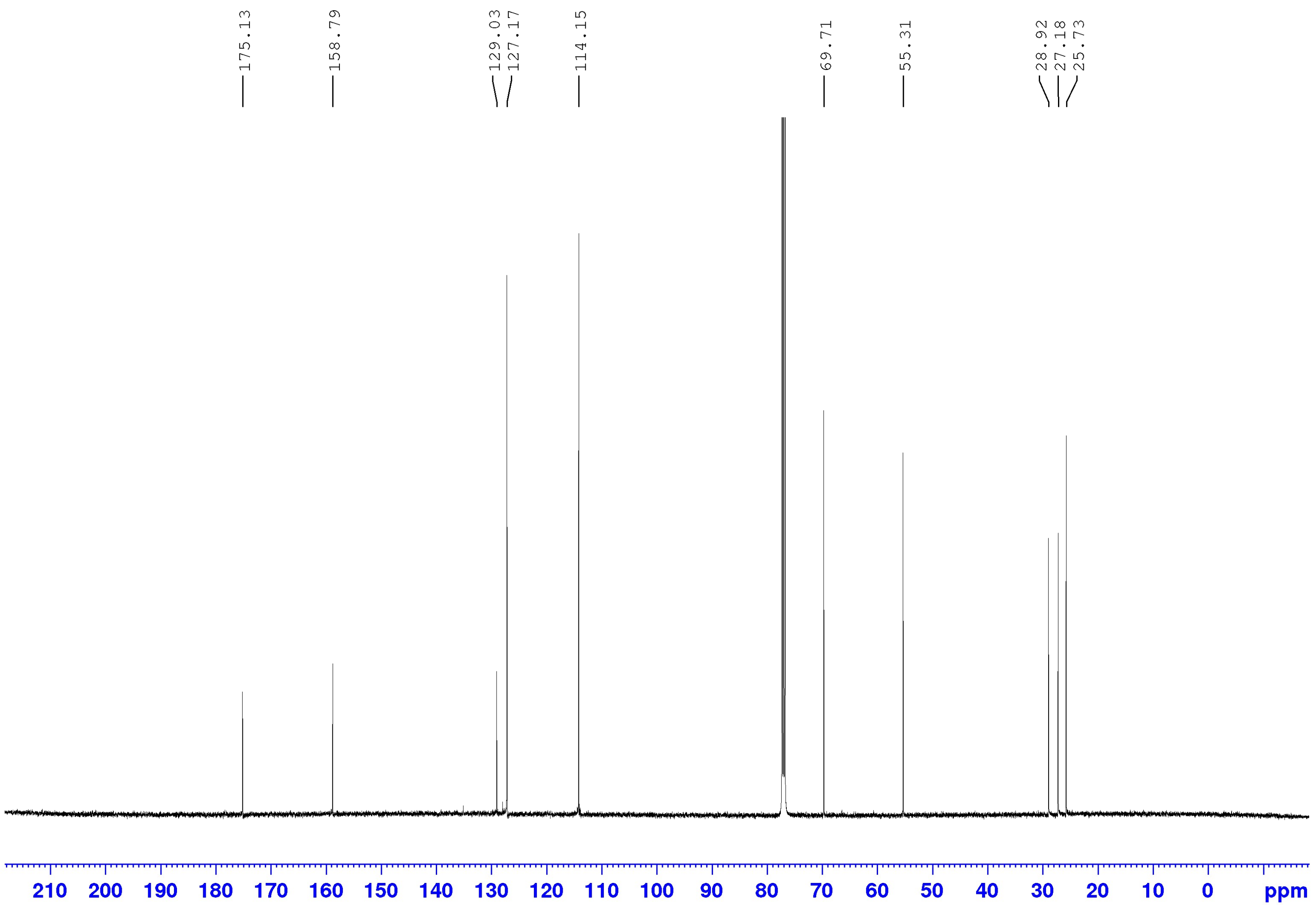

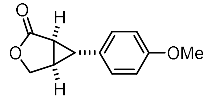

(–)-2c

Figure S63. $^{13}C\{^{1}H\}$ NMR (126 MHz, $CDCl_3$) of **2c**

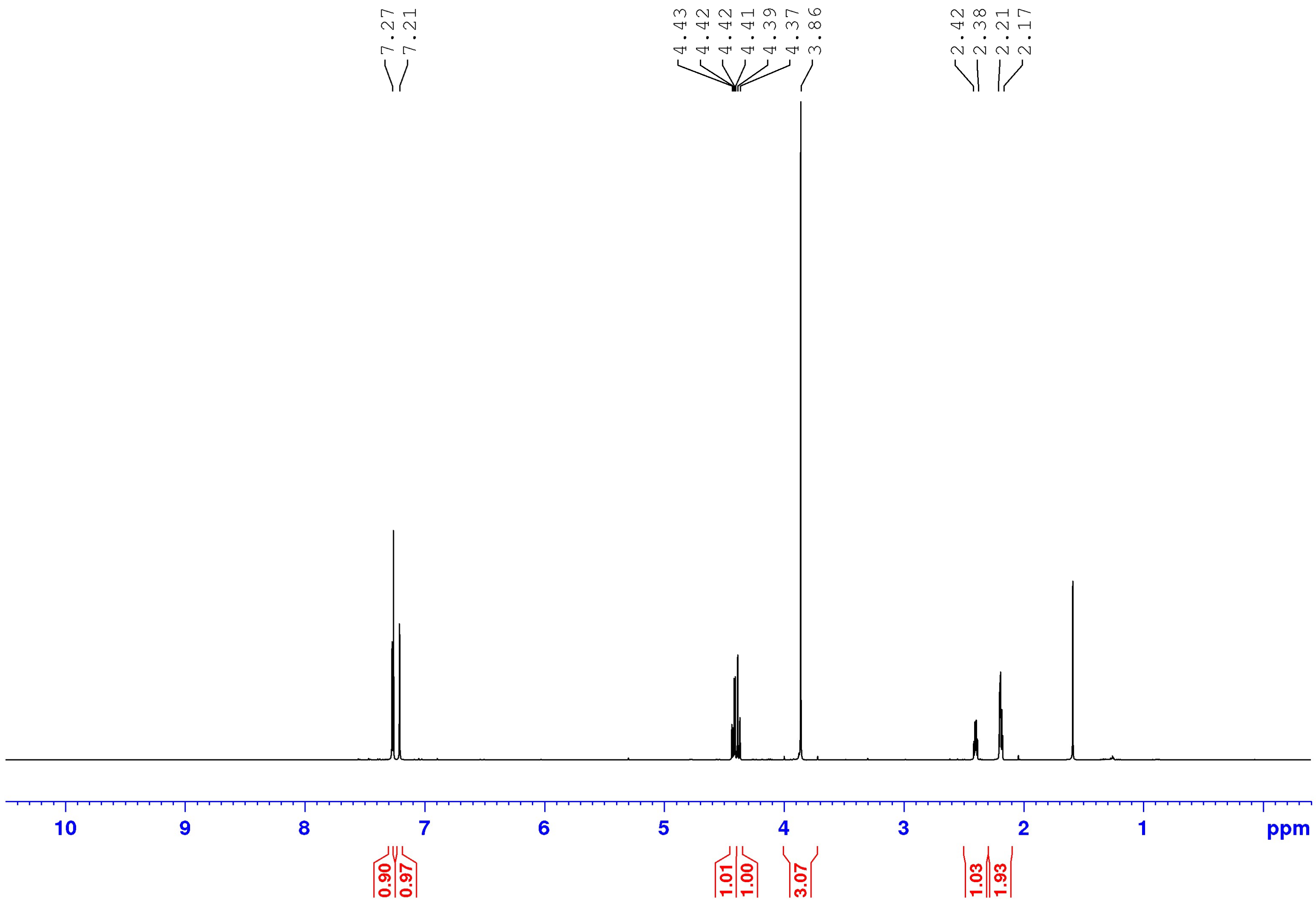

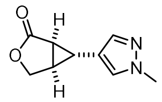

(–)-2d

Figure S64. ^{1}H NMR (500 MHz, $CDCl_3$) of **2d**

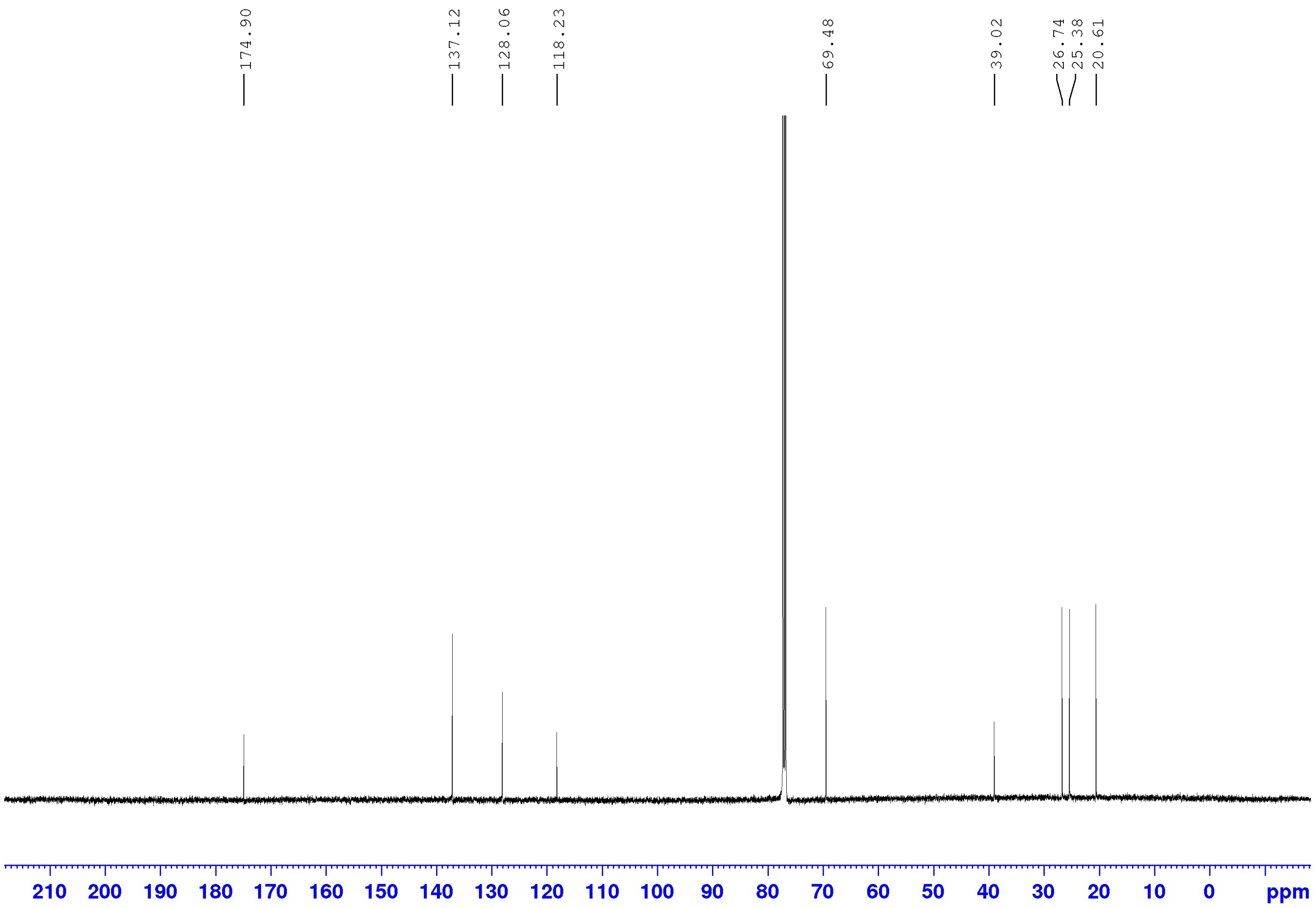

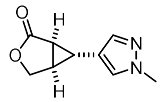

(–)-2d

Figure S65. $^{13}C\{^{1}H\}$ NMR (126 MHz, $CDCl_3$) of **2d**

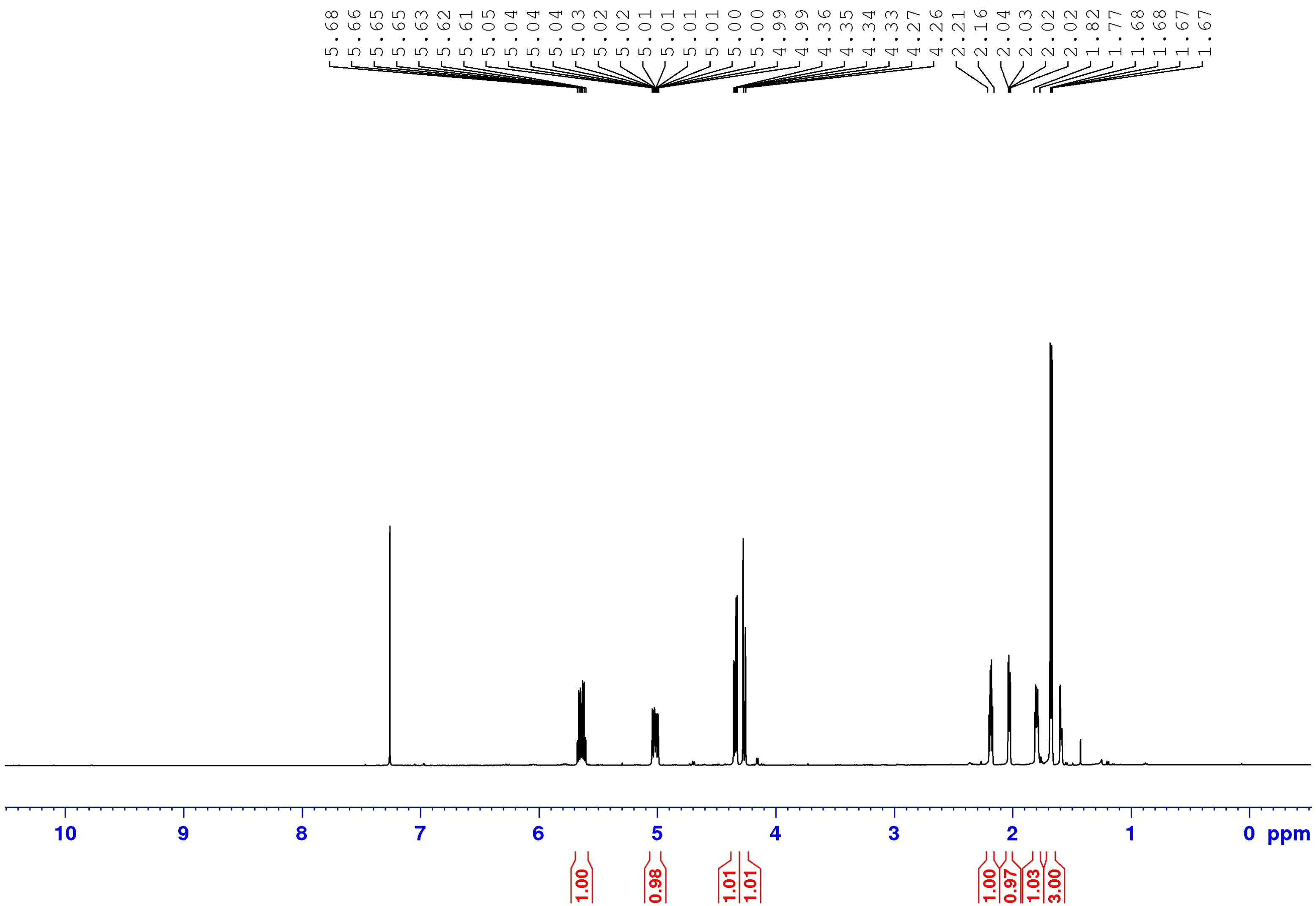

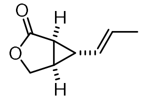

(–)-2e

Figure S66. ^{1}H NMR (500 MHz, $CDCl_3$) of **2e**

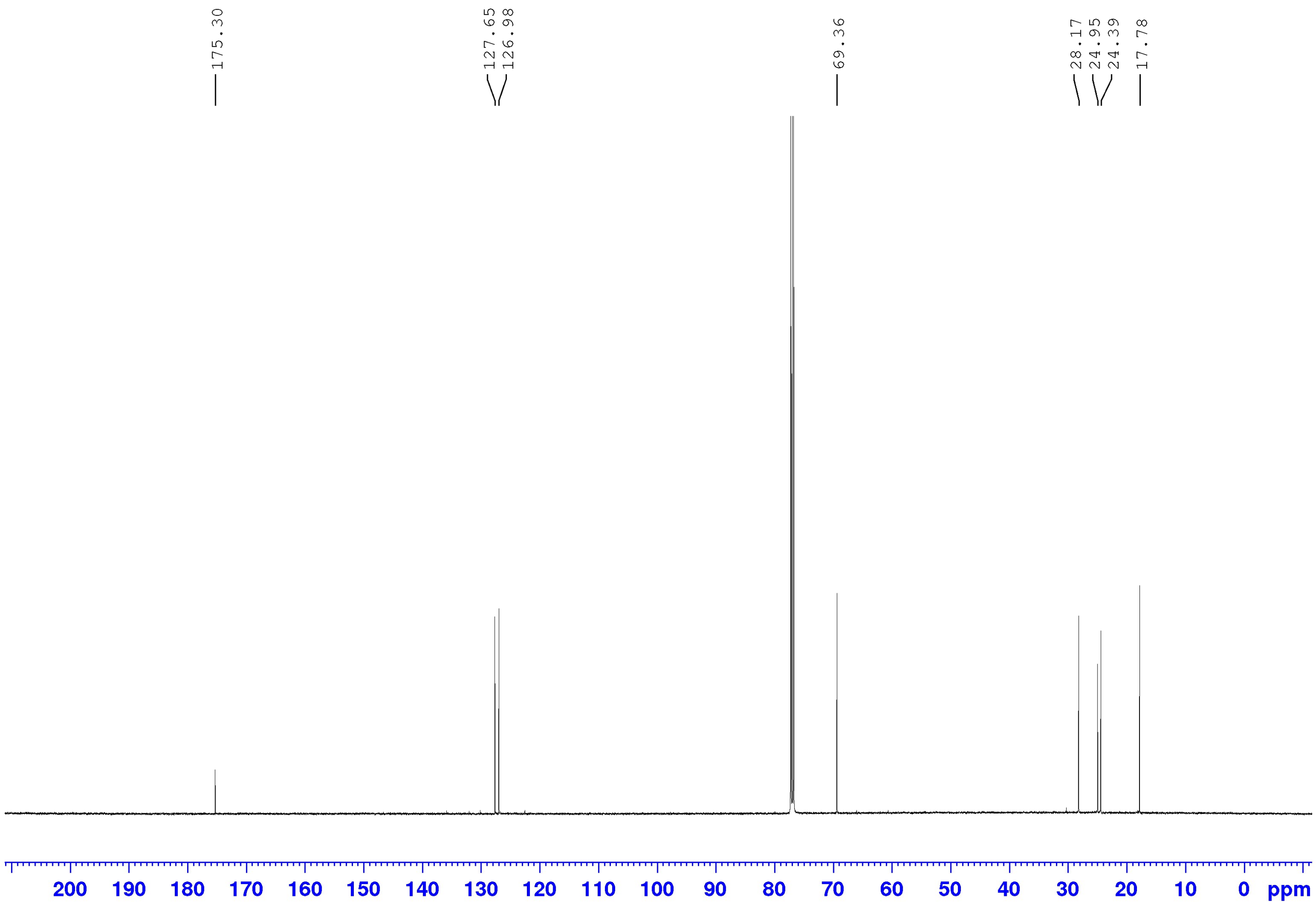

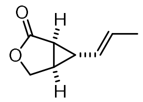

(–)-2e

Figure S67. $^{13}C\{^1H\}$ NMR (126 MHz, $CDCl_3$) of **2e**

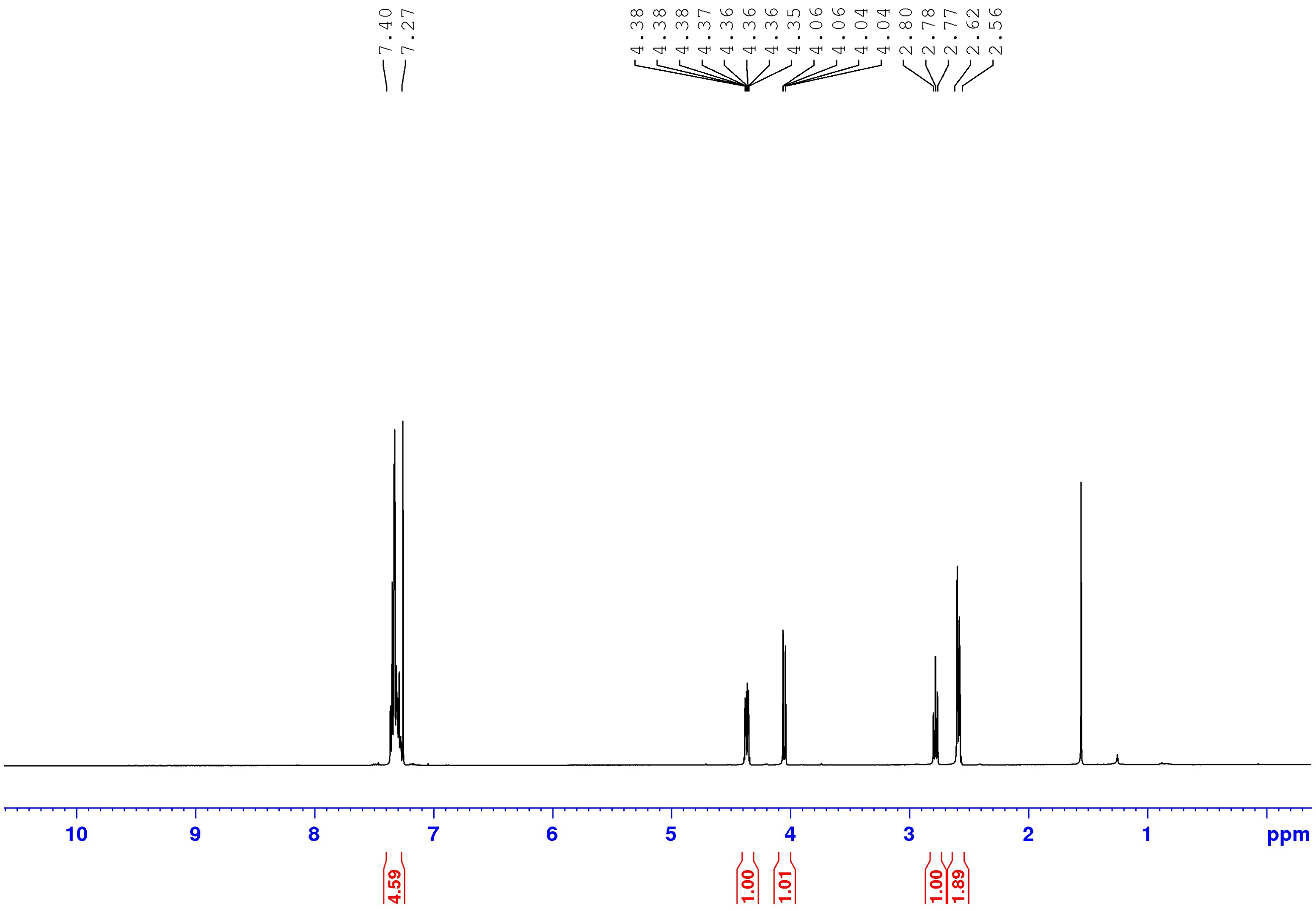

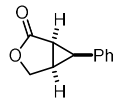

(+)-2f

Figure S68. ^{1}H NMR (500 MHz, $CDCl_3$) of **2f**

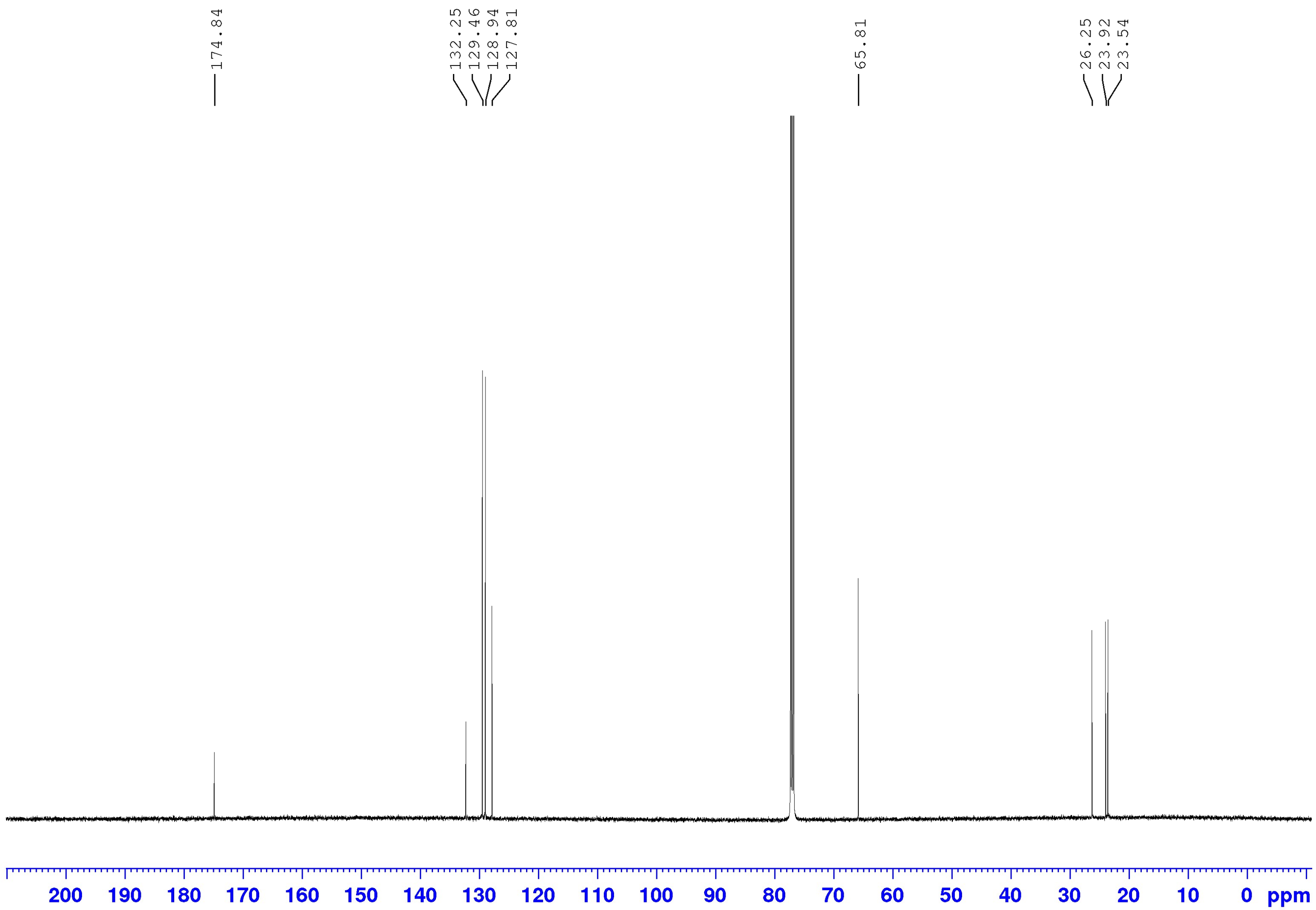

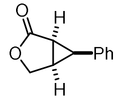

(+)-2f

Figure S69. $^{13}C\{^1H\}$ NMR (126 MHz, $CDCl_3$) of **2f**

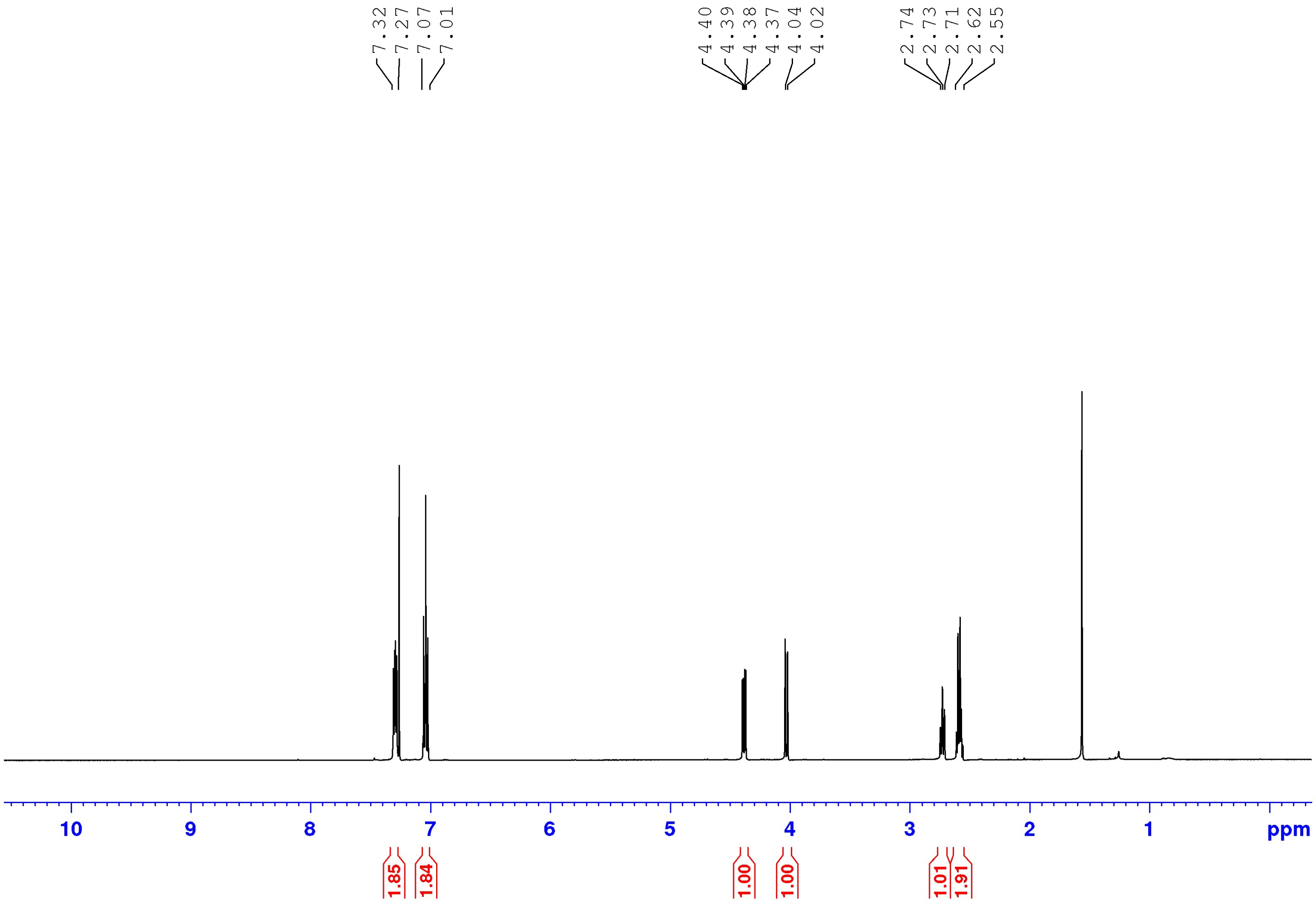

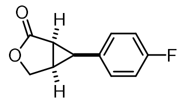

(+)-2g

Figure S70. ^{1}H NMR (500 MHz, $CDCl_3$) of **2g**

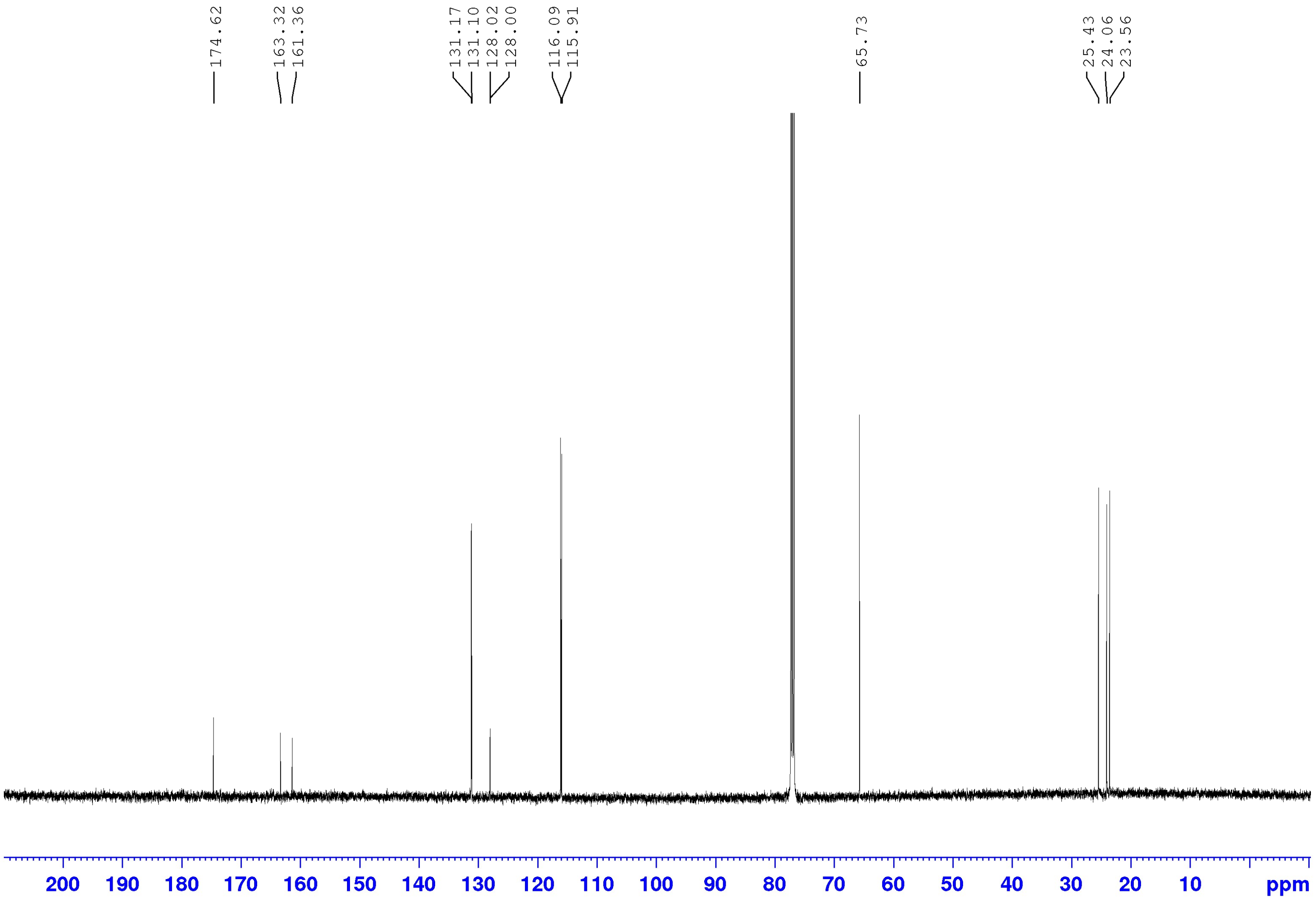

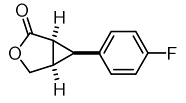

(+)-2g

Figure S71. $^{13}C\{^{1}H\}$ NMR (126 MHz, $CDCl_3$) of **2g**

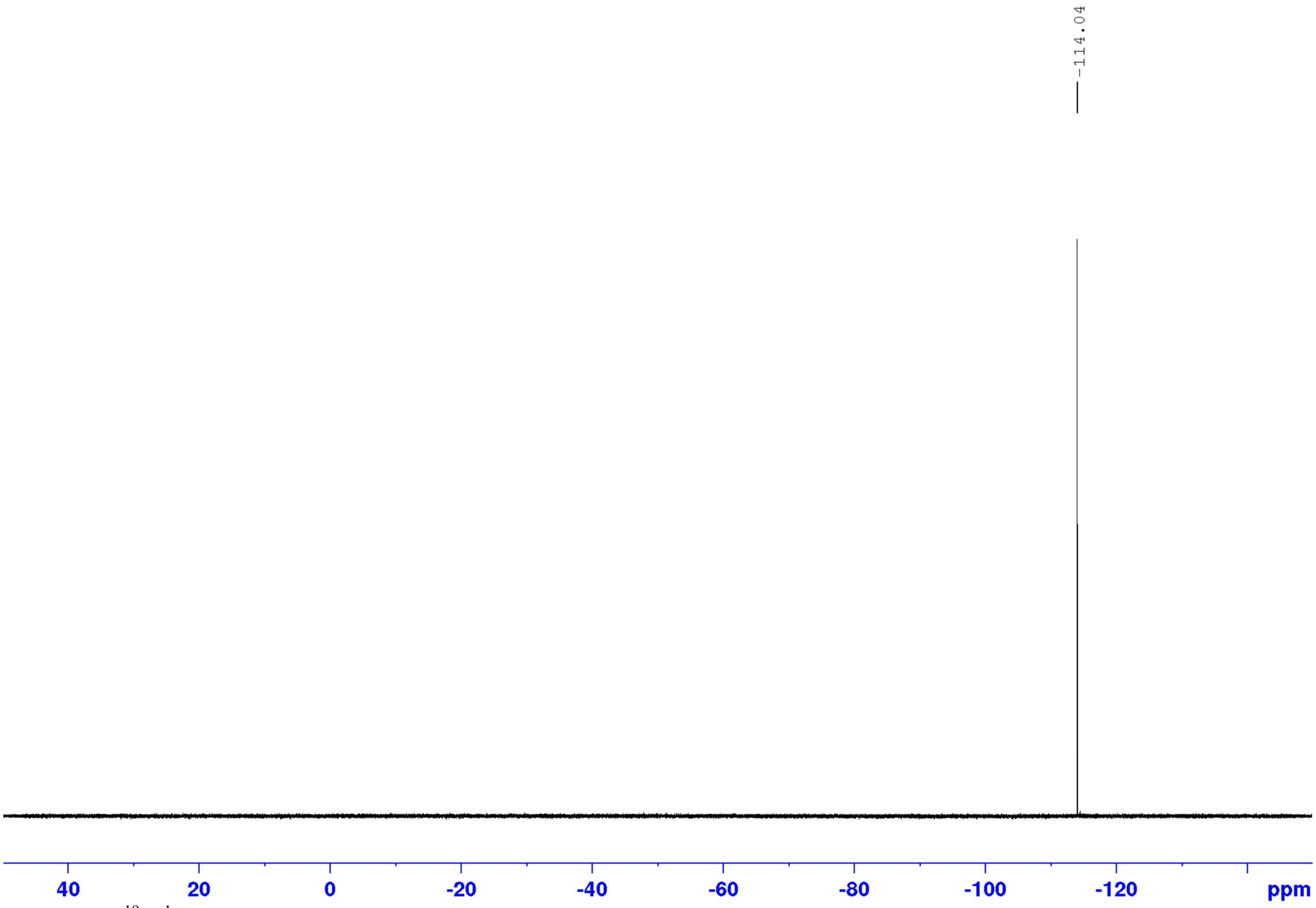

Figure S72. $^{19}F\{^{1}H\}$ NMR (470MHz, $CDCl_3$) of **2g**

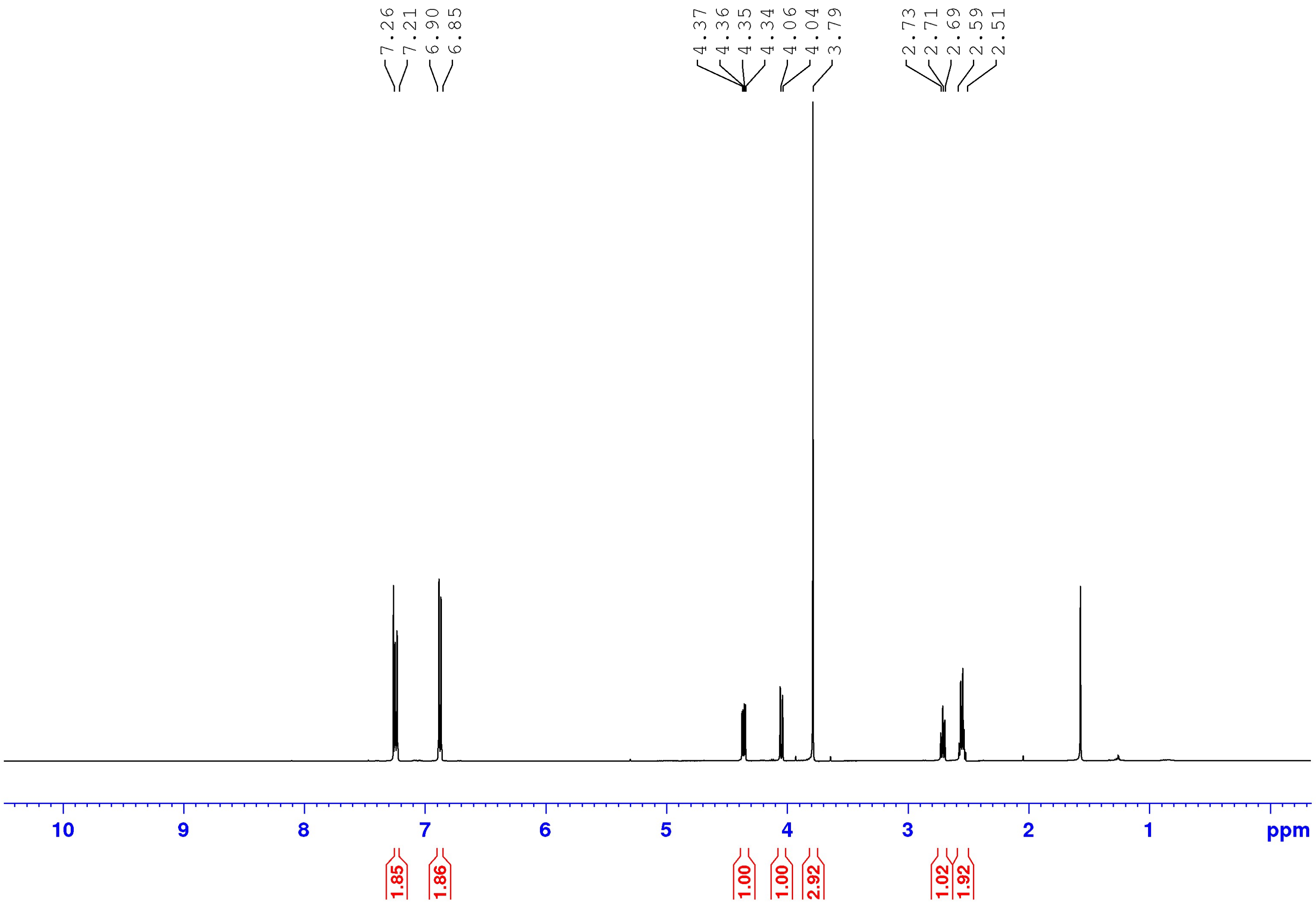

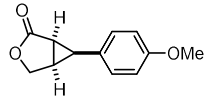

(+)-2h

Figure S73. ^{1}H NMR (500 MHz, $CDCl_3$) of **2h**

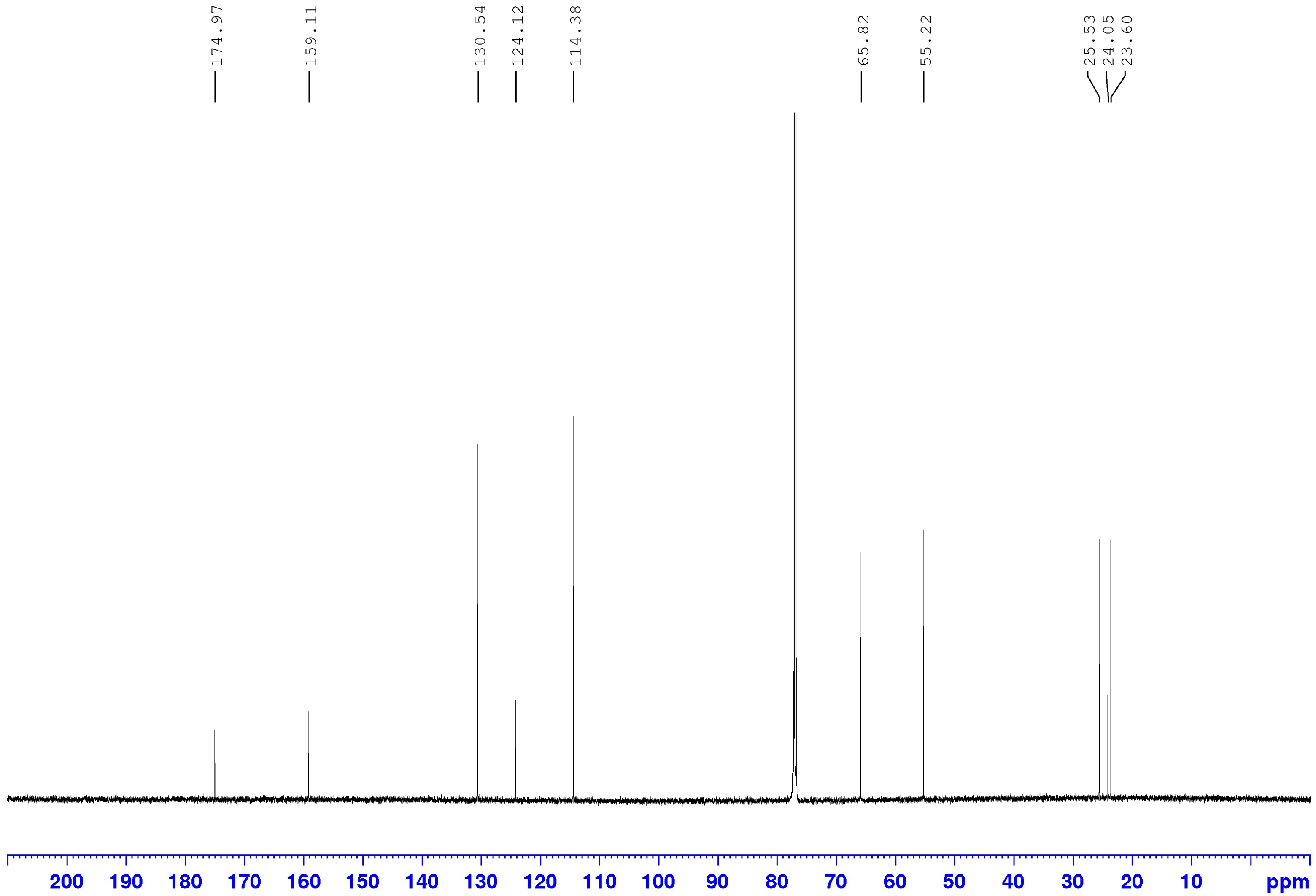

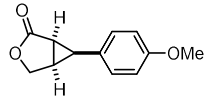

(+)-2h

Figure S74. $^{13}C\{^{1}H\}$ NMR (126 MHz, $CDCl_3$) of **2h**

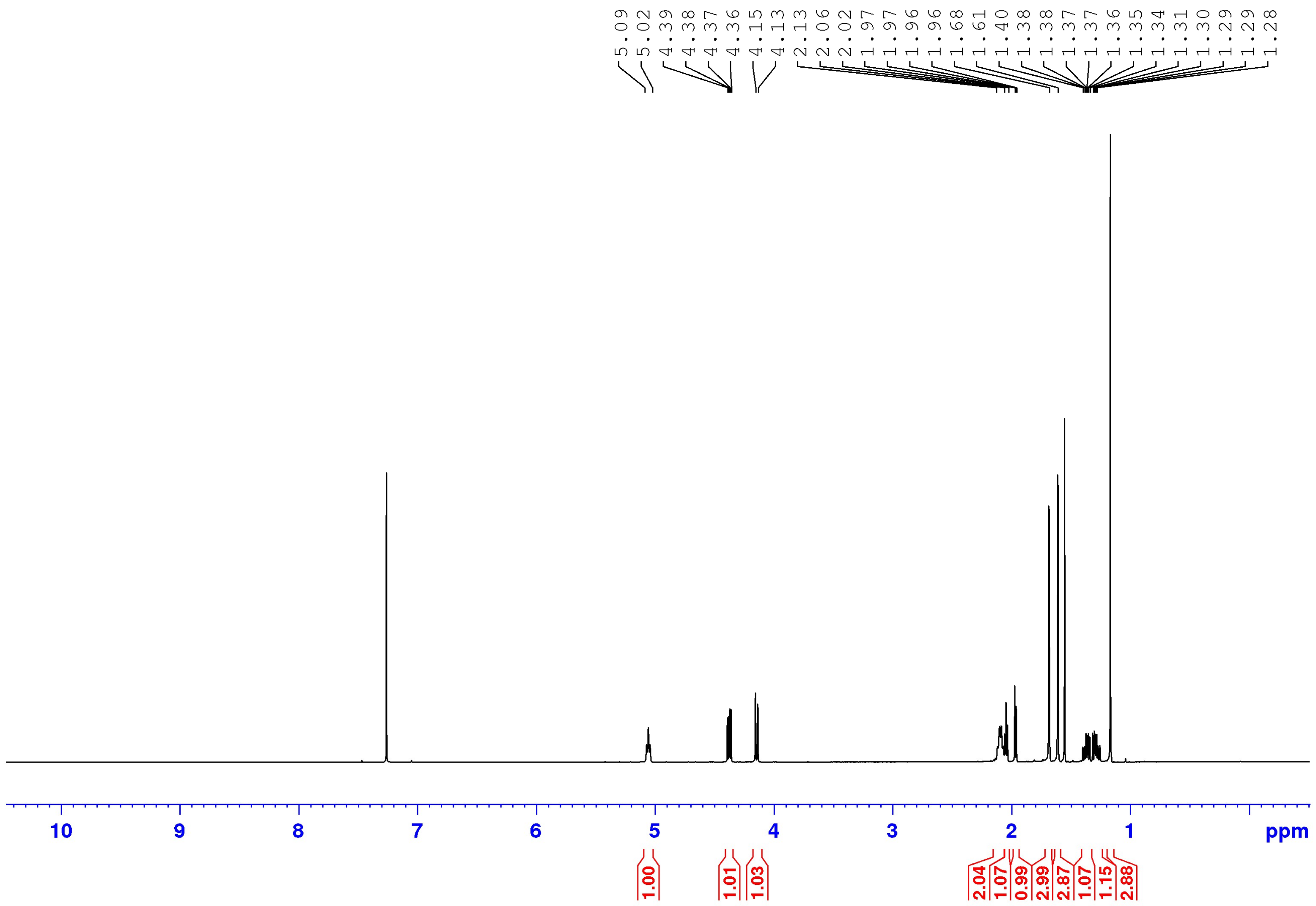

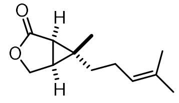
(−)-2i

Figure S75. ^{1}H NMR (500 MHz, $CDCl_3$) of **2i**

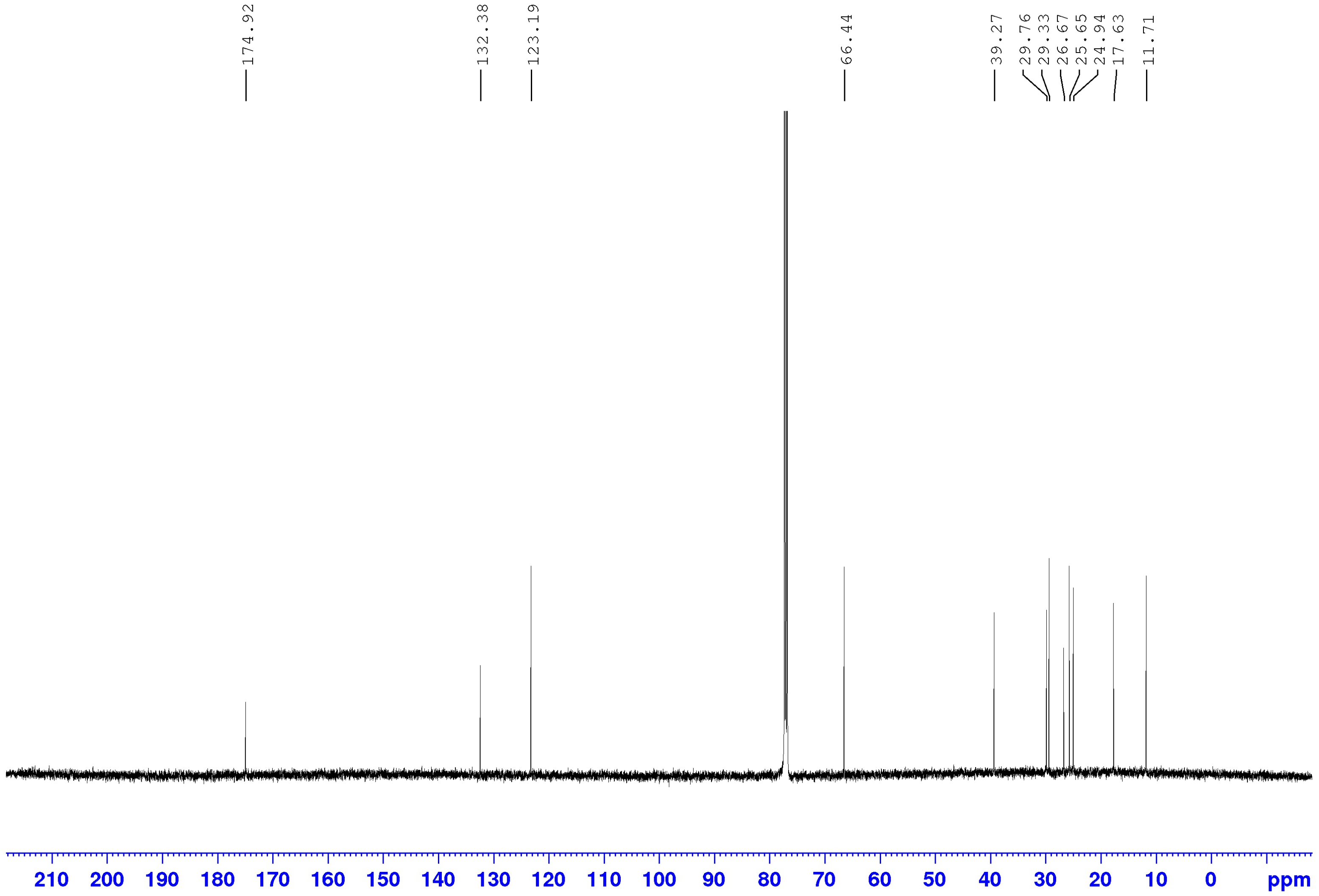

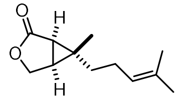

(–)-2i

Figure S76. $^{13}C\{^{1}H\}$ NMR (126 MHz, $CDCl_3$) of **2i**

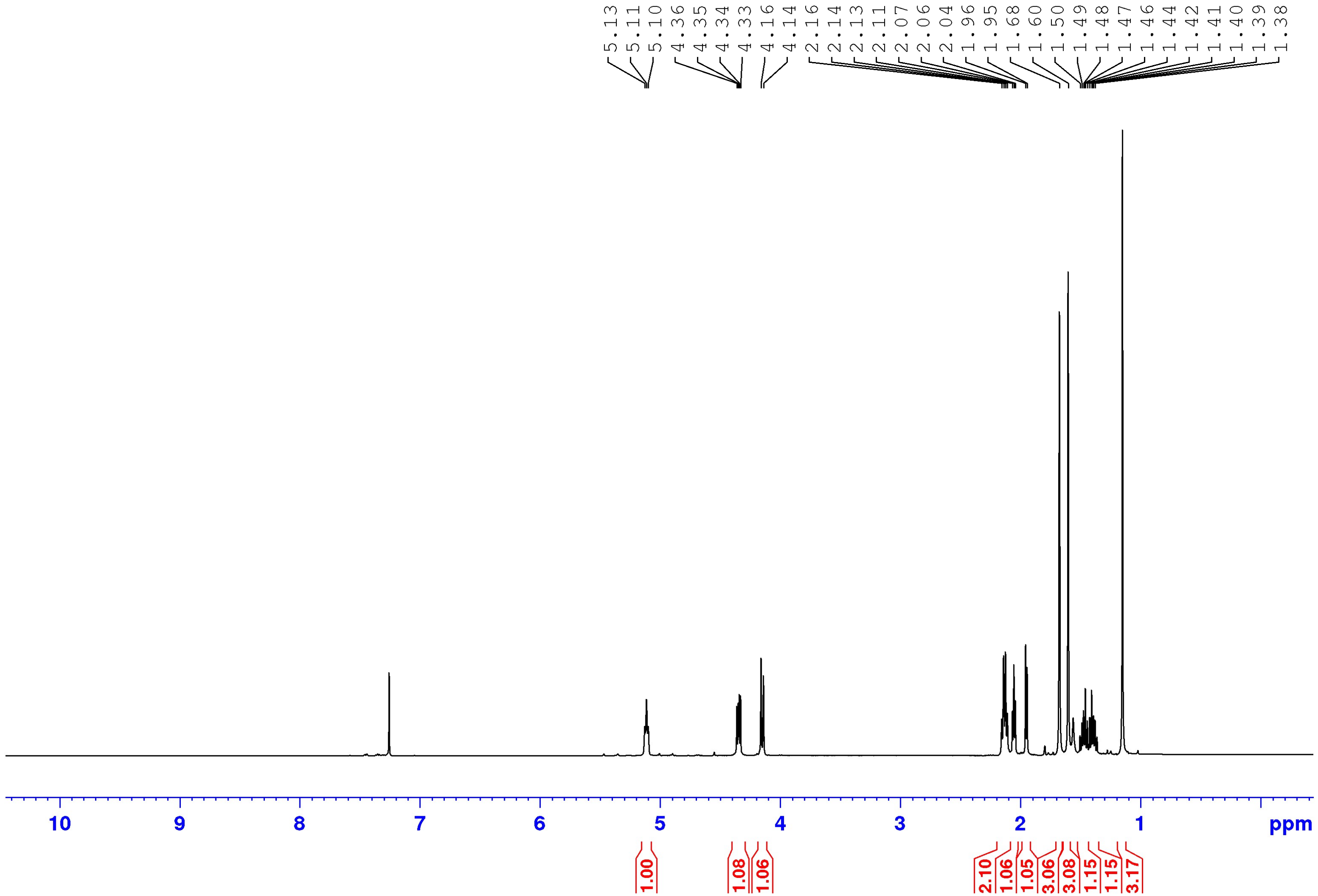

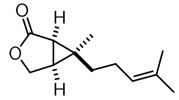

(−)-2j

Figure S77. ^{1}H NMR (500 MHz, $CDCl_3$) of **2j**

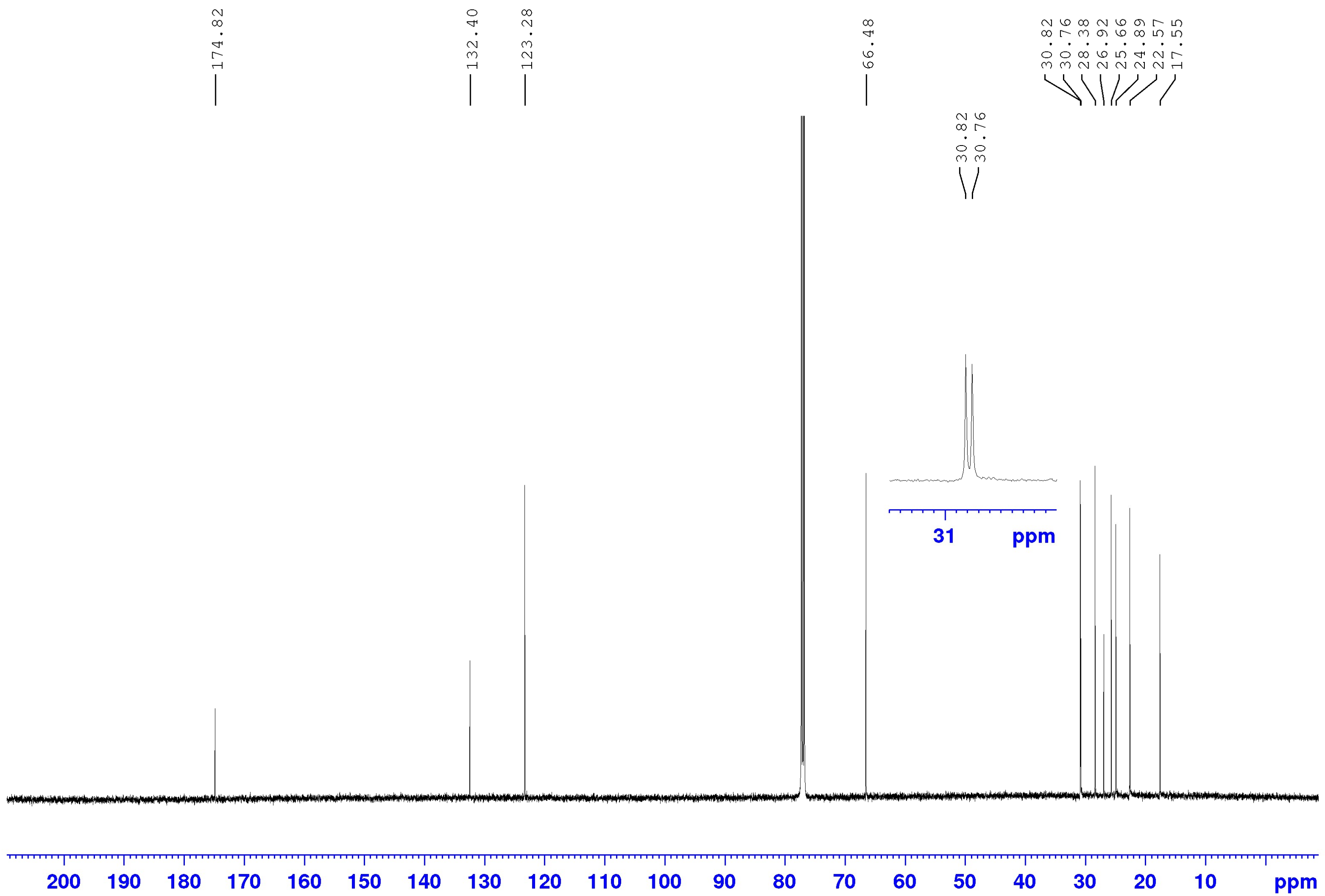

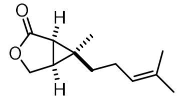

(–)-2j

Figure S78. $^{13}C\{^1H\}$ NMR (126 MHz, $CDCl_3$) of **2j**

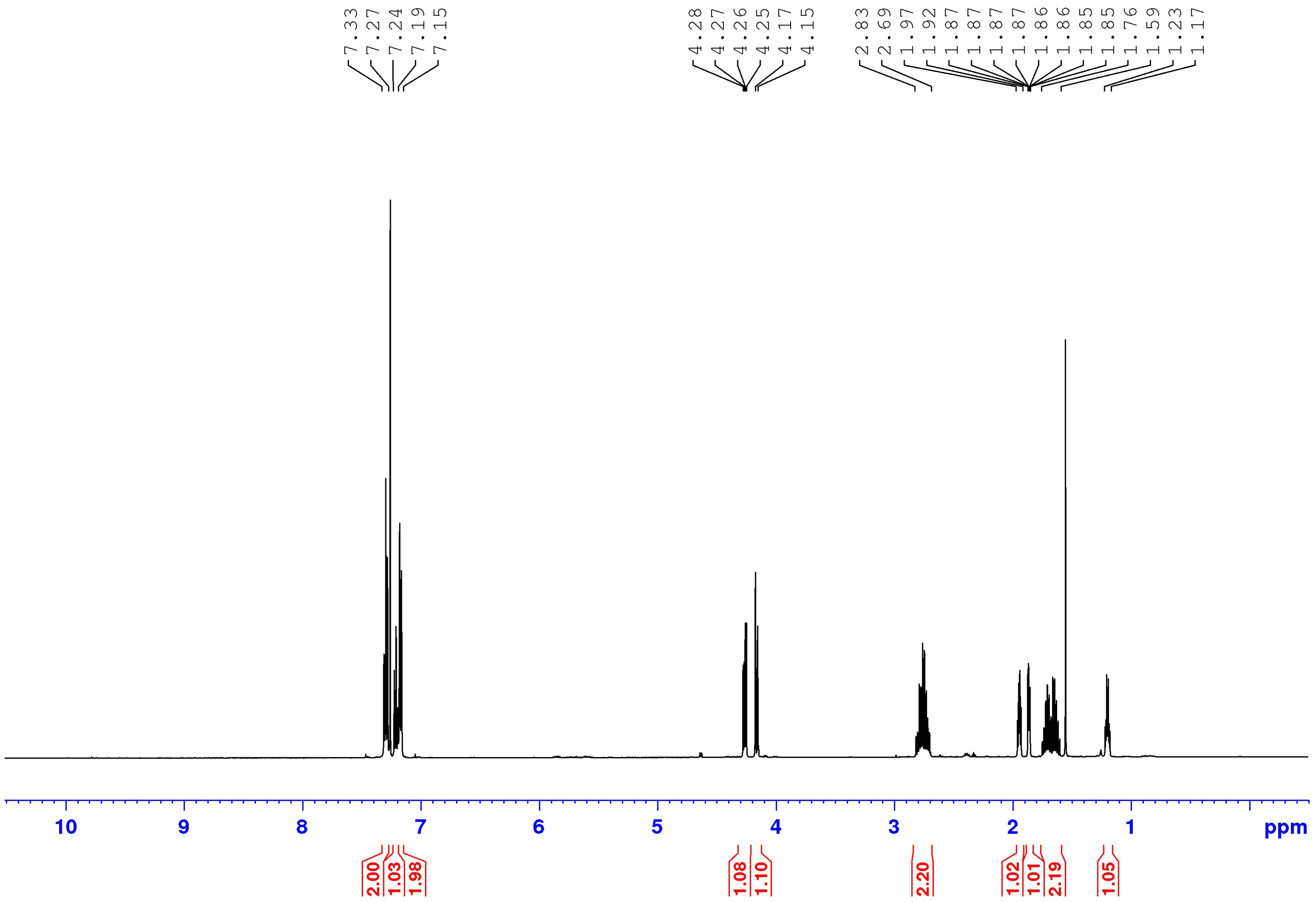

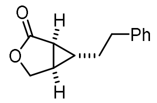

(–)-2k

Figure S79. ^{1}H NMR (500 MHz, $CDCl_3$) of **2k**

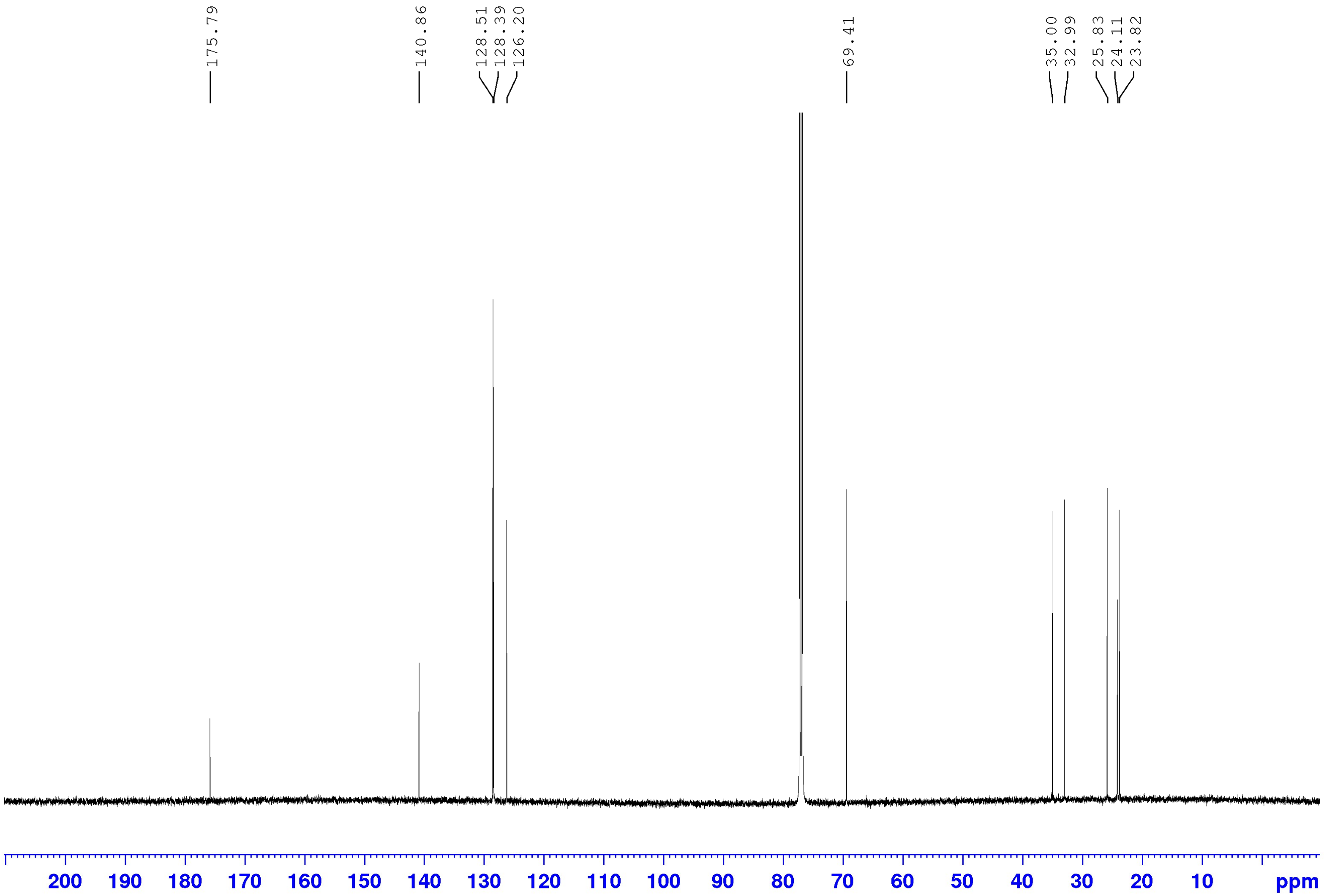

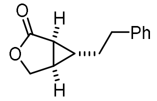

(–)-2k

Figure S80. $^{13}C\{^1H\}$ NMR (126 MHz, $CDCl_3$) of **2k**

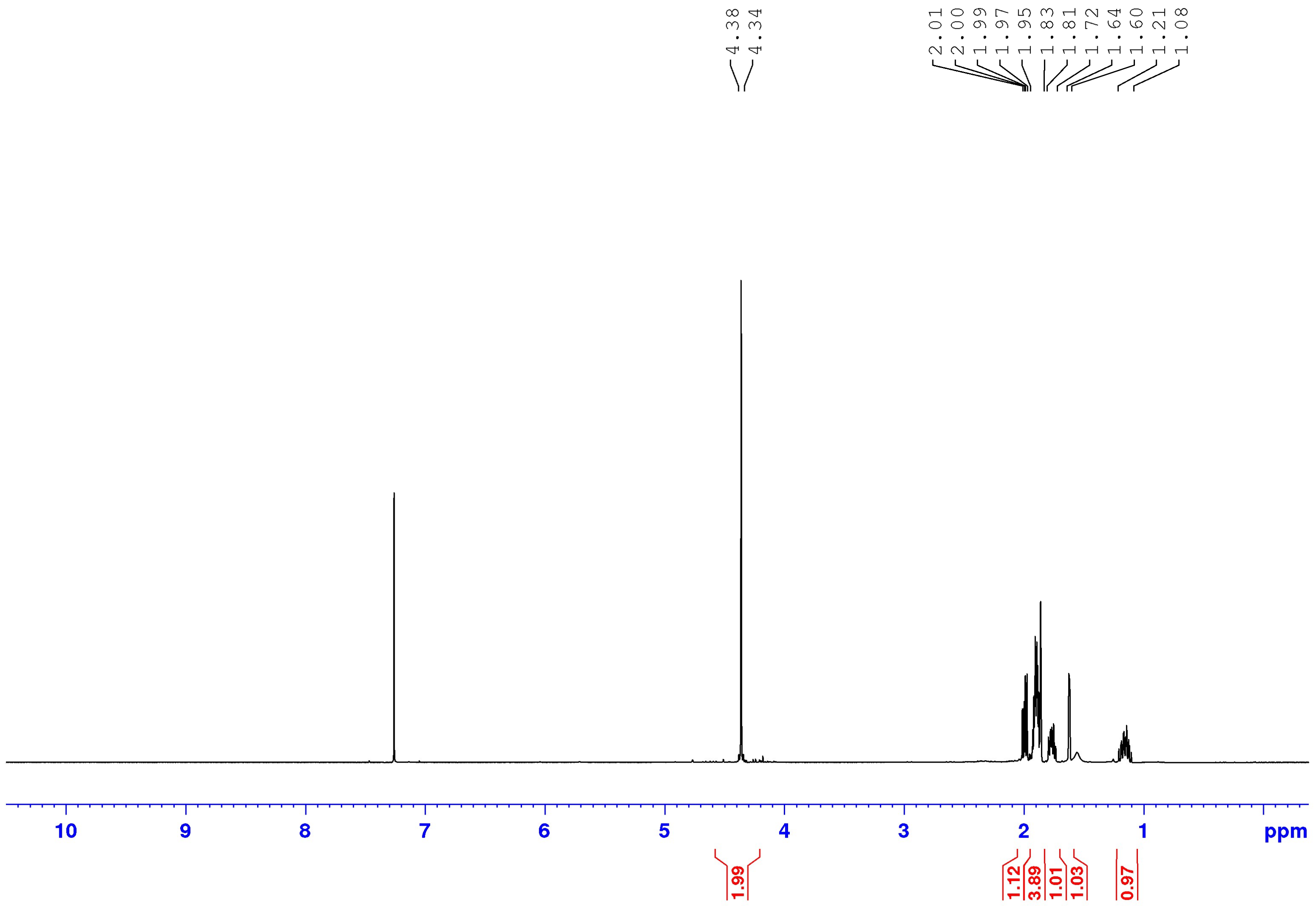

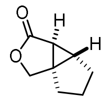

(+)-2l

Figure S81. ^{1}H NMR (500 MHz, $CDCl_3$) of **2l**

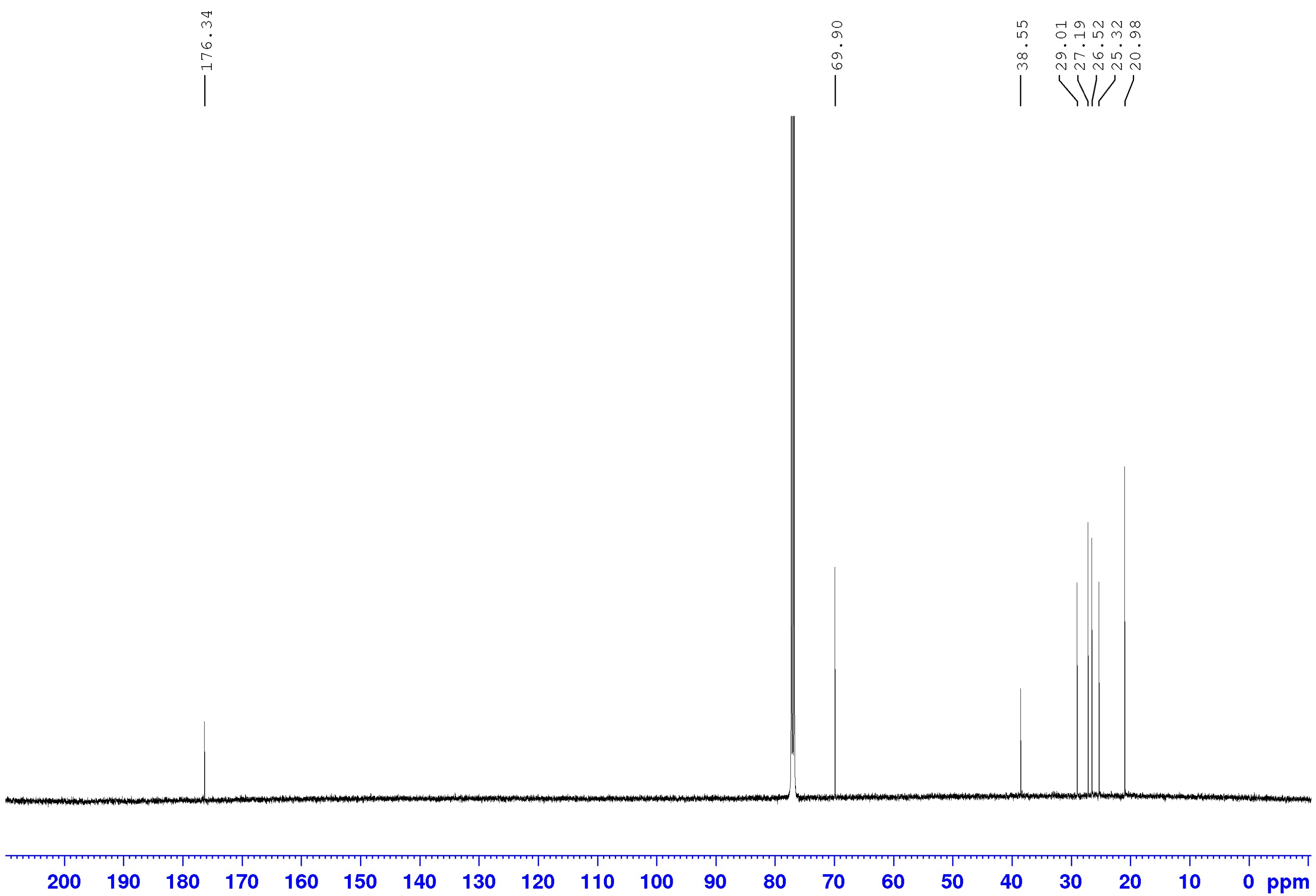

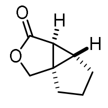

(+)-2l

Figure S82. $^{13}C\{^{1}H\}$ NMR (126 MHz, $CDCl_3$) of **2l**

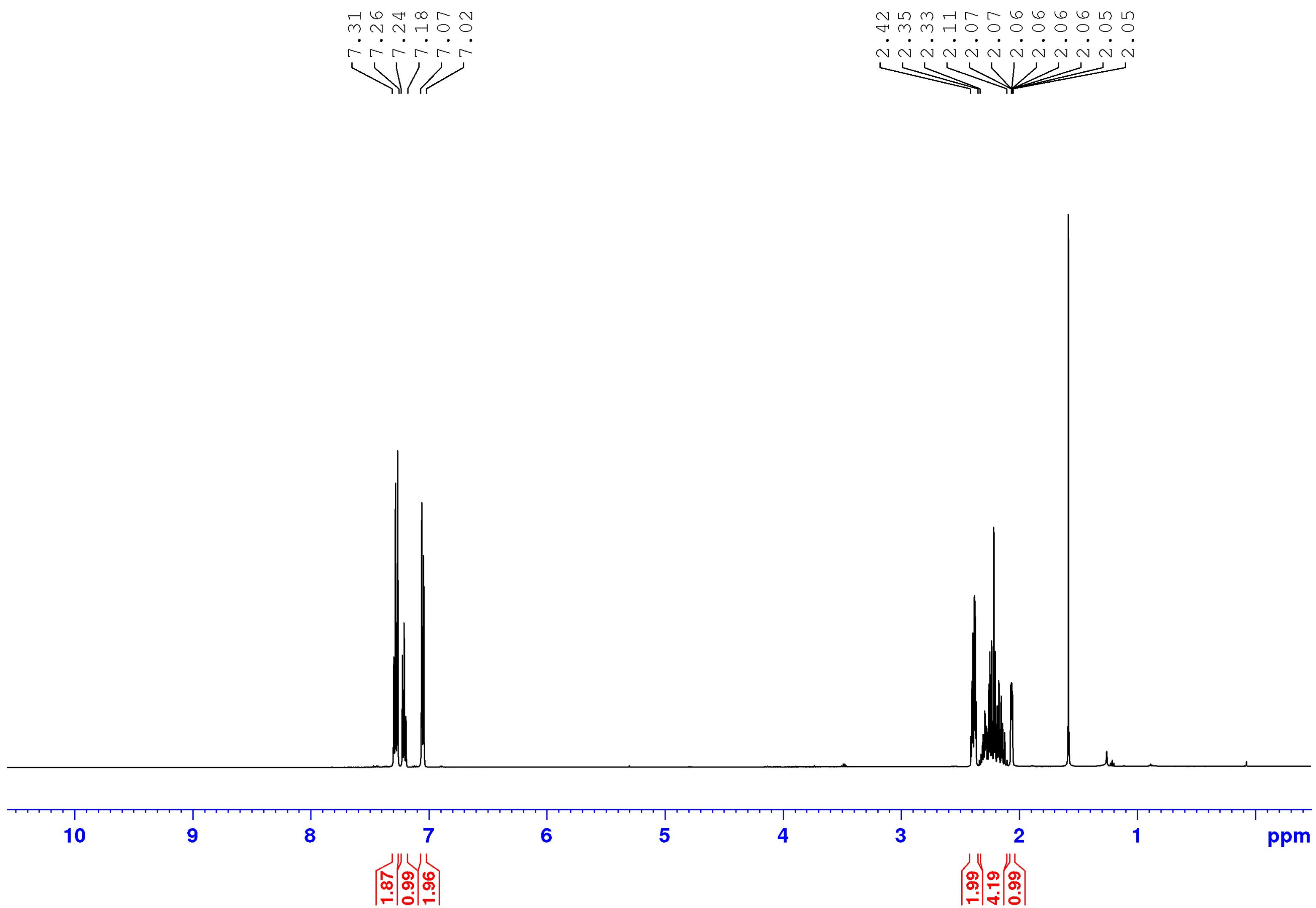

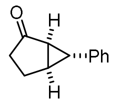

(–)-2m

Figure S83. ^{1}H NMR (500 MHz, $CDCl_3$) of **2m**

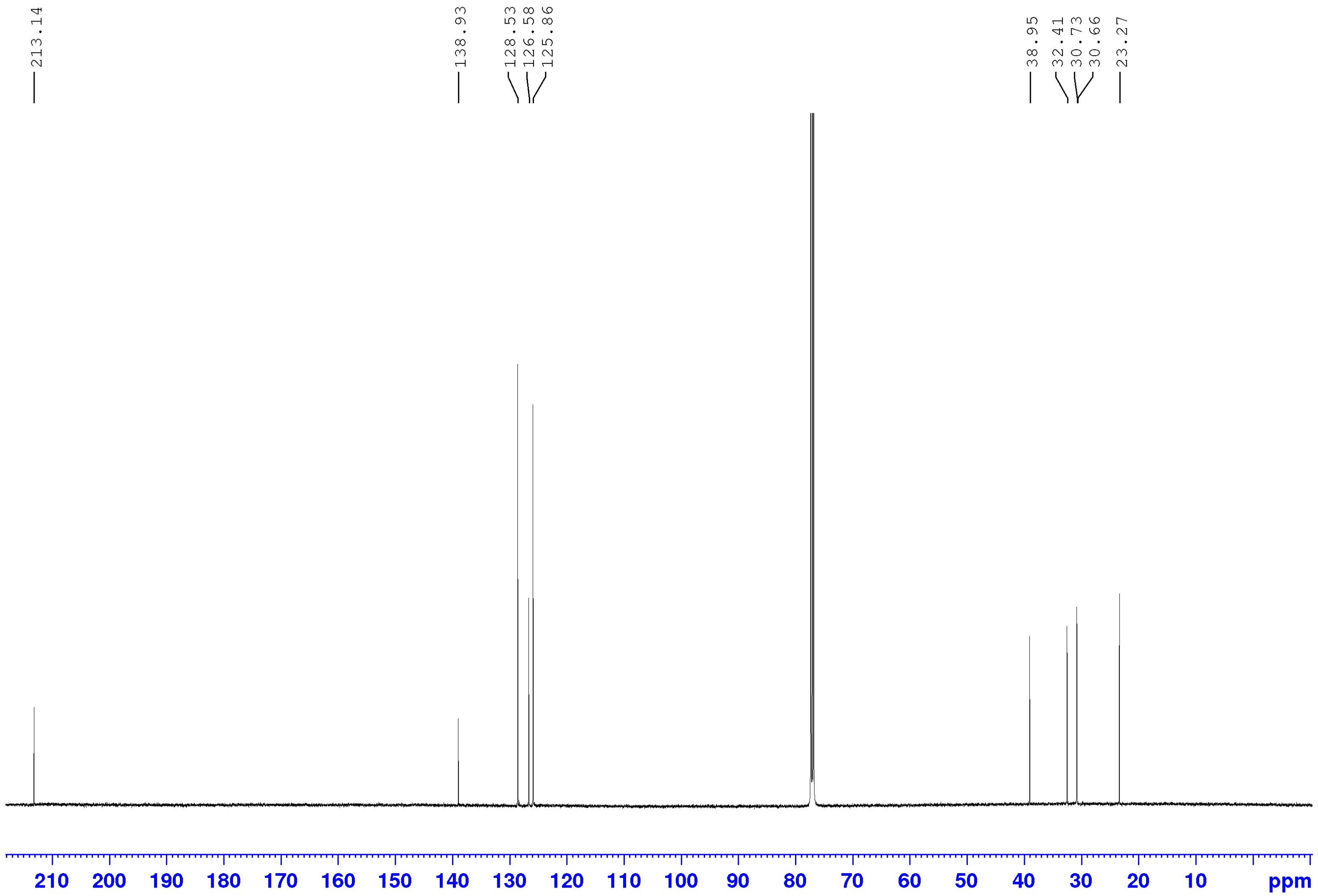

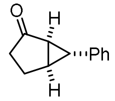

(–)-2m

Figure S84. $^{13}C\{^{1}H\}$ NMR (126 MHz, $CDCl_3$) of **2m**

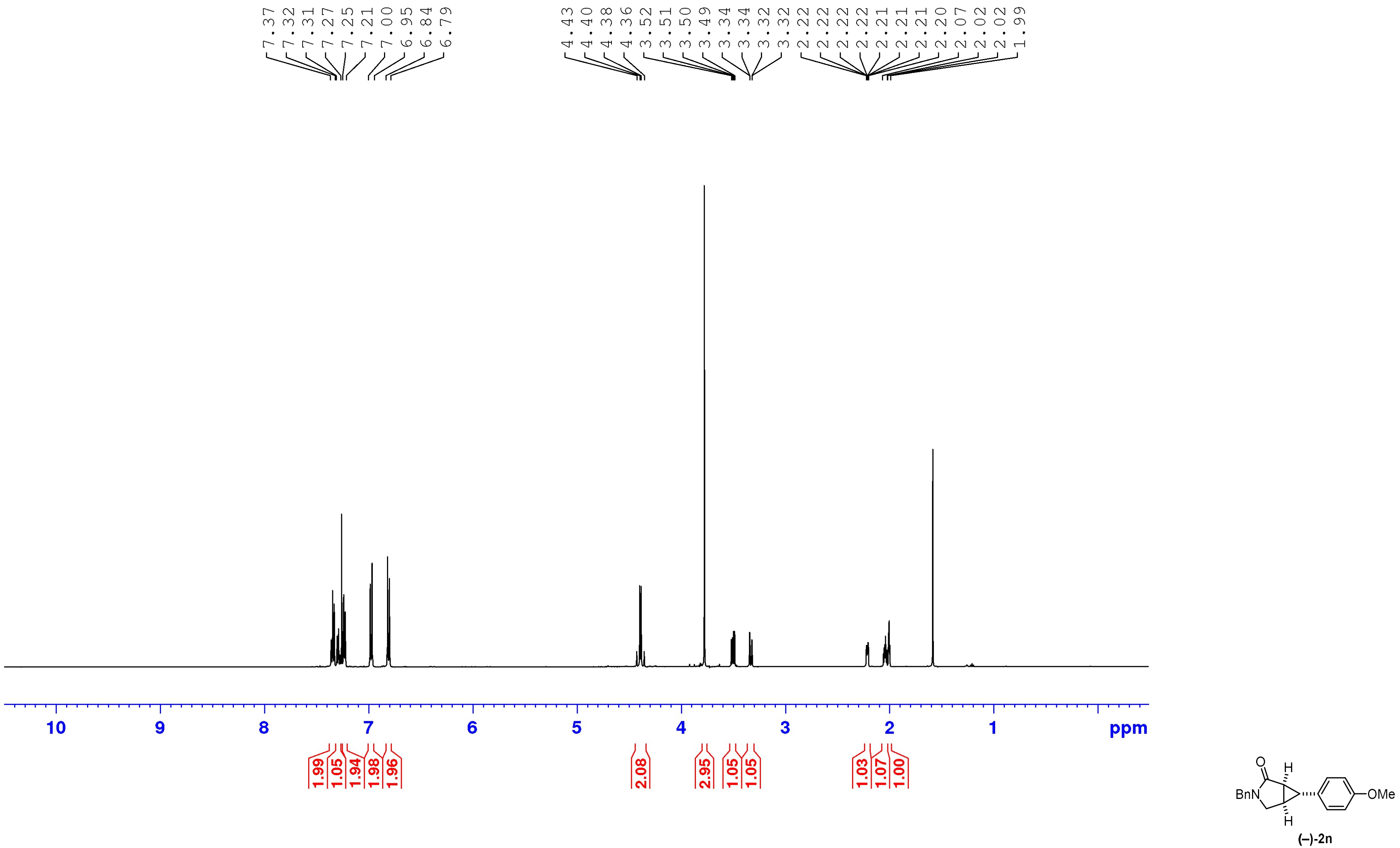

Figure S85. ^{1}H NMR (500 MHz, $CDCl_3$) of **2n**

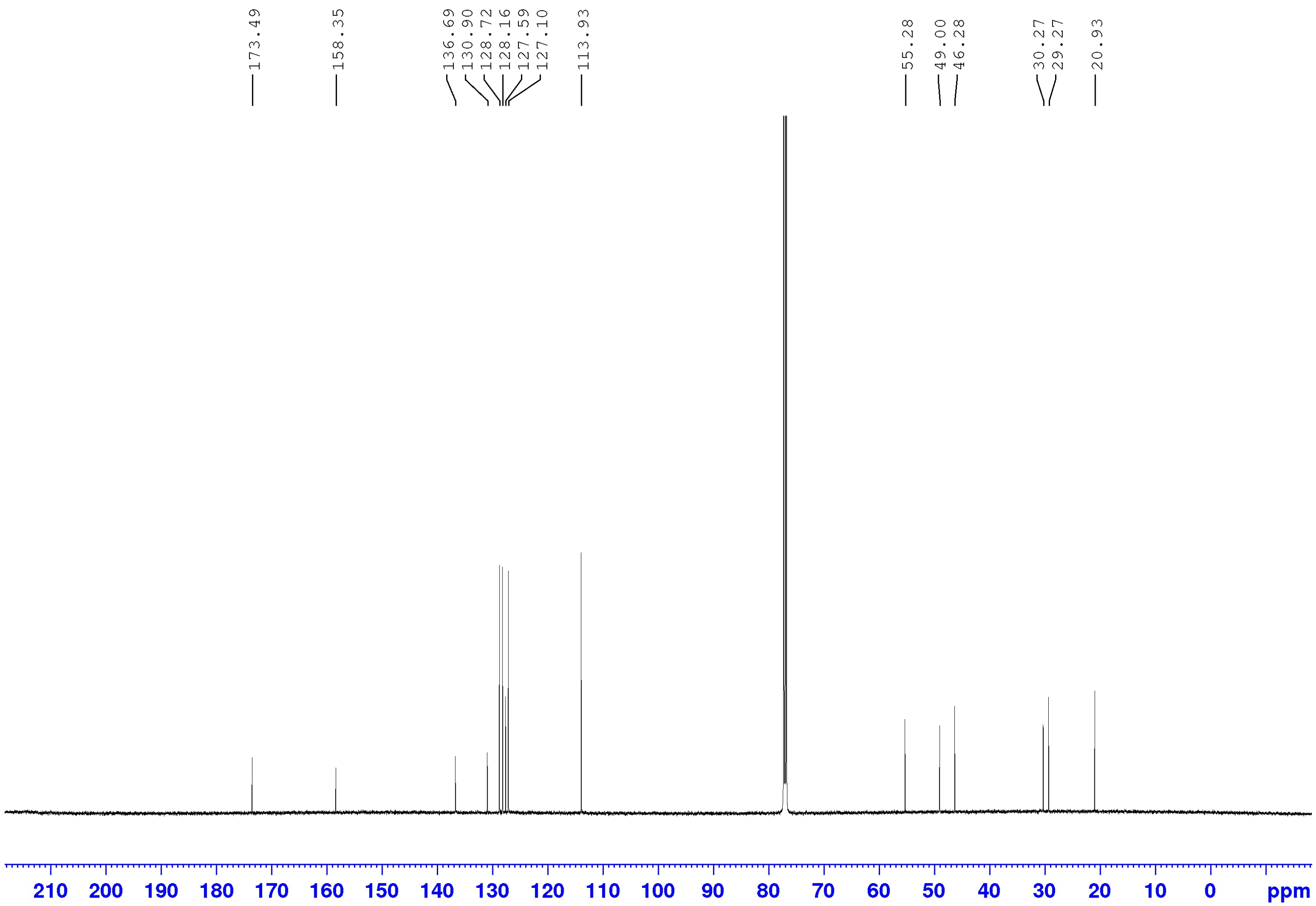

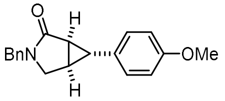

(–)-2n

Figure S86. $^{13}C\{^1H\}$ NMR (126 MHz, $CDCl_3$) of **2n**

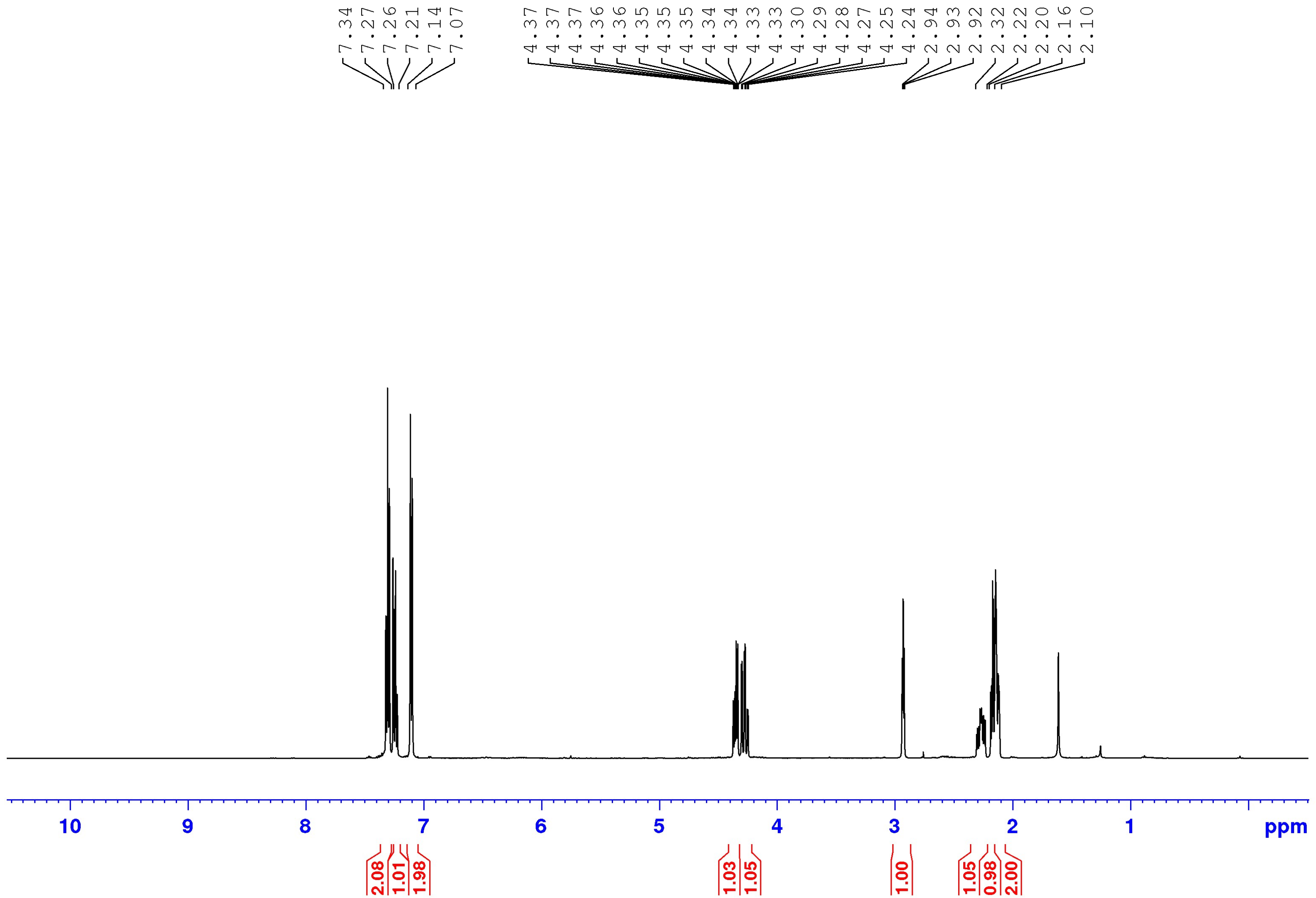

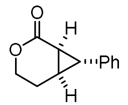

(–)-2o

Figure S87. ^{1}H NMR (500 MHz, $CDCl_3$) of **2o**

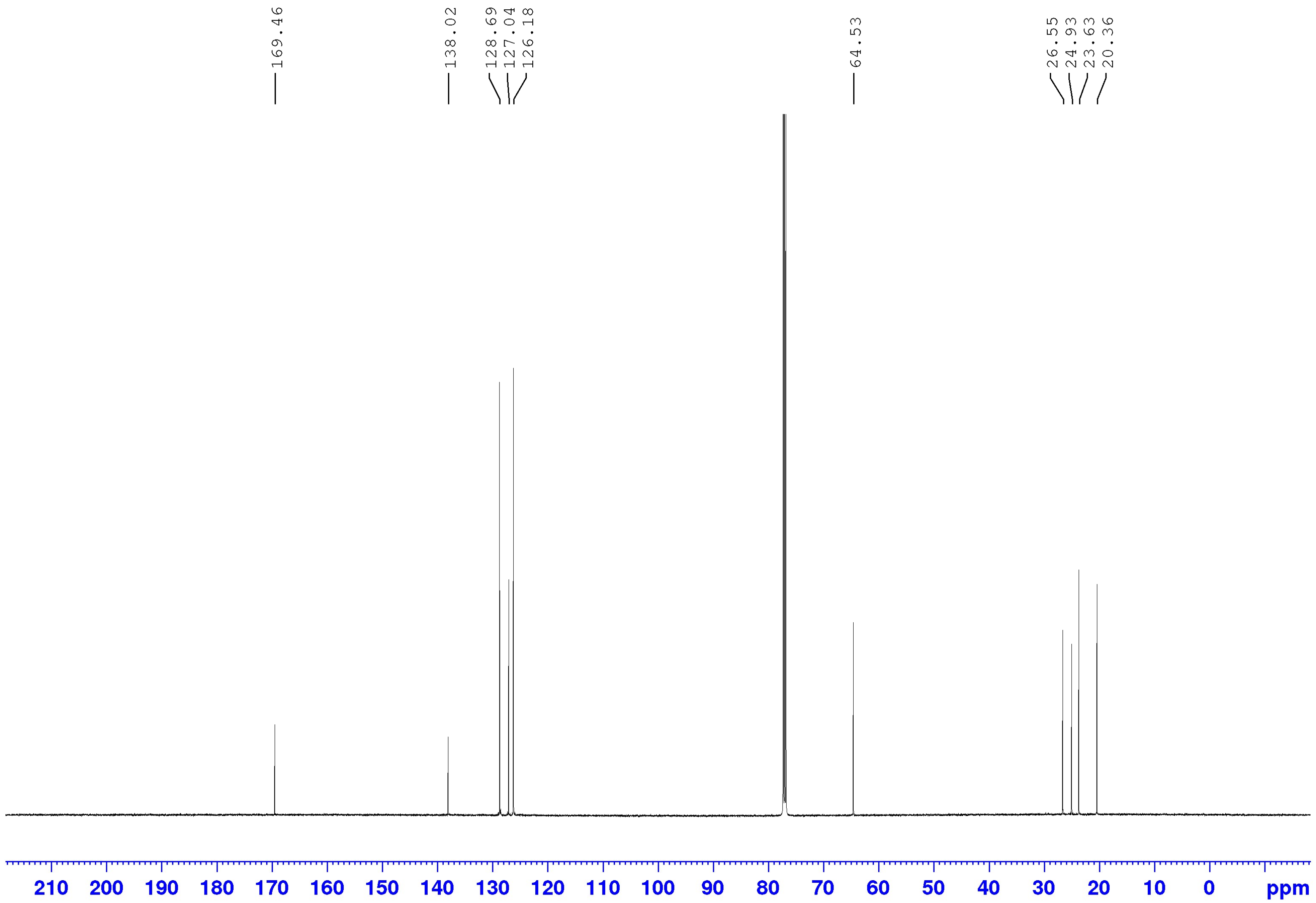

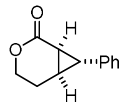

(–)-2o

Figure S88. $^{13}C\{^{1}H\}$ NMR (126 MHz, $CDCl_3$) of **2o**

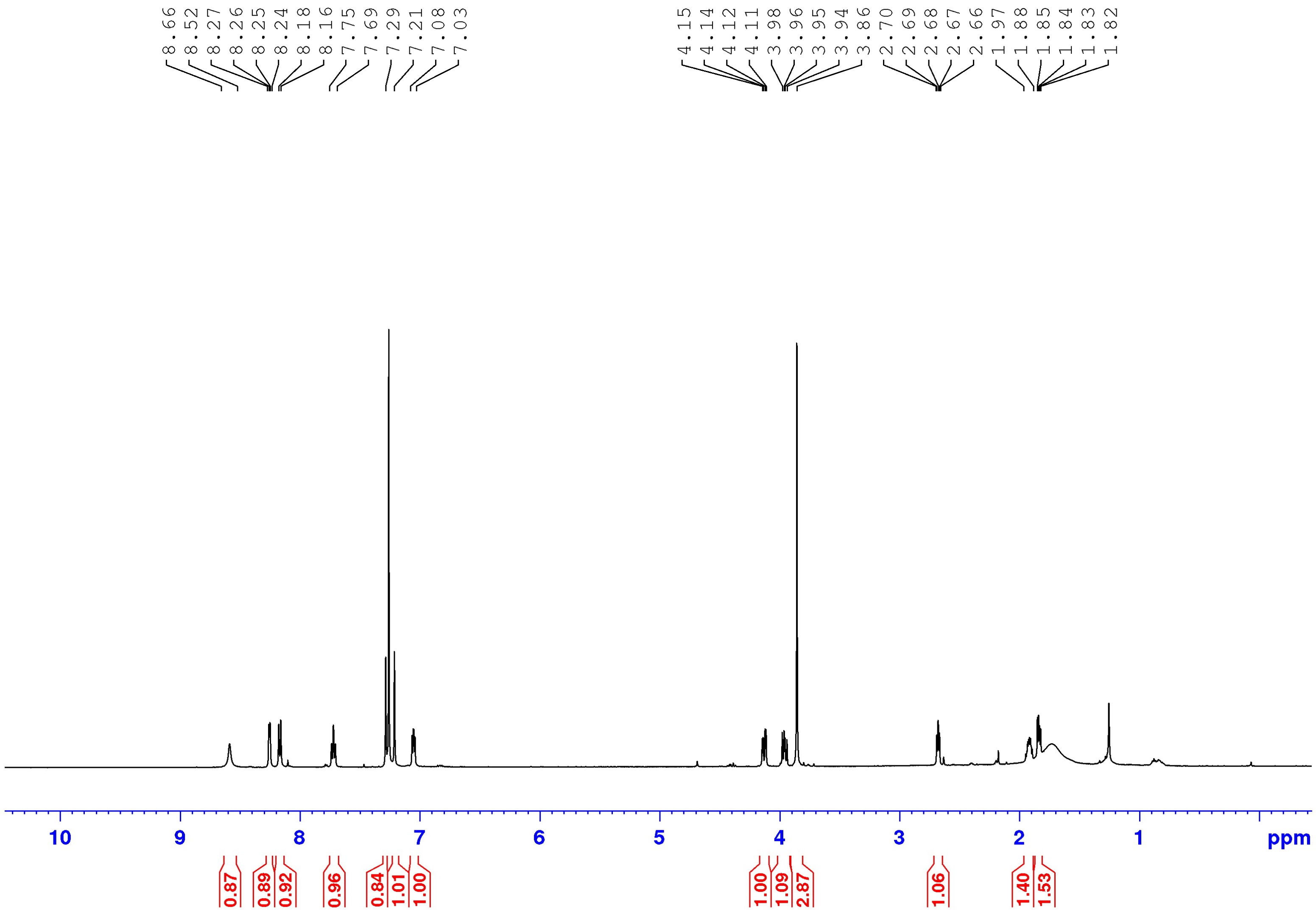

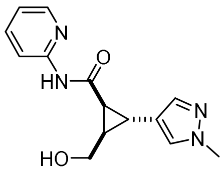

(±)-**9**

Figure S89. ^{1}H NMR (500 MHz, $CDCl_3$) of **9**

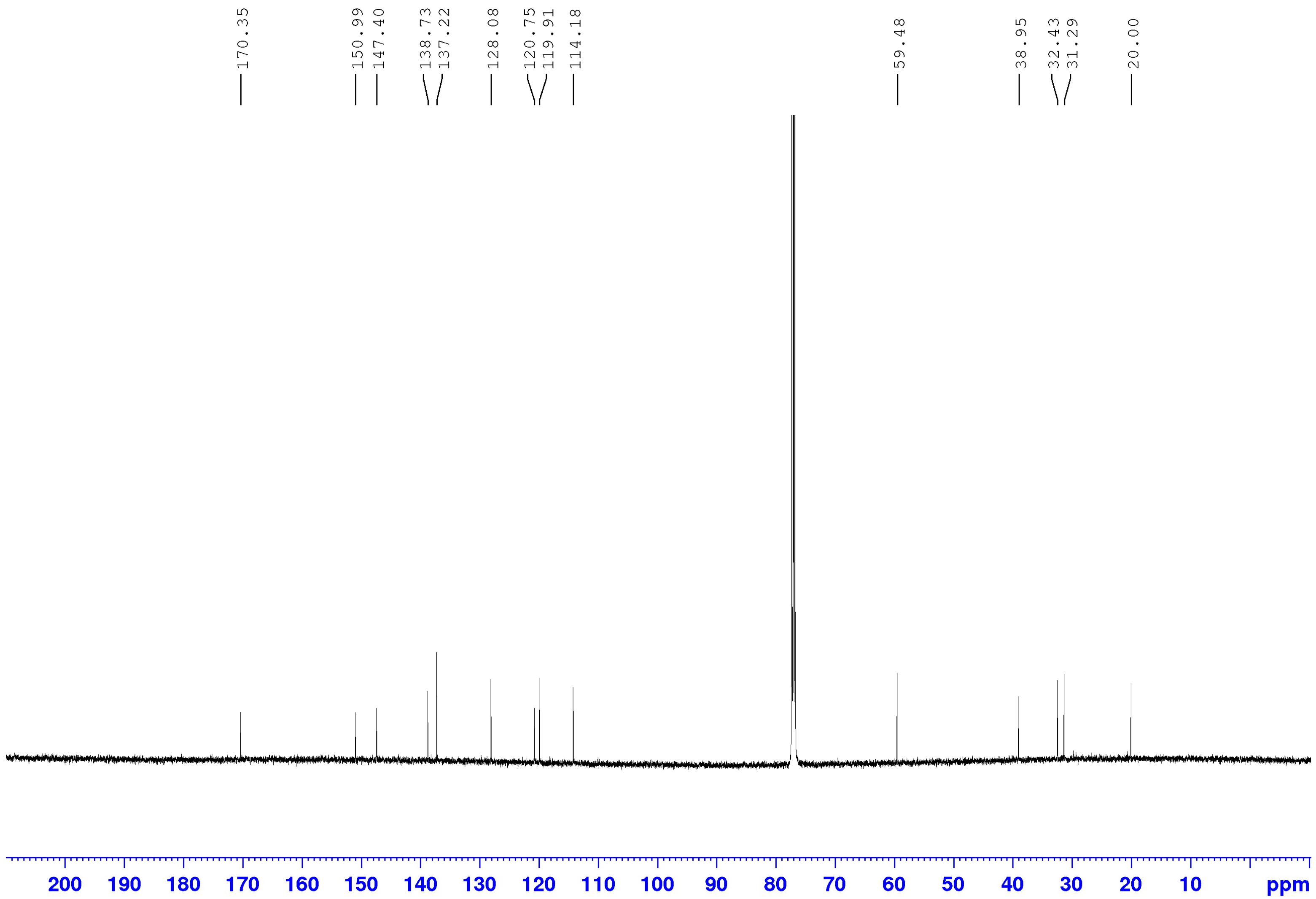

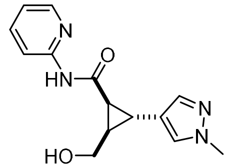

(±)-**9**

Figure S90. $^{13}C\{^{1}H\}$ NMR (126 MHz, $CDCl_3$) of **9**

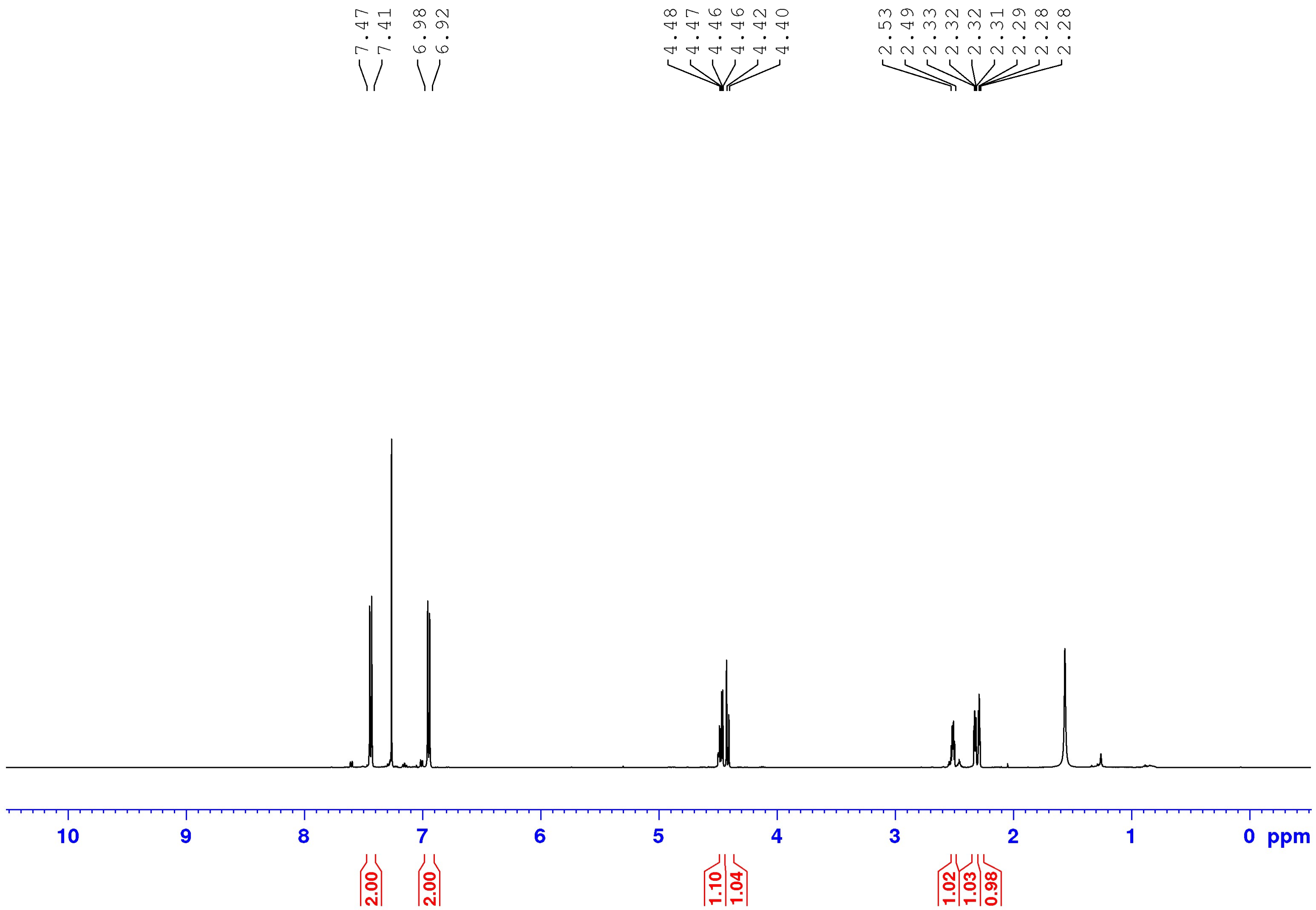

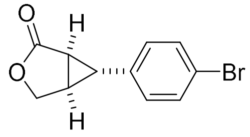

(–)-**SI-2a-Br**

Figure S91. ^{1}H NMR (500 MHz, $CDCl_3$) of **(–)-SI-2a-Br**

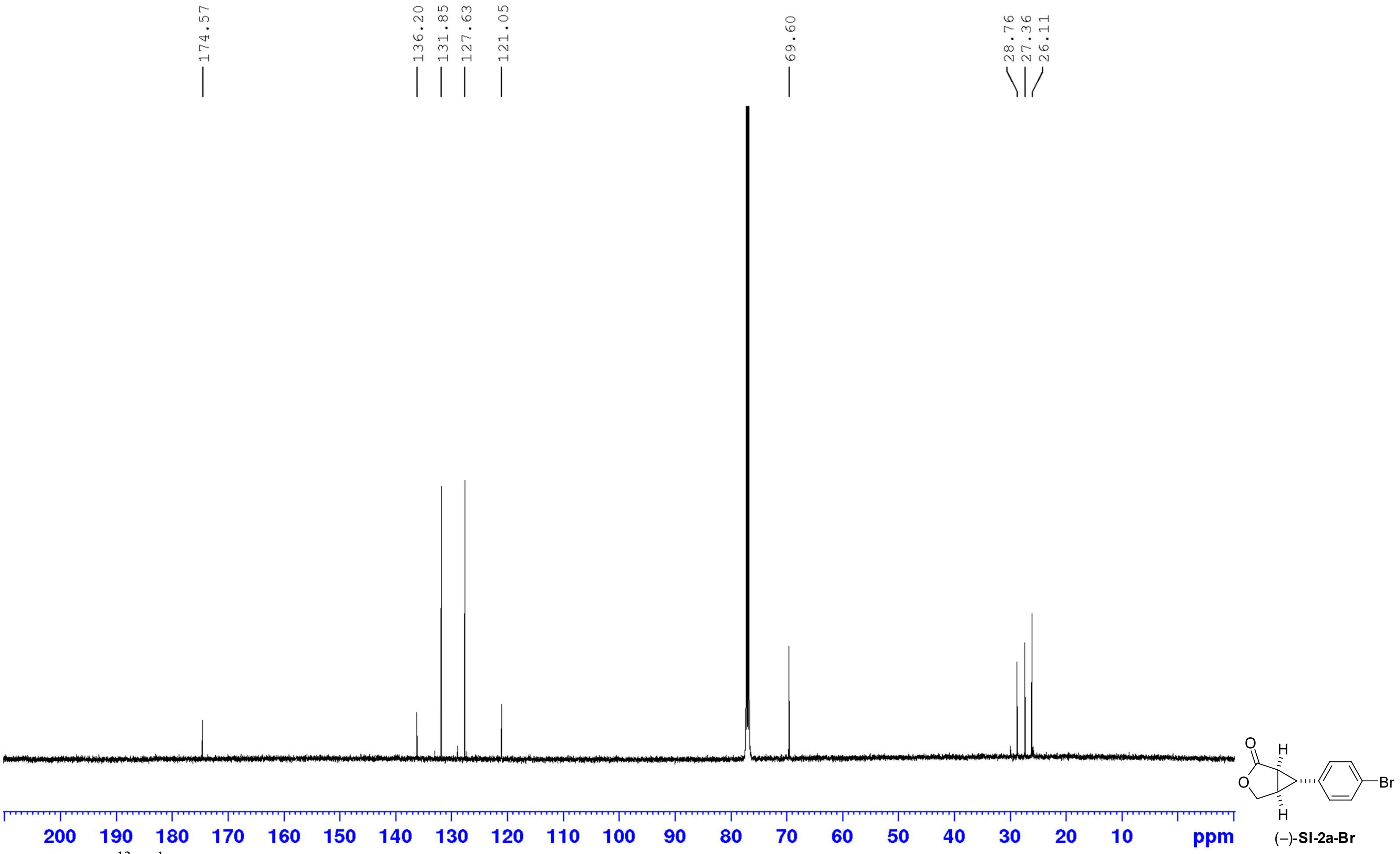

Figure S92. $^{13}C\{^{1}H\}$ NMR (126 MHz, $CDCl_3$) of **(–)-SI-2a-Br**

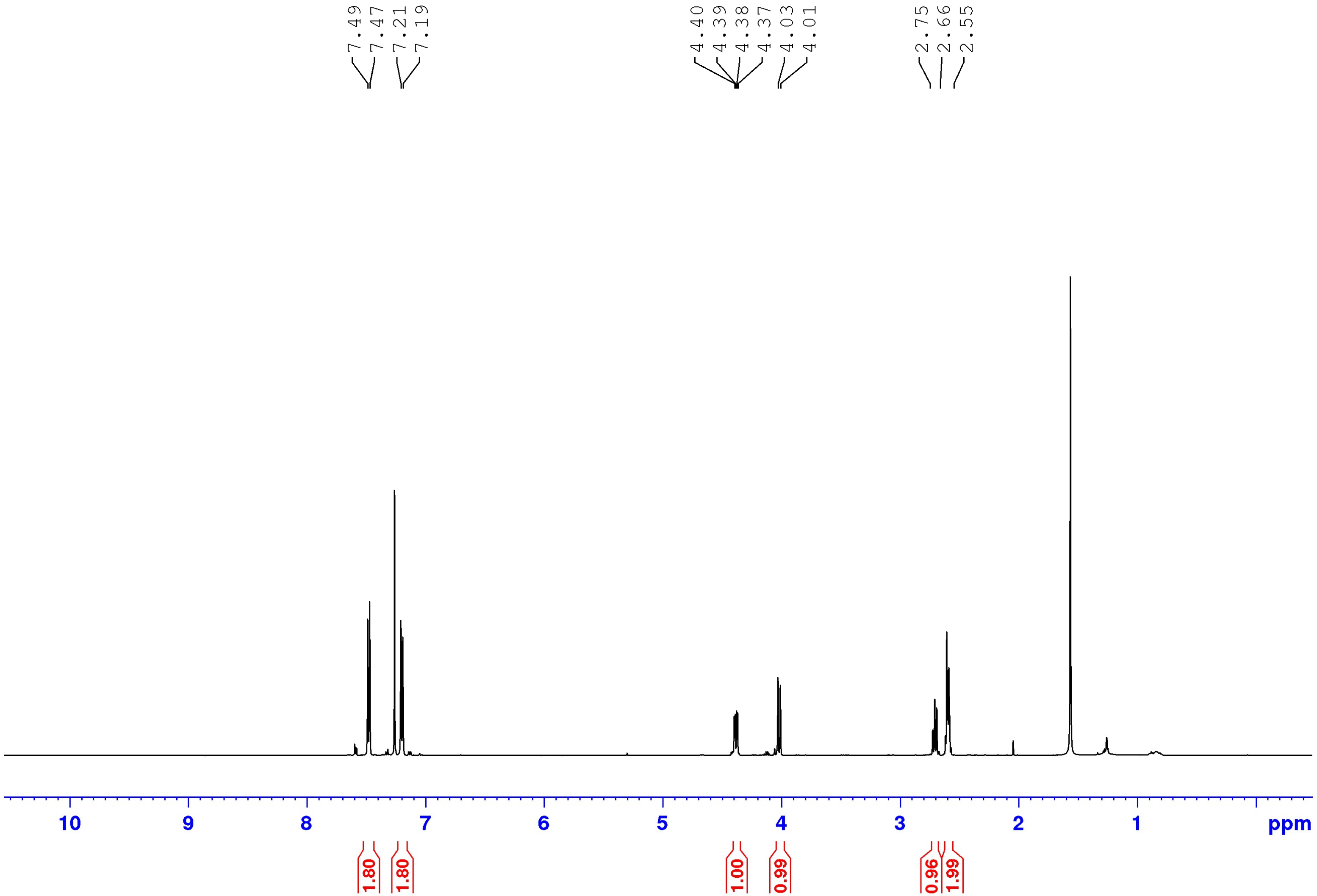

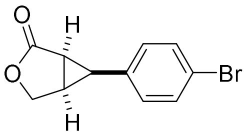

(+)-**SI-2f-Br**

Figure S93. ^{1}H NMR (500 MHz, $CDCl_3$) of **(+)-SI-2f-Br**

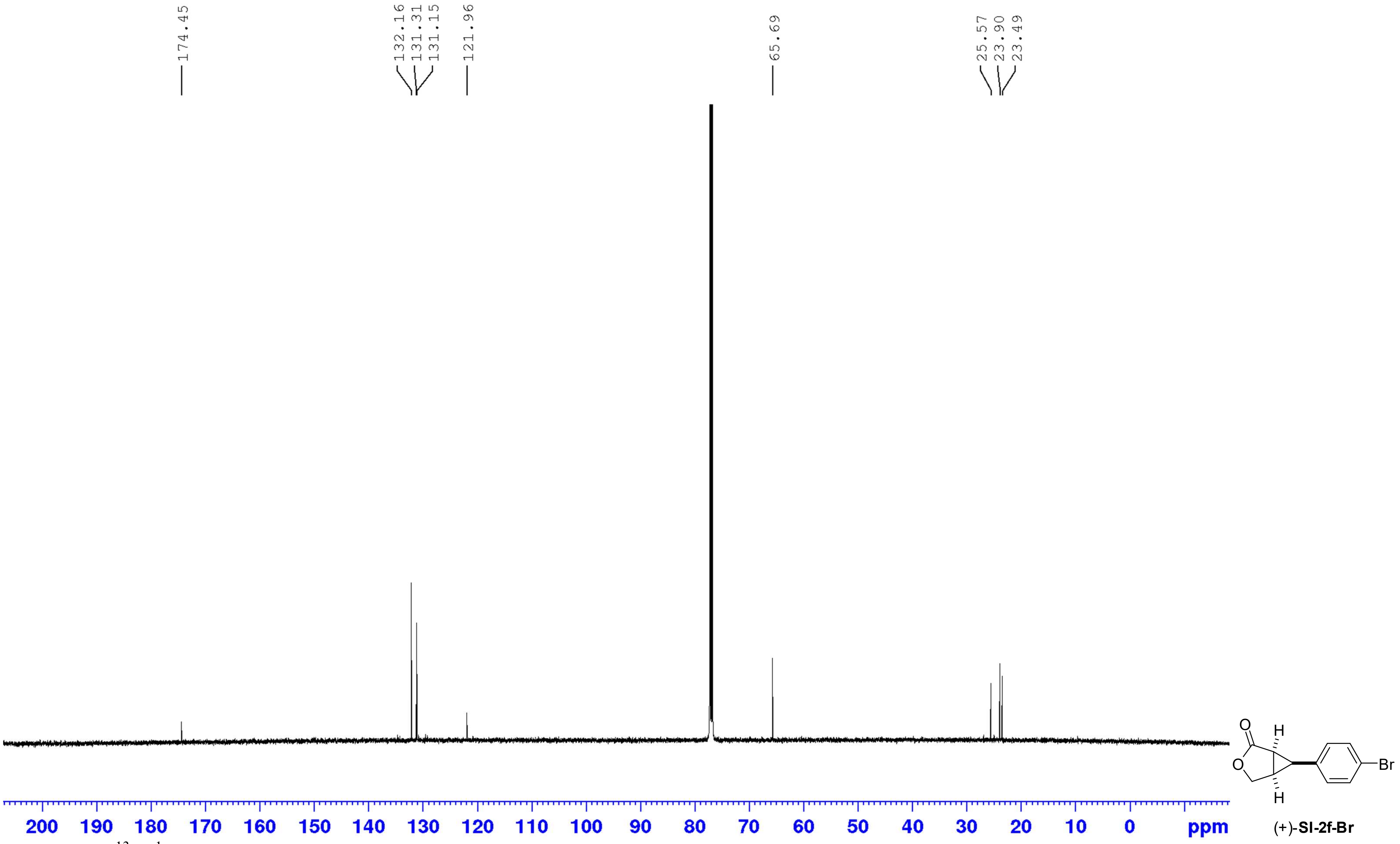

Figure S94. $^{13}C\{^{1}H\}$ NMR (126 MHz, $CDCl_3$) of **(+)-SI-2f-Br**

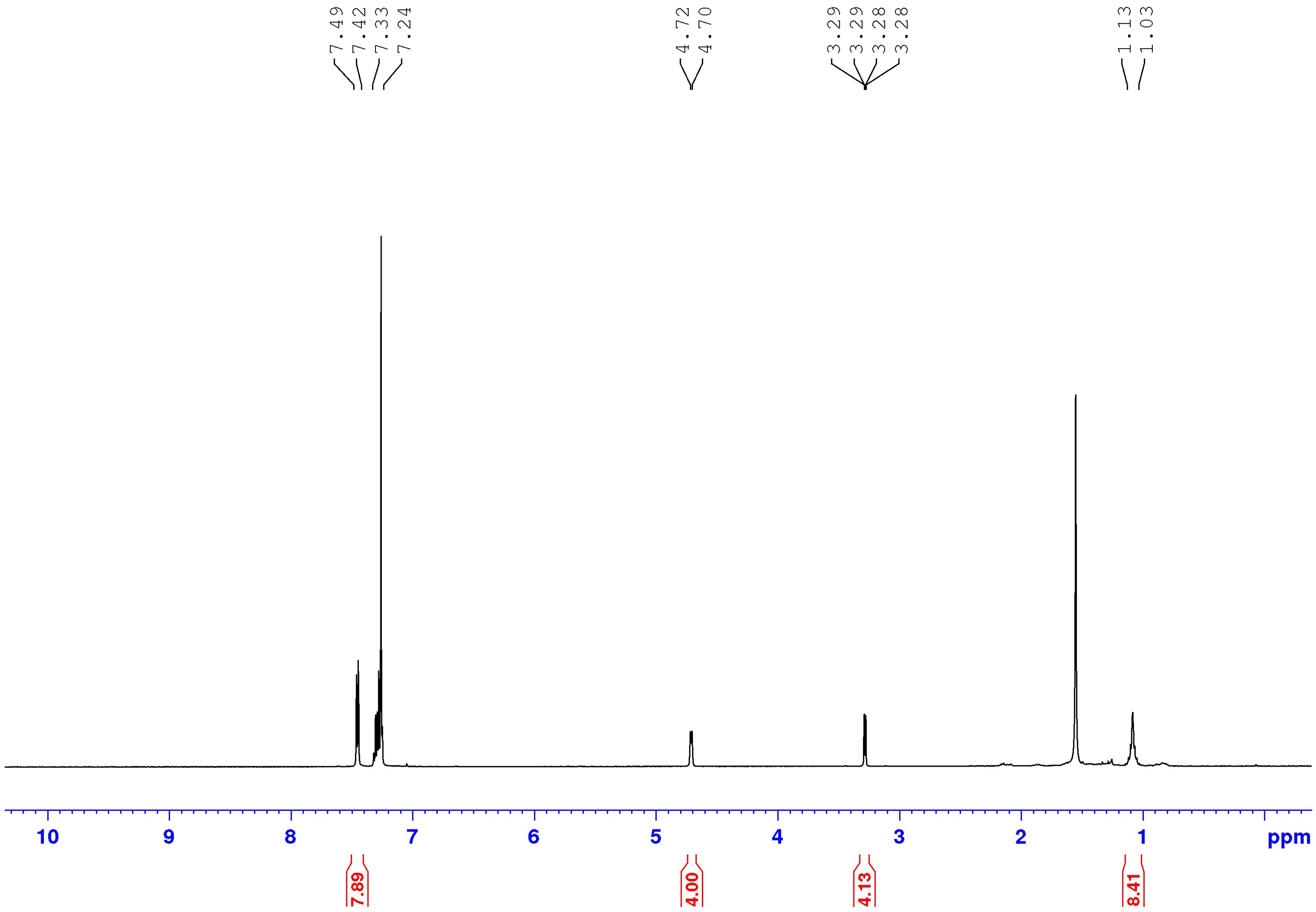
7.49
7.42
7.33
7.24
4.72
4.70
3.29
3.29
3.28
3.28
1.13
1.03
10
9
8
7
6
5
4
3
2
1
ppm
7.89
4.00
4.13
8.41

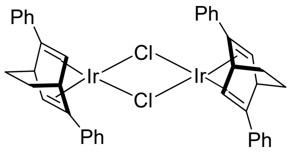

Figure S95. ^{1}H NMR (500 MHz, $CDCl_3$) of **[((*R*,*R*)-3)IrCl]$_2$**

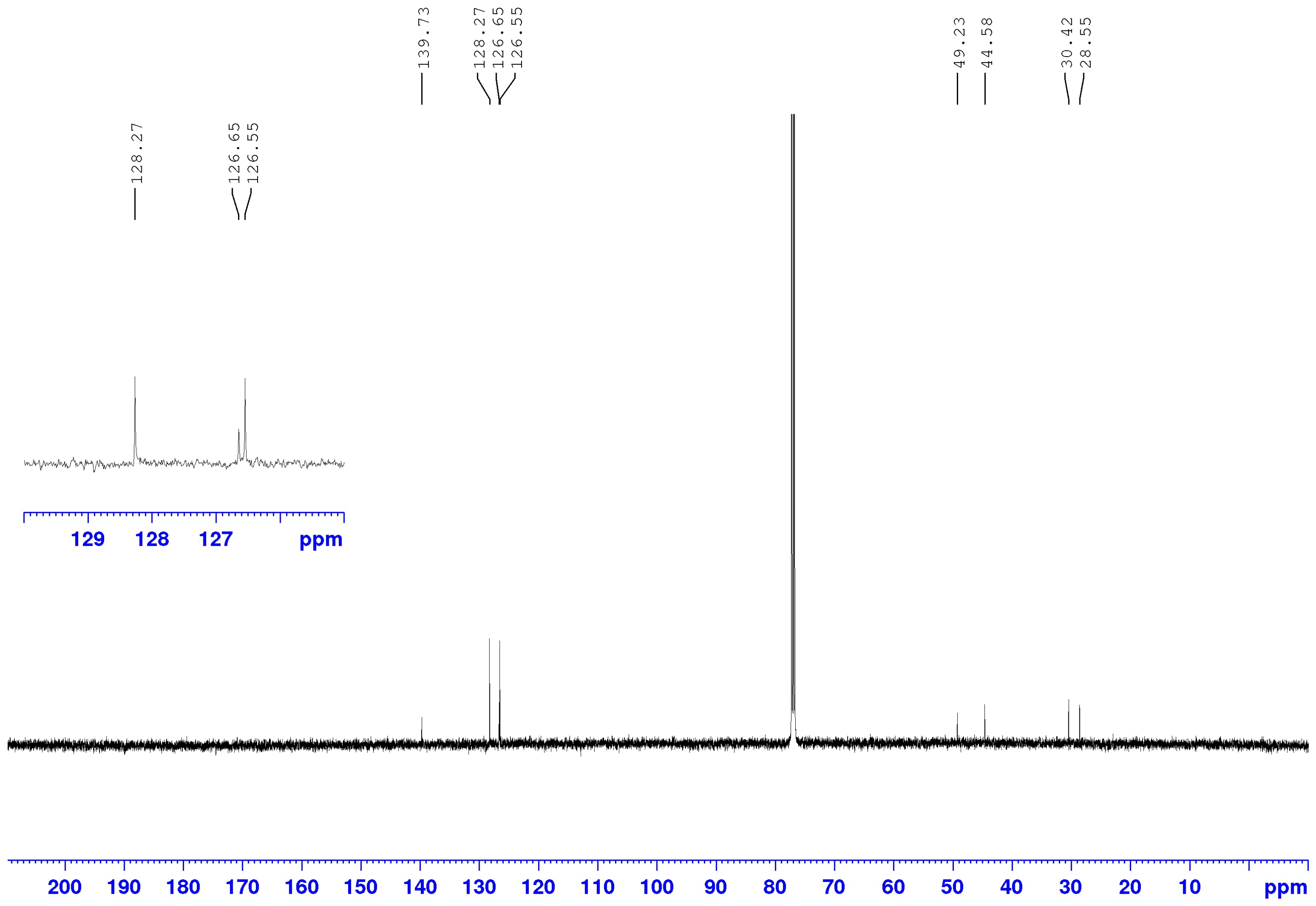
139.73
128.27
126.65
126.55
49.23
44.58
30.42
28.55
128.27
126.65
126.55
129
128
127
ppm
200
190
180
170
160
150
140
130
120
110
100
90
80
70
60
50
40
30
20
10
ppm

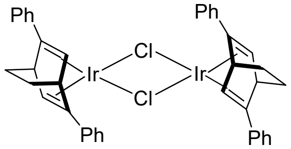

Figure S96. $^{13}C\{^{1}H\}$ NMR (126 MHz, $CDCl_3$) of **[((*R*,*R*)-3)IrCl]$_2$**

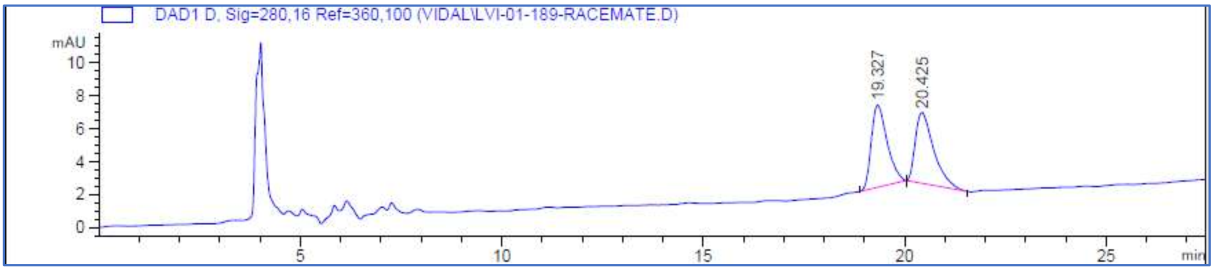

Signal 4: DAD1 D, Sig=280,16 Ref=360,100

Peak #	RetTime [min]	Type	Width [min]	Area [mAU*s]	Height [mAU]	Area %
1	19.327	BB	0.4136	136.43080	4.98006	49.9147
2	20.425	BB	0.4603	136.89690	4.28969	50.0853
Totals :				273.32770	9.26975	

Figure S97. Chiral HPLC trace of compound (±)-**S6b**

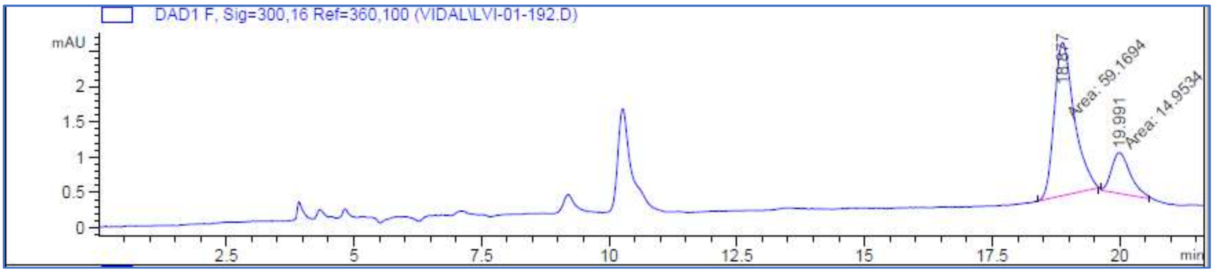

Signal 4: DAD1 F, Sig=300,16 Ref=360,100

Peak #	RetTime [min]	Type	Width [min]	Area [mAU*s]	Height [mAU]	Area %
1	18.877	MM	0.4543	59.16943	2.17060	79.8262
2	19.991	MM	0.4302	14.95335	5.79354e-1	20.1738
Totals :				74.12278	2.74996	

Figure S98. Chiral HPLC trace of compound (*S*,*S*)-**S6b** after first fermentation

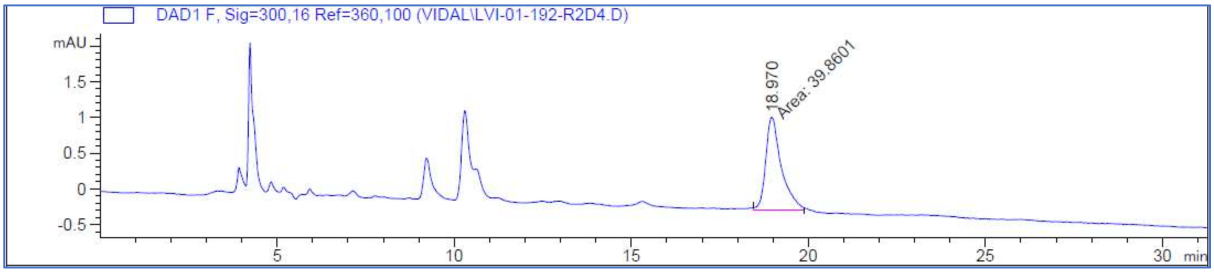

Signal 4: DAD1 F, Sig=300,16 Ref=360,100

Peak #	RetTime [min]	Type	Width [min]	Area [mAU*s]	Height [mAU]	Area %
1	18.970	MM	0.5112	39.86008	1.29963	100.0000
Totals :				39.86008	1.29963	

Figure S99. Chiral HPLC trace of compound (*S*,*S*)-**S6b** after second fermentation

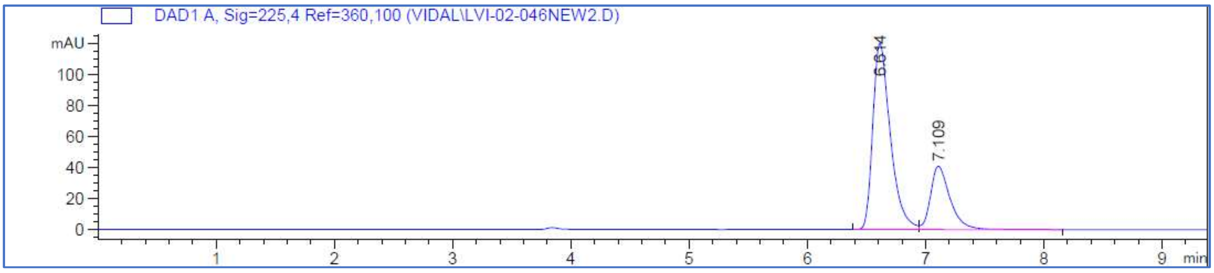

Signal 1: DAD1 A, Sig=225,4 Ref=360,100

Peak #	RetTime [min]	Type	Width [min]	Area [mAU*s]	Height [mAU]	Area %
1	6.614	BV	0.1568	1241.76550	119.96014	72.5695
2	7.109	VB	0.1720	469.37378	40.86735	27.4305
Totals :				1711.13928	160.82749	

Figure S101. Chiral HPLC trace of compound (±)-**S78**

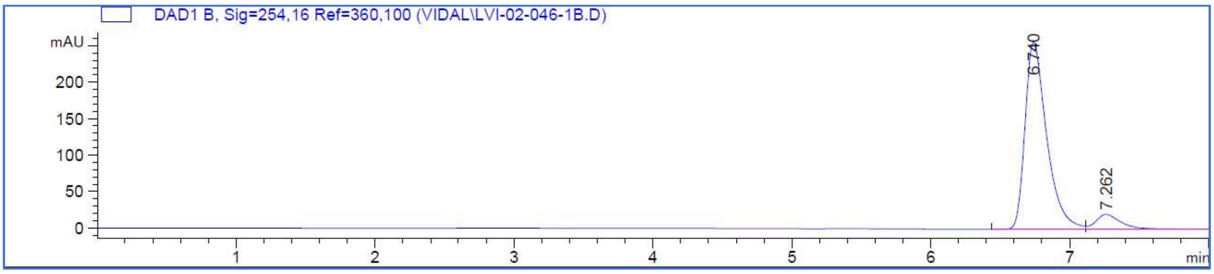

Signal 2: DAD1 B, Sig=254,16 Ref=360,100

Peak #	RetTime [min]	Type	Width [min]	Area [mAU*s]	Height [mAU]	Area %
1	6.740	BV	0.1620	2771.11572	256.57169	91.7123
2	7.262	VBA	0.1816	250.41550	20.34597	8.2877
Totals :				3021.53122	276.91766	

Figure S102. Chiral HPLC trace of compound (*R*,*R*)-**S78** after first recrystallization

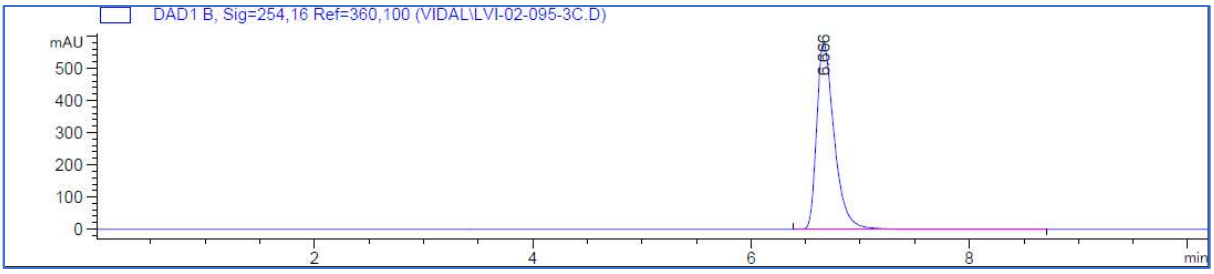

Signal 2: DAD1 B, Sig=254,16 Ref=360,100

Peak #	RetTime [min]	Type	Width [min]	Area [mAU*s]	Height [mAU]	Area %
1	6.666	BB	0.1677	6450.14404	580.03278	100.0000
Totals :				6450.14404	580.03278	

Figure S103. Chiral HPLC trace of compound (*R*,*R*)-**S78** after second recrystallization

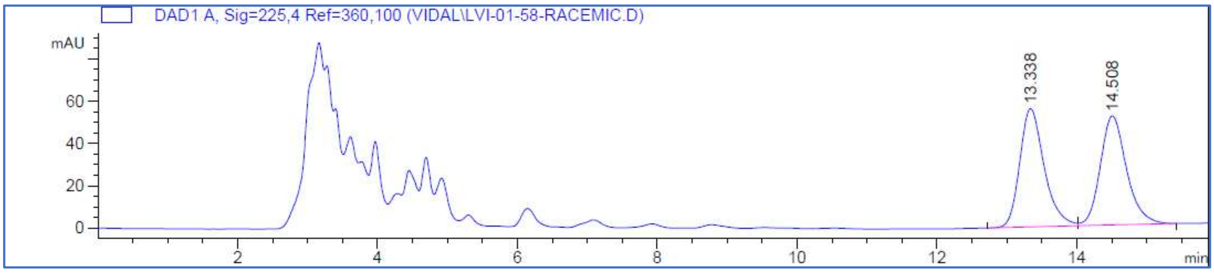

Signal 1: DAD1 A, Sig=225,4 Ref=360,100

Peak #	RetTime [min]	Type	Width [min]	Area [mAU*s]	Height [mAU]	Area %
1	13.338	BV	0.3692	1359.26831	56.00029	50.2005
2	14.508	VB	0.3968	1348.41003	51.61428	49.7995
Totals :				2707.67834	107.61457	

Figure S104. Chiral HPLC trace of compound (±)-**2a**

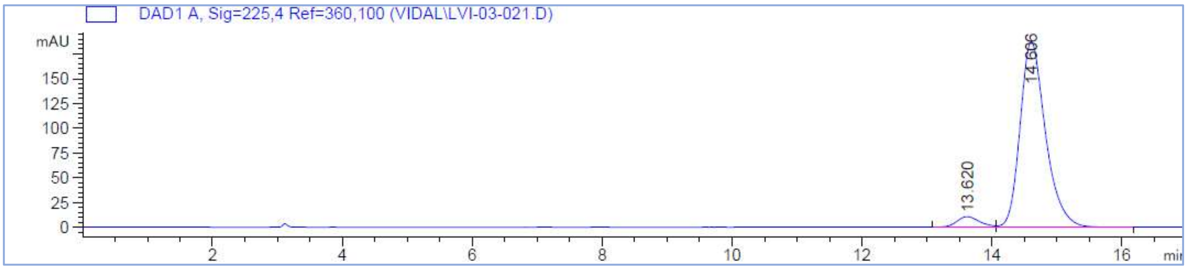

Signal 1: DAD1 A, Sig=225,4 Ref=360,100

Peak #	RetTime [min]	Type	Width [min]	Area [mAU*s]	Height [mAU]	Area %
1	13.620	BV	0.3720	256.26837	10.52576	4.8060
2	14.606	VB	0.4126	5076.02783	187.07167	95.1940
Totals :				5332.29620	197.59743	

Figure S105. Chiral HPLC trace of compound (–)-**2a** obtained with [Ir(coe)$_2$Cl]$_2$/(*R*,*R*)-**3**

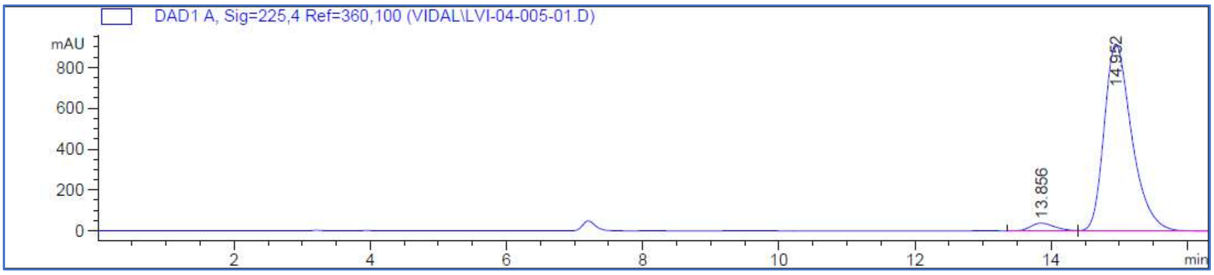

Signal 1: DAD1 A, Sig=225,4 Ref=360,100

Peak #	RetTime [min]	Type	Width [min]	Area [mAU*s]	Height [mAU]	Area %
1	13.856	VV	0.3834	972.78571	38.67639	3.6341
2	14.952	VBA	0.4290	2.57954e4	914.78687	96.3659
Totals :				2.67682e4	953.46325	

Figure S106. Chiral HPLC trace of compound (–)-**2a** obtained with [((*R*,*R*)-**3**)IrCl]$_2$

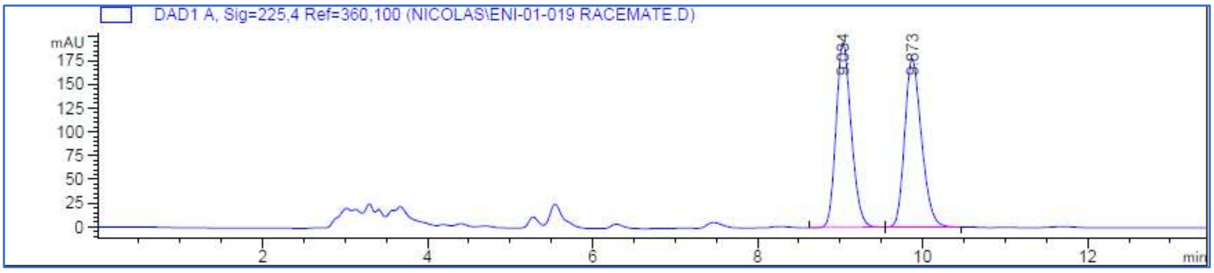

Peak #	RetTime [min]	Type	Width [min]	Area [mAU*s]	Height [mAU]	Area %
1	9.034	BB	0.1976	2494.97437	194.55182	49.8825
2	9.873	BB	0.2173	2506.72998	176.87372	50.1175
Totals :				5001.70435	371.42554	

Signal 1: DAD1 A, Sig=225,4 Ref=360,100

Figure S107. Chiral HPLC trace of compound (±)-**2b**

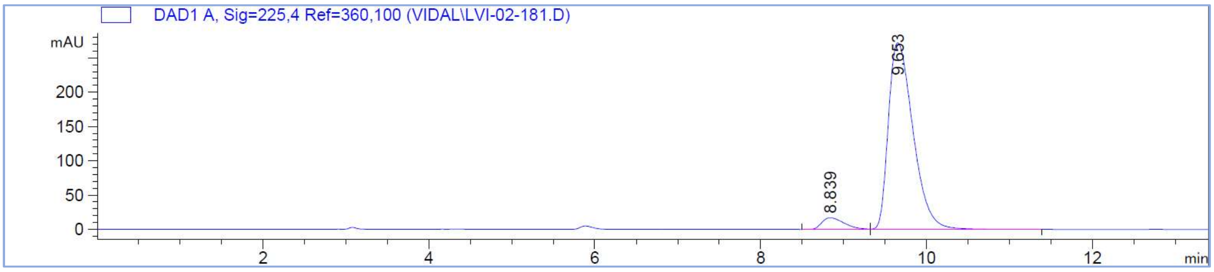

Signal 1: DAD1 A, Sig=225,4 Ref=360,100

Peak #	RetTime [min]	Type	Width [min]	Area [mAU*s]	Height [mAU]	Area %
1	8.839	BV	0.2968	322.48520	17.03998	5.4162
2	9.653	VB	0.3212	5631.64697	272.42474	94.5838
Totals :				5954.13217	289.46472	

Figure S108. Chiral HPLC trace of compound (–)-**2b**

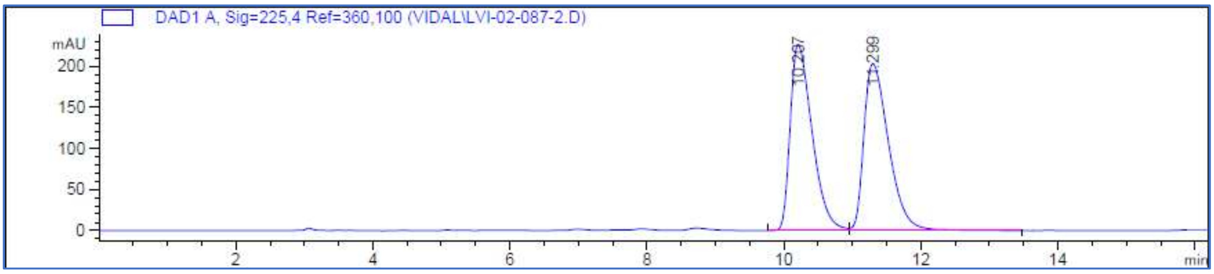

Signal 1: DAD1 A, Sig=225,4 Ref=360,100

Peak #	RetTime [min]	Type	Width [min]	Area [mAU*s]	Height [mAU]	Area %
1	10.207	BV	0.3424	5016.40723	226.52025	49.6933
2	11.299	VB	0.3855	5078.33691	203.24330	50.3067
Totals :				1.00947e4	429.76355	

Figure S109. Chiral HPLC trace of compound (±)-**2c**

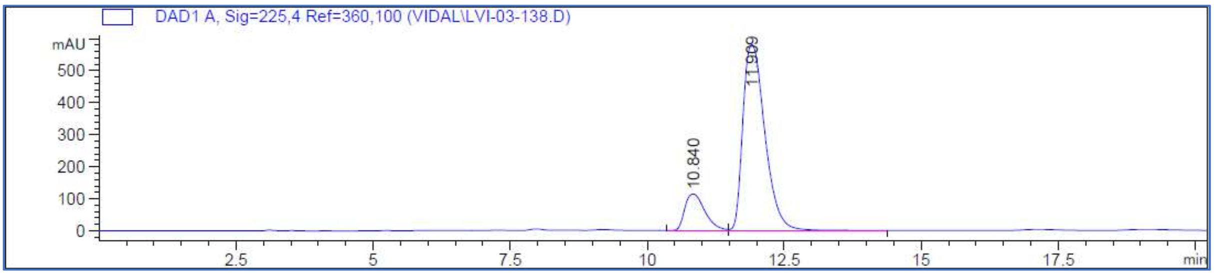

Signal 1: DAD1 A, Sig=225,4 Ref=360,100

Peak #	RetTime [min]	Type	Width [min]	Area [mAU*s]	Height [mAU]	Area %
1	10.840	BV	0.3942	2872.57178	113.15848	15.0798
2	11.909	VB	0.4312	1.61766e4	580.38623	84.9202
Totals :				1.90492e4	693.54471	

Figure S110. Chiral HPLC trace of compound (–)-**2c**

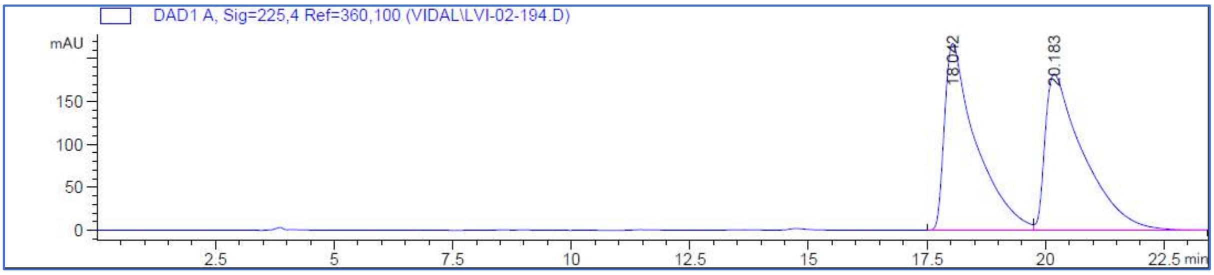

Signal 1: DAD1 A, Sig=225,4 Ref=360,100

Peak #	RetTime [min]	Type	Width [min]	Area [mAU*s]	Height [mAU]	Area %
1	18.042	BV	0.6427	1.00773e4	217.41820	49.6230
2	20.183	VBA	0.7778	1.02304e4	182.00763	50.3770
Totals :				2.03076e4	399.42583	

Figure S111. Chiral HPLC trace of compound (±)-**2d**

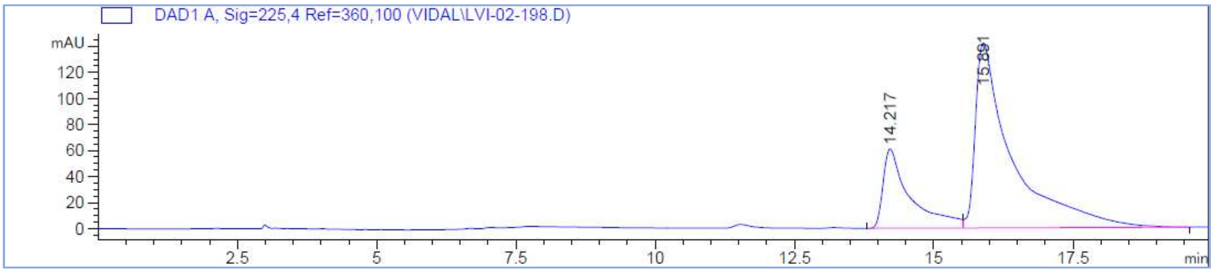

Signal 1: DAD1 A, Sig=225,4 Ref=360,100

Peak #	RetTime [min]	Type	Width [min]	Area [mAU*s]	Height [mAU]	Area %
1	14.217	BV	0.4647	2072.44824	60.94343	24.0576
2	15.891	VB	0.6232	6542.08545	142.06633	75.9424
Totals :				8614.53369	203.00976	

Figure S112. Chiral HPLC trace of compound (–)-**2d**

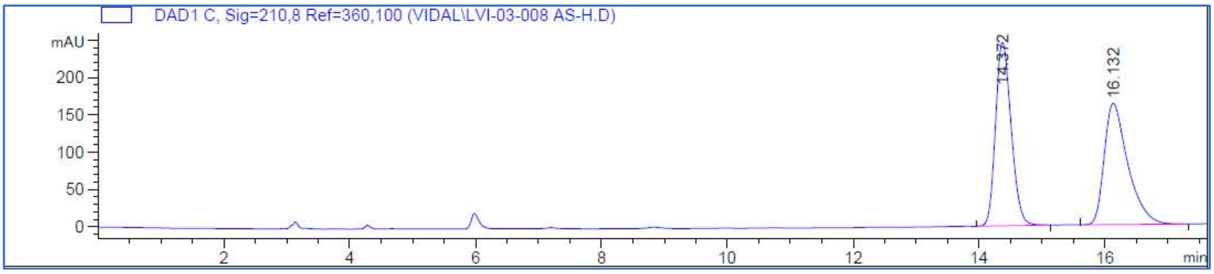

Signal 3: DAD1 C, Sig=210,8 Ref=360,100

Peak #	RetTime [min]	Type	Width [min]	Area [mAU*s]	Height [mAU]	Area %
1	14.372	BB	0.2668	4224.91553	245.43188	49.9544
2	16.132	BB	0.3902	4232.62988	162.31223	50.0456
Totals :				8457.54541	407.74411	

Figure S113. Chiral HPLC trace of compound (±)-**2e**

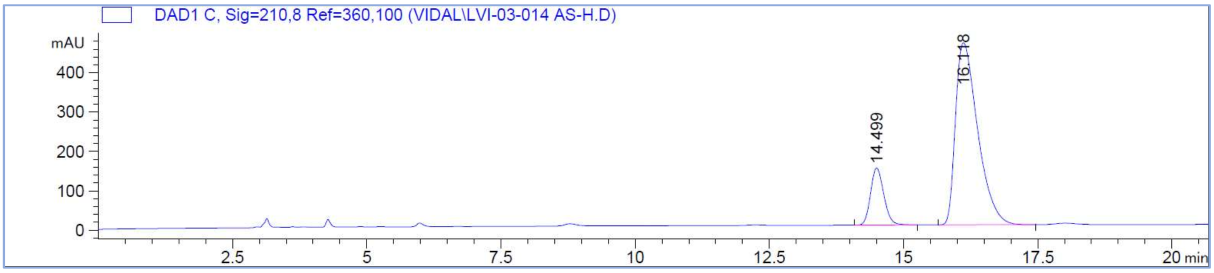

Signal 3: DAD1 C, Sig=210,8 Ref=360,100

Peak #	RetTime [min]	Type	Width [min]	Area [mAU*s]	Height [mAU]	Area %
1	14.499	BB	0.2659	2484.07690	144.94411	15.7475
2	16.118	BB	0.4434	1.32904e4	462.28415	84.2525
Totals :				1.57745e4	607.22826	

Figure S114. Chiral HPLC trace of compound (–)-**2e**

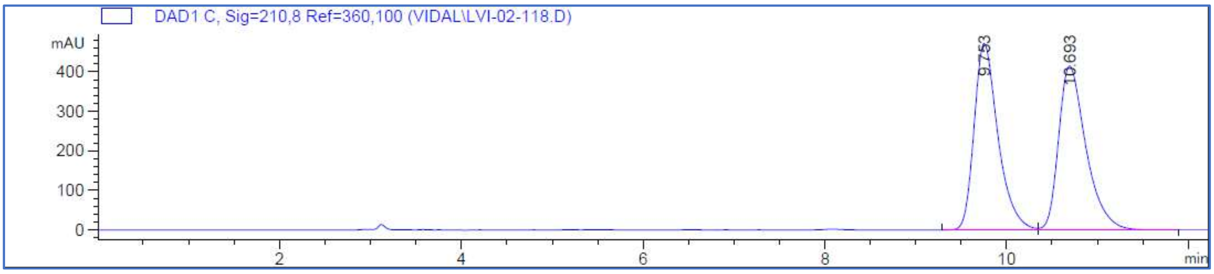

Signal 3: DAD1 C, Sig=210,8 Ref=360,100

Peak #	RetTime [min]	Type	Width [min]	Area [mAU*s]	Height [mAU]	Area %
1	9.753	BV	0.2754	8609.62695	470.71152	49.9149
2	10.693	VB	0.3155	8638.98242	413.83514	50.0851
Totals :				1.72486e4	884.54666	

Figure S115. Chiral HPLC trace of compound (±)-**2f**

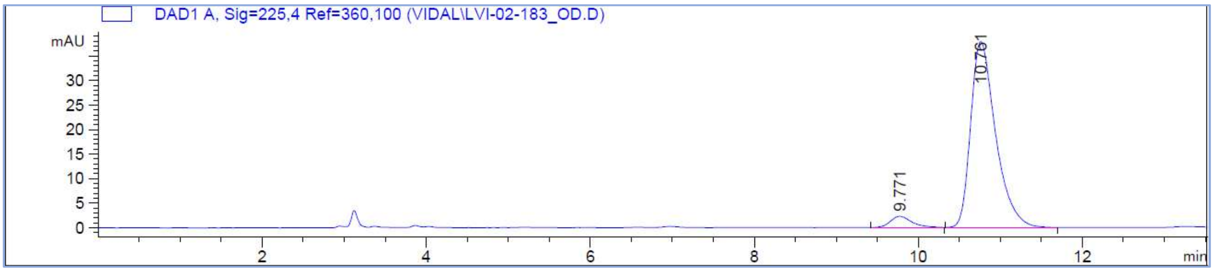

Signal 1: DAD1 A, Sig=225,4 Ref=360,100

Peak #	RetTime [min]	Type	Width [min]	Area [mAU*s]	Height [mAU]	Area %
1	9.771	BB	0.2679	40.31196	2.26266	4.8070
2	10.761	BB	0.3179	798.29480	37.87061	95.1930
Totals :				838.60676	40.13327	

Figure S116. Chiral HPLC trace of compound (+)-**2f**

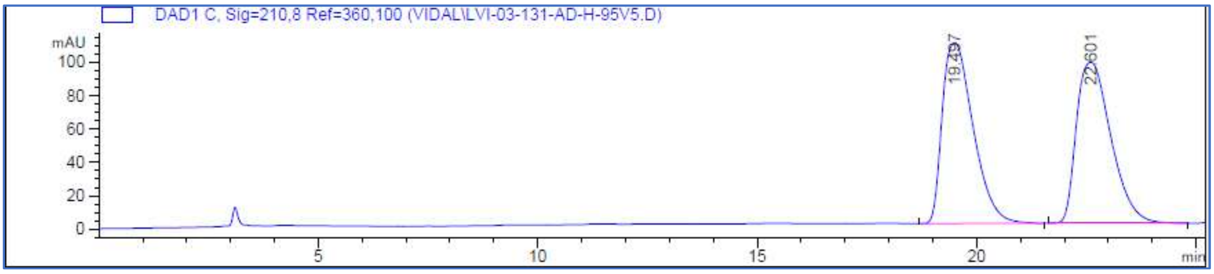

Signal 3: DAD1 C, Sig=210,8 Ref=360,100

Peak #	RetTime [min]	Type	Width [min]	Area [mAU*s]	Height [mAU]	Area %
1	19.497	BB	0.7563	5287.21191	108.16035	50.1273
2	22.601	BB	0.8477	5260.35547	96.23649	49.8727
Totals :				1.05476e4	204.39684	

Figure S117. Chiral HPLC trace of compound (±)-**2g**

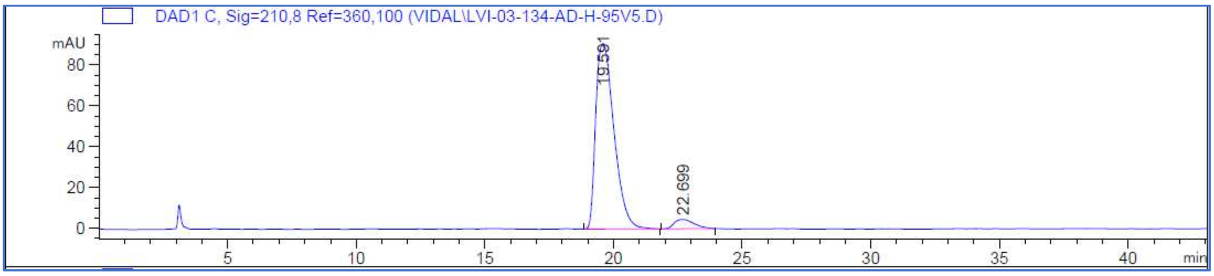

Signal 2: DAD1 B, Sig=254,16 Ref=360,100

Signal 3: DAD1 C, Sig=210,8 Ref=360,100

Peak #	RetTime [min]	Type	Width [min]	Area [mAU*s]	Height [mAU]	Area %
1	19.591	BB	0.7565	4473.06299	90.84206	94.8466
2	22.699	BB	0.6466	243.04028	4.55448	5.1534
Totals :				4716.10327	95.39654	

Figure S118. Chiral HPLC trace of compound (+)-**2g**

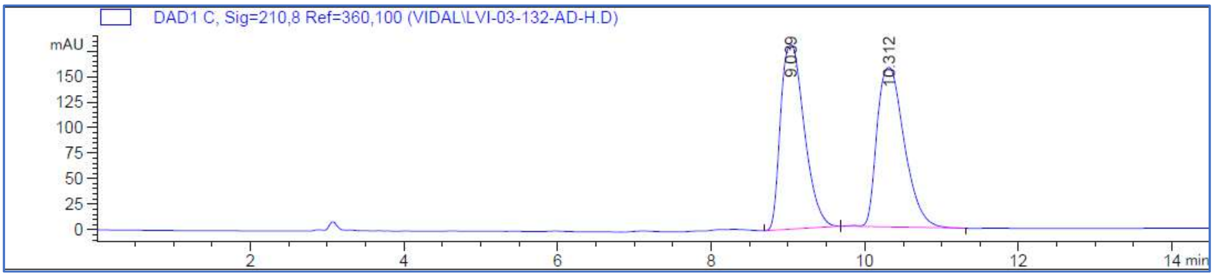

Signal 3: DAD1 C, Sig=210,8 Ref=360,100

Peak #	RetTime [min]	Type	Width [min]	Area [mAU*s]	Height [mAU]	Area %
1	9.039	BB	0.3362	3876.56201	180.73363	50.0790
2	10.312	BB	0.3859	3864.33276	156.57106	49.9210
Totals :				7740.89478	337.30469	

Figure S119. Chiral HPLC trace of compound (±)-**2h**

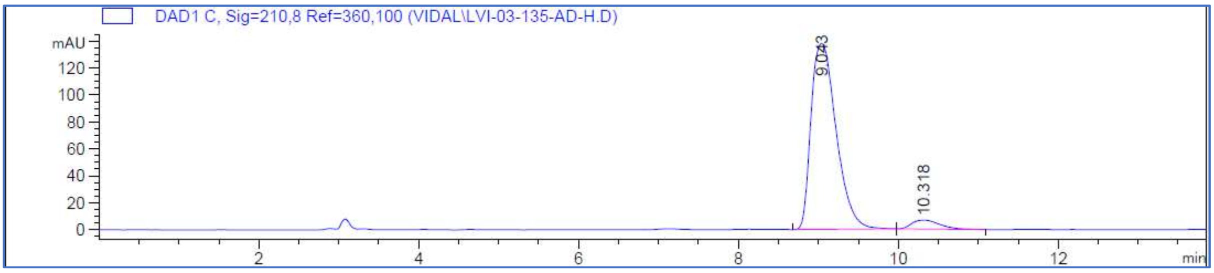

Signal 3: DAD1 C, Sig=210,8 Ref=360,100

Peak #	RetTime [min]	Type	Width [min]	Area [mAU*s]	Height [mAU]	Area %
1	9.043	BB	0.3408	3013.06812	137.93880	95.0510
2	10.318	BB	0.3735	156.87943	6.54931	4.9490
Totals :				3169.94754	144.48811	

Figure S120. Chiral HPLC trace of compound (+)-**2h**

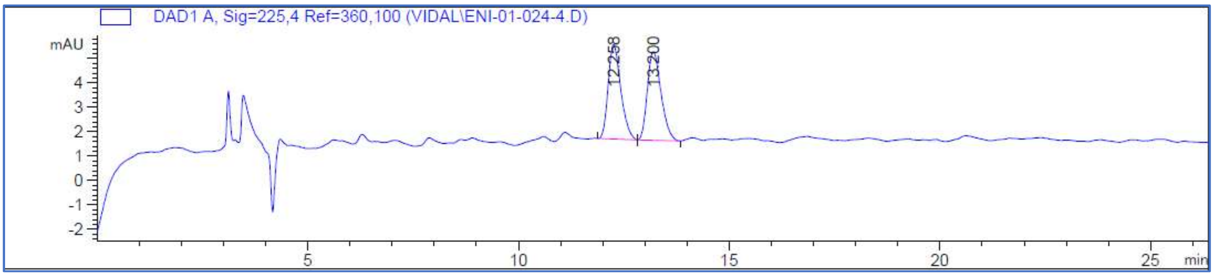

Signal 1: DAD1 A, Sig=225,4 Ref=360,100

Peak #	RetTime [min]	Type	Width [min]	Area [mAU*s]	Height [mAU]	Area %
1	12.258	BB	0.3113	80.73673	3.87186	49.5870
2	13.200	BB	0.3392	82.08173	3.63852	50.4130
Totals :				162.81846	7.51038	

Figure S121. Chiral HPLC trace of compound (±)-**2i**

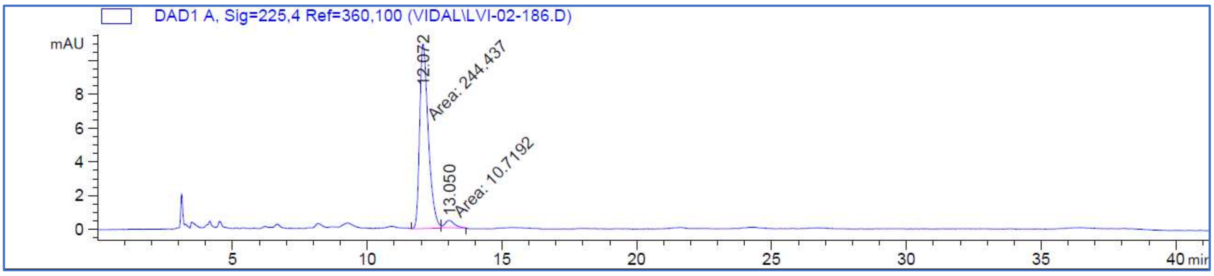

Signal 1: DAD1 A, Sig=225,4 Ref=360,100

Peak #	RetTime [min]	Type	Width [min]	Area [mAU*s]	Height [mAU]	Area %
1	12.072	MM	0.3718	244.43683	10.95871	95.7990
2	13.050	MM	0.4092	10.71917	4.36556e-1	4.2010
Totals :				255.15600	11.39527	

Figure S122. Chiral HPLC trace of compound (–)-**2i**

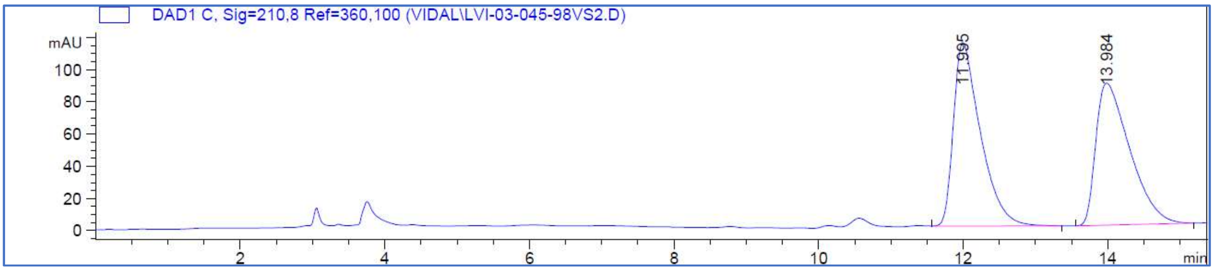

Signal 3: DAD1 C, Sig=210,8 Ref=360,100

Peak #	RetTime [min]	Type	Width [min]	Area [mAU*s]	Height [mAU]	Area %
1	11.995	BB	0.3783	2950.27173	113.85667	50.6970
2	13.984	BB	0.4961	2869.15259	88.07438	49.3030
Totals :				5819.42432	201.93105	

Figure S123. Chiral HPLC trace of compound (±)-**2j**

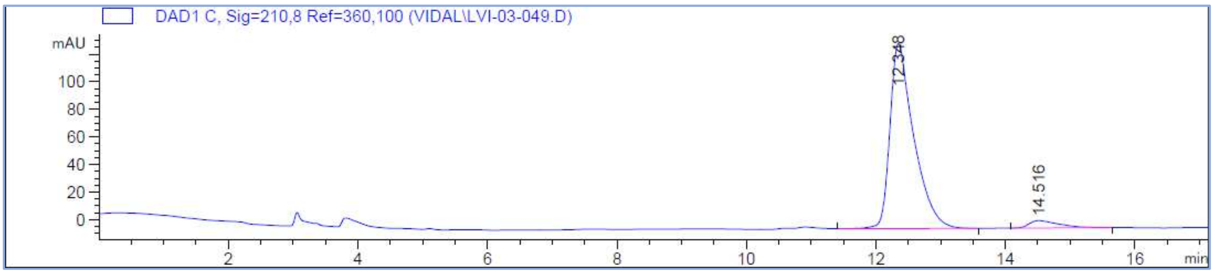

Signal 3: DAD1 C, Sig=210,8 Ref=360,100

Peak #	RetTime [min]	Type	Width [min]	Area [mAU*s]	Height [mAU]	Area %
1	12.348	BB	0.3658	3423.23071	134.10480	95.2189
2	14.516	BB	0.4451	171.88721	5.40338	4.7811
Totals :				3595.11792	139.50818	

Figure S124. Chiral HPLC trace of compound (–)-**2j**

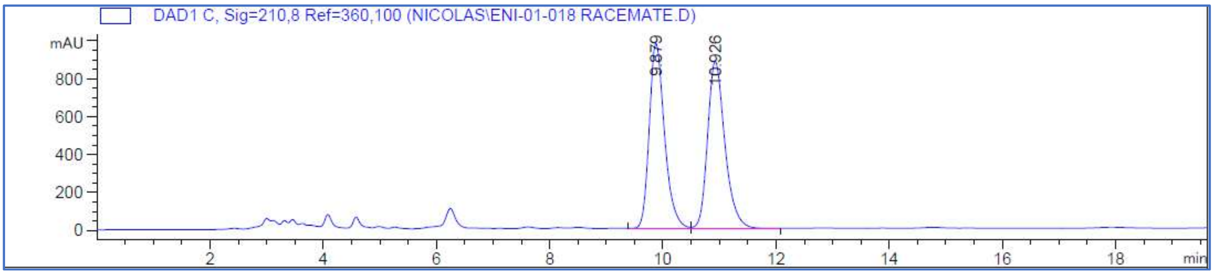

Signal 3: DAD1 C, Sig=210,8 Ref=360,100

Peak #	RetTime [min]	Type	Width [min]	Area [mAU*s]	Height [mAU]	Area %
1	9.879	BV	0.2869	1.82848e4	974.58423	49.4416
2	10.926	VB	0.3239	1.86979e4	880.03857	50.5584
Totals :				3.69827e4	1854.62280	

Figure S125. Chiral HPLC trace of compound (±)-**2k**

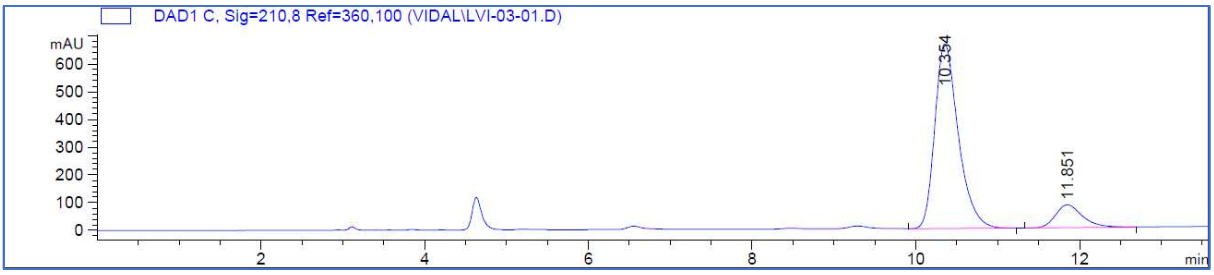

Signal 3: DAD1 C, Sig=210,8 Ref=360,100

Peak #	RetTime [min]	Type	Width [min]	Area [mAU*s]	Height [mAU]	Area %
1	10.354	BB	0.3028	1.32823e4	665.74725	87.5630
2	11.851	BB	0.3469	1886.53674	82.47292	12.4370
Totals :				1.51688e4	748.22018	

Figure S126. Chiral HPLC trace of compound (–)-**2k**

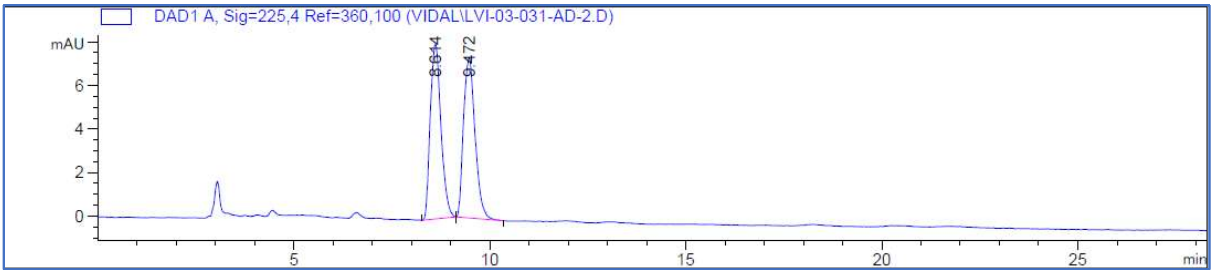

Signal 1: DAD1 A, Sig=225,4 Ref=360,100

Peak #	RetTime [min]	Type	Width [min]	Area [mAU*s]	Height [mAU]	Area %
1	8.614	BB	0.3009	151.67168	8.01078	50.2090
2	9.472	BB	0.3247	150.40903	7.35058	49.7910
Totals :				302.08070	15.36136	

Figure S127. Chiral HPLC trace of compound (±)-**2l**

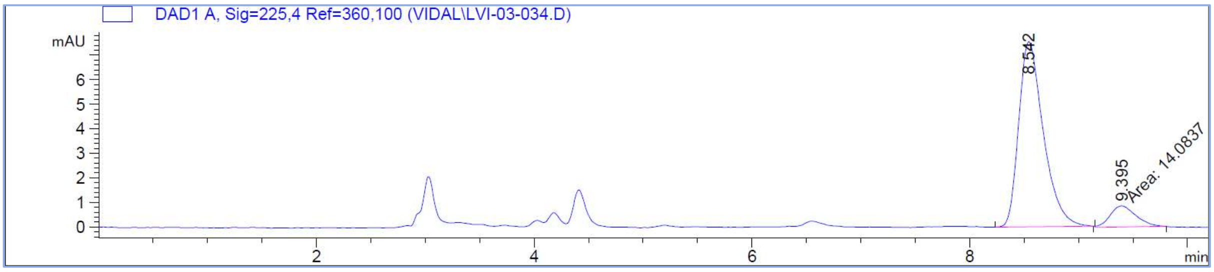

Signal 1: DAD1 A, Sig=225,4 Ref=360,100

Peak #	RetTime [min]	Type	Width [min]	Area [mAU*s]	Height [mAU]	Area %
1	8.542	BB	0.2373	118.55526	7.54773	89.3820
2	9.395	MM	0.2733	14.08366	8.58882e-1	10.6180
Totals :				132.63892	8.40661	

Figure S128. Chiral HPLC trace of compound (+)-**2l**

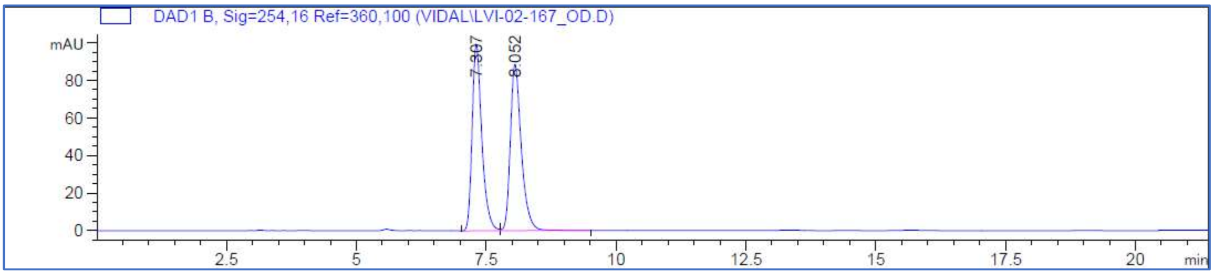

Signal 2: DAD1 B, Sig=254,16 Ref=360,100

Peak #	RetTime [min]	Type	Width [min]	Area [mAU*s]	Height [mAU]	Area %
1	7.307	BV	0.1951	1286.77002	99.38347	49.7286
2	8.052	VB	0.2214	1300.81702	88.53204	50.2714
Totals :				2587.58704	187.91551	

Figure S129. Chiral HPLC trace of compound (±)-**2m**

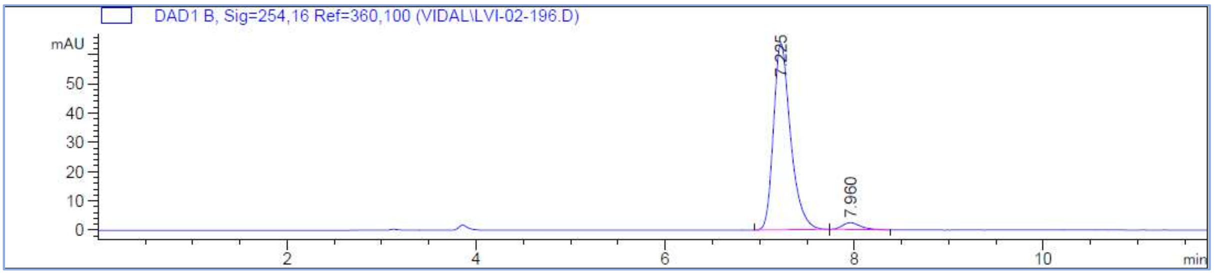

Signal 2: DAD1 B, Sig=254,16 Ref=360,100

Peak #	RetTime [min]	Type	Width [min]	Area [mAU*s]	Height [mAU]	Area %
1	7.225	BB	0.1905	799.47339	63.64623	96.3039
2	7.960	BB	0.2017	30.68304	2.32860	3.6961
Totals :				830.15643	65.97483	

Figure S130. Chiral HPLC trace of compound (–)-**2m**

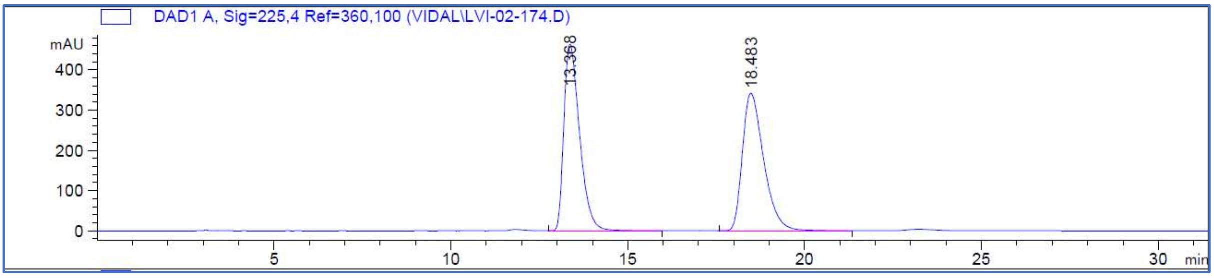

Signal 1: DAD1 A, Sig=225,4 Ref=360,100

Peak #	RetTime [min]	Type	Width [min]	Area [mAU*s]	Height [mAU]	Area %
1	13.368	BB	0.4770	1.43263e4	463.05487	50.0038
2	18.483	BB	0.6409	1.43241e4	341.80426	49.9962
Totals :				2.86504e4	804.85913	

Figure S131. Chiral HPLC trace of compound (±)-**2n**

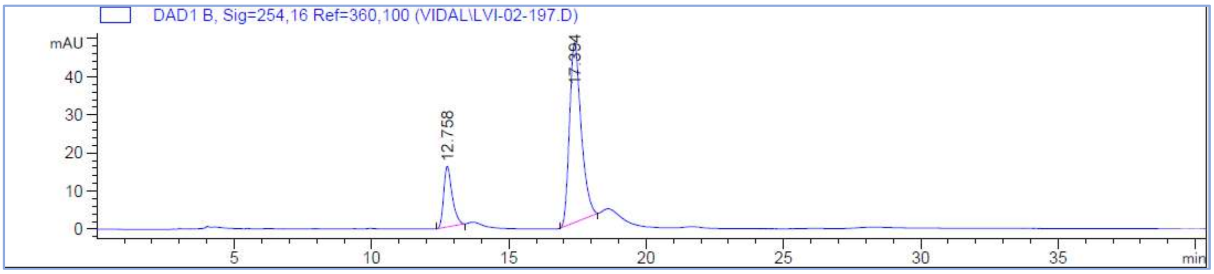

Signal 2: DAD1 B, Sig=254,16 Ref=360,100

Peak #	RetTime [min]	Type	Width [min]	Area [mAU*s]	Height [mAU]	Area %
1	12.758	BB	0.3221	340.72238	15.89664	19.6563
2	17.394	BB	0.4470	1392.67932	47.10191	80.3437
Totals :				1733.40170	62.99855	

Figure S132. Chiral HPLC trace of compound (–)-**2n**

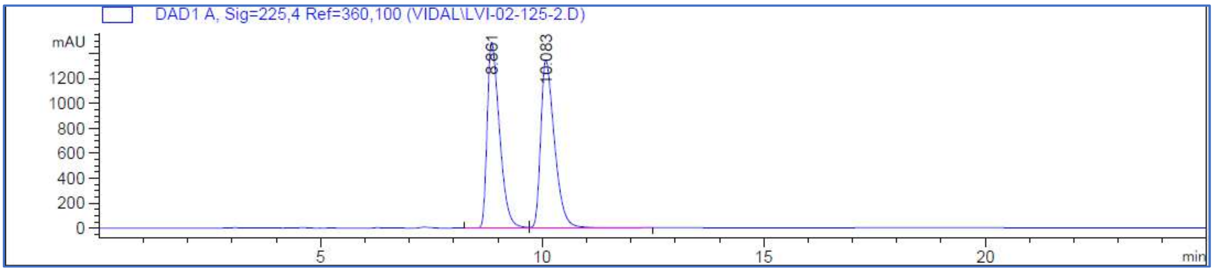

Signal 1: DAD1 A, Sig=225,4 Ref=360,100

Peak #	RetTime [min]	Type	Width [min]	Area [mAU*s]	Height [mAU]	Area %
1	8.861	BV	0.2970	2.87043e4	1488.54077	49.7136
2	10.083	VB	0.3297	2.90351e4	1345.99048	50.2864
Totals :				5.77395e4	2834.53125	

Figure S133. Chiral HPLC trace of compound (±)-**2o**

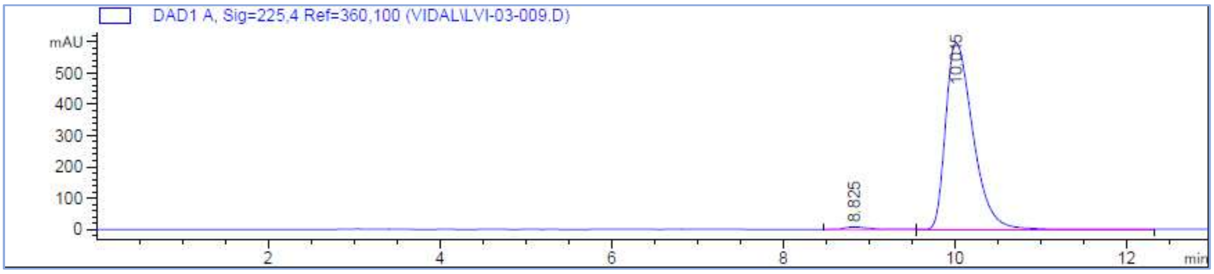

Signal 1: DAD1 A, Sig=225,4 Ref=360,100

Peak #	RetTime [min]	Type	Width [min]	Area [mAU*s]	Height [mAU]	Area %
1	8.825	BB	0.3053	142.82217	7.26591	1.0736
2	10.015	BB	0.3414	1.31605e4	596.55731	98.9264
Totals :				1.33033e4	603.82323	

Figure S134. Chiral HPLC trace of compound (–)-**2o**

References

[1] Spoehrle, S. S.; West, T. H.; Taylor, J. E.; Slawin, A. M.; Smith, A. D. Tandem Palladium and Isothiourea Relay Catalysis: Enantioselective Synthesis of α-Amino Acid Derivatives via Allylic Amination and [2, 3]-Sigmatropic Rearrangement. *J. Am. Chem. Soc.* **2017**, *139*, 11895-11902.

[2] West, T. H.; Daniels, D. S.; Slawin, A. M.; Smith, A. D. An isothiourea-catalyzed asymmetric [2, 3]-rearrangement of allylic ammonium ylides. *J. Am. Chem. Soc.* **2014**, *136*, 4476-4479.

[3] Hu, D. X.; Shibuya, G. M.; Burns, N. Z. Catalytic enantioselective dibromination of allylic alcohols. *J. Am. Chem. Soc.* **2013**, *135*, 12960-12963

[4] Davies, S. G.; Fletcher, A. M.; Roberts, P. M.; Thomson, J. E.; Zammit, C. M. Asymmetric syntheses of enantiopure C(5)-substituted transpentacins via diastereoselective Ireland–Claisen rearrangements. *Chem. Commun.* **2013**, *49*, 7037–7039.

[5] Chen, C.; Huang, Y.; Zhang, Z.; Dong, X. Q.; Zhang, X. Cobalt-catalyzed (Z)-selective semihydrogenation of alkynes with molecular hydrogen. *Chem. Commun.* **2017**, *53*, 4612-4615.

[6] Lu, Z.; Hu, X. D.; Zhang, H.; Zhang, X. W.; Cai, J.; Usman, M.; Kong, H.; Liu, W. B. Enantioselective assembly of cycloenones with a nitrile-containing all-carbon quaternary center from malononitriles enabled by Ni catalysis. *J. AM. Chem. Soc.* **2020**, *142*, 7328-7333

[7] Zhang, Y. Q.; Funken, N.; Winterscheid, P.; Gansäuer, A. Hydroxy-Directed, Fluoride-Catalyzed Epoxide Hydrosilylation for the Synthesis of 1, 4-Diols. *Angew. Chem. Int. Ed.* **2015**, *54*, 6931-6934.

[8] Prepared according to: McCourt, R. O.; Dénès, F.; Sanchez-Sanz, G.; & Scanlan, E. M. Rapid Access to Thiolactone Derivatives through Radical-Mediated Acyl Thiol–Ene and Acyl Thiol–Yne Cyclization. *Org. Lett.* **2018**, *20*, 2948-2951.

[9] RaghavaáSharma, G. V. Synthesis of chiral bicyclo[2.2.2]oct-5-en-2-ones via an intramolecular alkylation reaction. *J. Chem. Soc.*, *Perkin Trans. 1.* **1996**, 1305-1311.

[10] Ruggles, E. L.; Maleczka, R. E. Bleach/acetic acid-promoted chlorinative ring expansion of [2.2.1]- and [2.2.2]-bicycles. *Org. Lett.* **2002**, *4*, 3899–3902

[11] Gendrineau, T.; Chuzel, O.; Eijsberg, H.; Genet, J. P.; Darses, S. C1-Symmetric Monosubstituted Chiral Diene Ligands in Asymmetric Rhodium-Catalyzed 1, 4-Addition Reactions. *Angew. Chem. Int. Ed.* **2008**, *47*, 7669 –7672.

[12] Wallentin, C. J.; Orentas, E.; Butkus, E.; Wärnmark, K. Baker's yeast for sweet dough enables large-scale synthesis of enantiomerically pure bicyclo[3.3. 1]nonane-2,6-dione. *Synthesis* **2009**, 864-867.

[13] Otomaru, Y., Tokunaga, N., Shintani, R., & Hayashi, T. (2005). C 2-Symmetric Bicyclo[3.3.1]nonadiene as a Chiral Ligand for Rhodium-Catalyzed Asymmetric Arylation of N-(4-Nitrobenzenesulfonyl) arylimines. *Org. Lett.* **2005**, *7*, 307–310.

[14] Abele, S.; Inauen, R.; Spielvogel, D.; Moessner, C. Scalable Synthesis of Enantiomerically Pure Bicyclo[2.2.2]octadiene Ligands. *J. Org. Chem.* **2012**, *77*, 4765–4773.

[15] Zhu, D. X.; Liu, J. G.; Xu, M. H. Stereodivergent synthesis of enantioenriched 2, 3-disubstituted dihydrobenzofurans via a one-pot C–H functionalization/oxa-Michael addition cascade. *J. Am. Chem. Soc.* **2021**, *143*, 8583–8589

[16] Chen, D.; Zhang, X.; Qi, W. Y.; Xu, B.; Xu, M. H. (2015). Rhodium (I)-catalyzed asymmetric carbene insertion into B–H bonds: highly enantioselective access to functionalized organoboranes. *J. Am. Chem. Soc.* **2015**, *137*, 5268–5271.

[17] Doyle, M. P., Austin, R. E., Bailey, A. S., Dwyer, M. P., Dyatkin, A. B., Kalinin, A. V., Kwan, M. M. Y.; Liras, S.; Oalmann, C. J.; Pieters, R. J.; Protopopova, M. N.; Raab, C. E.; Roos, G. H. P.; Zhou, Q.-L.; Martin, S. F. Enantioselective intramolecular cyclopropanations of allylic and homoallylic diazoacetates and diazoacetamides using chiral dirhodium (II) carboxamide catalysts. *J. Am. Chem. Soc.* **1995**, *117*, 5763-5775.

[18] Abu-Elfotoh, A. M.; Phomkeona, K.; Shibatomi, K.; Iwasa, S. Asymmetric inter-and intramolecular cyclopropanation reactions catalyzed by a reusable macroporous-polymer-supported chiral ruthenium (II)/phenyloxazoline complex. *Angew. Chem. Int. Ed.* **2010**, *49*, 8439-8443.

[19] Baker, W.R.; Martin, S. F. Renin inhibiting compounds. Patent WO9200972 A1.

[20] Abu-Elfotoh, A. M.; Nguyen, D. P. T.; Chanthamath, S.; Phomkeona, K.; Shibatomi, K.; & Iwasa, S. Water-Soluble Chiral Ruthenium (II) Phenyloxazoline Complex: Reusable and Highly Enantioselective Catalyst for Intramolecular Cyclopropanation Reactions. *Adv. Synth. Catal.* **2012**, *354*, 3435-3439.

[21] Xu, Z. J.; Fang, R.; Zhao, C.; Huang, J. S.; Li, G. Y.; Zhu, N.; Che, C. M. *cis*-β-Bis(carbonyl) ruthenium−salen complexes: X-ray crystal structures and remarkable catalytic properties toward asymmetric intramolecular alkene cyclopropanation. *J. Am. Chem. Soc.* **2009**, *131*, 4405–4417.

[22] Langlotz, B. K.; Wadepohl, H.; Gade, L. H. Chiral bis(pyridylimino)isoindoles: a highly modular class of pincer ligands for enantioselective catalysis. *Angew. Chem. Int. Ed.* **2008**, *47*, 4670–4674.

[23] Clemenceau, A.; Thesmar, P.; Gicquel, M.; Le Flohic, A.; Baudoin, O. Direct synthesis of cyclopropanes from gem-dialkyl groups through double C–H activation. *J. Am. Chem. Soc.* **2020**, *142*, 15355–15361.

[24] Mo, F.; Yan, J. M.; Qiu, D.; Li, F.; Zhang, Y.; Wang, J. Gold-Catalyzed Halogenation of Aromatics by N-Halosuccinimides. *Angew. Chem. Int. Ed.* **2010**,49, 2028 –2032.

[25] Chandgude, A. L.; Ren, X.; Fasan, R. Stereodivergent intramolecular cyclopropanation enabled by engineered carbene transferases. *J. Am. Chem. Soc.* **2019**, *141*, 9145-9150.

[26] Dolomanov, O.V.; Bourhis, L.J.; Gildea, R.J, Howard, J.A.K.; Puschmann, H. OLEX2: a complete structure solution, refinement and analysis program. *J. Appl. Cryst.* **2009**, *42*, 339–341.

[27] Sheldrick, G.M. SHELXT–Integrated space-group and crystal-structure determination. *Acta Cryst.* **2015**, *C71*, 3-8.

[28] Sheldrick, G.M. Crystal structure refinement with SHELXL. *Acta Cryst*. **2008**, *A64*, 112-122.
[29] Parsons, S. Determination of absolute configuration using X-ray diffraction. *Tetrahedron: Asymmetry* **2017**, *28*, 1304-1313.
[30] Bourhis, L.J.; Dolomanov, O.V.; Gildea, R.J.; Howard, J.A.K.; Puschmann, H. The anatomy of a comprehensive constrained, restrained refinement program for the modern computing environment–Olex2 dissected. *Acta Cryst.* **2015**, *A71*, 59-75.
[31] Frisch, M. J.; Trucks, G. W.; Schlegel, H. B.; Scuseria, G. E.; Robb, M. A.; Cheeseman, J. R.; Scalmani, G.; Barone, V.; Petersson, G. A.; Nakatsuji, H.; Li, X.; Caricato, M.; Marenich, A. V.; Bloino, J.; Janesko, B. G.; Gom-perts, R.; Mennucci, B.; Hratchian, H. P.; Ortiz, J. V.; Izmaylov, A. F.; Sonnenberg, J. L.; Williams; Ding, F.; Lipparini, F.; Egidi, F.; Goings, J.; Peng, B.; Petrone, A.; Henderson, T.; Ranasinghe, D.; Zakrzewski, V. G.; Gao, J.; Rega, N.; Zheng, G.; Liang, W.; Hada, M.; Ehara, M.; Toyota, K.; Fukuda, R.; Hasegawa, J.; Ishida, M.; Nakajima, T.; Honda, Y.; Kitao, O.; Nakai, H.; Vreven, T.; Throssell, K.; Montgomery Jr., J. A.; Peralta, J. E.; Ogliaro, F.; Bearpark, M. J.; Heyd, J. J.; Brothers, E. N.; Kudin, K. N.; Staroverov, V. N.; Keith, T. A.; Kobayashi, R.; Normand, J.; Raghavachari, K.; Rendell, A. P.; Burant, J. C.; Iyengar, S. S.; Tomasi, J.; Cossi, M.; Millam, J. M.; Klene, M.; Adamo, C.; Cammi, R.; Ochterski, J. W.; Martin, R. L.; Morokuma, K.; Farkas, O.; Foresman, J. B.; Fox, D. J. Gaussian 16, Rev. A.03, Wallingford, CT, 2016.
[32] (a) Zhao, Y.; Truhlar, D. G. The M06 Suite of Density Functionals for Main Group Thermochemistry, Thermochemical Kinetics, Noncovalent Interactions, Excited States, and Transition Elements: Two New Functionals and Systematic Testing of Four M06-Class Functionals and 12 Other Functionals. *Theor. Chem. Acc*. **2008**, *120*, 215−241. (b) Zhao, Y.; Truhlar, D. G. Density Functionals with Broad Applicability in Chemistry. *Acc. Chem. Res*. **2008**, *41*, 157−167.
[33] Weigend, F.; Ahlrichs, R. Balanced Basis Sets of Split Valence, Triple Zeta Valence and Quadruple Zeta Valence Quality for H to Rn: Design and Assessment of Accuracy. *Phys. Chem. Chem. Phys*. **2005**, *7*, 3297−3305.
[34] Grimme, S.; Antony, J.; Ehrlich, S.; Krieg, H. A Consistent and Accurate Ab Initio Parametrization of Density Functional Dispersion Correction (DFT-D) for the 94 Elements H-Pu. *J. Chem. Phys*. **2010**, *132*, 154104.
[35] (a) Zhao, Y.; Truhlar, D. G. The M06 Suite of Density Functionals for Main Group Thermochemistry, Thermochemical Kinetics, Noncovalent Interactions, Excited States, and Transition Elements: Two New Functionals and Systematic Testing of Four M06-Class Functionals and 12 Other Functionals. *Theor. Chem. Acc*. **2008**, *120*, 215−241. (b) Zhao, Y.; Truhlar, D. G. Density Functionals with Broad Applicability in Chemistry. *Acc. Chem. Res*. **2008**, *41*, 157−167.
[36] Weigend, F. Accurate Coulomb-Fitting Basis Sets for H to Rn. *Phys. Chem. Chem. Phys*. **2006**, *8*, 1057−1065.
[37] Marenich, A. V.; Cramer, C. J.; Truhlar, D. G. Universal Solvation Model Based on Solute Electron Density and on a Continuum Model of the Solvent Defined by the Bulk Dielectric Constant and Atomic Surface Tensions. *J. Phys. Chem. B*. **2009**, *113*, 6378−6396.
[38] Lefebvre, C.; Rubez, G.; Khartabil, H.; Boisson, J.-C.; Contreras-García, J.; Hénon, E. Accurately Extracting the Signature of Intermolecular Interactions Present in the NCI Plot of the Reduced Density Gradient versus Electron Density. *Phys. Chem. Chem. Phys.* **2017,** 19, 17928–17936.
[39] Lu, T.; Chen, F. Multiwfn: A multifunctional wavefunction analyzer. *J. Comput. Chem*. **2012**, *33*, 580–592.
[40] Humphrey, W.; Dalke, A.; Schulten, K. VMD: Visual molecular dynamics. *J. Mol. Graph. 1996,* **14**, 33–38.
[41] Legault, C. Y. *CYLview*, version 1.0b; Université de Sherbrooke, 2009 (http://www.cylview.org).
[42] Kelly, C. P.; Cramer, C. J.; Truhlar, D. G. SM6: A Density Functional Theory Continuum Solvation Model for Calculating Aqueous Solvation Free Energies of Neutrals, Ions, and Solute-Water Clusters. *J. Chem. Teory Comput*. **2005**, *1*, 1133–1152. (b) Kelly, C. P.; Cramer, C. J.; Truhlar, D. G. Aqueous Solvation Free Energies of Ions and Ion−Water Clusters Based on an Accurate Value for the Absolute Aqueous Solvation Free Energy of the Proton. *J. Phys. Chem. B*. **2006**, *110*, 16066–16081.
[43] For early studies relate to distortion/interaction analysis, see: (a) Kitaura, K.; Morokuma, K. A New Energy Decomposition Scheme for Molecular Interactions within the Hartree-Fock Approximation. *Int. J. Quantum Chem*. **1976**, *10*, 325−340. (b) Ziegler, T.; Rauk, A. A Theoretical Study of the Ethylene-Metal Bond in Complexes between Copper(1+), Silver(1+), Gold(1+), Platinum(0) or Platinum(2+) and Ethylene, Based on the Hartree-Fock-Slater Transition-State Method. *Inorg. Chem*. **1979**, *18*, 1558−1565.
[44] For reviews of distortion/interaction analysis, see: (a) van Leeuwen, P. W. N. M.; Kamer, P. C. J.; Reek, J. N. H.; Dierkes, P. Ligand Bite Angle Effects in Metal-catalyzed C−C Bond Formation. *Chem. Rev*. **2000**, *100*, 2741−2770. (b) van Zeist, W.-J.; Bickelhaupt, F. M. The Activation Strain Model of Chemical Reactivity. *Org. Biomol. Chem*. **2010**, *8*, 3118−3127. (c) Fernández, I.; Bickelhaupt, F. M. The Activation Strain Model and Molecular Orbital Theory: Understanding and Designing Chemical Reactions. *Chem. Soc. Rev*. **2014**, *43*, 4953−4967. (d) Bickelhaupt, F. M.; Houk, K. N. Analyzing Reaction Rates with the Distortion/Interaction-Activation Strain Model. *Angew. Chem., Int. Ed*. **2017**, *56*, 10070−10086
